# Tigray War and HIV Care Cascade Entry in Mekelle, Ethiopia

**DOI:** 10.1001/jamanetworkopen.2026.25130

**Published:** 2026-07-24

**Authors:** Hafte Kahsay Kebede, Hailay Abrha Gesesew, Lillian Mwanri, Paul Ward

**Affiliations:** 1 Torrens University Australia, Adelaide, South Australia, Australia; 2 Tigray Health Research Institute, Mekelle, Tigray, Ethiopia; 3 School of Society and Culture, Adelaide University, Adelaide, South Australia, Australia

## Abstract

**Importance:**

Armed conflicts disrupt health care delivery, yet quantitative evidence on conflict-related HIV care cascade disruptions in sub-Saharan Africa remains limited.

**Objective:**

To examine associations between the Tigray War and HIV diagnosis, linkage to care, and antiretroviral therapy (ART) initiation in Mekelle City, Tigray, Ethiopia.

**Design, Setting, and Participants:**

This cohort study used an interrupted time-series analysis using quarterly electronic medical record data from 7 health facilities in Mekelle City, Tigray, Ethiopia (quarter [Q] 1 2005 to Q1 2025; 81 quarters; 2020 retained as a separate pandemic period). All individuals accessing HIV care services at participating facilities were included. Data were analyzed from October 2024 to May 2025.

**Exposure:**

Four study periods: prewar (Q1 2005 to Q4 2019; reference), pandemic (Q1 to Q4 2020), war (Q1 2021 to Q4 2022), and postwar (Q1 2023 to Q1 2025).

**Main Outcomes and Measures:**

Quarterly counts of HIV diagnoses, linkages to care, and ART initiations, modeled using negative binomial segmented regression with seasonal harmonics. Autoregression with integrated moving averages with intervention regressors served as a complementary analysis.

**Results:**

Over the study period, 13 478 HIV diagnoses, 11 959 linkages to care, and 9399 ART initiations were recorded; women represented 7913 diagnoses (58.7%), and individuals aged 35 to 44 years had the most diagnoses (4414 [32.7%]). During the pandemic, HIV diagnoses declined relative to prewar trajectories (incidence rate ratio [IRR], 0.65; 95% CI, 0.51-0.83; *P* < .001). During the war, HIV diagnoses declined further (IRR, 0.71; 95% CI, 0.58-0.86; *P* < .001), with comparable reductions in linkages to care (IRR, 0.68; 95% CI, 0.53-0.86; *P* = .002) and ART initiations (IRR, 0.70; 95% CI, 0.54-0.91; *P* = .008). Women experienced larger war-period reductions than men (diagnoses: IRR, 0.57; 95% CI, 0.46-0.72 vs IRR, 0.92; 95% CI, 0.74-1.15). Individuals aged 0 to 24 years showed the largest declines in diagnoses (IRR, 0.59; 95% CI, 0.43-0.81). Secondary facilities showed war-period increases in diagnoses (IRR, 1.44; 95% CI, 1.09-1.90), while tertiary and primary facilities showed reductions (tertiary: IRR, 0.18; 95% CI, 0.10-0.32; *P* < .001; primary: IRR, 0.29; 95% CI, 0.17-0.50; *P* < .001). The nongovernmental organization–managed facility showed war-period and postwar IRRs of 0.21 (95% CI, 0.13-0.36) and 0.20 (95% CI, 0.12-0.35), with no recovery.

**Conclusions and Relevance:**

In this cohort study of HIV care services in Mekelle, Ethiopia, the Tigray War period was associated with substantial declines in HIV diagnoses, linkages to care, and ART initiations, with services remaining below counterfactual expectations more than 2 years after the ceasefire.

## Key Points

**Question:**

Was the Tigray War (November 2020 to November 2022) associated with changes in HIV diagnoses, linkage to care, and antiretroviral therapy (ART) initiation in Mekelle, Ethiopia?

**Findings:**

In this cohort study using 20 years of routine clinical data from 7 health facilities in Mekelle and examining 13 478 HIV diagnoses, the war period was associated with reductions of approximately 29% to 32% in HIV diagnoses, linkages to care, and ART initiations, with the largest reductions among women, individuals aged 0 to 24 years, and patients treated at the single nongovernmental organization (NGO)–managed facility, where postwar values remained close to war-period levels.

**Meaning:**

In this study, armed conflict was associated with severe, sustained, and inequitable disruptions to HIV care entry in Tigray, suggesting that conflict-sensitive HIV programming should specifically attend to women, youths, and NGO-supported facilities.

## Teaser

This cohort study examines whether the Tigray War was associated with HIV diagnosis, linkage to care, and provision of antiretroviral therapy in Mekelle City, Ethiopia.

## Introduction

Armed conflict represents one of the most severe threats to population health and health care delivery globally. Approximately 1.9 to 2.0 billion people—about a quarter of the global population—live in fragile or conflict-affected settings, underscoring the scale of vulnerability worldwide.^1^ Such settings frequently experience health system disruption or collapse, undermining capacity to deliver essential services, including HIV prevention, testing, and treatment.^2,3^ The intersection of conflict and HIV/AIDS creates a particularly dangerous synergy: armed conflict has been associated with disruption of health systems and social structures necessary for HIV prevention and treatment, while high HIV prevalence may exacerbate humanitarian consequences through increased morbidity and mortality.^3,4^

The global community has committed to the Joint United Nations Programme on HIV/AIDS (UNAIDS) 95-95-95 targets: 95% of people living with HIV knowing their status, 95% of diagnosed individuals receiving antiretroviral therapy (ART), and 95% of those receiving ART achieving viral suppression.^5^ However, these targets prove substantially harder to achieve in fragile or conflict-affected settings. In Ethiopia, approximately 505 000 people received ART in 2022, but disruption of donor funding threatens long-term target attainment.^6,7^

The Tigray conflict began in early November 2020 and continued until a ceasefire agreement mediated by the African Union took effect on November 3, 2022.^8,9,10,11^ Within 6 months, the region’s health system suffered catastrophic losses: only 30% of hospitals, 17% of health centers, and 12% of ambulances remained functional by June 2021, while none of the 712 health posts were operational.^10^ International agencies documented that blockade and restricted access substantially impeded delivery of health services and supplies.^12,13^ Mass displacement compounded these challenges, with approximately 2.5 million internally displaced persons in Tigray alone by mid-2021.^14,15,16^

Health infrastructure destruction severely undermined HIV services. A facility-based study documented that patients with HIV attending follow-up visits dropped from 3274 and 3298 in September and October 2020 (prewar) to just 847 (approximately 25%) in January 2021, declining further to only 331 (approximately 17%) by May 2021.^17^ Despite this well-documented devastation, rigorous quantitative evidence of conflict associations with HIV service delivery remains scarce. In our recent systematic review,^3^ we identified substantial research gaps linking armed conflict to HIV treatment outcomes in sub-Saharan Africa, noting inadequate study designs, limited data sources, and insufficient attention to the HIV care cascade.

This study addresses these evidence gaps by conducting an interrupted time-series analysis of HIV care cascade trajectories during and after the Tigray conflict. Using 20 years of electronic medical record data spanning the prewar, pandemic, war, and postwar periods from 7 health facilities in Mekelle City, we estimate level changes in HIV diagnoses, linkages to care, and ART initiations across the pandemic, war, and postwar periods compared with prewar trajectories and examine differential associations across sex, age groups, facility levels, and ownership types.

## Methods

### Study Design and Ethical Approval

For this cohort study, we conducted an interrupted time-series (ITS) analysis using quarterly aggregated facility-level HIV routine clinical data, complemented by segmented regression modeling and counterfactual projection. ITS is a robust quasi-experimental design well-suited for evaluating population-level discontinuities when randomization is neither feasible nor ethical.^18^ This approach enables assessment of level changes following an interruption, while establishing a counterfactual trajectory representing expected outcomes in the absence of the disruption. Ethical approval was obtained from the institutional review board of Tigray Health Research Institute (THRI/4031/0502/16) and the Torrens University Australia Human Research Ethics Committee (Ethics Application 0333). The Tigray Region Health Bureau granted permission (Ref No. 2579/365/16). Because data were retrospectively extracted in deidentified form, the requirement for individual informed consent was waived by both review boards. The study was conducted in accordance with the Strengthening the Reporting of Observational Studies in Epidemiology (STROBE) reporting guideline.

### Setting and Participants

The study was conducted in Mekelle City, the capital of Tigray Region, northern Ethiopia. Mekelle (population approximately 559 000)^19^ maintained a relatively concentrated HIV care infrastructure compared with rural Tigray, where conflict-related facility damage was more severe.^13,17^ According to the Tigray Region Health Bureau, 24 health facilities provided routine HIV care services in Mekelle City before the war. After excluding 4 privately owned facilities that declined participation and 13 facilities meeting 1 of 2 documented exclusion criteria (11 using Health Management Information System [HMIS] rather than SmartCare for HIV records; 2 having migrated from SmartCare to another data system since February 2024), 7 facilities were eligible for analysis: 1 tertiary referral hospital, 2 secondary general hospitals, and 4 primary-level facilities (1 nongovernmental organization [NGO]–managed primary hospital, 2 health centers, and 1 NGO-managed clinic). Together, these facilities serve a catchment population exceeding 1 million and delivered an estimated 78% to 82% of prewar routine HIV care services in Mekelle based on 2018-2019 HMIS reports. The study population comprised all individuals accessing HIV care services at participating facilities during the study period, with no exclusions by age, sex, or clinical status.

### Data Sources and Measurement

This study utilized data from SmartCare, an electronic medical record platform supported by the President’s Emergency Plan for AIDS Relief (PEPFAR) deployed across health facilities in Mekelle City.^20^ The system maintains comprehensive longitudinal records of HIV care at the individual patient level. Between October 2024 and May 2025, deidentified clinical data were extracted from 7 health facilities that had maintained continuous SmartCare records throughout the study period.

The analytical timeframe spanned quarter [Q] 1 2005 through Q1 2025, yielding 81 quarterly observations per facility. Q2 2025 was excluded because it contained only 2 of 3 calendar months (April and May). The study timeline was divided into 4 periods: prewar (Q1 2005 to Q4 2019; 60 quarters; reference period), pandemic (Q1 to Q4 2020; 4 quarters), war (Q1 2021 to Q4 2022; 8 quarters), and postwar (Q1 2023 to Q1 2025; 9 quarters). The year 2020 was designated as a distinct pandemic period rather than excluded, allowing the war-period coefficient to be interpreted as the change beyond the pandemic baseline.

### Variables

Three key indicators along the HIV care cascade were analyzed. Each of the following were measured as quarterly aggregate counts: HIV diagnoses (number of individuals newly diagnosed with HIV through facility-based testing services during the quarter); linkages to care (number of HIV-positive individuals who enrolled in HIV care services at the diagnosing facility during the quarter in which they were diagnosed); and ART initiations (number of HIV-positive individuals who commenced antiretroviral therapy at the facility during the quarter of diagnosis; this represents incident treatment starts, not the prevalence of patients on treatment).

### Data Cleaning and Exclusions

Transfer-outs and transfer-ins were defined operationally from the SmartCare dataset. A transfer-out was recorded when a patient’s status was documented as transferred out, and a transfer-in was recorded when a patient’s status was documented as transferred in. These classifications were accepted as recorded without independent verification.

Transfer-ins were retained in the analysis. Transfer-outs were handled conditionally on cascade completion: patients who had completed all 3 cascade stages (HIV diagnosis, linkage to care, and ART initiation) at a study facility before transfer were retained; those transferred out after diagnosis but before completion of linkage or ART initiation were excluded. Patients lacking a unique ART identifier and those whose recorded dates violated the expected chronological sequence (date of birth, followed by HIV diagnosis, which was followed by or simultaneous to linkage to care and ART initiation), were additionally excluded.

### Statistical Analysis

We used interrupted time-series analysis with segmented regression to assess changes in each outcome. The primary model took the form log(*Y_t_*) = β_0_ + β_1_(time) + β_2_(pandemic) + β3(war) + β_4_(postwar) + β_5_sin(2π*t*/4) + β_6_cos(2π*t*/4) + ε_t_, where *Y*_t_ represents the count outcome at time *t*. All effect estimates are reported relative to the prewar period (reference period). The simple level-change specification, without time × period interactions, was chosen because the question of clinical interest is the average level shift in each period; a slope-change specification with time-after-war and time-after-postwar terms is reported as a sensitivity analysis (eTable 5 in Supplement 1).^18,21^

Specifically, the negative binomial count segmented regression model fitted to each cascade outcome was log(*E*[*Y_t_*]) = β_0_ + (β_1_ × *t*) + (β_2_ × pandemic_t_) + (β_3_ × war*_t_*) + (β_4_ × postwar*_t_*) + (β_5_ × sin[2π*t*/4]) + (β_6_ × cos[2π*t*/4]), where *t* is the quarter index (1-81); pandemic*_t_*, war*_t_*, and postwar*_t_* are 0 or 1 indicators for the corresponding analytical periods, and the sine-cosine pair captures annual seasonality at quarterly frequency. The complete coefficient table for the 3 overall count models, including the intercept, the linear time trend (β_1_), the period-specific level shifts (β_2_, β_3_, and β_4_), and the seasonal harmonic terms (β_5_ and β_6_) is reported in eTable 5A in Supplement 1. Because our outcomes were counts with overdispersion, we primarily used negative binomial regression models with a log-link function. Model convergence was verified through positive-definite Hessian matrices and valid parameter SEs.^22^ When negative binomial models failed to converge, quasi-Poisson regression models were used as fallbacks. Standard Poisson regression was not used as a fallback because it would understate uncertainty when overdispersion is present. Of the 36 count models fitted, 35 used negative binomial regression and 1 used quasi-Poisson regression. Model coefficients were exponentiated to obtain incidence rate ratios (IRRs) with 95% CIs.^23,24^

Counterfactual projections represent the trajectory the outcome was expected to follow in the absence of the pandemic and war shocks, holding all other model terms (linear time, seasonal harmonics) at their fitted values. Operationally, we set pandemic_dummy = 0, war_dummy = 0, and post_dummy = 0 for all post-2019 observations and re-estimated on the response scale. Cumulative cascade deficits were computed as the sum across war and postwar quarters of the difference between counterfactual estimation and observed value. Confidence intervals for cumulative deficits were obtained by parametric bootstrap (5000 replications) drawing from the negative binomial distribution implied by each fitted model.

We validated model adequacy using the R DHARMa package (package version 0.4.6 or later) for residual diagnostics. Temporal autocorrelation was assessed using DHARMa’s testTemporalAutocorrelation() function applied to ordered quantile residuals, which is appropriate for generalized linear models because the residuals are uniform under correct model specification; we did not rely on Durbin-Watson tests applied directly to Pearson residuals from generalized linear models, as those tests are not strictly valid in the generalized linear model context.^25^ Four prespecified sensitivity analyses assessed robustness: (1) removal of seasonal harmonic terms; (2) refitting with the slope-change specification (full model with time-after-war and time-after-postwar terms); (3) refitting with war onset moved to Q4 2020; and (4) restricting the prewar baseline to 2015 onward (post test-and-treat era, when Ethiopia adopted universal ART eligibility).^18,21^ As a complementary time-series approach, we additionally fitted autoregression with integrated moving averages (ARIMA) models with intervention regressors (auto.arima with xreg matrix of pandemic, war, and postwar indicators). To assess heterogeneity, we conducted stratified analyses by sex, age group (0-24, 25-34, 35-44, and ≥45 years; the cut points align with WHO age categories used in HIV surveillance reporting in sub-Saharan Africa and reflect the concentration of HIV epidemiology in those aged 25-44 years), facility level (primary, secondary, tertiary), and facility ownership (public vs NGO; with the caveat that our NGO stratum represents a single facility and findings should not be generalized to NGO-managed HIV programs broadly). To formally test effect modification, we fitted long-format models with period × stratifier interactions and likelihood-ratio tested the interaction terms. All analyses were conducted in R version 4.6.0 (R Project for Statistical Computing). Statistical significance was set at a 2-sided *P* < .05.

Comprehensive methodological description, full segmented regression coefficient tables, ARIMA outputs, sensitivity analyses, period × stratifier interaction tests, cumulative deficits, and DHARMa diagnostic plots are provided in the eTables (eTables 1-13 in Supplement 1, including the overall coefficient table eTable 5A and the full stratified coefficient table eTable 5B in Supplement 1), eFigures (eAppendix 1 and eFigures 1-12 in Supplement 1), and eMethods (eMethods 1-3 in Supplement 1). The complete R code (974 lines) is provided as eAppendix 2 in Supplement 1 for full reproducibility.

## Results

### Participant Characteristics

Between January 2005 and March 2025, 7 health facilities reported 13 478 HIV diagnoses, 11 959 linkages to care, and 9399 ART initiations across the 20-year study period (Table 1). Women represented the majority of individuals accessing services across all periods: 6977 prewar diagnoses (58.0%), 747 (51.5%) during war, and 449 (56.3%) postwar. Individuals aged 25 to 44 years represented the largest proportion of cases, with 4414 diagnoses (32.7%) among those aged 35 to 44 years. Public facilities delivered 11 293 prewar diagnoses (93.9%), whereas the NGO-managed facility increased its proportional contribution during war (180 [12.4%]) and postwar (112 [14.0%]) periods. Secondary-level facilities consistently provided more than half of all services across all periods.

**Table 1.** Characteristics of Individuals Accessing HIV Care Services by Study Period, Mekelle City, Tigray, Ethiopia, 2005-2025

Characteristic	No. (%)^a^			
	Prewar (2005-2019) (n = 12 027)	Pandemic (2020) (n = 303)	War (2021-2022) (n = 598)	Postwar (2023-2025) (n = 811)
**HIV diagnoses**				
Sex				
Female	6977 (58.0)	173 (57.1)	308 (51.5)	455 (56.1)
Male	5050 (42.0)	130 (42.9)	290 (48.5)	356 (43.9)
Age, y				
0-24	1925 (16.0)	48 (15.8)	89 (14.9)	148 (18.2)
25-34	2073 (17.2)	111 (36.7)	262 (43.9)	302 (37.2)
35-44	3862 (32.1)	121 (39.9)	198 (33.1)	233 (28.7)
≥45	2061 (17.1)	45 (14.9)	113 (18.9)	156 (19.2)
Facility level				
Primary	3104 (25.8)	123 (40.6)	156 (26.1)	254 (31.3)
Secondary	7040 (58.5)	119 (39.3)	325 (54.3)	413 (50.9)
Tertiary	1883 (15.7)	61 (20.1)	117 (19.6)	144 (17.8)
Ownership				
NGO	739 (6.1)	49 (16.2)	74 (12.4)	107 (13.2)
Public	11 288 (93.9)	254 (83.8)	524 (87.6)	704 (86.8)
**Linkage to care**				
Sex				
Female	6138 (58.0)	161 (57.5)	282 (51.6)	434 (55.2)
Male	4446 (42.0)	119 (42.5)	265 (48.4)	352 (44.8)
Age, y				
0-24	1720 (16.3)	47 (16.8)	85 (15.5)	140 (17.8)
25-34	2068 (19.5)	107 (38.3)	259 (47.4)	299 (38.1)
35-44	3417 (32.3)	118 (42.1)	178 (32.5)	226 (28.8)
≥45	1811 (17.1)	42 (15.0)	105 (19.2)	154 (19.6)
Facility level				
Primary	2779 (26.3)	119 (42.5)	145 (26.5)	250 (31.8)
Secondary	6249 (59.0)	109 (38.9)	298 (54.5)	397 (50.5)
Tertiary	1556 (14.7)	52 (18.6)	104 (19.0)	139 (17.7)
Ownership				
NGO	672 (6.3)	48 (17.1)	72 (13.2)	107 (13.6)
Public	9912 (93.7)	232 (82.9)	475 (86.8)	679 (86.4)
**ART initiation**				
Sex				
Female	4547 (56.6)	160 (57.8)	280 (51.7)	432 (55.5)
Male	3490 (43.4)	117 (42.2)	262 (48.3)	346 (44.5)
Age, y				
0-24	1347 (16.8)	47 (17.0)	85 (15.7)	140 (18.0)
25-34	2335 (29.1)	108 (39.2)	262 (48.4)	302 (38.9)
35-44	2715 (33.8)	118 (42.6)	178 (32.8)	226 (29.0)
≥45	1416 (17.6)	42 (15.2)	105 (19.4)	154 (19.8)
Facility level				
Primary	1966 (24.5)	117 (42.2)	142 (26.2)	250 (32.1)
Secondary	4541 (56.5)	108 (39.0)	297 (54.8)	395 (50.8)
Tertiary	1530 (19.0)	52 (18.8)	103 (19.0)	133 (17.1)
Ownership				
NGO	451 (5.6)	47 (17.0)	69 (12.7)	107 (13.8)
Public	7586 (94.4)	230 (83.0)	473 (87.3)	671 (86.2)

Abbreviations: ART, antiretroviral therapy; NGO, nongovernmental organization.

^a^ Counts represent newly diagnosed individuals (HIV diagnoses), newly diagnosed individuals enrolled in care (linkage to care), or newly diagnosed individuals starting antiretroviral therapy (ART initiation) within each period. Percentages are within-period proportions of the characteristic total. The prewar period included 60 quarters; pandemic, 4 quarters of 2020; war, 8 quarters (quarter 1 2021 to quarter 4 2022); postwar: 9 quarters (quarter 1 2023 to quarter 1 2025; quarter 2 2025 excluded as it contained only 2 of 3 calendar months at data extraction).

### Prewar Trends

**Figure 1.**
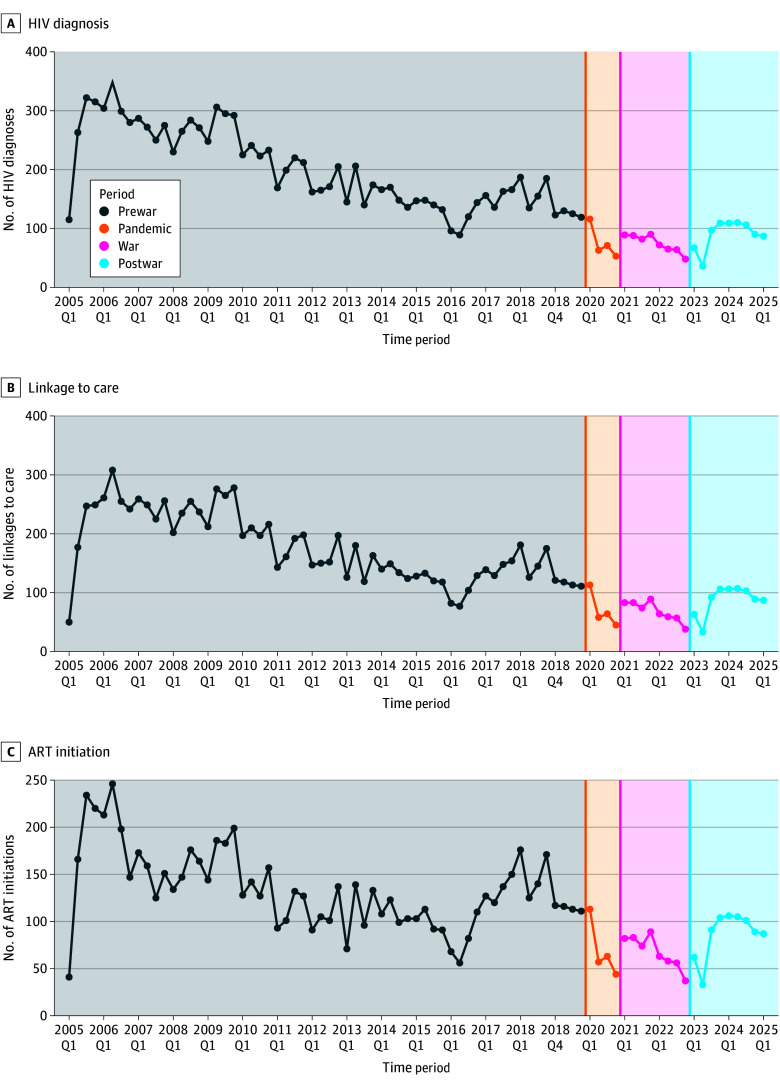
Line Graphs Showing Quarterly Trends in HIV Care Cascade Outcomes Across Study Periods, Mekelle City, Tigray, Ethiopia, Quarter (Q) 1 2005 to Q1 2025 ART indicates antiretroviral therapy.

During the 60-quarter prewar period, HIV service delivery demonstrated established but slowly declining temporal patterns (Figure 1). HIV diagnoses showed a modest decrease over time (IRR per quarter, 0.98; 95% CI, 0.98-0.99; *P* < .001), representing approximately 2% quarterly decline. Similar declining patterns were observed for linkages to care (IRR, 0.99; 95% CI, 0.98-0.99; *P* < .001) and ART initiations (IRR, 0.99; 95% CI, 0.99-1.00; *P* = .048). The mean (SD) prewar service volumes were 200.5 (85.3) HIV diagnoses per quarter, 176.4 (72.8) linkages to care per quarter, and 133.9 (62.1) ART initiations per quarter.

Full segmented regression coefficients from the count models confirmed the declining prewar secular trend (diagnoses: time-slope IRR per quarter, 0.98; 95% CI, 0.98-0.99; linkage to care: IRR per quarter, 0.99; 0.98-0.99; ART initiation: IRR per quarter, 0.99; 95% CI, 0.99-1.00; all *P* < .001) and showed small, nonsignificant seasonal harmonic terms (sin and cos IRRs near 1.0) (eTable 5A in Supplement 1). These results indicate that the period-level shifts reported for the pandemic, war, and postwar periods are estimated relative to a counterfactual that already accounts for the underlying secular trend and quarterly seasonality.

### Pandemic Period

During the 4 quarters of 2020, HIV diagnoses were significantly lower than the prewar trajectory (IRR, 0.65; 95% CI, 0.51-0.83; *P* < .001), with comparable reductions in linkages to care (IRR, 0.64; 95% CI, 0.48-0.86; *P* = .003) and ART initiations (IRR, 0.68; 95% CI, 0.50-0.94; *P* = .02). These pandemic-period reductions are interpreted as the change relative to the prewar period, adjusting for linear time and seasonality, and indicate measurable suppression of cascade entry during the COVID-19 period that is independent of the war-period changes reported in the next section.

### War-Period Changes

**Figure 2.**
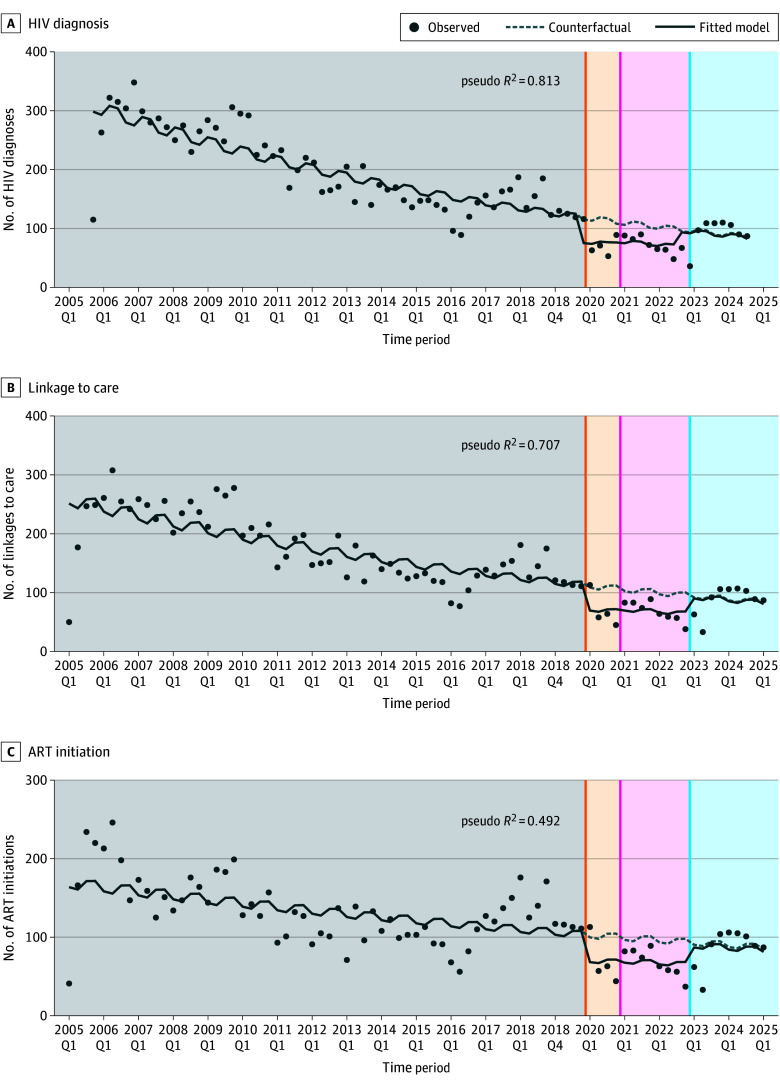
Line Graphs Showing Interrupted Time-Series Analysis of HIV Care Cascade Outcomes, Mekelle City, Tigray, Ethiopia, Quarter (Q) 1 2005 to Q1 2025 Negative binomial regression models with 4 periods (prewar, pandemic, war, postwar). Dark blue dots indicate observed quarterly counts aggregated across 7 facilities. Solid lines indicate fitted values. Dashed lines indicate counterfactual projections of the prewar trend. The gap between the fitted and counterfactual lines during the war and postwar periods represents the estimated conflict impact. ART indicates antiretroviral therapy.

War onset was associated with further reductions across all HIV care cascade stages (Table 2 and Figure 2). HIV diagnoses declined by approximately 29% relative to prewar trajectories (IRR, 0.71; 95% CI, 0.58-0.86; *P* < .001). Linkage to care showed a 32% reduction (IRR, 0.68; 95% CI, 0.53-0.86; *P* = .002), and ART initiations declined 30% (IRR, 0.70; 95% CI, 0.54-0.91; *P* = .008). The war-period reductions are in addition to the pandemic-period reductions, indicating that conflict-related changes exceeded what would be expected from the pandemic alone. In absolute terms, war-period quarterly means (SD) were 74.8 (15.1) diagnoses, 68.4 (17.1) linkages, and 67.8 (17.4) ART initiations. Cumulative deficits summed across the war and postwar quarters, defined as the integrated difference between observed and counterfactual trajectories under our segmented regression model, were 265 diagnoses (95% CI, 7 to 544), 276 linkages (95% CI, −38 to 610), and 264 ART initiations (95% CI, −68 to 624). These estimates assume no concurrent unmeasured shocks during the war and postwar periods.

**Table 2.** Interrupted Time-Series Analysis of HIV Care Cascade Disruptions During the Tigray War, 2005-2025^a^

Characteristic	Family	AICc	Prewar trend, IRR (95% CI)	*P* value	Pandemic, IRR (95% CI)	*P* value	War, IRR (95% CI)	*P* value	Postwar, IRR (95% CI)	*P* value
**HIV diagnoses**											
Overall	NB	807.4	0.98 (0.98-0.99)	<.001	0.65 (0.51-0.83)	<.001	0.71 (0.58-0.86)	<.001	0.98 (0.80-1.21)	.86
Sex										
Female	NB	731.8	0.99 (0.98-0.99)	<.001	0.60 (0.45-0.78)	<.001	0.57 (0.46-0.72)	<.001	0.85 (0.68-1.07)	.18
Male	NB	680.0	0.98 (0.98-0.98)	<.001	0.74 (0.56-0.97)	.03	0.92 (0.74-1.15)	.47	1.19 (0.95-1.49)	.13
Age, y										
0-24	NB	575.2	0.99 (0.98-0.99)	<.001	0.59 (0.40-0.88)	.009	0.59 (0.43-0.81)	.001	0.99 (0.72-1.34)	.93
25-34	QP	NA	1.00 (1.00-1.00)	.30	0.78 (0.66-0.92)	.004	0.91 (0.81-1.04)	.17	0.92 (0.81-1.05)	.24
35-44	NB	628.4	0.98 (0.98-0.99)	<.001	0.84 (0.64-1.09)	.18	0.76 (0.61-0.94)	.01	0.92 (0.73-1.15)	.46
≥45	NB	560.6	0.98 (0.98-0.99)	<.001	0.60 (0.41-0.86)	.006	0.83 (0.64-1.09)	.19	1.20 (0.91-1.57)	.19
Facility level										
Primary	NB	743.8	1.01 (1.00-1.02)	.12	0.48 (0.26-0.92)	.03	0.29 (0.17-0.50)	<.001	0.39 (0.22-0.71)	.002
Secondary	NB	748.9	0.97 (0.96-0.97)	<.001	0.86 (0.61-1.22)	.41	1.44 (1.09-1.90)	.009	2.16 (1.62-2.87)	<.001
Tertiary	NB	656.0	1.03 (1.02-1.04)	<.001	0.22 (0.12-0.43)	<.001	0.18 (0.10-0.32)	<.001	0.16 (0.09-0.29)	<.001
Ownership										
NGO	NB	521.5	1.04 (1.03-1.05)	<.001	0.37 (0.20-0.66)	<.001	0.21 (0.13-0.36)	<.001	0.20 (0.12-0.35)	<.001
Public	NB	801.7	0.98 (0.98-0.98)	<.001	0.65 (0.50-0.84)	.001	0.74 (0.60-0.92)	.006	1.05 (0.84-1.31)	.65
**Linkages to care**											
Overall	NB	822.6	0.99 (0.98-0.99)	<.001	0.64 (0.48-0.86)	.003	0.68 (0.53-0.86)	.002	0.98 (0.76-1.26)	.88
Sex										
Female	NB	748.6	0.99 (0.98-0.99)	<.001	0.58 (0.42-0.81)	.001	0.55 (0.42-0.72)	<.001	0.83 (0.63-1.11)	.21
Male	NB	686.7	0.98 (0.98-0.99)	<.001	0.72 (0.53-0.99)	.04	0.89 (0.69-1.14)	.36	1.22 (0.94-1.58)	.13
Age, y										
0-24	NB	570.6	0.99 (0.99-1.00)	<.001	0.53 (0.35-0.80)	.003	0.50 (0.36-0.70)	<.001	0.79 (0.57-1.10)	.16
25-34	NB	496.2	1.00 (1.00-1.00)	.60	0.76 (0.62-0.94)	.01	0.92 (0.78-1.08)	.29	0.94 (0.79-1.11)	.43
35-44	NB	639.3	0.98 (0.98-0.99)	<.001	0.87 (0.65-1.18)	.38	0.72 (0.56-0.93)	.01	0.93 (0.72-1.21)	.59
≥45	NB	569.7	0.98 (0.98-0.99)	<.001	0.59 (0.39-0.89)	.01	0.81 (0.59-1.11)	.18	1.21 (0.88-1.65)	.25
Facility level										
Primary	NB	742.3	1.01 (1.00-1.02)	.04	0.47 (0.23-0.95)	.04	0.26 (0.14-0.49)	<.001	0.37 (0.19-0.71)	.003
Secondary	NB	749.1	0.97 (0.96-0.97)	<.001	0.84 (0.57-1.23)	.36	1.38 (1.02-1.87)	.04	2.15 (1.57-2.95)	<.001
Tertiary	NB	632.5	1.03 (1.02-1.04)	<.001	0.21 (0.11-0.42)	<.001	0.18 (0.10-0.32)	<.001	0.16 (0.08-0.29)	<.001
Ownership										
NGO	NB	516.5	1.04 (1.03-1.06)	<.001	0.34 (0.18-0.65)	.001	0.19 (0.11-0.34)	<.001	0.17 (0.09-0.32)	<.001
Public	NB	815.7	0.98 (0.98-0.99)	<.001	0.63 (0.46-0.86)	.004	0.71 (0.55-0.92)	.009	1.05 (0.80-1.38)	.71
**ART initiations**											
Overall	NB	806.6	0.99 (0.99-1.00)	<.001	0.68 (0.50-0.94)	.02	0.70 (0.54-0.91)	.008	0.96 (0.73-1.26)	.78
Sex										
Female	NB	723.9	0.99 (0.99-1.00)	.01	0.63 (0.44-0.89)	.009	0.56 (0.42-0.75)	<.001	0.81 (0.60-1.09)	.16
Male	NB	680.5	0.99 (0.98-0.99)	<.001	0.77 (0.54-1.09)	.14	0.92 (0.70-1.22)	.57	1.21 (0.90-1.62)	.20
Age, y										
0-24	NB	547.5	1.00 (1.00-1.01)	.66	0.51 (0.34-0.77)	.001	0.46 (0.32-0.64)	<.001	0.66 (0.47-0.92)	.01
25-34	NB	524.7	1.00 (1.00-1.00)	.83	0.70 (0.57-0.87)	<.001	0.85 (0.73-0.99)	.04	0.87 (0.74-1.02)	.10
35-44	NB	618.5	0.99 (0.99-0.99)	<.001	0.90 (0.66-1.22)	.49	0.72 (0.55-0.92)	.01	0.88 (0.68-1.14)	.34
≥45	NB	557.6	0.99 (0.99-0.99)	<.001	0.62 (0.40-0.96)	.03	0.83 (0.59-1.15)	.26	1.18 (0.84-1.65)	.34
Facility level										
Primary	NB	685.5	1.02 (1.01-1.03)	<.001	0.52 (0.28-0.97)	.04	0.28 (0.17-0.48)	<.001	0.37 (0.21-0.65)	<.001
Secondary	NB	743.9	0.97 (0.97-0.98)	<.001	0.91 (0.58-1.43)	.68	1.46 (1.02-2.09)	.04	2.16 (1.49-3.13)	<.001
Tertiary	NB	630.7	1.03 (1.02-1.04)	<.001	0.22 (0.11-0.44)	<.001	0.18 (0.10-0.33)	<.001	0.15 (0.08-0.29)	<.001
Ownership										
NGO	NB	430.0	1.05 (1.04-1.06)	<.001	0.48 (0.30-0.76)	.002	0.27 (0.18-0.40)	<.001	0.25 (0.16-0.38)	<.001
Public	NB	797.5	0.99 (0.99-0.99)	<.001	0.66 (0.47-0.92)	.02	0.72 (0.55-0.94)	.02	1.00 (0.75-1.33)	>.99

Abbreviations: AICc, corrected Akaike information criterion; ART, antiretroviral therapy; IRR, incidence rate ratio; NA, not applicable; NB, negative binomial; NGO, nongovernmental organization; QP, quasi-Poisson.

^a^ NB or QP segmented regression with seasonal harmonics. Full coefficient table for the 3 overall outcomes is provided as eTable 5A in Supplement 1; complete stratum-level IRRs and *P* values are in eTable 5 in Supplement 1.

The full publication-style table of period-specific IRRs across all outcomes and strata is provided as eTable 5 in Supplement 1. Cumulative deficits with parametric bootstrap 95% CIs (5000 replications) are detailed in eTable 11 in Supplement 1.

### Postwar Trends

Following the November 2022 ceasefire, HIV services showed no statistically significant change relative to the prewar period for diagnoses (IRR, 0.98; 95% CI, 0.80-1.21; *P* = .86), linkages to care (IRR, 0.98; 95% CI, 0.76-1.26; *P* = .88), or ART initiations (IRR, 0.96; 95% CI, 0.73-1.27; *P* = .78). These postwar IRRs indicate that, after adjusting for linear time and seasonality, postwar service volumes had returned to levels consistent with prewar trajectories on average; however, the cumulative deficits accrued during the war period had not been recovered.

### Subgroup Analyses

Stratified analyses revealed substantial heterogeneity across population subgroups (Table 2). Women experienced larger war-period reductions than men. HIV diagnoses declined significantly among women (IRR, 0.57; 95% CI, 0.46-0.72; *P* < .001) but not among men (IRR, 0.92; 95% CI, 0.74-1.15; *P* = .47), with similar patterns for linkages to care and ART initiations. Interaction analyses confirmed significant effect modification by sex (*P* < .001 for war × sex interaction for diagnoses).

Age-stratified analyses showed that individuals aged 0 to 24 years experienced the largest war-period reductions for diagnoses (IRR, 0.59; 95% CI, 0.43-0.81; *P* = .001), with comparable reductions in linkages to care and ART initiations. Those aged 25 to 34 years showed smaller, nonsignificant war-period changes (diagnoses IRR, 0.91; 95% CI, 0.81-1.04; *P* = .17). Adults aged 35 to 44 years showed significant war-period declines (diagnoses: IRR, 0.76; 95% CI, 0.61-0.94; *P* = .01).

Facility-level analyses revealed marked differences by both facility level and ownership. Primary health care facilities experienced 71% war-period declines (IRR, 0.29; 95% CI, 0.17-0.50; *P* < .001) across all indicators without recovery (postwar IRR, 0.39; 95% CI, 0.22-0.71; *P* = .002). Tertiary facilities showed similarly large reductions (war-period IRR, 0.18; 95% CI, 0.10-0.32; *P* < .001). In contrast, secondary-level general hospitals showed war-period increases (diagnoses: IRR, 1.44; 95% CI, 1.09-1.90; *P* = .009) and continued elevation in the postwar period (IRR, 2.16; 95% CI, 1.62-2.87; *P* < .001), consistent with redistribution of services from primary and tertiary sites toward secondary general hospitals during and after the conflict.

By ownership, the NGO-managed facility experienced large war-period reductions (diagnoses: IRR, 0.21; 95% CI, 0.13-0.36; *P* < .001) with no evidence of postwar recovery (IRR, 0.20; 95% CI, 0.12-0.35; *P* < .001), whereas public facilities showed moderate war-period reductions (IRR, 0.74; 95% CI, 0.60-0.92; *P* = .006) and postwar values consistent with the prewar period (IRR, 1.05; 95% CI, 0.84-1.31; *P* = .65). The war × ownership interaction was significant (*P* < .001). Because the NGO stratum reflects a single facility, this finding describes one institution’s trajectory and should not be generalized to NGO-managed HIV programs broadly (eFigures 1-4 in Supplement 1).

Likelihood-ratio interaction tests for period × stratifier (sex, age, facility level, ownership) are reported in Table 3 and eTable 11 in Supplement 1. Significant interactions for age and ownership across all 3 outcomes (all *P* < .001) confirmed heterogeneous outcomes by these stratifiers.

**Table 3.** Period × Stratifier Interaction Tests by LR Comparison (Count Models)^a^

Outcome	LR χ^2 ^(*df*)	*P* value
Sex		
HIV diagnoses	4.86 (3)	.18
Linkage to care	3.70 (3)	.30
ART initiation	1.69 (3)	.64
Age group		
HIV diagnoses	92.08 (9)	<.001
Linkage to care	70.41 (9)	<.001
ART initiation	28.02 (9)	<.001
Facility level		
HIV diagnoses	4.55 (6)	.60
Linkage to care	5.35 (6)	.50
ART initiation	5.98 (6)	.43
Ownership		
HIV diagnoses	20.78 (3)	<.001
Linkage to care	20.05 (3)	<.001
ART initiation	31.98 (3)	<.001

Abbreviations: ART, antiretroviral therapy; LR, likelihood ratio.

^a^ Likelihood-ratio test comparing a main-effects negative binomial model (count ~ time + period dummies + stratum + seasonal harmonics) to one with period × stratum interactions. Significant interactions (*P* < .05) indicate that the period outcome varies across strata after adjusting for the main stratum. Strong interactions for age and ownership across all 3 outcomes (all *P* < .001); sex and facility-level interactions did not reach conventional significance, although stratum-level incidence rate ratios differed substantially in those analyses (Table 2; eFigures 1 and 3 in Supplement 1).

### Sensitivity and Diagnostic Analyses

Model diagnostics supported the validity of our analytical approach (eFigures 5-7 in Supplement 1). DHARMa-based dispersion tests indicated no detectable overdispersion in the negative binomial models, and DHARMa temporal autocorrelation tests showed no significant residual autocorrelation across the main models. Sensitivity analyses demonstrated robustness of main findings: removal of seasonal harmonic terms changed war-period IRRs by less than 2%; the slope-change specification (full model with time-after-war and time-after-postwar terms) yielded similar level shifts at war onset, with nonsignificant slope-change terms (eTable 5 in Supplement 1); restricting the prewar baseline to 2015 onward yielded slightly larger war-period reductions (eg, diagnoses: IRR, 0.52; 95% CI, 0.38-0.73), reflecting a higher recent baseline; an ARIMA model with intervention regressors yielded the same direction of pandemic, war, and postwar associations as the segmented regression.

Numerical results for the 4 sensitivity analyses are tabulated in eTables 7-10 in Supplement 1 (1 table per sensitivity), with corresponding overall cascade fits in eFigures 8-11 and DHARMa diagnostic composites in eFigure 12 in Supplement 1. Complementary ARIMA-with-intervention-regressors analyses are reported in eTable 6 (parameter estimates) and visualized in eFigures 6 (fitted vs observed) and 7 (residual diagnostics) in Supplement 1.

## Discussion

This interrupted time-series analysis examines associations between the Tigray conflict and HIV care cascade entry using 20 years of electronic medical record data from 7 health facilities in Mekelle City. During the 2-year war period, HIV diagnoses, linkage to care, and ART initiation all declined relative to expected prewar trajectories, with inequitable postwar values returning to levels consistent with prewar trajectories on average but cumulative deficits accrued during the war not recovered. The associations were heterogeneous: women experienced larger reductions than men; individuals aged 0 to 24 years had the largest declines, while those aged 25 to 34 years showed smaller, nonsignificant changes; the NGO-managed facility had large war-period and postwar reductions without recovery, whereas public facilities had moderate reductions during war and postwar values consistent with the prewar period.

Our findings align with and extend limited evidence on conflict associations with HIV services. In our systematic review of 16 quantitative studies across sub-Saharan Africa (2002-2022), we documented wide outcome variation: loss to follow-up ranged from 5.4% to 43.5%, virologic nonsuppression was pooled at 30.0% in active conflict settings, and mortality ranged between 4.2% and 13.0%.^3^ Our interrupted time-series design, which modeled changes at war onset and ceasefire, directly addresses these limitations. The magnitude of service reduction we observed (29%-32% overall war-period declines) appears more moderate than the near-total collapse documented at population level, where only 3.6% of all Tigray health facilities were fully functional by mid-2021.^13^ This discrepancy should be interpreted in light of our data source. Data were collected from health facilities in Mekelle City, where infrastructure remained relatively intact compared with other areas of Tigray; approximately 75% of Mekelle facilities maintained functionality.^13^

Multiple converging mechanisms may contribute to the observed service reductions. Structurally, approximately 86% of health facilities experienced damage, 28 were destroyed, and equipment in 232 facilities became unusable.^26^ Health workforce displacement and intensifying blockade restrictions on fuel, medicines, and laboratory reagents disrupted supply chains, testing, and ART distribution.^27^ Humanitarian crises displaced 4.8 to 5.2 million people, creating acute survival needs that overrode chronic disease management.^28^ Gendered conflict associations, including increased caregiving responsibilities, reduced mobility, gender-based violence exposure, and transport access barriers, plausibly contribute to larger service declines among women.^29^

The prewar period was characterized by a declining secular trend in incident HIV diagnoses, consistent with regional reports of declining HIV incidence in Ethiopia and improving treatment uptake.^30,31^ The 2015 WHO recommendation for universal treatment regardless of CD4+ T cell count, adopted by Ethiopia in 2016,^32,33^ accelerated ART initiation among individuals already in care, but the overall declining trend in newly diagnosed cases persisted because of declining HIV transmission and improved viral suppression among those already receiving ART.^34,35^ Our model accounted for this baseline trend through the linear time term, and the sensitivity analysis restricting the prewar baseline to the 2015-and-after era yielded comparable war-period estimates, indicating that our findings are not an artifact of the longer prewar window.

Facility-level differences require careful interpretation. The single NGO-managed facility in our sample showed large war-period and postwar reductions without recovery toward prewar levels. Because this stratum reflects one institution, the finding cannot be generalized to NGO-managed HIV programs broadly. NGO programs typically depend on external funding and supply chains that may be inaccessible during humanitarian blockades and employ staff who may evacuate during conflict; if these dynamics generalize, contingency planning for restricted-access scenarios would strengthen NGO program resilience.

These findings inform conflict-sensitive HIV programming: emergency preparedness may benefit from incorporating HIV service continuity planning with specific attention to vulnerable subgroups including women and individuals aged 0 to 24 years. Service decentralization to secondary-level facilities, which in our data showed war-period increases consistent with redistribution from other levels, combined with task-shifting and community health worker deployment, could be integrated into conflict contingency plans.

### Limitations

Several limitations warrant consideration. Our data derive from facilities maintaining operational capacity; completely nonfunctional facilities contributed no data, potentially underestimating the magnitude of conflict-period reductions. We used aggregate quarterly counts rather than patient-level data, precluding individual-level outcome analysis. Population-level denominators were unavailable, preventing coverage rate calculation. Our Mekelle-based sample likely represents best-case urban scenarios, with rural areas experiencing worse reductions in service delivery. Patients displaced from Mekelle to other regions, refugee settings, or facilities outside our network during the conflict cannot be enumerated in this dataset; some of the apparent decline in cascade entry may therefore reflect geographic redistribution of patients rather than service contraction alone. Our NGO-managed stratum represents a single facility, and findings should not be generalized to NGO-managed HIV programs broadly. Furthermore, we were unable to include viral suppression as a fourth cascade outcome because routine viral load testing in Tigray was suspended from approximately late 2020 through 2022 owing to destruction of laboratory infrastructure, reagent stockouts, and disruption of sample referral networks; testing resumed only intermittently in 2023, and the available viral load data therefore do not span enough quarters during and after the war for a valid interrupted time-series analysis.

## Conclusions

In this cohort study using interrupted time-series analysis to assess 7 health facilities in Mekelle, Ethiopia, the Tigray War period was associated with severe and inequitable reductions in HIV care cascade entry, with diagnoses, linkages to care, and ART initiations declining by approximately 29% to 32% relative to prewar trajectories. Reductions were larger among women, individuals aged 0 to 24 years, primary-level facilities, and the single NGO-managed facility, while secondary-level general hospitals showed war-period increases consistent with service redistribution. Postwar IRRs returned to levels consistent with prewar trajectories on average, but the cumulative deficits accrued during the war and postwar periods (approximately 265 diagnoses, 276 linkages, and 264 ART initiations) had not been recovered by Q1 2025. In the context of a doubled HIV prevalence reported by regional surveillance, current service output appears insufficient to match prewar epidemic control without substantial scale-up. These findings provide evidence to support conflict-sensitive HIV programming in Ethiopia and other settings affected by armed conflict.

## Notes

Supplement 1. **eMethods 1.** Extended Statistical Methods **eMethods 2.** Data Sources, Period Structure, and Operational Definitions **eTable 1.** Quarterly Observation Count by Analytical Period **eTable 2.** Period-Level Summary Statistics for Cascade Outcomes (Fully Stratified) **eTable 3.** Prewar Secular Trend Analysis (Q1 2005 to Q4 2019) **eTable 4.** Stratum Contribution to Total Cascade Volume by Period **eTable 5.** Main Analysis: Negative Binomial Segmented Regression IRRs With DHARMa Diagnostics **eTable 6.** Rate (Binomial GLM) Models: Linkage and ART Initiation Conditional Probabilities **eTable 7.** ARIMA Models With Intervention Regressors **eTable 8.** Sensitivity Analysis 1: Model Without Seasonal Harmonics **eTable 9.** Sensitivity Analysis 2: Full Slope-Change Model **eTable 10.** Sensitivity Analysis 3: Alternative War Onset (Q4 2020) **eTable 11.** Sensitivity Analysis 4: Prewar Restricted to 2015 Onward **eTable 12.** Period × Stratifier Interaction Tests (Likelihood Ratio Tests) **eTable 13.** Cumulative Cascade Deficits With Parametric Bootstrap 95% CIs **eAppendix 1.** Description of eFigures **eFigure 1.** Sex-Stratified Main Analysis: Cascade Fits by Female and Male **eFigure 2.** Age-Stratified Main Analysis: Cascade Fits by 4 Age Groups **eFigure 3.** Facility-Level Stratified Main Analysis: Primary, Secondary, Tertiary **eFigure 4.** Ownership-Stratified Main Analysis: Public and the Single NGO-Managed Facility **eFigure 5.** DHARMa Residual Diagnostics: Overall Negative Binomial Models **eFigure 6.** ARIMA Fitted vs Observed: With Intervention Regressors **eFigure 7.** ARIMA Residual Diagnostics: Residuals Over Time and ACF **eFigure 8.** Sensitivity Analysis 1 (No Seasonal Harmonics), Overall **eFigure 9.** Sensitivity Analysis 2 (Full Slope-Change Specification), Overall **eFigure 10.** Sensitivity Analysis 3 (Alternative War Onset Q4 2020), Overall **eFigure 11.** Sensitivity Analysis 4 (Prewar Baseline Restricted to 2015+), Overall **eFigure 12.** Sensitivity DHARMa Overview: 4 Sensitivities × 3 Outcomes **eMethods 3.** Detailed Methodology for the Count-Model Interrupted Time-Series Analysis **eAppendix 2.** Complete R Analysis Script

Supplement 2.
Data Sharing Statement

## References

[1] OECD. *States of Fragility 2022*. OECD; 2022. doi:10.1787/c7fedf5e-en

[2] Marley J, Desai H. Fragility and Agenda 2030: navigating shocks and pressures in fragile contexts. OECD Development Co-operation Paper 82. September 2020. Accessed June 16, 2026. https://www.oecd.org/content/dam/oecd/en/publications/reports/2020/09/fragility-and-agenda-2030_7bd9908c/65d5cb9c-en.pdf

[3] Kebede HK, Gesesew HA, Gebremedhin AT, Ward P. The impact of armed conflicts on HIV treatment outcomes in Sub-Saharan Africa: a systematic review and meta-analysis. Confl Health. 2024;18(1):40. doi:10.1186/s13031-024-00591-8 38760792 PMC11100029

[4] Deribew A, Biadgilign S, Deribe K, . The burden of HIV/AIDS in Ethiopia from 1990 to 2016: evidence from the global burden of diseases 2016 study. Ethiop J Health Sci. 2019;29(1):859-868. 30700953 10.4314/ejhs.v29i1.7 PMC6341438

[5] UNAIDS. Findings of the mid-term review of the global AIDS strategy. November 15, 2024. Accessed June 16, 2026. https://www.unaids.org/sites/default/files/media_asset/PCB55_Findings_MTR_Global_AIDS_Strategy.pdf

[6] World Health Organization. African region: country disease outlook. Accessed June 16, 2026. https://www.afro.who.int/publications/country-disease-outlook

[7] UNAIDS update: Impact of the pause of U.S. foreign assistance in Ethiopia. February 5, 2025. Accessed June 16, 2026. https://www.unaids.org/en/pepfar-impact-ethiopia

[8] Winning A, Cocks T. Combatants in Ethiopia’s Tigray war agree to stop fighting. Reuters. November 2, 2022. Accessed June 16, 2026. https://www.reuters.com/world/africa/african-union-parties-ethiopia-conflict-have-agreed-cease-hostilities-2022-11-02/

[9] African Union. Cessation of Hostilities Agreement between the Government of the Federal Democratic Republic of Ethiopia and the Tigray Peoples’ Liberation Front (TPLF). November 2, 2022. Accessed June 16, 2026. https://www.peaceau.org/en/article/cessation-of-hostilities-agreement-between-the-government-of-the-federal-democratic-republic-of-ethiopia-and-the-tigray-peoples-liberation-front-tplf

[10] Gesesew H, Berhane K, Siraj ES, . The impact of war on the health system of the Tigray region in Ethiopia: an assessment. BMJ Glob Health. 2021;6(11):e007328. doi:10.1136/bmjgh-2021-007328 34815244 PMC8611430

[11] Tsadik M, Gebretnsae H, Ayalew A, . Child health services and armed conflict in Tigray, North Ethiopia: a community-based study. Confl Health. 2023;17(1):47. doi:10.1186/s13031-023-00545-6 37798759 PMC10557173

[12] Gesesew H, Kebede H, Berhe K, Fauk N, Ward P. Perilous medicine in Tigray: a systematic review. Confl Health. 2023;17(1):26. doi:10.1186/s13031-023-00524-x 37254199 PMC10228460

[13] Gebregziabher M, Amdeselassie F, Esayas R, . Geographical distribution of the health crisis of war in the Tigray region of Ethiopia. BMJ Glob Health. 2022;7(4):e008475. doi:10.1136/bmjgh-2022-008475 35487674 PMC9058686

[14] Ajemu KF, Hadgu T, Gebreegziabher G, . Conflict and fragmented public health emergency management system in Tigray region of Northern Ethiopia: a double burden to accommodate resilient and advanced public health emergency management—a commentary review for policy-makers and a call to action. Health Res Policy Syst. 2024;22(1):121. doi:10.1186/s12961-024-01176-w 39227920 PMC11370228

[15] Government of Ethiopia. Ethiopia resilient recovery and reconstruction planning framework (2023-28): Volume B. March 2023. Accessed June 16, 2026. https://documents1.worldbank.org/curated/en/099062223114014041/pdf/P1786960f38c8d051096ac0d915ec0d710a.pdf

[16] UNICEF. Ethiopia humanitarian situation report, October 2021. Accessed June 22, 2026. https://www.unicef.org/documents/ethiopia-humanitarian-situation-report-october-2021

[17] Weledegebriel MG, Abebe HT, Gidey K, . The impact of war on HIV/AIDS service provision: In rural health facilities of Tigray, northern Ethiopia, a cross-sectional study. PLoS One. 2023;18(5):e0278976. doi:10.1371/journal.pone.0278976 37130130 PMC10153695

[18] Bernal JL, Cummins S, Gasparrini A. Interrupted time series regression for the evaluation of public health interventions: a tutorial. Int J Epidemiol. 2017;46(1):348-355. doi:10.1093/ije/dyw098 27283160 PMC5407170

[19] Cities Alliance. Mekelle: a regional commitment to urban expansion planning. December 8, 2023. Accessed June 16, 2026. https://www.citiesalliance.org/newsroom/news/results/mekelle-regional-commitment-urban-expansion-planning

[20] Tilahun B, Fritz F. Comprehensive evaluation of electronic medical record system use and user satisfaction at five low-resource setting hospitals in Ethiopia. JMIR Med Inform. 2015;3(2):e22. doi:10.2196/medinform.4106 26007237 PMC4460264

[21] Jiang H, Feng X, Lange S, Tran A, Manthey J, Rehm J. Estimating effects of health policy interventions using interrupted time-series analyses: a simulation study. BMC Med Res Methodol. 2022;22(1):235. doi:10.1186/s12874-022-01716-4 36045338 PMC9429656

[22] Gardner W, Mulvey EP, Shaw EC. Regression analyses of counts and rates: Poisson, overdispersed Poisson, and negative binomial models. Psychol Bull. 1995;118(3):392-404. doi:10.1037/0033-2909.118.3.392 7501743

[23] Advanced Research Computing UCLA. Poisson regression—Stata annotated output. Accessed June 16, 2026. https://stats.oarc.ucla.edu/stata/output/poisson-regression/

[24] Musa KI, Mansor WNAW, Hanis TK. Poisson regression. In: Musa KI, Mansor WNAW, Hanis TK, eds. Data Analysis in Medicine and Health Using R. Chapman and Hall; 2023.

[25] Quantargo. DHARMa: testTemporalAutocorrelation. Accessed June 16, 2026. https://www.quantargo.com/help/r/latest/packages/DHARMa/0.4.1/testTemporalAutocorrelation

[26] Gufue ZH, Haftu HK, Alemayehu Y, Tsegay EW, Mengesha MB, Dessalegn B. Damage to the public health system caused by war-related looting or vandalism in the Tigray region of Northern Ethiopia. Front Public Health. 2024;12:1271028. doi:10.3389/fpubh.2024.1271028 38645448 PMC11026641

[27] World Health Organization. Crisis in Northern Ethiopia. June 18, 2022. Accessed June 16, 2026. https://www.who.int/emergencies/situations/crisis-in-tigray-ethiopia

[28] Inter-Agency Standing Committee. Inter-agency humanitarian evaluation of the response to the crisis in Northern Ethiopia. June 3, 2024. Accessed June 16, 2026. https://interagencystandingcommittee.org/inter-agency-humaniatrian-evaluations-steering-group/inter-agency-humanitarian-evaluation-response-humanitarian-crisis-northern-ethiopia

[29] Papas L, Hollingdrake O, Currie J. Social determinant factors and access to health care for women experiencing domestic and family violence: qualitative synthesis. J Adv Nurs. 2023;79(5):1633-1649. doi:10.1111/jan.15565 36695338

[30] Kitaw TA, Azmeraw M, Abate BB, . The burden of HIV/AIDS in Ethiopia: unveiling 30 years of trends in incidence, mortality, and disability—insights from the Global Burden of Disease study (1990-2021). PLoS One. 2025;20(4):e0321024. doi:10.1371/journal.pone.0321024 40233050 PMC11999106

[31] Girum T, Wasie A, Worku A. Trend of HIV/AIDS for the last 26 years and predicting achievement of the 90-90-90 HIV prevention targets by 2020 in Ethiopia: a time series analysis. BMC Infect Dis. 2018;18(1):320. doi:10.1186/s12879-018-3214-6 29996776 PMC6042262

[32] Ahmed I, Demissie M, Worku A, Gugsa S, Berhane Y. Effectiveness of same-day antiretroviral therapy initiation in retention outcomes among people living with human immunodeficiency virus in Ethiopia: empirical evidence. BMC Public Health. 2020;20(1):1802. doi:10.1186/s12889-020-09887-9 33243185 PMC7690160

[33] Bantie B, Abate MW, Nigat AB, . Attrition rate and its predictors among adults receiving anti-retroviral therapy following the implementation of the “Universal Test and Treat strategy” at public health institutions in Northern Ethiopia: a retrospective follow-up study. Heliyon. 2022;8(11):e11527. doi:10.1016/j.heliyon.2022.e11527 36411907 PMC9674913

[34] Montaner JSG, Lima VD, Harrigan PR, . Expansion of HAART coverage is associated with sustained decreases in HIV/AIDS morbidity, mortality and HIV transmission: the “HIV Treatment as Prevention” experience in a Canadian setting. PLoS One. 2014;9(2):e87872. doi:10.1371/journal.pone.0087872 24533061 PMC3922718

[35] Ford N, Vitoria M, Doherty M. Providing antiretroviral therapy to all who are HIV positive: the clinical, public health and programmatic benefits of Treat All. J Int AIDS Soc. 2018;21(2):e25078. doi:10.1002/jia2.25078 29436776 PMC5810349

