# Supplementary material for: Tigray War and HIV Care Cascade Entry in Mekelle, Ethiopia
Source: JAMA Netw Open. 2026 Jul 24;9(7):e2625130. doi: 10.1001/jamanetworkopen.2026.25130 (PMC13401200; doi:10.1001/jamanetworkopen.2026.25130)
Supplement: Supplement 1. — eMethods 1. Extended Statistical Methods eMethods 2. Data Sources, Period Structure, and Operational Definitions eTable 1. Quarterly Observation Count by Analytical Period eTable 2. Period-Level Summary Statistics for Cascade Outcomes (Fully Stratified) eTable 3. Prewar Secular Trend Analysis (Q1 2005 to Q4 2019) eTable 4. Stratum Contribution to Total Cascade Volume by Period eTable 5. Main Analysis: Negative Binomial Segmented Regression IRRs With DHARMa Diagnostics eTable 6. Rate (Binomial GLM) Models: Linkage and ART Initiation Conditional Probabilities eTable 7. ARIMA Models With Intervention Regressors eTable 8. Sensitivity Analysis 1: Model Without Seasonal Harmonics eTable 9. Sensitivity Analysis 2: Full Slope-Change Model eTable 10. Sensitivity Analysis 3: Alternative War Onset (Q4 2020) eTable 11. Sensitivity Analysis 4: Prewar Restricted to 2015 Onward eTable 12. Period × Stratifier Interaction Tests (Likelihood Ratio Tests) eTable 13. Cumulative Cascade Deficits With Parametric Bootstrap 95% CIs eAppendix 1. Description of eFigures eFigure 1. Sex-Stratified Main Analysis: Cascade Fits by Female and Male eFigure 2. Age-Stratified Main Analysis: Cascade Fits by 4 Age Groups eFigure 3. Facility-Level Stratified Main Analysis: Primary, Secondary, Tertiary eFigure 4. Ownership-Stratified Main Analysis: Public and the Single NGO-Managed Facility eFigure 5. DHARMa Residual Diagnostics: Overall Negative Binomial Models eFigure 6. ARIMA Fitted vs Observed: With Intervention Regressors eFigure 7. ARIMA Residual Diagnostics: Residuals Over Time and ACF eFigure 8. Sensitivity Analysis 1 (No Seasonal Harmonics), Overall eFigure 9. Sensitivity Analysis 2 (Full Slope-Change Specification), Overall eFigure 10. Sensitivity Analysis 3 (Alternative War Onset Q4 2020), Overall eFigure 11. Sensitivity Analysis 4 (Prewar Baseline Restricted to 2015+), Overall eFigure 12. Sensitivity DHARMa Overview: 4 Sensitivities × 3 Outcomes eMethods 3. Detailed Methodology for the Count-M [file jamanetwopen-e2625130-s001.pdf]

Supplemental Online Content

Kebede HK, Gesesew HA, Mwanri L, Ward P. Tigray War and HIV care cascade entry in Mekelle, Ethiopia. *JAMA Netw Open.* 2026;9(7):e2625130. doi:10.1001/jamanetworkopen.2026.25130

eMethods 1. Extended Statistical Methods
eMethods 2. Data Sources, Period Structure, and Operational Definitions
eTable 1. Quarterly Observation Count by Analytical Period
eTable 2. Period-Level Summary Statistics for Cascade Outcomes (Fully Stratified)
eTable 3. Prewar Secular Trend Analysis (Q1 2005 to Q4 2019)
eTable 4. Stratum Contribution to Total Cascade Volume by Period
eTable 5. Main Analysis: Negative Binomial Segmented Regression IRRs With DHARMa Diagnostics
eTable 6. Rate (Binomial GLM) Models: Linkage and ART Initiation Conditional Probabilities
eTable 7. ARIMA Models With Intervention Regressors
eTable 8. Sensitivity Analysis 1: Model Without Seasonal Harmonics
eTable 9. Sensitivity Analysis 2: Full Slope-Change Model
eTable 10. Sensitivity Analysis 3: Alternative War Onset (Q4 2020)
eTable 11. Sensitivity Analysis 4: Prewar Restricted to 2015 Onward
eTable 12. Period × Stratifier Interaction Tests (Likelihood Ratio Tests)
eTable 13. Cumulative Cascade Deficits With Parametric Bootstrap 95% CIs
eAppendix 1. Description of eFigures
eFigure 1. Sex-Stratified Main Analysis: Cascade Fits by Female and Male
eFigure 2. Age-Stratified Main Analysis: Cascade Fits by 4 Age Groups
eFigure 3. Facility-Level Stratified Main Analysis: Primary, Secondary, Tertiary
eFigure 4. Ownership-Stratified Main Analysis: Public and the Single NGO-Managed Facility
eFigure 5. DHARMa Residual Diagnostics: Overall Negative Binomial Models
eFigure 6. ARIMA Fitted vs Observed: With Intervention Regressors
eFigure 7. ARIMA Residual Diagnostics: Residuals Over Time and ACF
eFigure 8. Sensitivity Analysis 1 (No Seasonal Harmonics), Overall
eFigure 9. Sensitivity Analysis 2 (Full Slope-Change Specification), Overall
eFigure 10. Sensitivity Analysis 3 (Alternative War Onset Q4 2020), Overall
eFigure 11. Sensitivity Analysis 4 (Prewar Baseline Restricted to 2015+), Overall
eFigure 12. Sensitivity DHARMa Overview: 4 Sensitivities × 3 Outcomes
eMethods 3. Detailed Methodology for the Count-Model Interrupted Time-Series Analysis

© 2026 Kebede HK et al. *JAMA Network Open*.

eAppendix 2. Complete R Analysis Script

This supplemental material has been provided by the authors to give readers additional information about their work.

© 2026 Kebede HK et al. *JAMA Network Open*.

eMethods 1. Extended Statistical Methods

1.1 Primary segmented regression specification

The primary analysis used a four-period interrupted time-series segmented regression with seasonal harmonic terms. For each cascade outcome Y_t at quarter t (t = 1, ..., 81), we fitted the negative binomial regression:

$$log(E[Y\_t]) = \beta_0 + \beta_1 \cdot t + \beta_2 \cdot pandemic\_t + \beta_3 \cdot war\_t + \beta_4 \cdot post\_t + \beta_5 \cdot sin(2\pi t/4) + \beta_6 \cdot cos(2\pi t/4)$$

where pandemic_t, war_t, and post_t are 0/1 indicators for the corresponding periods, and the sine–cosine pair captures annual seasonality at a quarterly frequency. The pre-war period (Q1 2005–Q4 2019; 60 quarters) is the reference, so all effect estimates are reported relative to pre-war. Coefficients β_2, β_3, and β_4 were exponentiated to obtain incidence rate ratios (IRRs) with 95% Wald confidence intervals.

1.2 Model fitting and convergence handling

Negative binomial models were fitted using glmmTMB::glmmTMB() with family = nbinom2 and the nlminb optimizer (eval.max = 2000, iter.max = 1000). Convergence was verified by checking that the Hessian was positive-definite (sdr$pdHess == TRUE) and that all standard errors were finite and below 1000.

When negative binomial models did not converge, we refitted using quasi-Poisson regression (stats::glm with family = quasipoisson(link = "log")). Of the 36 count models reported in this manuscript, 35 used negative binomial regression and 1 (HIV Diagnoses, age 25–34 years) used quasi-Poisson regression. Quasi-Poisson, rather than ordinary Poisson, was used as the fallback because it estimates the dispersion parameter from the data and does not assume mean = variance.

1.3 Rate (binomial) models

To complement the count-model analysis, we fitted binomial generalised linear models on conditional cascade probabilities (linkage given diagnosis; ART initiation given linkage). The structural form was identical to the count models, log-link in counts, logit-link in rates, same time index, period indicators, and seasonal harmonics, but rate models could not be fitted for every stratum because of small denominators in early pre-war quarters (e.g., NGO stratum had quarterly diagnosis counts of 0). The full set of 17 rate models that successfully converged is reported in eTable 6 with coefficient details in eTable 6a.

1.4 Counterfactual projection and cumulative deficits

Counterfactual projections represent the trajectory each outcome would have followed in the absence of the pandemic and war shocks, holding linear time and seasonal harmonic terms at their fitted values. We set pandemic_dummy = 0, war_dummy = 0, and post_dummy = 0 for all post-2019 observations, then re-predicted on the response scale.

Cumulative cascade deficits were computed as the sum across war and post-war quarters of (counterfactual prediction − observed value). Confidence intervals were obtained by parametric bootstrap (5000 replications): for each replicate we drew Y_t* from a negative binomial distribution with mean equal to the fitted value μ_t and dispersion θ from the original model, refitted glmmTMB on the bootstrap sample, computed the cumulative deficit for that replicate, and took the 2.5th and 97.5th percentiles of the resulting empirical distribution. Results are reported in eTable 13.

1.5 Residual diagnostics with DHARMa

Model adequacy was assessed using the R DHARMa package (Hartig 2022, version 0.4.6 or later). DHARMa generates simulation-based scaled residuals that are uniformly distributed under correct model specification regardless of the underlying GLM family, providing valid diagnostic tests for negative binomial and quasi-Poisson models.

We applied two formal tests: testDispersion() for over-/underdispersion and testTemporalAutocorrelation() applied to ordered quarterly residuals. P values from both DHARMa tests are reported in eTable 5 alongside the IRR estimates.

1.6 ARIMA with intervention regressors

As a complementary time-series approach, we fitted ARIMA models with the same intervention regressors using forecast::auto.arima with xreg = matrix(pandemic, war, postwar). stepwise = FALSE and approximation = FALSE were specified to ensure full search over candidate models with max.p = 4 and max.q = 4. Results are reported in eTable 7.

1.7 Sensitivity analyses

Four pre-specified sensitivity analyses evaluated robustness. Each was run for all 53 models (36 count + 17 rate). Full stratified results are reported in eTables 8–11.

#	Sensitivity analysis	Description	Reported in
1	No seasonality	Removed sin/cos harmonic terms.	eTable 8
2	Full slope-change model	Added time-after-war and time-after-postwar slope-change terms.	eTable 9
3	Alternative war onset (Q4 2020)	Reassigned Q4 2020 from pandemic to war.	eTable 10

#	Sensitivity analysis	Description	Reported in
4	Pre-war restricted to 2015 onward	Restricted the pre-war baseline to Q1 2015–Q4 2019.	eTable 11

1.8 Period × stratifier interaction tests

To formally test effect modification, we constructed long-format datasets by stacking the stratum-level counts within each stratifier and fitted two MASS::glm.nb models per outcome–stratifier combination (main effects vs main + period × stratum interactions), comparing them by likelihood ratio test. Results are reported in eTable 12.

© 2026 Kebede HK et al. *JAMA Network Open*.

eMethods 2. Data Sources, Period Structure, and Operational Definitions

2.1 Data source

Data were extracted from SmartCare, an electronic medical record platform supported by PEPFAR/ICAP (Columbia University) and deployed across health facilities in Mekelle City. SmartCare maintains comprehensive longitudinal records of HIV care at the individual patient level, with quarterly aggregation supported by built-in reporting modules. De-identified clinical data were extracted on May 2025 from 7 health facilities with continuous quarterly records spanning Q1 2005 – Q1 2025.

2.2 Period structure (4-period model)

Period	n quarters	Start	End
Pre-war	60	2005 Q1	2019 Q4
Pandemic	4	2020 Q1	2020 Q4
War	8	2021 Q1	2022 Q4
Post-war	9	2023 Q1	2025 Q1

Total = 81 quarters (Q1 2005 – Q1 2025); Q2 2025 was excluded because it contains only 2 of 3 calendar months. The pre-war period serves as the reference group in all regression models.

2.3 Variable definitions

We analyzed 3 cascade outcomes, each measured as quarterly aggregate counts: HIV diagnoses (number of individuals newly diagnosed each quarter); linkage to care (number of newly diagnosed HIV-positive individuals enrolled in care during the quarter); ART initiation (number of newly diagnosed HIV-positive individuals starting ART during the quarter; incident treatment starts, not ART coverage). Rate (conditional probability) versions of linkage and ART initiation were also analysed using binomial GLMs (eTable 6).

2.4 Transfer-in / transfer-out definitions

Transfer-outs and transfer-ins were defined operationally from the SmartCare dataset. A transfer-out was recorded when a patient's status was documented as transferred out, and a transfer-in was recorded when a patient's status was documented as transferred in. These classifications were accepted as recorded without independent verification.

2.5 Stratification scheme

Pre-specified strata: sex (Female, Male); age (0–24, 25–34, 35–44, ≥45 years); facility level (Primary, Secondary, Tertiary); facility ownership (Public, NGO; the NGO stratum reflects a single facility and is not generalisable to NGO programs broadly).

2.6 Software and reproducibility

All analyses were conducted in R version 4.6.0 using CRAN-published packages only: dplyr, tidyr, readr, stringr, purrr, tibble (data wrangling); glmmTMB, MASS, DHARMa, forecast (modelling and diagnostics); ggplot2, scales, patchwork (figures); openxlsx (output); zoo (interpolation of <5% missing). The complete R code is provided as the eAppendix at the end of this combined supplement document. Random seed: 2025.

© 2026 Kebede HK et al. *JAMA Network Open*.

eTable 1. Quarterly Observation Count by Analytical Period

Period	n quarters	Start quarter	End quarter
Pre-war	60	2005 Q1	2019 Q4
Pandemic	4	2020 Q1	2020 Q4
War	8	2021 Q1	2022 Q4
Post-war	9	2023 Q1	2025 Q1

Total = 81 quarters. The pre-war period (60 quarters) serves as the reference group.

© 2026 Kebede HK et al. *JAMA Network Open*.

eTable 2. Period-Level Summary Statistics for Cascade Outcomes (Fully Stratified)

All combinations of outcome × stratifier × stratum × period (3 outcomes × 12 stratum-levels × 4 periods = 144 rows). Mean (SD), median [IQR], min–max, and total represent quarterly counts within each period. CV = SD/mean × 100%. Δ mean vs pre-war is the percentage change in mean quarterly count from the pre-war reference period within that stratum.

Outcome	Stratifier	Stratum	Period	n Q	Mean (SD)/Q	Median [IQR]/Q	Min–max/Q	Period total	CV %	Δ vs pre-war (%)
HIV Diagnoses	Overall	All	Pre-war	60	200.4 (67.3)	179.5 [145–264]	89–348	12,027	33.6	ref
HIV Diagnoses	Overall	All	Pandemic	4	75.8 (27.8)	67.0 [60–82]	53–116	303	36.7	-62.2
HIV Diagnoses	Overall	All	War	8	74.8 (15.1)	77.0 [65–88]	48–90	598	20.2	-62.7
HIV Diagnoses	Overall	All	Post-war	9	90.1 (24.7)	97.0 [87–109]	36–110	811	27.4	-55.0
HIV Diagnoses	Sex	Female	Pre-war	60	116.3 (36.7)	108.0 [88–148]	53–199	6,977	31.5	ref
HIV Diagnoses	Sex	Female	Pandemic	4	43.2 (17.9)	38.0 [34–47]	28–69	173	41.3	-62.8
HIV Diagnoses	Sex	Female	War	8	38.5 (10.3)	39.5 [30–46]	23–52	308	26.6	-66.9
HIV Diagnoses	Sex	Female	Post-war	9	50.6 (14.7)	50.0 [40–64]	25–68	455	29.1	-56.5
HIV Diagnoses	Sex	Male	Pre-war	60	84.2 (33.7)	75.0 [58–108]	36–179	5,050	40.0	ref
HIV Diagnoses	Sex	Male	Pandemic	4	32.5 (10.0)	29.0 [26–35]	25–47	130	30.7	-61.4
HIV Diagnoses	Sex	Male	War	8	36.2 (5.8)	36.5 [34–39]	25–45	290	16.1	-56.9
HIV Diagnoses	Sex	Male	Post-war	9	39.6 (12.1)	42.0 [41–47]	11–51	356	30.6	-53.0
HIV Diagnoses	Age	0-24 years	Pre-war	60	32.1 (11.4)	33.5 [23–40]	8–53	1,925	35.4	ref
HIV Diagnoses	Age	0-24 years	Pandemic	4	12.0 (7.2)	10.5 [9–14]	5–22	48	59.7	-62.6
HIV Diagnoses	Age	0-24 years	War	8	11.1 (3.5)	12.0 [8–13]	6–16	89	31.3	-65.3
HIV Diagnoses	Age	0-24 years	Post-war	9	16.4 (5.6)	18.0 [14–21]	6–21	148	34.0	-48.7
HIV Diagnoses	Age	25-34 years	Pre-war	60	34.6 (4.3)	35.2 [32–37]	24–48	2,073	12.4	ref
HIV Diagnoses	Age	25-34 years	Pandemic	4	27.8 (6.9)	29.2 [26–31]	18–34	111	24.7	-19.5
HIV Diagnoses	Age	25-34 years	War	8	32.9 (5.7)	33.8 [28–36]	25–42	262	17.3	-4.9
HIV Diagnoses	Age	25-34 years	Post-war	9	33.6 (4.2)	33.9 [31–37]	26–39	302	12.6	-2.9
HIV Diagnoses	Age	35-44 years	Pre-war	60	64.4 (23.4)	56.0 [46–85]	28–121	3,862	36.3	ref
HIV Diagnoses	Age	35-44 years	Pandemic	4	30.2 (8.7)	30.5 [25–36]	20–40	121	28.6	-53.0
HIV Diagnoses	Age	35-44 years	War	8	24.8 (5.7)	24.5 [22–27]	17–36	198	23.0	-61.5
HIV Diagnoses	Age	35-44 years	Post-war	9	25.9 (6.8)	28.0 [24–29]	10–33	233	26.2	-59.8
HIV Diagnoses	Age	45+ years	Pre-war	60	34.4 (13.5)	31.0 [23–45]	15–63	2,061	39.3	ref
HIV Diagnoses	Age	45+ years	Pandemic	4	11.2 (3.5)	13.0 [11–13]	6–13	45	31.1	-67.2
HIV Diagnoses	Age	45+ years	War	8	14.1 (5.0)	16.5 [11–17]	5–19	113	35.2	-58.9
HIV Diagnoses	Age	45+ years	Post-war	9	17.3 (5.2)	19.0 [14–21]	7–24	156	30.1	-49.5

© 2026 Kebede HK et al. *JAMA Network Open*.

Outcome	Stratifier	Stratum	Period	n Q	Mean (SD)/Q	Median [IQR]/Q	Min–max/Q	Period total	CV %	Δ vs pre-war (%)
HIV Diagnoses	Facility level	Primary	Pre-war	60	51.7 (23.7)	53.0 [40–65]	1–105	3,104	45.7	ref
HIV Diagnoses	Facility level	Primary	Pandemic	4	30.8 (13.9)	30.0 [21–40]	16–47	123	45.2	-40.6
HIV Diagnoses	Facility level	Primary	War	8	19.5 (8.9)	17.5 [14–24]	9–36	156	45.5	-62.3
HIV Diagnoses	Facility level	Primary	Post-war	9	28.2 (11.6)	33.0 [24–34]	7–43	254	41.0	-45.4
HIV Diagnoses	Facility level	Secondary	Pre-war	60	117.3 (82.5)	78.5 [57–169]	32–333	7,040	70.3	ref
HIV Diagnoses	Facility level	Secondary	Pandemic	4	29.8 (12.9)	31.0 [22–38]	14–43	119	43.3	-74.6
HIV Diagnoses	Facility level	Secondary	War	8	40.6 (4.7)	40.0 [37–44]	36–48	325	11.5	-65.4
HIV Diagnoses	Facility level	Secondary	Post-war	9	45.9 (11.8)	52.0 [40–54]	23–59	413	25.7	-60.9
HIV Diagnoses	Facility level	Tertiary	Pre-war	60	31.4 (18.0)	34.5 [13–42]	2–82	1,883	57.2	ref
HIV Diagnoses	Facility level	Tertiary	Pandemic	4	15.2 (7.2)	12.0 [12–16]	11–26	61	47.1	-51.4
HIV Diagnoses	Facility level	Tertiary	War	8	14.6 (8.9)	15.0 [8–21]	3–27	117	60.7	-53.4
HIV Diagnoses	Facility level	Tertiary	Post-war	9	16.0 (5.5)	17.0 [13–19]	6–23	144	34.4	-49.0
HIV Diagnoses	Ownership	NGO	Pre-war	60	12.3 (8.2)	13.0 [5–18]	0–31	739	66.8	ref
HIV Diagnoses	Ownership	NGO	Pandemic	4	12.2 (6.7)	9.5 [8–14]	8–22	49	54.3	-0.5
HIV Diagnoses	Ownership	NGO	War	8	9.2 (1.9)	9.5 [8–10]	6–12	74	20.6	-24.9
HIV Diagnoses	Ownership	NGO	Post-war	9	11.9 (3.3)	12.0 [10–14]	6–17	107	27.7	-3.5
HIV Diagnoses	Ownership	Public	Pre-war	60	188.1 (72.4)	162.0 [126–260]	71–348	11,288	38.5	ref
HIV Diagnoses	Ownership	Public	Pandemic	4	63.5 (21.6)	57.5 [50–71]	45–94	254	34.1	-66.2
HIV Diagnoses	Ownership	Public	War	8	65.5 (14.2)	68.0 [55–77]	42–81	524	21.6	-65.2
HIV Diagnoses	Ownership	Public	Post-war	9	78.2 (23.7)	87.0 [70–96]	30–100	704	30.2	-58.4
Linkage to Care	Overall	All	Pre-war	60	176.4 (59.2)	162.0 [129–228]	50–308	10,584	33.5	ref
Linkage to Care	Overall	All	Pandemic	4	70.0 (29.7)	61.0 [55–76]	45–113	280	42.5	-60.3
Linkage to Care	Overall	All	War	8	68.4 (17.1)	69.0 [58–83]	38–89	547	25.0	-61.2
Linkage to Care	Overall	All	Post-war	9	87.3 (24.7)	92.0 [87–106]	33–107	786	28.3	-50.5
Linkage to Care	Sex	Female	Pre-war	60	102.3 (33.3)	97.5 [80–127]	19–174	6,138	32.5	ref
Linkage to Care	Sex	Female	Pandemic	4	40.2 (18.9)	35.5 [30–45]	23–67	161	46.9	-60.7
Linkage to Care	Sex	Female	War	8	35.2 (10.6)	35.5 [28–45]	18–47	282	29.9	-65.5
Linkage to Care	Sex	Female	Post-war	9	48.2 (14.8)	49.0 [40–61]	22–65	434	30.6	-52.9
Linkage to Care	Sex	Male	Pre-war	60	74.1 (29.3)	66.0 [50–94]	31–151	4,446	39.5	ref
Linkage to Care	Sex	Male	Pandemic	4	29.8 (11.0)	25.5 [24–31]	22–46	119	36.9	-59.9
Linkage to Care	Sex	Male	War	8	33.1 (7.0)	34.0 [30–36]	20–44	265	21.2	-55.3
Linkage to Care	Sex	Male	Post-war	9	39.1 (11.8)	42.0 [40–46]	11–50	352	30.3	-47.2
Linkage to Care	Age	0-24 years	Pre-war	60	28.7 (9.4)	28.0 [22–36]	6–47	1,720	32.7	ref
Linkage to Care	Age	0-24 years	Pandemic	4	11.8 (7.2)	10.0 [9–13]	5–22	47	61.5	-59.0

© 2026 Kebede HK et al. *JAMA Network Open*.

Outcome	Stratifier	Stratum	Period	n Q	Mean (SD)/Q	Median [IQR]/Q	Min–max/Q	Period total	CV %	Δ vs pre-war (%)
Linkage to Care	Age	0-24 years	War	8	10.6 (3.4)	12.0 [7–13]	6–15	85	32.2	-62.9
Linkage to Care	Age	0-24 years	Post-war	9	15.6 (5.5)	17.0 [14–20]	6–21	140	35.1	-45.7
Linkage to Care	Age	25-34 years	Pre-war	60	34.5 (4.2)	34.9 [32–37]	22–45	2,068	12.1	ref
Linkage to Care	Age	25-34 years	Pandemic	4	26.8 (6.9)	28.2 [25–30]	17–34	107	25.8	-22.2
Linkage to Care	Age	25-34 years	War	8	32.4 (5.6)	32.4 [28–37]	25–40	259	17.4	-5.9
Linkage to Care	Age	25-34 years	Post-war	9	33.3 (4.1)	33.3 [30–36]	27–40	299	12.4	-3.5
Linkage to Care	Age	35-44 years	Pre-war	60	57.0 (20.9)	50.5 [40–74]	19–113	3,417	36.6	ref
Linkage to Care	Age	35-44 years	Pandemic	4	29.5 (9.9)	31.0 [27–33]	16–40	118	33.7	-48.2
Linkage to Care	Age	35-44 years	War	8	22.2 (5.8)	23.0 [18–24]	14–33	178	25.8	-60.9
Linkage to Care	Age	35-44 years	Post-war	9	25.1 (6.9)	27.0 [24–28]	9–32	226	27.3	-55.9
Linkage to Care	Age	45+ years	Pre-war	60	30.2 (12.5)	26.0 [20–40]	12–58	1,811	41.3	ref
Linkage to Care	Age	45+ years	Pandemic	4	10.5 (3.1)	11.5 [10–12]	6–13	42	29.6	-65.2
Linkage to Care	Age	45+ years	War	8	13.1 (4.9)	15.5 [10–16]	5–18	105	37.2	-56.5
Linkage to Care	Age	45+ years	Post-war	9	17.1 (5.2)	19.0 [14–20]	7–24	154	30.1	-43.3
Linkage to Care	Facility level	Primary	Pre-war	60	46.3 (22.3)	48.5 [38–59]	0–93	2,779	48.1	ref
Linkage to Care	Facility level	Primary	Pandemic	4	29.8 (13.4)	28.5 [20–38]	16–46	119	45.1	-35.8
Linkage to Care	Facility level	Primary	War	8	18.1 (8.9)	16.5 [13–24]	6–33	145	49.1	-60.9
Linkage to Care	Facility level	Primary	Post-war	9	27.8 (11.7)	32.0 [23–33]	6–43	250	42.3	-40.0
Linkage to Care	Facility level	Secondary	Pre-war	60	104.2 (71.1)	74.0 [50–152]	28–295	6,249	68.2	ref
Linkage to Care	Facility level	Secondary	Pandemic	4	27.2 (12.4)	27.0 [18–36]	14–41	109	45.6	-73.8
Linkage to Care	Facility level	Secondary	War	8	37.2 (5.8)	37.0 [33–41]	30–47	298	15.7	-64.2
Linkage to Care	Facility level	Secondary	Post-war	9	44.1 (11.4)	50.0 [37–52]	22–57	397	25.8	-57.6
Linkage to Care	Facility level	Tertiary	Pre-war	60	25.9 (15.7)	27.5 [10–37]	1–66	1,556	60.4	ref
Linkage to Care	Facility level	Tertiary	Pandemic	4	13.0 (8.7)	9.0 [9–13]	8–26	52	66.8	-49.9
Linkage to Care	Facility level	Tertiary	War	8	13.0 (8.2)	14.5 [6–20]	2–23	104	62.8	-49.9
Linkage to Care	Facility level	Tertiary	Post-war	9	15.4 (5.6)	17.0 [13–18]	5–23	139	36.2	-40.4
Linkage to Care	Ownership	NGO	Pre-war	60	11.2 (8.0)	12.5 [4–17]	0–29	672	71.6	ref
Linkage to Care	Ownership	NGO	Pandemic	4	12.0 (6.2)	9.5 [8–14]	8–21	48	51.4	+7.1
Linkage to Care	Ownership	NGO	War	8	9.0 (2.0)	9.5 [8–10]	5–11	72	22.2	-19.6
Linkage to Care	Ownership	NGO	Post-war	9	11.9 (3.3)	12.0 [10–14]	6–17	107	27.7	+6.2
Linkage to Care	Ownership	Public	Pre-war	60	165.2 (63.7)	150.0 [112–226]	50–308	9,912	38.5	ref
Linkage to Care	Ownership	Public	Pandemic	4	58.0 (24.0)	51.5 [44–65]	37–92	232	41.3	-64.9
Linkage to Care	Ownership	Public	War	8	59.4 (16.0)	60.0 [49–73]	33–79	475	26.9	-64.1
Linkage to Care	Ownership	Public	Post-war	9	75.4 (23.6)	82.0 [70–93]	27–97	679	31.3	-54.3

© 2026 Kebede HK et al. *JAMA Network Open*.

Outcome	Stratifier	Stratum	Period	n Q	Mean (SD)/Q	Median [IQR]/Q	Min–max/Q	Period total	CV %	Δ vs pre-war (%)
ART Initiation	Overall	All	Pre-war	60	133.9 (42.1)	127.5 [104–158]	41–246	8,037	31.4	ref
ART Initiation	Overall	All	Pandemic	4	69.2 (30.2)	60.0 [54–76]	44–113	277	43.6	-48.3
ART Initiation	Overall	All	War	8	67.8 (17.4)	68.5 [58–82]	37–89	542	25.7	-49.4
ART Initiation	Overall	All	Post-war	9	86.4 (24.3)	91.0 [87–104]	33–106	778	28.1	-35.5
ART Initiation	Sex	Female	Pre-war	60	75.8 (22.5)	74.0 [62–89]	16–141	4,547	29.7	ref
ART Initiation	Sex	Female	Pandemic	4	40.0 (18.9)	35.0 [30–44]	23–67	160	47.3	-47.2
ART Initiation	Sex	Female	War	8	35.0 (10.7)	35.0 [27–45]	18–47	280	30.5	-53.8
ART Initiation	Sex	Female	Post-war	9	48.0 (14.6)	49.0 [40–60]	22–65	432	30.3	-36.7
ART Initiation	Sex	Male	Pre-war	60	58.2 (23.0)	53.5 [43–69]	23–135	3,490	39.5	ref
ART Initiation	Sex	Male	Pandemic	4	29.2 (11.4)	25.0 [23–31]	21–46	117	38.8	-49.7
ART Initiation	Sex	Male	War	8	32.8 (7.3)	33.0 [30–36]	19–44	262	22.2	-43.7
ART Initiation	Sex	Male	Post-war	9	38.4 (11.6)	41.0 [40–45]	11–48	346	30.1	-33.9
ART Initiation	Age	0-24 years	Pre-war	60	22.4 (7.3)	22.0 [18–26]	4–46	1,347	32.7	ref
ART Initiation	Age	0-24 years	Pandemic	4	11.8 (7.2)	10.0 [9–13]	5–22	47	61.5	-47.7
ART Initiation	Age	0-24 years	War	8	10.6 (3.4)	12.0 [7–13]	6–15	85	32.2	-52.7
ART Initiation	Age	0-24 years	Post-war	9	15.6 (5.5)	17.0 [14–20]	6–21	140	35.1	-30.7
ART Initiation	Age	25-34 years	Pre-war	60	38.9 (5.9)	38.8 [35–42]	24–58	2,335	15.2	ref
ART Initiation	Age	25-34 years	Pandemic	4	27.2 (6.9)	28.8 [25–31]	18–34	108	25.2	-30.2
ART Initiation	Age	25-34 years	War	8	32.8 (5.5)	33.0 [28–37]	26–40	262	16.8	-15.8
ART Initiation	Age	25-34 years	Post-war	9	33.6 (4.2)	33.3 [31–36]	28–41	302	12.7	-13.7
ART Initiation	Age	35-44 years	Pre-war	60	45.2 (14.0)	41.0 [37–55]	15–75	2,715	31.0	ref
ART Initiation	Age	35-44 years	Pandemic	4	29.5 (9.9)	31.0 [27–33]	16–40	118	33.7	-34.8
ART Initiation	Age	35-44 years	War	8	22.2 (5.8)	23.0 [18–24]	14–33	178	25.8	-50.8
ART Initiation	Age	35-44 years	Post-war	9	25.1 (6.9)	27.0 [24–28]	9–32	226	27.3	-44.5
ART Initiation	Age	45+ years	Pre-war	60	23.6 (9.4)	22.0 [15–31]	10–42	1,416	39.8	ref
ART Initiation	Age	45+ years	Pandemic	4	10.5 (3.1)	11.5 [10–12]	6–13	42	29.6	-55.5
ART Initiation	Age	45+ years	War	8	13.1 (4.9)	15.5 [10–16]	5–18	105	37.2	-44.4
ART Initiation	Age	45+ years	Post-war	9	17.1 (5.2)	19.0 [14–20]	7–24	154	30.1	-27.5
ART Initiation	Facility level	Primary	Pre-war	60	32.8 (16.5)	33.5 [23–41]	0–72	1,966	50.2	ref
ART Initiation	Facility level	Primary	Pandemic	4	29.2 (13.5)	27.5 [20–37]	16–46	117	46.2	-10.7
ART Initiation	Facility level	Primary	War	8	17.8 (9.3)	16.5 [12–24]	5–33	142	52.4	-45.8
ART Initiation	Facility level	Primary	Post-war	9	27.8 (11.7)	32.0 [23–33]	6–43	250	42.3	-15.2
ART Initiation	Facility level	Secondary	Pre-war	60	75.7 (54.0)	48.5 [39–101]	16–233	4,541	71.3	ref
ART Initiation	Facility level	Secondary	Pandemic	4	27.0 (12.6)	26.5 [18–36]	14–41	108	46.8	-64.3

© 2026 Kebede HK et al. *JAMA Network Open*.

Outcome	Stratifier	Stratum	Period	n Q	Mean (SD)/Q	Median [IQR]/Q	Min–max/Q	Period total	CV %	Δ vs pre-war (%)
ART Initiation	Facility level	Secondary	War	8	37.1 (6.0)	37.0 [32–41]	30–47	297	16.1	-50.9
ART Initiation	Facility level	Secondary	Post-war	9	43.9 (11.3)	50.0 [36–52]	22–56	395	25.8	-42.0
ART Initiation	Facility level	Tertiary	Pre-war	60	25.5 (15.6)	27.0 [9–35]	1–66	1,530	61.1	ref
ART Initiation	Facility level	Tertiary	Pandemic	4	13.0 (8.7)	9.0 [9–13]	8–26	52	66.8	-49.0
ART Initiation	Facility level	Tertiary	War	8	12.9 (8.0)	14.5 [6–20]	2–22	103	62.1	-49.5
ART Initiation	Facility level	Tertiary	Post-war	9	14.8 (5.2)	16.0 [13–17]	5–23	133	35.1	-42.0
ART Initiation	Ownership	NGO	Pre-war	60	7.5 (6.1)	7.5 [2–11]	0–25	451	81.2	ref
ART Initiation	Ownership	NGO	Pandemic	4	11.8 (6.4)	9.5 [8–14]	7–21	47	54.4	+56.3
ART Initiation	Ownership	NGO	War	8	8.6 (2.3)	9.5 [8–10]	4–11	69	26.2	+14.7
ART Initiation	Ownership	NGO	Post-war	9	11.9 (3.3)	12.0 [10–14]	6–17	107	27.7	+58.2
ART Initiation	Ownership	Public	Pre-war	60	126.4 (44.4)	122.5 [96–152]	41–246	7,586	35.1	ref
ART Initiation	Ownership	Public	Pandemic	4	57.5 (24.4)	51.0 [44–65]	36–92	230	42.4	-54.5
ART Initiation	Ownership	Public	War	8	59.1 (15.9)	60.0 [48–72]	33–79	473	26.9	-53.2
ART Initiation	Ownership	Public	Post-war	9	74.6 (23.2)	81.0 [70–91]	27–97	671	31.1	-41.0

© 2026 Kebede HK et al. *JAMA Network Open*.

eTable 3. Prewar Secular Trend Analysis (Q1 2005 to Q4 2019)

Outcome	Slope (count/quarter)	SE	P value	R^2	Direction
HIV Diagnoses	-3.130	0.295	<.001	0.660	Decreasing
Linkage to Care	-2.395	0.315	<.001	0.500	Decreasing
ART Initiation	-1.161	0.278	<.001	0.232	Decreasing

Linear regression of quarterly count on time index for the 60 pre-war quarters.

© 2026 Kebede HK et al. *JAMA Network Open*.

eTable 4. Stratum Contribution to Total Cascade Volume by Period

Each cell shows the percentage of the period total contributed by that stratum within each of the 3 cascade outcomes.

eTable 4a. HIV Diagnoses

Stratifier	Stratum	Pre-war (%)	Pandemic (%)	War (%)	Post-war (%)
Sex	Female	58.0	57.1	51.5	56.1
Sex	Male	42.0	42.9	48.5	43.9
Age	0-24 years	16.0	15.8	14.9	18.2
Age	25-34 years	17.2	36.7	43.9	37.2
Age	35-44 years	32.1	39.9	33.1	28.7
Age	45+ years	17.1	14.9	18.9	19.2
Facility level	Primary	25.8	40.6	26.1	31.3
Facility level	Secondary	58.5	39.3	54.3	50.9
Facility level	Tertiary	15.7	20.1	19.6	17.8
Ownership	NGO	6.1	16.2	12.4	13.2
Ownership	Public	93.9	83.8	87.6	86.8

eTable 4b. Linkage to Care

Stratifier	Stratum	Pre-war (%)	Pandemic (%)	War (%)	Post-war (%)
Sex	Female	58.0	57.5	51.6	55.2
Sex	Male	42.0	42.5	48.4	44.8
Age	0-24 years	16.3	16.8	15.5	17.8
Age	25-34 years	19.5	38.3	47.4	38.1
Age	35-44 years	32.3	42.1	32.5	28.8
Age	45+ years	17.1	15.0	19.2	19.6
Facility level	Primary	26.3	42.5	26.5	31.8
Facility level	Secondary	59.0	38.9	54.5	50.5
Facility level	Tertiary	14.7	18.6	19.0	17.7
Ownership	NGO	6.3	17.1	13.2	13.6
Ownership	Public	93.7	82.9	86.8	86.4

eTable 4c. ART Initiation

Stratifier	Stratum	Pre-war (%)	Pandemic (%)	War (%)	Post-war (%)
Sex	Female	56.6	57.8	51.7	55.5
Sex	Male	43.4	42.2	48.3	44.5
Age	0-24 years	16.8	17.0	15.7	18.0
Age	25-34 years	29.1	39.2	48.4	38.9
Age	35-44 years	33.8	42.6	32.8	29.0
Age	45+ years	17.6	15.2	19.4	19.8
Facility level	Primary	24.5	42.2	26.2	32.1
Facility level	Secondary	56.5	39.0	54.8	50.8
Facility level	Tertiary	19.0	18.8	19.0	17.1
Ownership	NGO	5.6	17.0	12.7	13.8
Ownership	Public	94.4	83.0	87.3	86.2

© 2026 Kebede HK et al. *JAMA Network Open*.

eTable 5. Main Analysis: Negative Binomial Segmented Regression IRRs With DHARMa Diagnostics

Specification: $\log(Y_t) = \beta_0 + \beta_1 \cdot t + \beta_2 \cdot pandemic + \beta_3 \cdot war + \beta_4 \cdot post + \beta_5 \cdot sin + \beta_6 \cdot cos$. All IRRs reported relative to the pre-war period. Significance markers: *P<.05, **P<.01, ***P<.001. Total models = 36 (3 outcomes × 12 stratum-levels). DHARMa P values: dispersion test for over-/under-dispersion (P > .05 supports model fit); autocorrelation test for residual temporal autocorrelation. "—" indicates that the test is not applicable (quasi-Poisson models).

Outcome	Stratifier	Stratum	Fam	n obs	AIC	Pandemic IRR (95% CI)	P	War IRR (95% CI)	P	Post-war IRR (95% CI)	P	Disp P	Autocorr P
HIV Diagnoses	Overall	all	NB	81	807.4	0.65 (0.51-0.83)***	<.001	0.71 (0.58-0.86)***	<.001	0.98 (0.80-1.21)	0.86	0.52	<.001
HIV Diagnoses	Sex	Female	NB	81	731.8	0.60 (0.45-0.78)***	<.001	0.57 (0.46-0.72)***	<.001	0.85 (0.68-1.07)	0.18	0.82	<.001
HIV Diagnoses	Sex	Male	NB	81	680.0	0.74 (0.56-0.97)*	0.032	0.92 (0.74-1.15)	0.47	1.19 (0.95-1.49)	0.13	0.83	<.001
HIV Diagnoses	Age	0-24 years	NB	81	575.2	0.59 (0.40-0.88)**	0.009	0.59 (0.43-0.81)**	0.001	0.99 (0.72-1.34)	0.93	0.55	0.01
HIV Diagnoses	Age	25-34 years	QP	81	—	0.78 (0.66-0.92)**	0.004	0.91 (0.81-1.04)	0.17	0.92 (0.81-1.05)	0.24	—	—
HIV Diagnoses	Age	35-44 years	NB	81	628.4	0.84 (0.64-1.09)	0.18	0.76 (0.61-0.94)*	0.013	0.92 (0.73-1.15)	0.46	0.97	<.001
HIV Diagnoses	Age	45+ years	NB	81	560.6	0.60 (0.41-0.86)**	0.006	0.83 (0.64-1.09)	0.19	1.20 (0.91-1.57)	0.19	0.96	<.001
HIV Diagnoses	Facility level	Primary	NB	81	743.8	0.48 (0.26-0.92)*	0.026	0.29 (0.17-0.50)***	<.001	0.39 (0.22-0.71)**	0.002	0.03	<.001
HIV Diagnoses	Facility level	Secondary	NB	81	748.9	0.86 (0.61-1.22)	0.41	1.44 (1.09-1.90)**	0.009	2.16 (1.62-2.87)***	<.001	0.90	<.001
HIV Diagnoses	Facility level	Tertiary	NB	81	656.0	0.22 (0.12-0.43)***	<.001	0.18 (0.10-0.32)***	<.001	0.16 (0.09-0.29)***	<.001	0.58	<.001
HIV Diagnoses	Ownership	NGO	NB	81	521.5	0.37 (0.20-0.66)***	<.001	0.21 (0.13-0.36)***	<.001	0.20 (0.12-0.35)***	<.001	0.82	<.001
HIV Diagnoses	Ownership	Public	NB	81	801.7	0.65 (0.50-0.84)**	0.001	0.74 (0.60-0.92)**	0.006	1.05 (0.84-1.31)	0.65	0.53	<.001
Linkage to Care	Overall	all	NB	81	822.6	0.64 (0.48-0.86)**	0.003	0.68 (0.53-0.86)**	0.002	0.98 (0.76-1.26)	0.88	0.47	<.001
Linkage to Care	Sex	Female	NB	81	748.6	0.58 (0.42-0.81)**	0.001	0.55 (0.42-0.72)***	<.001	0.83 (0.63-1.11)	0.21	0.61	<.001
Linkage to Care	Sex	Male	NB	81	686.7	0.72 (0.53-0.99)*	0.044	0.89 (0.69-1.14)	0.36	1.22 (0.94-1.58)	0.13	0.79	<.001
Linkage to Care	Age	0-24 years	NB	81	570.6	0.53 (0.35-0.80)**	0.003	0.50 (0.36-0.70)***	<.001	0.79 (0.57-1.10)	0.16	0.80	0.07
Linkage to Care	Age	25-34 years	NB	81	496.2	0.76 (0.62-0.94)*	0.011	0.92 (0.78-1.08)	0.29	0.94 (0.79-1.11)	0.43	<.001	0.78
Linkage to Care	Age	35-44 years	NB	81	639.3	0.87 (0.65-1.18)	0.38	0.72 (0.56-0.93)*	0.012	0.93 (0.72-1.21)	0.59	0.86	<.001
Linkage to Care	Age	45+ years	NB	81	569.7	0.59 (0.39-0.89)*	0.012	0.81 (0.59-1.11)	0.18	1.21 (0.88-1.65)	0.25	0.87	<.001
Linkage to Care	Facility level	Primary	NB	81	742.3	0.47 (0.23-0.95)*	0.036	0.26 (0.14-0.49)***	<.001	0.37 (0.19-0.71)**	0.003	0.02	<.001
Linkage to Care	Facility level	Secondary	NB	81	749.1	0.84 (0.57-1.23)	0.36	1.38 (1.02-1.87)*	0.037	2.15 (1.57-2.95)***	<.001	0.82	<.001
Linkage to Care	Facility level	Tertiary	NB	81	632.5	0.21 (0.11-0.42)***	<.001	0.18 (0.10-0.32)***	<.001	0.16 (0.08-0.29)***	<.001	0.34	<.001
Linkage to Care	Ownership	NGO	NB	81	516.5	0.34 (0.18-0.65)**	0.001	0.19 (0.11-0.34)***	<.001	0.17 (0.09-0.32)***	<.001	0.85	<.001
Linkage to Care	Ownership	Public	NB	81	815.7	0.63 (0.46-0.86)**	0.004	0.71 (0.55-0.92)**	0.009	1.05 (0.80-1.38)	0.71	0.52	<.001
ART Initiation	Overall	all	NB	81	806.6	0.68 (0.50-0.94)*	0.021	0.70 (0.54-0.91)**	0.008	0.96 (0.73-1.26)	0.78	0.70	<.001
ART Initiation	Sex	Female	NB	81	723.9	0.63 (0.44-0.89)**	0.009	0.56 (0.42-0.75)***	<.001	0.81 (0.60-1.09)	0.16	0.62	<.001
ART Initiation	Sex	Male	NB	81	680.5	0.77 (0.54-1.09)	0.14	0.92 (0.70-1.22)	0.57	1.21 (0.90-1.62)	0.20	0.82	<.001
ART Initiation	Age	0-24 years	NB	81	547.5	0.51 (0.34-0.77)**	0.001	0.46 (0.32-0.64)***	<.001	0.66 (0.47-0.92)*	0.013	0.98	0.06
ART Initiation	Age	25-34 years	NB	81	524.7	0.70 (0.57-0.87)***	<.001	0.85 (0.73-0.99)*	0.041	0.87 (0.74-1.02)	0.096	0.40	0.44
ART Initiation	Age	35-44 years	NB	81	618.5	0.90 (0.66-1.22)	0.49	0.72 (0.55-0.92)*	0.011	0.88 (0.68-1.14)	0.34	0.99	<.001

© 2026 Kebede HK et al. *JAMA Network Open*.

Outcome	Stratifier	Stratum	Fam	n obs	AIC	Pandemic IRR (95% CI)	P	War IRR (95% CI)	P	Post-war IRR (95% CI)	P	Disp P	Autocorr P
ART Initiation	Age	45+ years	NB	81	557.6	0.62 (0.40-0.96)*	0.033	0.83 (0.59-1.15)	0.26	1.18 (0.84-1.65)	0.34	0.95	<.001
ART Initiation	Facility level	Primary	NB	81	685.5	0.52 (0.28-0.97)*	0.040	0.28 (0.17-0.48)***	<.001	0.37 (0.21-0.65)***	<.001	0.04	<.001
ART Initiation	Facility level	Secondary	NB	81	743.9	0.91 (0.58-1.43)	0.68	1.46 (1.02-2.09)*	0.039	2.16 (1.49-3.13)***	<.001	0.50	<.001
ART Initiation	Facility level	Tertiary	NB	81	630.7	0.22 (0.11-0.44)***	<.001	0.18 (0.10-0.33)***	<.001	0.15 (0.08-0.29)***	<.001	0.44	<.001
ART Initiation	Ownership	NGO	NB	81	430.0	0.48 (0.30-0.76)**	0.002	0.27 (0.18-0.40)***	<.001	0.25 (0.16-0.38)***	<.001	0.84	<.001
ART Initiation	Ownership	Public	NB	81	797.5	0.66 (0.47-0.92)*	0.015	0.72 (0.55-0.94)*	0.018	1.00 (0.75-1.33)	1.00	0.64	<.001

eTable 5a. Full segmented regression coefficient table: overall count models

Specification: $\log(E[Y_t]) = \beta_0 + \beta_1 \cdot t + \beta_2 \cdot \text{pandemic} + \beta_3 \cdot \text{war} + \beta_4 \cdot \text{post} + \beta_5 \cdot \sin(2\pi t/4) + \beta_6 \cdot \cos(2\pi t/4)$

Term	HIV Dx: β (SE)	HIV Dx: IRR (95% CI)	HIV Dx: P	Linkage: β (SE)	Linkage: IRR (95% CI)	Linkage: P	ART: β (SE)	ART: IRR (95% CI)	ART: P
β_0 Intercept	5.7463 (0.0522)	313.0 (282.6–346.8)	<.001	5.5696 (0.0654)	262.3 (230.8–298.2)	<.001	5.1374 (0.0711)	170.3 (148.1–195.7)	<.001
β_1 Time (per quarter)	-0.0159 (0.0015)	0.98 (0.98–0.99)	<.001	-0.0140 (0.0019)	0.99 (0.98–0.99)	<.001	-0.0083 (0.0020)	0.99 (0.99–1.00)	<.001
β_2 Pandemic period	-0.4264 (0.1241)	0.65 (0.51–0.83)	<.001	-0.4454 (0.1510)	0.64 (0.48–0.86)	0.003	-0.3800 (0.1644)	0.68 (0.50–0.94)	0.02
β_3 War period	-0.3486 (0.1011)	0.71 (0.58–0.86)	<.001	-0.3901 (0.1235)	0.68 (0.53–0.86)	0.002	-0.3577 (0.1340)	0.70 (0.54–0.91)	0.008
β_4 Post-war period	-0.0183 (0.1051)	0.98 (0.80–1.21)	0.86	-0.0187 (0.1291)	0.98 (0.76–1.26)	0.88	-0.0392 (0.1399)	0.96 (0.73–1.26)	0.78
β_5 sin(2πt/4) seasonal	-0.0322 (0.0326)	0.97 (0.91–1.03)	0.32	-0.0282 (0.0404)	0.97 (0.90–1.05)	0.48	-0.0311 (0.0449)	0.97 (0.89–1.06)	0.49
β_6 cos(2πt/4) seasonal	0.0342 (0.0326)	1.03 (0.97–1.10)	0.29	0.0465 (0.0402)	1.05 (0.97–1.13)	0.25	0.0410 (0.0445)	1.04 (0.95–1.14)	0.36

Coefficients are on the log-rate scale (β) and exponentiated to IRRs. Seasonal harmonics (sin, cos) are small (IRRs near 1.0) and non-significant; this confirms that removing the seasonal terms in Sensitivity Analysis 1 (eTable 8) leaves the intervention IRRs essentially unchanged.

© 2026 Kebede HK et al. *JAMA Network Open*.

eTable 5b. Full segmented regression coefficient table — all 36 stratified count models

All 7 coefficient terms (β_0 intercept, β_1 time, β_2 pandemic, β_3 war, β_4 post-war, β_5 sin, β_6 cos) for every count model. 36 models × 7 terms = 252 rows. Use this table to verify any specific stratum-level β/SE/IRR. The first 3 outcome-level overall models are also tabulated in compact form in eTable 5a.

Outcome	Stratifier	Stratum	Term	β	SE	IRR (95% CI)	P
HIV Diagnoses	Overall	all	β_0 Intercept	5.7463	0.0522	313.0 (282.6–346.8)	<.001
HIV Diagnoses	Overall	all	β_1 Time (per quarter)	-0.0159	0.0015	0.98 (0.98–0.99)	<.001
HIV Diagnoses	Overall	all	β_2 Pandemic period	-0.4264	0.1241	0.65 (0.51–0.83)	<.001
HIV Diagnoses	Overall	all	β_3 War period	-0.3486	0.1011	0.71 (0.58–0.86)	<.001
HIV Diagnoses	Overall	all	β_4 Post-war period	-0.0183	0.1051	0.98 (0.80–1.21)	0.86
HIV Diagnoses	Overall	all	β_5 sin(2πt/4) seasonal	-0.0322	0.0326	0.97 (0.91–1.03)	0.32
HIV Diagnoses	Overall	all	β_6 cos(2πt/4) seasonal	0.0342	0.0326	1.03 (0.97–1.10)	0.29
HIV Diagnoses	Sex	Female	β_0 Intercept	5.1492	0.0569	172.3 (154.1–192.6)	<.001
HIV Diagnoses	Sex	Female	β_1 Time (per quarter)	-0.0138	0.0017	0.99 (0.98–0.99)	<.001
HIV Diagnoses	Sex	Female	β_2 Pandemic period	-0.5190	0.1393	0.60 (0.45–0.78)	<.001
HIV Diagnoses	Sex	Female	β_3 War period	-0.5543	0.1142	0.57 (0.46–0.72)	<.001
HIV Diagnoses	Sex	Female	β_4 Post-war period	-0.1568	0.1165	0.85 (0.68–1.07)	0.18
HIV Diagnoses	Sex	Female	β_5 sin(2πt/4) seasonal	-0.0396	0.0360	0.96 (0.90–1.03)	0.27
HIV Diagnoses	Sex	Female	β_6 cos(2πt/4) seasonal	0.0377	0.0353	1.04 (0.97–1.11)	0.29
HIV Diagnoses	Sex	Male	β_0 Intercept	4.9484	0.0531	140.9 (127.0–156.4)	<.001
HIV Diagnoses	Sex	Male	β_1 Time (per quarter)	-0.0187	0.0016	0.98 (0.98–0.98)	<.001
HIV Diagnoses	Sex	Male	β_2 Pandemic period	-0.2988	0.1393	0.74 (0.56–0.97)	0.03
HIV Diagnoses	Sex	Male	β_3 War period	-0.0802	0.1102	0.92 (0.74–1.15)	0.47
HIV Diagnoses	Sex	Male	β_4 Post-war period	0.1733	0.1140	1.19 (0.95–1.49)	0.13
HIV Diagnoses	Sex	Male	β_5 sin(2πt/4) seasonal	-0.0431	0.0346	0.96 (0.90–1.02)	0.21
HIV Diagnoses	Sex	Male	β_6 cos(2πt/4) seasonal	0.0506	0.0347	1.05 (0.98–1.13)	0.14
HIV Diagnoses	Age	0-24 years	β_0 Intercept	3.8464	0.0733	46.83 (40.56–54.06)	<.001
HIV Diagnoses	Age	0-24 years	β_1 Time (per quarter)	-0.0133	0.0022	0.99 (0.98–0.99)	<.001
HIV Diagnoses	Age	0-24 years	β_2 Pandemic period	-0.5252	0.2000	0.59 (0.40–0.88)	0.009
HIV Diagnoses	Age	0-24 years	β_3 War period	-0.5242	0.1621	0.59 (0.43–0.81)	0.001
HIV Diagnoses	Age	0-24 years	β_4 Post-war period	-0.0134	0.1575	0.99 (0.72–1.34)	0.93
HIV Diagnoses	Age	0-24 years	β_5 sin(2πt/4) seasonal	-0.0691	0.0476	0.93 (0.85–1.02)	0.15
HIV Diagnoses	Age	0-24 years	β_6 cos(2πt/4) seasonal	0.0258	0.0469	1.03 (0.94–1.12)	0.58
HIV Diagnoses	Age	25-34 years	β_0 Intercept	3.5109	0.0356	33.48 (31.22–35.90)	<.001
HIV Diagnoses	Age	25-34 years	β_1 Time (per quarter)	0.0010	0.0010	1.00 (1.00–1.00)	0.31
HIV Diagnoses	Age	25-34 years	β_2 Pandemic period	-0.2497	0.0838	0.78 (0.66–0.92)	0.004
HIV Diagnoses	Age	25-34 years	β_3 War period	-0.0898	0.0645	0.91 (0.81–1.04)	0.17
HIV Diagnoses	Age	25-34 years	β_4 Post-war period	-0.0796	0.0677	0.92 (0.81–1.05)	0.24
HIV Diagnoses	Age	25-34 years	β_5 sin(2πt/4) seasonal	0.0196	0.0214	1.02 (0.98–1.06)	0.36
HIV Diagnoses	Age	25-34 years	β_6 cos(2πt/4) seasonal	-0.0040	0.0216	1.00 (0.95–1.04)	0.85
HIV Diagnoses	Age	35-44 years	β_0 Intercept	4.6323	0.0504	102.7 (93.08–113.4)	<.001
HIV Diagnoses	Age	35-44 years	β_1 Time (per quarter)	-0.0167	0.0015	0.98 (0.98–0.99)	<.001
HIV Diagnoses	Age	35-44 years	β_2 Pandemic period	-0.1770	0.1335	0.84 (0.64–1.09)	0.18
HIV Diagnoses	Age	35-44 years	β_3 War period	-0.2786	0.1117	0.76 (0.61–0.94)	0.01
HIV Diagnoses	Age	35-44 years	β_4 Post-war period	-0.0855	0.1147	0.92 (0.73–1.15)	0.46
HIV Diagnoses	Age	35-44 years	β_5 sin(2πt/4) seasonal	-0.0343	0.0334	0.97 (0.91–1.03)	0.30
HIV Diagnoses	Age	35-44 years	β_6 cos(2πt/4) seasonal	0.0284	0.0337	1.03 (0.96–1.10)	0.40

© 2026 Kebede HK et al. *JAMA Network Open*.

Outcome	Stratifier	Stratum	Term	β	SE	IRR (95% CI)	P
HIV Diagnoses	Age	45+ years	β_0 Intercept	4.0221	0.0606	55.82 (49.57–62.85)	<.001
HIV Diagnoses	Age	45+ years	β_1 Time (per quarter)	-0.0174	0.0019	0.98 (0.98–0.99)	<.001
HIV Diagnoses	Age	45+ years	β_2 Pandemic period	-0.5143	0.1866	0.60 (0.41–0.86)	0.006
HIV Diagnoses	Age	45+ years	β_3 War period	-0.1819	0.1386	0.83 (0.64–1.09)	0.19
HIV Diagnoses	Age	45+ years	β_4 Post-war period	0.1792	0.1381	1.20 (0.91–1.57)	0.19
HIV Diagnoses	Age	45+ years	β_5 $\sin(2\pi t/4)$ seasonal	-0.0723	0.0407	0.93 (0.86–1.01)	0.08
HIV Diagnoses	Age	45+ years	β_6 $\cos(2\pi t/4)$ seasonal	0.0571	0.0408	1.06 (0.98–1.15)	0.16
HIV Diagnoses	Facility level	Primary	β_0 Intercept	3.7194	0.1608	41.24 (30.09–56.52)	<.001
HIV Diagnoses	Facility level	Primary	β_1 Time (per quarter)	0.0072	0.0047	1.01 (1.00–1.02)	0.13
HIV Diagnoses	Facility level	Primary	β_2 Pandemic period	-0.7253	0.3256	0.48 (0.26–0.92)	0.03
HIV Diagnoses	Facility level	Primary	β_3 War period	-1.2420	0.2787	0.29 (0.17–0.50)	<.001
HIV Diagnoses	Facility level	Primary	β_4 Post-war period	-0.9304	0.2990	0.39 (0.22–0.71)	0.002
HIV Diagnoses	Facility level	Primary	β_5 $\sin(2\pi t/4)$ seasonal	-0.0508	0.0856	0.95 (0.80–1.12)	0.55
HIV Diagnoses	Facility level	Primary	β_6 $\cos(2\pi t/4)$ seasonal	0.0307	0.0889	1.03 (0.87–1.23)	0.73
HIV Diagnoses	Facility level	Secondary	β_0 Intercept	5.5949	0.0694	269.1 (234.8–308.3)	<.001
HIV Diagnoses	Facility level	Secondary	β_1 Time (per quarter)	-0.0330	0.0020	0.97 (0.96–0.97)	<.001
HIV Diagnoses	Facility level	Secondary	β_2 Pandemic period	-0.1486	0.1788	0.86 (0.61–1.22)	0.41
HIV Diagnoses	Facility level	Secondary	β_3 War period	0.3649	0.1402	1.44 (1.09–1.90)	0.009
HIV Diagnoses	Facility level	Secondary	β_4 Post-war period	0.7699	0.1455	2.16 (1.62–2.87)	<.001
HIV Diagnoses	Facility level	Secondary	β_5 $\sin(2\pi t/4)$ seasonal	-0.0393	0.0462	0.96 (0.88–1.05)	0.39
HIV Diagnoses	Facility level	Secondary	β_6 $\cos(2\pi t/4)$ seasonal	0.0747	0.0457	1.08 (0.99–1.18)	0.10
HIV Diagnoses	Facility level	Tertiary	β_0 Intercept	2.5469	0.1743	12.77 (9.07–17.97)	<.001
HIV Diagnoses	Facility level	Tertiary	β_1 Time (per quarter)	0.0270	0.0051	1.03 (1.02–1.04)	<.001
HIV Diagnoses	Facility level	Tertiary	β_2 Pandemic period	-1.4985	0.3379	0.22 (0.12–0.43)	<.001
HIV Diagnoses	Facility level	Tertiary	β_3 War period	-1.6933	0.2877	0.18 (0.10–0.32)	<.001
HIV Diagnoses	Facility level	Tertiary	β_4 Post-war period	-1.8558	0.3109	0.16 (0.09–0.29)	<.001
HIV Diagnoses	Facility level	Tertiary	β_5 $\sin(2\pi t/4)$ seasonal	-0.0165	0.0886	0.98 (0.83–1.17)	0.85
HIV Diagnoses	Facility level	Tertiary	β_6 $\cos(2\pi t/4)$ seasonal	-0.0573	0.0884	0.94 (0.79–1.12)	0.52
HIV Diagnoses	Ownership	NGO	β_0 Intercept	1.2017	0.1761	3.33 (2.36–4.70)	<.001
HIV Diagnoses	Ownership	NGO	β_1 Time (per quarter)	0.0375	0.0048	1.04 (1.03–1.05)	<.001
HIV Diagnoses	Ownership	NGO	β_2 Pandemic period	-1.0056	0.3029	0.37 (0.20–0.66)	<.001
HIV Diagnoses	Ownership	NGO	β_3 War period	-1.5498	0.2652	0.21 (0.13–0.36)	<.001
HIV Diagnoses	Ownership	NGO	β_4 Post-war period	-1.6062	0.2824	0.20 (0.12–0.35)	<.001
HIV Diagnoses	Ownership	NGO	β_5 $\sin(2\pi t/4)$ seasonal	-0.1211	0.0842	0.89 (0.75–1.04)	0.15
HIV Diagnoses	Ownership	NGO	β_6 $\cos(2\pi t/4)$ seasonal	-0.0441	0.0848	0.96 (0.81–1.13)	0.60
HIV Diagnoses	Ownership	Public	β_0 Intercept	5.7548	0.0552	315.7 (283.3–351.8)	<.001
HIV Diagnoses	Ownership	Public	β_1 Time (per quarter)	-0.0187	0.0016	0.98 (0.98–0.98)	<.001
HIV Diagnoses	Ownership	Public	β_2 Pandemic period	-0.4347	0.1330	0.65 (0.50–0.84)	0.001
HIV Diagnoses	Ownership	Public	β_3 War period	-0.2954	0.1077	0.74 (0.60–0.92)	0.006
HIV Diagnoses	Ownership	Public	β_4 Post-war period	0.0501	0.1119	1.05 (0.84–1.31)	0.65
HIV Diagnoses	Ownership	Public	β_5 $\sin(2\pi t/4)$ seasonal	-0.0290	0.0348	0.97 (0.91–1.04)	0.40
HIV Diagnoses	Ownership	Public	β_6 $\cos(2\pi t/4)$ seasonal	0.0448	0.0347	1.05 (0.98–1.12)	0.20
Linkage to Care	Overall	all	β_0 Intercept	5.5696	0.0654	262.3 (230.8–298.2)	<.001
Linkage to Care	Overall	all	β_1 Time (per quarter)	-0.0140	0.0019	0.99 (0.98–0.99)	<.001
Linkage to Care	Overall	all	β_2 Pandemic period	-0.4454	0.1510	0.64 (0.48–0.86)	0.003
Linkage to Care	Overall	all	β_3 War period	-0.3901	0.1235	0.68 (0.53–0.86)	0.002
Linkage to Care	Overall	all	β_4 Post-war period	-0.0187	0.1291	0.98 (0.76–1.26)	0.88

© 2026 Kebede HK et al. *JAMA Network Open*.

Outcome	Stratifier	Stratum	Term	β	SE	IRR (95% CI)	P
Linkage to Care	Overall	all	β_5 sin(2πt/4) seasonal	-0.0282	0.0404	0.97 (0.90–1.05)	0.48
Linkage to Care	Overall	all	β_6 cos(2πt/4) seasonal	0.0465	0.0402	1.05 (0.97–1.13)	0.25
Linkage to Care	Sex	Female	β_0 Intercept	4.9646	0.0725	143.3 (124.3–165.1)	<.001
Linkage to Care	Sex	Female	β_1 Time (per quarter)	-0.0117	0.0021	0.99 (0.98–0.99)	<.001
Linkage to Care	Sex	Female	β_2 Pandemic period	-0.5404	0.1696	0.58 (0.42–0.81)	0.001
Linkage to Care	Sex	Female	β_3 War period	-0.6017	0.1397	0.55 (0.42–0.72)	<.001
Linkage to Care	Sex	Female	β_4 Post-war period	-0.1822	0.1441	0.83 (0.63–1.11)	0.21
Linkage to Care	Sex	Female	β_5 sin(2πt/4) seasonal	-0.0374	0.0452	0.96 (0.88–1.05)	0.41
Linkage to Care	Sex	Female	β_6 cos(2πt/4) seasonal	0.0553	0.0438	1.06 (0.97–1.15)	0.21
Linkage to Care	Sex	Male	β_0 Intercept	4.7782	0.0634	118.9 (105.0–134.6)	<.001
Linkage to Care	Sex	Male	β_1 Time (per quarter)	-0.0170	0.0019	0.98 (0.98–0.99)	<.001
Linkage to Care	Sex	Male	β_2 Pandemic period	-0.3225	0.1598	0.72 (0.53–0.99)	0.04
Linkage to Care	Sex	Male	β_3 War period	-0.1173	0.1274	0.89 (0.69–1.14)	0.36
Linkage to Care	Sex	Male	β_4 Post-war period	0.2000	0.1316	1.22 (0.94–1.58)	0.13
Linkage to Care	Sex	Male	β_5 sin(2πt/4) seasonal	-0.0379	0.0407	0.96 (0.89–1.04)	0.35
Linkage to Care	Sex	Male	β_6 cos(2πt/4) seasonal	0.0604	0.0407	1.06 (0.98–1.15)	0.14
Linkage to Care	Age	0-24 years	β_0 Intercept	3.5859	0.0802	36.09 (30.84–42.23)	<.001
Linkage to Care	Age	0-24 years	β_1 Time (per quarter)	-0.0079	0.0023	0.99 (0.99–1.00)	<.001
Linkage to Care	Age	0-24 years	β_2 Pandemic period	-0.6309	0.2095	0.53 (0.35–0.80)	0.003
Linkage to Care	Age	0-24 years	β_3 War period	-0.6853	0.1709	0.50 (0.36–0.70)	<.001
Linkage to Care	Age	0-24 years	β_4 Post-war period	-0.2357	0.1672	0.79 (0.57–1.10)	0.16
Linkage to Care	Age	0-24 years	β_5 sin(2πt/4) seasonal	-0.0011	0.0511	1.00 (0.90–1.10)	0.98
Linkage to Care	Age	0-24 years	β_6 cos(2πt/4) seasonal	0.0420	0.0505	1.04 (0.94–1.15)	0.41
Linkage to Care	Age	25-34 years	β_0 Intercept	3.5201	0.0447	33.79 (30.95–36.88)	<.001
Linkage to Care	Age	25-34 years	β_1 Time (per quarter)	0.0007	0.0013	1.00 (1.00–1.00)	0.60
Linkage to Care	Age	25-34 years	β_2 Pandemic period	-0.2720	0.1069	0.76 (0.62–0.94)	0.01
Linkage to Care	Age	25-34 years	β_3 War period	-0.0860	0.0815	0.92 (0.78–1.08)	0.29
Linkage to Care	Age	25-34 years	β_4 Post-war period	-0.0668	0.0854	0.94 (0.79–1.11)	0.43
Linkage to Care	Age	25-34 years	β_5 sin(2πt/4) seasonal	0.0065	0.0269	1.01 (0.95–1.06)	0.81
Linkage to Care	Age	25-34 years	β_6 cos(2πt/4) seasonal	-0.0043	0.0272	1.00 (0.94–1.05)	0.87
Linkage to Care	Age	35-44 years	β_0 Intercept	4.4717	0.0621	87.51 (77.48–98.83)	<.001
Linkage to Care	Age	35-44 years	β_1 Time (per quarter)	-0.0152	0.0018	0.98 (0.98–0.99)	<.001
Linkage to Care	Age	35-44 years	β_2 Pandemic period	-0.1348	0.1541	0.87 (0.65–1.18)	0.38
Linkage to Care	Age	35-44 years	β_3 War period	-0.3285	0.1305	0.72 (0.56–0.93)	0.01
Linkage to Care	Age	35-44 years	β_4 Post-war period	-0.0717	0.1337	0.93 (0.72–1.21)	0.59
Linkage to Care	Age	35-44 years	β_5 sin(2πt/4) seasonal	-0.0251	0.0401	0.98 (0.90–1.05)	0.53
Linkage to Care	Age	35-44 years	β_6 cos(2πt/4) seasonal	0.0296	0.0402	1.03 (0.95–1.11)	0.46
Linkage to Care	Age	45+ years	β_0 Intercept	3.8387	0.0753	46.46 (40.08–53.86)	<.001
Linkage to Care	Age	45+ years	β_1 Time (per quarter)	-0.0153	0.0022	0.98 (0.98–0.99)	<.001
Linkage to Care	Age	45+ years	β_2 Pandemic period	-0.5316	0.2121	0.59 (0.39–0.89)	0.01
Linkage to Care	Age	45+ years	β_3 War period	-0.2144	0.1610	0.81 (0.59–1.11)	0.18
Linkage to Care	Age	45+ years	β_4 Post-war period	0.1868	0.1615	1.21 (0.88–1.65)	0.25
Linkage to Care	Age	45+ years	β_5 sin(2πt/4) seasonal	-0.0641	0.0490	0.94 (0.85–1.03)	0.19
Linkage to Care	Age	45+ years	β_6 cos(2πt/4) seasonal	0.0776	0.0493	1.08 (0.98–1.19)	0.12
Linkage to Care	Facility level	Primary	β_0 Intercept	3.4879	0.1821	32.72 (22.90–46.74)	<.001
Linkage to Care	Facility level	Primary	β_1 Time (per quarter)	0.0109	0.0053	1.01 (1.00–1.02)	0.04
Linkage to Care	Facility level	Primary	β_2 Pandemic period	-0.7607	0.3633	0.47 (0.23–0.95)	0.04

© 2026 Kebede HK et al. *JAMA Network Open*.

Outcome	Stratifier	Stratum	Term	β	SE	IRR (95% CI)	P
Linkage to Care	Facility level	Primary	β_3 War period	-1.3337	0.3114	0.26 (0.14–0.49)	<.001
Linkage to Care	Facility level	Primary	β_4 Post-war period	-1.0064	0.3357	0.37 (0.19–0.71)	0.003
Linkage to Care	Facility level	Primary	β_5 sin(2πt/4) seasonal	-0.0376	0.0956	0.96 (0.80–1.16)	0.69
Linkage to Care	Facility level	Primary	β_6 cos(2πt/4) seasonal	0.0309	0.0990	1.03 (0.85–1.25)	0.76
Linkage to Care	Facility level	Secondary	β_0 Intercept	5.4496	0.0782	232.7 (199.6–271.2)	<.001
Linkage to Care	Facility level	Secondary	β_1 Time (per quarter)	-0.0315	0.0023	0.97 (0.96–0.97)	<.001
Linkage to Care	Facility level	Secondary	β_2 Pandemic period	-0.1803	0.1969	0.84 (0.57–1.23)	0.36
Linkage to Care	Facility level	Secondary	β_3 War period	0.3231	0.1553	1.38 (1.02–1.87)	0.04
Linkage to Care	Facility level	Secondary	β_4 Post-war period	0.7656	0.1612	2.15 (1.57–2.95)	<.001
Linkage to Care	Facility level	Secondary	β_5 sin(2πt/4) seasonal	-0.0370	0.0512	0.96 (0.87–1.07)	0.47
Linkage to Care	Facility level	Secondary	β_6 cos(2πt/4) seasonal	0.0700	0.0507	1.07 (0.97–1.18)	0.17
Linkage to Care	Facility level	Tertiary	β_0 Intercept	2.2064	0.1828	9.08 (6.35–13.00)	<.001
Linkage to Care	Facility level	Tertiary	β_1 Time (per quarter)	0.0310	0.0053	1.03 (1.02–1.04)	<.001
Linkage to Care	Facility level	Tertiary	β_2 Pandemic period	-1.5536	0.3529	0.21 (0.11–0.42)	<.001
Linkage to Care	Facility level	Tertiary	β_3 War period	-1.7239	0.2990	0.18 (0.10–0.32)	<.001
Linkage to Care	Facility level	Tertiary	β_4 Post-war period	-1.8574	0.3222	0.16 (0.08–0.29)	<.001
Linkage to Care	Facility level	Tertiary	β_5 sin(2πt/4) seasonal	-0.0361	0.0949	0.96 (0.80–1.16)	0.70
Linkage to Care	Facility level	Tertiary	β_6 cos(2πt/4) seasonal	0.0349	0.0906	1.04 (0.87–1.24)	0.70
Linkage to Care	Ownership	NGO	β_0 Intercept	0.8856	0.2033	2.42 (1.63–3.61)	<.001
Linkage to Care	Ownership	NGO	β_1 Time (per quarter)	0.0435	0.0056	1.04 (1.03–1.06)	<.001
Linkage to Care	Ownership	NGO	β_2 Pandemic period	-1.0812	0.3352	0.34 (0.18–0.65)	0.001
Linkage to Care	Ownership	NGO	β_3 War period	-1.6642	0.2958	0.19 (0.11–0.34)	<.001
Linkage to Care	Ownership	NGO	β_4 Post-war period	-1.7503	0.3186	0.17 (0.09–0.32)	<.001
Linkage to Care	Ownership	NGO	β_5 sin(2πt/4) seasonal	-0.1044	0.0929	0.90 (0.75–1.08)	0.26
Linkage to Care	Ownership	NGO	β_6 cos(2πt/4) seasonal	-0.0304	0.0937	0.97 (0.81–1.17)	0.75
Linkage to Care	Ownership	Public	β_0 Intercept	5.5804	0.0689	265.2 (231.7–303.5)	<.001
Linkage to Care	Ownership	Public	β_1 Time (per quarter)	-0.0169	0.0020	0.98 (0.98–0.99)	<.001
Linkage to Care	Ownership	Public	β_2 Pandemic period	-0.4609	0.1610	0.63 (0.46–0.86)	0.004
Linkage to Care	Ownership	Public	β_3 War period	-0.3410	0.1310	0.71 (0.55–0.92)	0.009
Linkage to Care	Ownership	Public	β_4 Post-war period	0.0512	0.1368	1.05 (0.80–1.38)	0.71
Linkage to Care	Ownership	Public	β_5 sin(2πt/4) seasonal	-0.0242	0.0429	0.98 (0.90–1.06)	0.57
Linkage to Care	Ownership	Public	β_6 cos(2πt/4) seasonal	0.0571	0.0426	1.06 (0.97–1.15)	0.18
ART Initiation	Overall	all	β_0 Intercept	5.1374	0.0711	170.3 (148.1–195.7)	<.001
ART Initiation	Overall	all	β_1 Time (per quarter)	-0.0083	0.0020	0.99 (0.99–1.00)	<.001
ART Initiation	Overall	all	β_2 Pandemic period	-0.3800	0.1644	0.68 (0.50–0.94)	0.02
ART Initiation	Overall	all	β_3 War period	-0.3577	0.1340	0.70 (0.54–0.91)	0.008
ART Initiation	Overall	all	β_4 Post-war period	-0.0392	0.1399	0.96 (0.73–1.26)	0.78
ART Initiation	Overall	all	β_5 sin(2πt/4) seasonal	-0.0311	0.0449	0.97 (0.89–1.06)	0.49
ART Initiation	Overall	all	β_6 cos(2πt/4) seasonal	0.0410	0.0445	1.04 (0.95–1.14)	0.36
ART Initiation	Sex	Female	β_0 Intercept	4.4799	0.0759	88.22 (76.02–102.4)	<.001
ART Initiation	Sex	Female	β_1 Time (per quarter)	-0.0051	0.0022	0.99 (0.99–1.00)	0.02
ART Initiation	Sex	Female	β_2 Pandemic period	-0.4657	0.1770	0.63 (0.44–0.89)	0.009
ART Initiation	Sex	Female	β_3 War period	-0.5728	0.1453	0.56 (0.42–0.75)	<.001
ART Initiation	Sex	Female	β_4 Post-war period	-0.2099	0.1498	0.81 (0.60–1.09)	0.16
ART Initiation	Sex	Female	β_5 sin(2πt/4) seasonal	-0.0296	0.0478	0.97 (0.88–1.07)	0.54
ART Initiation	Sex	Female	β_6 cos(2πt/4) seasonal	0.0269	0.0472	1.03 (0.94–1.13)	0.57
ART Initiation	Sex	Male	β_0 Intercept	4.4141	0.0732	82.61 (71.57–95.34)	<.001

© 2026 Kebede HK et al. *JAMA Network Open*.

Outcome	Stratifier	Stratum	Term	β	SE	IRR (95% CI)	P
ART Initiation	Sex	Male	β_1 Time (per quarter)	-0.0123	0.0021	0.99 (0.98–0.99)	<.001
ART Initiation	Sex	Male	β_2 Pandemic period	-0.2631	0.1790	0.77 (0.54–1.09)	0.14
ART Initiation	Sex	Male	β_3 War period	-0.0809	0.1433	0.92 (0.70–1.22)	0.57
ART Initiation	Sex	Male	β_4 Post-war period	0.1903	0.1488	1.21 (0.90–1.62)	0.20
ART Initiation	Sex	Male	β_5 sin(2πt/4) seasonal	-0.0406	0.0472	0.96 (0.88–1.05)	0.39
ART Initiation	Sex	Male	β_6 cos(2πt/4) seasonal	0.0576	0.0471	1.06 (0.97–1.16)	0.22
ART Initiation	Age	0-24 years	β_0 Intercept	3.0783	0.0855	21.72 (18.37–25.68)	<.001
ART Initiation	Age	0-24 years	β_1 Time (per quarter)	0.0010	0.0024	1.00 (1.00–1.01)	0.67
ART Initiation	Age	0-24 years	β_2 Pandemic period	-0.6782	0.2103	0.51 (0.34–0.77)	0.001
ART Initiation	Age	0-24 years	β_3 War period	-0.7873	0.1720	0.46 (0.32–0.64)	<.001
ART Initiation	Age	0-24 years	β_4 Post-war period	-0.4180	0.1690	0.66 (0.47–0.92)	0.01
ART Initiation	Age	0-24 years	β_5 sin(2πt/4) seasonal	0.0105	0.0526	1.01 (0.91–1.12)	0.84
ART Initiation	Age	0-24 years	β_6 cos(2πt/4) seasonal	0.0599	0.0529	1.06 (0.96–1.18)	0.26
ART Initiation	Age	25-34 years	β_0 Intercept	3.6690	0.0418	39.21 (36.13–42.56)	<.001
ART Initiation	Age	25-34 years	β_1 Time (per quarter)	-0.0003	0.0012	1.00 (1.00–1.00)	0.83
ART Initiation	Age	25-34 years	β_2 Pandemic period	-0.3511	0.1053	0.70 (0.57–0.87)	<.001
ART Initiation	Age	25-34 years	β_3 War period	-0.1626	0.0795	0.85 (0.73–0.99)	0.04
ART Initiation	Age	25-34 years	β_4 Post-war period	-0.1379	0.0828	0.87 (0.74–1.02)	0.10
ART Initiation	Age	25-34 years	β_5 sin(2πt/4) seasonal	0.0214	0.0257	1.02 (0.97–1.07)	0.40
ART Initiation	Age	25-34 years	β_6 cos(2πt/4) seasonal	-0.0049	0.0260	1.00 (0.95–1.05)	0.85
ART Initiation	Age	35-44 years	β_0 Intercept	4.0871	0.0641	59.57 (52.54–67.54)	<.001
ART Initiation	Age	35-44 years	β_1 Time (per quarter)	-0.0095	0.0019	0.99 (0.99–0.99)	<.001
ART Initiation	Age	35-44 years	β_2 Pandemic period	-0.1072	0.1543	0.90 (0.66–1.22)	0.49
ART Initiation	Age	35-44 years	β_3 War period	-0.3353	0.1311	0.72 (0.55–0.92)	0.01
ART Initiation	Age	35-44 years	β_4 Post-war period	-0.1292	0.1345	0.88 (0.68–1.14)	0.34
ART Initiation	Age	35-44 years	β_5 sin(2πt/4) seasonal	-0.0357	0.0412	0.96 (0.89–1.05)	0.39
ART Initiation	Age	35-44 years	β_6 cos(2πt/4) seasonal	0.0351	0.0411	1.04 (0.96–1.12)	0.39
ART Initiation	Age	45+ years	β_0 Intercept	3.4482	0.0837	31.44 (26.69–37.05)	<.001
ART Initiation	Age	45+ years	β_1 Time (per quarter)	-0.0100	0.0024	0.99 (0.99–0.99)	<.001
ART Initiation	Age	45+ years	β_2 Pandemic period	-0.4760	0.2229	0.62 (0.40–0.96)	0.03
ART Initiation	Age	45+ years	β_3 War period	-0.1912	0.1709	0.83 (0.59–1.15)	0.26
ART Initiation	Age	45+ years	β_4 Post-war period	0.1642	0.1727	1.18 (0.84–1.65)	0.34
ART Initiation	Age	45+ years	β_5 sin(2πt/4) seasonal	-0.0741	0.0545	0.93 (0.83–1.03)	0.17
ART Initiation	Age	45+ years	β_6 cos(2πt/4) seasonal	0.0584	0.0542	1.06 (0.95–1.18)	0.28
ART Initiation	Facility level	Primary	β_0 Intercept	2.8754	0.1562	17.73 (13.06–24.08)	<.001
ART Initiation	Facility level	Primary	β_1 Time (per quarter)	0.0187	0.0045	1.02 (1.01–1.03)	<.001
ART Initiation	Facility level	Primary	β_2 Pandemic period	-0.6523	0.3168	0.52 (0.28–0.97)	0.04
ART Initiation	Facility level	Primary	β_3 War period	-1.2659	0.2699	0.28 (0.17–0.48)	<.001
ART Initiation	Facility level	Primary	β_4 Post-war period	-0.9972	0.2871	0.37 (0.21–0.65)	<.001
ART Initiation	Facility level	Primary	β_5 sin(2πt/4) seasonal	-0.0184	0.0855	0.98 (0.83–1.16)	0.83
ART Initiation	Facility level	Primary	β_6 cos(2πt/4) seasonal	0.0166	0.0881	1.02 (0.86–1.21)	0.85
ART Initiation	Facility level	Secondary	β_0 Intercept	4.9944	0.0929	147.6 (123.0–177.1)	<.001
ART Initiation	Facility level	Secondary	β_1 Time (per quarter)	-0.0257	0.0026	0.97 (0.97–0.98)	<.001
ART Initiation	Facility level	Secondary	β_2 Pandemic period	-0.0952	0.2308	0.91 (0.58–1.43)	0.68
ART Initiation	Facility level	Secondary	β_3 War period	0.3783	0.1829	1.46 (1.02–2.09)	0.04
ART Initiation	Facility level	Secondary	β_4 Post-war period	0.7696	0.1899	2.16 (1.49–3.13)	<.001
ART Initiation	Facility level	Secondary	β_5 sin(2πt/4) seasonal	-0.0456	0.0621	0.96 (0.85–1.08)	0.46

© 2026 Kebede HK et al. *JAMA Network Open*.

Outcome	Stratifier	Stratum	Term	β	SE	IRR (95% CI)	P
ART Initiation	Facility level	Secondary	β_6 cos(2πt/4) seasonal	0.0680	0.0618	1.07 (0.95–1.21)	0.27
ART Initiation	Facility level	Tertiary	β_0 Intercept	2.2006	0.1846	9.03 (6.29–12.97)	<.001
ART Initiation	Facility level	Tertiary	β_1 Time (per quarter)	0.0307	0.0054	1.03 (1.02–1.04)	<.001
ART Initiation	Facility level	Tertiary	β_2 Pandemic period	-1.5286	0.3558	0.22 (0.11–0.44)	<.001
ART Initiation	Facility level	Tertiary	β_3 War period	-1.7078	0.3018	0.18 (0.10–0.33)	<.001
ART Initiation	Facility level	Tertiary	β_4 Post-war period	-1.8718	0.3256	0.15 (0.08–0.29)	<.001
ART Initiation	Facility level	Tertiary	β_5 sin(2πt/4) seasonal	-0.0379	0.0960	0.96 (0.80–1.16)	0.69
ART Initiation	Facility level	Tertiary	β_6 cos(2πt/4) seasonal	0.0349	0.0912	1.04 (0.87–1.24)	0.70
ART Initiation	Ownership	NGO	β_0 Intercept	0.3502	0.1770	1.42 (1.00–2.01)	0.05
ART Initiation	Ownership	NGO	β_1 Time (per quarter)	0.0457	0.0043	1.05 (1.04–1.06)	<.001
ART Initiation	Ownership	NGO	β_2 Pandemic period	-0.7401	0.2350	0.48 (0.30–0.76)	0.002
ART Initiation	Ownership	NGO	β_3 War period	-1.3249	0.2123	0.27 (0.18–0.40)	<.001
ART Initiation	Ownership	NGO	β_4 Post-war period	-1.4064	0.2249	0.25 (0.16–0.38)	<.001
ART Initiation	Ownership	NGO	β_5 sin(2πt/4) seasonal	0.0279	0.0728	1.03 (0.89–1.19)	0.70
ART Initiation	Ownership	NGO	β_6 cos(2πt/4) seasonal	-0.0713	0.0732	0.93 (0.81–1.07)	0.33
ART Initiation	Ownership	Public	β_0 Intercept	5.1513	0.0731	172.7 (149.6–199.3)	<.001
ART Initiation	Ownership	Public	β_1 Time (per quarter)	-0.0109	0.0021	0.99 (0.99–0.99)	<.001
ART Initiation	Ownership	Public	β_2 Pandemic period	-0.4169	0.1712	0.66 (0.47–0.92)	0.01
ART Initiation	Ownership	Public	β_3 War period	-0.3293	0.1388	0.72 (0.55–0.94)	0.02
ART Initiation	Ownership	Public	β_4 Post-war period	0.0004	0.1448	1.00 (0.75–1.33)	1.00
ART Initiation	Ownership	Public	β_5 sin(2πt/4) seasonal	-0.0340	0.0464	0.97 (0.88–1.06)	0.46
ART Initiation	Ownership	Public	β_6 cos(2πt/4) seasonal	0.0521	0.0461	1.05 (0.96–1.15)	0.26

© 2026 Kebede HK et al. *JAMA Network Open*.

eTable 6. Rate (Binomial GLM) Models: Linkage and ART Initiation Conditional Probabilities

Binomial GLMs (logit link) for conditional cascade probabilities: linkage rate = linked / diagnosed; ART initiation rate = ART starts / linked. Coefficients exponentiated to odds ratios (here reported as IRR for consistency with count models). The "Converged" column flags models where the binomial GLM Hessian was positive-definite and standard errors were finite; one model (ART rate, Primary facility) failed convergence and post-war estimates are unstable. Significance markers as in eTable 5.

Outcome	Stratifier	Stratum	Family	Converged	Pandemic OR (95% CI)	P	War OR (95% CI)	P	Post-war OR (95% CI)	P
Linkage to Care Rate	Overall	all	Bin	Yes	0.82 (0.52-1.29)	0.39	0.65 (0.46-0.90)*	0.010	1.58 (1.01-2.46)*	0.046
Linkage to Care Rate	Sex	Female	Bin	Yes	0.81 (0.43-1.50)	0.50	0.60 (0.38-0.96)*	0.031	0.94 (0.56-1.56)	0.80
Linkage to Care Rate	Sex	Male	Bin	Yes	0.80 (0.41-1.54)	0.50	0.71 (0.44-1.15)	0.17	5.03 (1.79-14.13)**	0.002
Linkage to Care Rate	Age	0-24 years	Bin	Yes	1.82 (0.24-13.76)	0.56	0.68 (0.22-2.06)	0.49	0.33 (0.14-0.78)*	0.011
Linkage to Care Rate	Age	25-34 years	Bin	Yes	0.57 (0.18-1.83)	0.35	1.23 (0.38-4.02)	0.73	1.94 (0.51-7.35)	0.33
Linkage to Care Rate	Age	35-44 years	Bin	Yes	1.17 (0.52-2.63)	0.70	0.58 (0.34-1.01)	0.056	1.83 (0.80-4.20)	0.15
Linkage to Care Rate	Age	45+ years	Bin	Yes	0.90 (0.26-3.07)	0.86	0.79 (0.34-1.82)	0.58	3.76 (0.86-16.54)	0.079
Linkage to Care Rate	Facility level	Primary	Bin	Yes	0.98 (0.34-2.82)	0.98	0.36 (0.18-0.75)**	0.006	1.16 (0.39-3.45)	0.79
Linkage to Care Rate	Facility level	Secondary	Bin	Yes	0.49 (0.24-0.98)*	0.043	0.43 (0.26-0.70)***	<.001	0.78 (0.43-1.43)	0.43
Linkage to Care Rate	Facility level	Tertiary	Bin	Yes	0.57 (0.26-1.24)	0.16	0.74 (0.38-1.46)	0.38	1.77 (0.66-4.77)	0.26
Linkage to Care Rate	Ownership	Public	Bin	Yes	0.75 (0.47-1.19)	0.22	0.62 (0.44-0.87)**	0.006	1.46 (0.93-2.29)	0.10
ART Initiation Rate	Overall	all	Bin	Yes	12.48 (3.97-39.18)***	<.001	12.74 (5.23-31.06)***	<.001	9.27 (4.54-18.95)***	<.001
ART Initiation Rate	Sex	Female	Bin	Yes	0.30 (0.21-0.44)***	<.001	16.02 (3.94-65.09)***	<.001	19.43 (4.77-79.16)***	<.001
ART Initiation Rate	Sex	Male	Bin	Yes	7.66 (1.87-31.42)**	0.005	10.12 (3.18-32.26)***	<.001	5.62 (2.41-13.11)***	<.001
ART Initiation Rate	Facility level	Primary	Bin	No	7.30 (1.78-30.05)**	0.006	4.93 (1.53-15.90)**	0.007	33963571.38 (0.00-Inf)	0.98
ART Initiation Rate	Facility level	Secondary	Bin	Yes	24.99 (3.46-180.21)**	0.001	63.65 (8.87-456.87)***	<.001	38.36 (9.40-156.50)***	<.001
ART Initiation Rate	Ownership	Public	Bin	Yes	13.65 (3.37-55.25)***	<.001	24.07 (5.96-97.30)***	<.001	6.78 (3.30-13.91)***	<.001

© 2026 Kebede HK et al. *JAMA Network Open*.

eTable 6a. Full coefficient table for all 17 rate models

All 7 coefficient terms for every rate (binomial) model. 17 models × 7 terms = 119 rows. Coefficients are on the log-odds scale (β) and exponentiated to odds ratios. Where ORs are extreme (e.g., > 10^6) the binomial GLM failed to converge in that stratum (typically when one or more pre-war quarters contained zero events in the denominator).

Outcome	Stratifier	Stratum	Term	β	SE	OR (95% CI)	P
Linkage to Care Rate	Overall	all	β_0 Intercept	1.5531	0.0484	4.73 (4.30–5.20)	<.001
Linkage to Care Rate	Overall	all	β_1 Time (per quarter)	0.0186	0.0018	1.02 (1.02–1.02)	<.001
Linkage to Care Rate	Overall	all	β_2 Pandemic period	-0.1989	0.2305	0.82 (0.52–1.29)	0.39
Linkage to Care Rate	Overall	all	β_3 War period	-0.4369	0.1705	0.65 (0.46–0.90)	0.01
Linkage to Care Rate	Overall	all	β_4 Post-war period	0.4554	0.2278	1.58 (1.01–2.46)	0.05
Linkage to Care Rate	Overall	all	β_5 sin(2πt/4) seasonal	-0.0154	0.0390	0.98 (0.91–1.06)	0.69
Linkage to Care Rate	Overall	all	β_6 cos(2πt/4) seasonal	0.1352	0.0382	1.14 (1.06–1.23)	<.001
Linkage to Care Rate	Sex	Female	β_0 Intercept	1.4885	0.0649	4.43 (3.90–5.03)	<.001
Linkage to Care Rate	Sex	Female	β_1 Time (per quarter)	0.0208	0.0024	1.02 (1.02–1.03)	<.001
Linkage to Care Rate	Sex	Female	β_2 Pandemic period	-0.2150	0.3167	0.81 (0.43–1.50)	0.50
Linkage to Care Rate	Sex	Female	β_3 War period	-0.5060	0.2351	0.60 (0.38–0.96)	0.03
Linkage to Care Rate	Sex	Female	β_4 Post-war period	-0.0666	0.2616	0.94 (0.56–1.56)	0.80
Linkage to Care Rate	Sex	Female	β_5 sin(2πt/4) seasonal	-0.0272	0.0517	0.97 (0.88–1.08)	0.60
Linkage to Care Rate	Sex	Female	β_6 cos(2πt/4) seasonal	0.1574	0.0498	1.17 (1.06–1.29)	0.002
Linkage to Care Rate	Sex	Male	β_0 Intercept	1.6330	0.0729	5.12 (4.44–5.91)	<.001
Linkage to Care Rate	Sex	Male	β_1 Time (per quarter)	0.0158	0.0027	1.02 (1.01–1.02)	<.001
Linkage to Care Rate	Sex	Male	β_2 Pandemic period	-0.2294	0.3372	0.80 (0.41–1.54)	0.50
Linkage to Care Rate	Sex	Male	β_3 War period	-0.3437	0.2488	0.71 (0.44–1.15)	0.17
Linkage to Care Rate	Sex	Male	β_4 Post-war period	1.6152	0.5271	5.03 (1.79–14.13)	0.002
Linkage to Care Rate	Sex	Male	β_5 sin(2πt/4) seasonal	-0.0018	0.0599	1.00 (0.89–1.12)	0.98
Linkage to Care Rate	Sex	Male	β_6 cos(2πt/4) seasonal	0.1145	0.0594	1.12 (1.00–1.26)	0.05
Linkage to Care Rate	Age	0-24 years	β_0 Intercept	1.1990	0.1221	3.32 (2.61–4.21)	<.001
Linkage to Care Rate	Age	0-24 years	β_1 Time (per quarter)	0.0327	0.0048	1.03 (1.02–1.04)	<.001
Linkage to Care Rate	Age	0-24 years	β_2 Pandemic period	0.5968	1.0332	1.82 (0.24–13.76)	0.56
Linkage to Care Rate	Age	0-24 years	β_3 War period	-0.3858	0.5652	0.68 (0.22–2.06)	0.49
Linkage to Care Rate	Age	0-24 years	β_4 Post-war period	-1.0997	0.4313	0.33 (0.14–0.78)	0.01
Linkage to Care Rate	Age	0-24 years	β_5 sin(2πt/4) seasonal	0.3235	0.0989	1.38 (1.14–1.68)	0.001
Linkage to Care Rate	Age	0-24 years	β_6 cos(2πt/4) seasonal	0.1624	0.0949	1.18 (0.98–1.42)	0.09
Linkage to Care Rate	Age	25-34 years	β_0 Intercept	4.8501	0.4380	127.7 (54.14–301.4)	<.001
Linkage to Care Rate	Age	25-34 years	β_1 Time (per quarter)	-0.0166	0.0111	0.98 (0.96–1.01)	0.13
Linkage to Care Rate	Age	25-34 years	β_2 Pandemic period	-0.5555	0.5925	0.57 (0.18–1.83)	0.35
Linkage to Care Rate	Age	25-34 years	β_3 War period	0.2083	0.6039	1.23 (0.38–4.02)	0.73
Linkage to Care Rate	Age	25-34 years	β_4 Post-war period	0.6647	0.6788	1.94 (0.51–7.35)	0.33
Linkage to Care Rate	Age	25-34 years	β_5 sin(2πt/4) seasonal	-0.4574	0.2252	0.63 (0.41–0.98)	0.04
Linkage to Care Rate	Age	25-34 years	β_6 cos(2πt/4) seasonal	0.1874	0.2203	1.21 (0.78–1.86)	0.40
Linkage to Care Rate	Age	35-44 years	β_0 Intercept	1.6730	0.0860	5.33 (4.50–6.31)	<.001
Linkage to Care Rate	Age	35-44 years	β_1 Time (per quarter)	0.0154	0.0031	1.02 (1.01–1.02)	<.001
Linkage to Care Rate	Age	35-44 years	β_2 Pandemic period	0.1586	0.4125	1.17 (0.52–2.63)	0.70
Linkage to Care Rate	Age	35-44 years	β_3 War period	-0.5375	0.2811	0.58 (0.34–1.01)	0.06
Linkage to Care Rate	Age	35-44 years	β_4 Post-war period	0.6035	0.4240	1.83 (0.80–4.20)	0.15
Linkage to Care Rate	Age	35-44 years	β_5 sin(2πt/4) seasonal	0.0329	0.0689	1.03 (0.90–1.18)	0.63
Linkage to Care Rate	Age	35-44 years	β_6 cos(2πt/4) seasonal	0.0510	0.0690	1.05 (0.92–1.20)	0.46

© 2026 Kebede HK et al. *JAMA Network Open*.

Outcome	Stratifier	Stratum	Term	β	SE	OR (95% CI)	P
Linkage to Care Rate	Age	45+ years	β_0 Intercept	1.5437	0.1159	4.68 (3.73–5.88)	<.001
Linkage to Care Rate	Age	45+ years	β_1 Time (per quarter)	0.0189	0.0044	1.02 (1.01–1.03)	<.001
Linkage to Care Rate	Age	45+ years	β_2 Pandemic period	-0.1082	0.6278	0.90 (0.26–3.07)	0.86
Linkage to Care Rate	Age	45+ years	β_3 War period	-0.2327	0.4249	0.79 (0.34–1.82)	0.58
Linkage to Care Rate	Age	45+ years	β_4 Post-war period	1.3256	0.7552	3.76 (0.86–16.54)	0.08
Linkage to Care Rate	Age	45+ years	β_5 sin(2πt/4) seasonal	0.0534	0.0943	1.05 (0.88–1.27)	0.57
Linkage to Care Rate	Age	45+ years	β_6 cos(2πt/4) seasonal	0.1660	0.0932	1.18 (0.98–1.42)	0.07
Linkage to Care Rate	Facility level	Primary	β_0 Intercept	1.0681	0.1296	2.91 (2.26–3.75)	<.001
Linkage to Care Rate	Facility level	Primary	β_1 Time (per quarter)	0.0375	0.0044	1.04 (1.03–1.05)	<.001
Linkage to Care Rate	Facility level	Primary	β_2 Pandemic period	-0.0168	0.5367	0.98 (0.34–2.82)	0.98
Linkage to Care Rate	Facility level	Primary	β_3 War period	-1.0086	0.3683	0.36 (0.18–0.75)	0.006
Linkage to Care Rate	Facility level	Primary	β_4 Post-war period	0.1479	0.5562	1.16 (0.39–3.45)	0.79
Linkage to Care Rate	Facility level	Primary	β_5 sin(2πt/4) seasonal	0.1302	0.0805	1.14 (0.97–1.33)	0.11
Linkage to Care Rate	Facility level	Primary	β_6 cos(2πt/4) seasonal	0.0365	0.0833	1.04 (0.88–1.22)	0.66
Linkage to Care Rate	Facility level	Secondary	β_0 Intercept	1.6407	0.0582	5.16 (4.60–5.78)	<.001
Linkage to Care Rate	Facility level	Secondary	β_1 Time (per quarter)	0.0236	0.0028	1.02 (1.02–1.03)	<.001
Linkage to Care Rate	Facility level	Secondary	β_2 Pandemic period	-0.7194	0.3562	0.49 (0.24–0.98)	0.04
Linkage to Care Rate	Facility level	Secondary	β_3 War period	-0.8513	0.2501	0.43 (0.26–0.70)	<.001
Linkage to Care Rate	Facility level	Secondary	β_4 Post-war period	-0.2451	0.3076	0.78 (0.43–1.43)	0.43
Linkage to Care Rate	Facility level	Secondary	β_5 sin(2πt/4) seasonal	-0.0582	0.0529	0.94 (0.85–1.05)	0.27
Linkage to Care Rate	Facility level	Secondary	β_6 cos(2πt/4) seasonal	0.0075	0.0510	1.01 (0.91–1.11)	0.88
Linkage to Care Rate	Facility level	Tertiary	β_0 Intercept	0.6525	0.1670	1.92 (1.38–2.66)	<.001
Linkage to Care Rate	Facility level	Tertiary	β_1 Time (per quarter)	0.0281	0.0047	1.03 (1.02–1.04)	<.001
Linkage to Care Rate	Facility level	Tertiary	β_2 Pandemic period	-0.5611	0.3952	0.57 (0.26–1.24)	0.16
Linkage to Care Rate	Facility level	Tertiary	β_3 War period	-0.3014	0.3455	0.74 (0.38–1.46)	0.38
Linkage to Care Rate	Facility level	Tertiary	β_4 Post-war period	0.5705	0.5061	1.77 (0.66–4.77)	0.26
Linkage to Care Rate	Facility level	Tertiary	β_5 sin(2πt/4) seasonal	-0.1227	0.0871	0.88 (0.75–1.05)	0.16
Linkage to Care Rate	Facility level	Tertiary	β_6 cos(2πt/4) seasonal	0.5625	0.0859	1.76 (1.48–2.08)	<.001
Linkage to Care Rate	Ownership	Public	β_0 Intercept	1.5744	0.0491	4.83 (4.38–5.32)	<.001
Linkage to Care Rate	Ownership	Public	β_1 Time (per quarter)	0.0174	0.0019	1.02 (1.01–1.02)	<.001
Linkage to Care Rate	Ownership	Public	β_2 Pandemic period	-0.2927	0.2374	0.75 (0.47–1.19)	0.22
Linkage to Care Rate	Ownership	Public	β_3 War period	-0.4804	0.1756	0.62 (0.44–0.87)	0.006
Linkage to Care Rate	Ownership	Public	β_4 Post-war period	0.3769	0.2302	1.46 (0.93–2.29)	0.10
Linkage to Care Rate	Ownership	Public	β_5 sin(2πt/4) seasonal	-0.0214	0.0399	0.98 (0.91–1.06)	0.59
Linkage to Care Rate	Ownership	Public	β_6 cos(2πt/4) seasonal	0.1334	0.0392	1.14 (1.06–1.23)	<.001
ART Initiation Rate	Overall	all	β_0 Intercept	0.5804	0.0407	1.79 (1.65–1.94)	<.001
ART Initiation Rate	Overall	all	β_1 Time (per quarter)	0.0230	0.0015	1.02 (1.02–1.03)	<.001
ART Initiation Rate	Overall	all	β_2 Pandemic period	2.5239	0.5838	12.48 (3.97–39.18)	<.001
ART Initiation Rate	Overall	all	β_3 War period	2.5450	0.4546	12.74 (5.23–31.06)	<.001
ART Initiation Rate	Overall	all	β_4 Post-war period	2.2267	0.3647	9.27 (4.54–18.95)	<.001
ART Initiation Rate	Overall	all	β_5 sin(2πt/4) seasonal	-0.0376	0.0332	0.96 (0.90–1.03)	0.26
ART Initiation Rate	Overall	all	β_6 cos(2πt/4) seasonal	-0.0307	0.0318	0.97 (0.91–1.03)	0.33
ART Initiation Rate	Sex	Female	β_0 Intercept	0.3810	0.0540	1.46 (1.32–1.63)	<.001
ART Initiation Rate	Sex	Female	β_1 Time (per quarter)	0.0263	0.0019	1.03 (1.02–1.03)	<.001
ART Initiation Rate	Sex	Female	β_2 Pandemic period	-1.1884	0.1890	0.30 (0.21–0.44)	<.001
ART Initiation Rate	Sex	Female	β_3 War period	2.7740	0.7152	16.02 (3.94–65.09)	<.001
ART Initiation Rate	Sex	Female	β_4 Post-war period	2.9670	0.7166	19.43 (4.77–79.16)	<.001

© 2026 Kebede HK et al. *JAMA Network Open*.

Outcome	Stratifier	Stratum	Term	β	SE	OR (95% CI)	P
ART Initiation Rate	Sex	Female	β_5 sin(2πt/4) seasonal	-0.0368	0.0426	0.96 (0.89–1.05)	0.39
ART Initiation Rate	Sex	Female	β_6 cos(2πt/4) seasonal	-0.0913	0.0401	0.91 (0.84–0.99)	0.02
ART Initiation Rate	Sex	Male	β_0 Intercept	0.8323	0.0629	2.30 (2.03–2.60)	<.001
ART Initiation Rate	Sex	Male	β_1 Time (per quarter)	0.0194	0.0023	1.02 (1.02–1.02)	<.001
ART Initiation Rate	Sex	Male	β_2 Pandemic period	2.0360	0.7201	7.66 (1.87–31.42)	0.005
ART Initiation Rate	Sex	Male	β_3 War period	2.3148	0.5913	10.12 (3.18–32.26)	<.001
ART Initiation Rate	Sex	Male	β_4 Post-war period	1.7257	0.4324	5.62 (2.41–13.11)	<.001
ART Initiation Rate	Sex	Male	β_5 sin(2πt/4) seasonal	-0.0412	0.0524	0.96 (0.87–1.06)	0.43
ART Initiation Rate	Sex	Male	β_6 cos(2πt/4) seasonal	-0.0169	0.0511	0.98 (0.89–1.09)	0.74
ART Initiation Rate	Facility level	Primary	β_0 Intercept	-0.2957	0.1008	0.74 (0.61–0.91)	0.003
ART Initiation Rate	Facility level	Primary	β_1 Time (per quarter)	0.0379	0.0031	1.04 (1.03–1.04)	<.001
ART Initiation Rate	Facility level	Primary	β_2 Pandemic period	1.9885	0.7217	7.30 (1.78–30.05)	0.006
ART Initiation Rate	Facility level	Primary	β_3 War period	1.5962	0.5968	4.93 (1.53–15.90)	0.007
ART Initiation Rate	Facility level	Primary	β_4 Post-war period	17.3408	842.5302	3.4e+07 (0.00–—)	0.98
ART Initiation Rate	Facility level	Primary	β_5 sin(2πt/4) seasonal	0.0859	0.0602	1.09 (0.97–1.23)	0.15
ART Initiation Rate	Facility level	Primary	β_6 cos(2πt/4) seasonal	-0.0441	0.0611	0.96 (0.85–1.08)	0.47
ART Initiation Rate	Facility level	Secondary	β_0 Intercept	0.7396	0.0459	2.10 (1.91–2.29)	<.001
ART Initiation Rate	Facility level	Secondary	β_1 Time (per quarter)	0.0118	0.0018	1.01 (1.01–1.02)	<.001
ART Initiation Rate	Facility level	Secondary	β_2 Pandemic period	3.2183	1.0081	24.99 (3.46–180.2)	0.001
ART Initiation Rate	Facility level	Secondary	β_3 War period	4.1534	1.0056	63.65 (8.87–456.9)	<.001
ART Initiation Rate	Facility level	Secondary	β_4 Post-war period	3.6471	0.7173	38.36 (9.40–156.5)	<.001
ART Initiation Rate	Facility level	Secondary	β_5 sin(2πt/4) seasonal	-0.0672	0.0413	0.93 (0.86–1.01)	0.10
ART Initiation Rate	Facility level	Secondary	β_6 cos(2πt/4) seasonal	-0.0279	0.0393	0.97 (0.90–1.05)	0.48
ART Initiation Rate	Ownership	Public	β_0 Intercept	0.5875	0.0417	1.80 (1.66–1.95)	<.001
ART Initiation Rate	Ownership	Public	β_1 Time (per quarter)	0.0251	0.0016	1.03 (1.02–1.03)	<.001
ART Initiation Rate	Ownership	Public	β_2 Pandemic period	2.6136	0.7134	13.65 (3.37–55.25)	<.001
ART Initiation Rate	Ownership	Public	β_3 War period	3.1810	0.7126	24.07 (5.96–97.30)	<.001
ART Initiation Rate	Ownership	Public	β_4 Post-war period	1.9136	0.3667	6.78 (3.30–13.91)	<.001
ART Initiation Rate	Ownership	Public	β_5 sin(2πt/4) seasonal	-0.0793	0.0347	0.92 (0.86–0.99)	0.02
ART Initiation Rate	Ownership	Public	β_6 cos(2πt/4) seasonal	-0.0321	0.0332	0.97 (0.91–1.03)	0.33

© 2026 Kebede HK et al. *JAMA Network Open*.

eTable 7. ARIMA Models With Intervention Regressors

Outcome	ARIMA order	AICc	Pandemic coef	P	War coef	P	Post-war coef	P
HIV Diagnoses	(0,1,0)(1,0,1)[4]	791.4	10.87	0.72	59.19	0.20	86.92	0.14
Linkage to Care	(0,1,0)(1,0,1)[4]	792.3	15.90	0.60	66.74	0.16	100.85	0.09
ART Initiation	(1,1,1)(0,0,1)[4]	785.7	-31.74	0.26	-27.71	0.48	-11.46	0.79

ARIMA(p,d,q)(P,D,Q)[4] notation; coefficients are on the count scale (not log). All ARIMA intervention coefficients had the same direction as the negative binomial segmented regression estimates.

© 2026 Kebede HK et al. *JAMA Network Open*.

eTable 8. Sensitivity Analysis 1: Model Without Seasonal Harmonics

Sin/cos seasonal terms removed. Pandemic and war IRRs change by <2% from the main analysis (compare with eTable 5).

Outcome	Stratifier	Stratum	Type	Fam	AIC	Pandemic IRR (95% CI)	P	War IRR (95% CI)	P	Post-war IRR (95% CI)	P
HIV Diagnoses	Overall	all	count	NB	805.4	0.65 (0.51-0.83)***	<.001	0.70 (0.57-0.86)***	<.001	0.97 (0.79-1.20)	0.81
HIV Diagnoses	Sex	Female	count	NB	730.1	0.59 (0.45-0.78)***	<.001	0.57 (0.45-0.72)***	<.001	0.85 (0.67-1.07)	0.16
HIV Diagnoses	Sex	Male	count	NB	679.6	0.73 (0.56-0.97)*	0.029	0.91 (0.73-1.14)	0.43	1.18 (0.94-1.48)	0.16
HIV Diagnoses	Age	0-24 years	count	NB	573.6	0.58 (0.39-0.87)**	0.008	0.59 (0.43-0.81)**	0.001	0.98 (0.71-1.34)	0.88
HIV Diagnoses	Age	25-34 years	count	NB	493.9	0.78 (0.63-0.96)*	0.018	0.92 (0.78-1.07)	0.28	0.93 (0.79-1.10)	0.38
HIV Diagnoses	Age	35-44 years	count	NB	626.1	0.83 (0.64-1.09)	0.18	0.75 (0.60-0.94)*	0.012	0.91 (0.73-1.14)	0.42
HIV Diagnoses	Age	45+ years	count	NB	561.6	0.59 (0.41-0.86)**	0.006	0.82 (0.62-1.09)	0.17	1.17 (0.89-1.55)	0.25
HIV Diagnoses	Facility level	Primary	count	NB	740.3	0.48 (0.25-0.90)*	0.023	0.29 (0.17-0.50)***	<.001	0.39 (0.22-0.70)**	0.002
HIV Diagnoses	Facility level	Secondary	count	NB	748.3	0.86 (0.60-1.22)	0.39	1.42 (1.08-1.88)*	0.013	2.14 (1.60-2.85)***	<.001
HIV Diagnoses	Facility level	Tertiary	count	NB	652.5	0.22 (0.12-0.43)***	<.001	0.19 (0.11-0.33)***	<.001	0.16 (0.09-0.29)***	<.001
HIV Diagnoses	Ownership	NGO	count	NB	519.8	0.35 (0.19-0.65)***	<.001	0.21 (0.13-0.36)***	<.001	0.20 (0.11-0.34)***	<.001
HIV Diagnoses	Ownership	Public	count	NB	800.0	0.64 (0.49-0.84)***	<.001	0.74 (0.60-0.91)**	0.005	1.04 (0.84-1.30)	0.71
Linkage to Care	Overall	all	count	NB	820.4	0.63 (0.47-0.85)**	0.003	0.67 (0.53-0.86)**	0.001	0.97 (0.75-1.26)	0.84
Linkage to Care Rate	Overall	all	rate	Bin	703.0	0.82 (0.52-1.29)	0.39	0.65 (0.46-0.90)*	0.010	1.58 (1.01-2.46)*	0.046
Linkage to Care	Sex	Female	count	NB	746.8	0.58 (0.42-0.82)**	0.002	0.54 (0.41-0.72)***	<.001	0.83 (0.62-1.10)	0.19
Linkage to Care Rate	Sex	Female	rate	Bin	544.3	0.81 (0.43-1.50)	0.50	0.60 (0.38-0.96)*	0.031	0.94 (0.56-1.56)	0.80
Linkage to Care	Sex	Male	count	NB	685.7	0.72 (0.52-0.98)*	0.039	0.88 (0.68-1.14)	0.33	1.21 (0.93-1.57)	0.16
Linkage to Care Rate	Sex	Male	rate	Bin	473.5	0.80 (0.41-1.54)	0.50	0.71 (0.44-1.15)	0.17	5.03 (1.79-14.13)**	0.002
Linkage to Care	Age	0-24 years	count	NB	567.3	0.53 (0.35-0.80)**	0.002	0.50 (0.36-0.70)***	<.001	0.79 (0.57-1.09)	0.15
Linkage to Care Rate	Age	0-24 years	rate	Bin	385.1	1.82 (0.24-13.76)	0.56	0.68 (0.22-2.06)	0.49	0.33 (0.14-0.78)*	0.011
Linkage to Care	Age	25-34 years	count	NB	492.3	0.76 (0.62-0.94)*	0.011	0.92 (0.78-1.08)	0.30	0.94 (0.79-1.11)	0.45
Linkage to Care Rate	Age	25-34 years	rate	Bin	181.0	0.57 (0.18-1.83)	0.35	1.23 (0.38-4.02)	0.73	1.94 (0.51-7.35)	0.33
Linkage to Care	Age	35-44 years	count	NB	636.2	0.87 (0.64-1.18)	0.36	0.72 (0.55-0.93)*	0.011	0.93 (0.71-1.20)	0.56
Linkage to Care Rate	Age	35-44 years	rate	Bin	440.1	1.17 (0.52-2.63)	0.70	0.58 (0.34-1.01)	0.056	1.83 (0.80-4.20)	0.15
Linkage to Care	Age	45+ years	count	NB	569.8	0.58 (0.38-0.89)*	0.012	0.79 (0.58-1.09)	0.16	1.18 (0.86-1.63)	0.31
Linkage to Care Rate	Age	45+ years	rate	Bin	357.6	0.90 (0.26-3.07)	0.86	0.79 (0.34-1.82)	0.58	3.76 (0.86-16.54)	0.079
Linkage to Care	Facility level	Primary	count	NB	738.5	0.46 (0.23-0.94)*	0.033	0.26 (0.14-0.49)***	<.001	0.36 (0.19-0.70)**	0.003
Linkage to Care Rate	Facility level	Primary	rate	Bin	355.0	0.98 (0.34-2.82)	0.98	0.36 (0.18-0.75)**	0.006	1.16 (0.39-3.45)	0.79
Linkage to Care	Facility level	Secondary	count	NB	747.4	0.83 (0.56-1.23)	0.35	1.37 (1.01-1.86)*	0.045	2.13 (1.55-2.94)***	<.001
Linkage to Care Rate	Facility level	Secondary	rate	Bin	633.3	0.49 (0.24-0.98)*	0.043	0.43 (0.26-0.70)***	<.001	0.78 (0.43-1.43)	0.43
Linkage to Care	Facility level	Tertiary	count	NB	628.8	0.21 (0.10-0.41)***	<.001	0.17 (0.10-0.31)***	<.001	0.15 (0.08-0.29)***	<.001
Linkage to Care Rate	Facility level	Tertiary	rate	Bin	362.2	0.57 (0.26-1.24)	0.16	0.74 (0.38-1.46)	0.38	1.77 (0.66-4.77)	0.26

© 2026 Kebede HK et al. *JAMA Network Open*.

Outcome	Stratifier	Stratum	Type	Fam	AIC	Pandemic IRR (95% CI)	P	War IRR (95% CI)	P	Post-war IRR (95% CI)	P
Linkage to Care	Ownership	NGO	count	NB	513.9	0.33 (0.17-0.64)***	<.001	0.19 (0.11-0.34)***	<.001	0.17 (0.09-0.32)***	<.001
Linkage to Care	Ownership	Public	count	NB	813.7	0.62 (0.45-0.86)**	0.004	0.70 (0.54-0.91)**	0.008	1.04 (0.80-1.37)	0.75
Linkage to Care Rate	Ownership	Public	rate	Bin	710.9	0.75 (0.47-1.19)	0.22	0.62 (0.44-0.87)**	0.006	1.46 (0.93-2.29)	0.10
ART Initiation	Overall	all	count	NB	803.9	0.68 (0.49-0.94)*	0.018	0.69 (0.53-0.90)**	0.007	0.95 (0.72-1.26)	0.74
ART Initiation Rate	Overall	all	rate	Bin	1.2e+03	12.48 (3.97-39.18)***	<.001	12.74 (5.23-31.06)***	<.001	9.27 (4.54-18.95)***	<.001
ART Initiation	Sex	Female	count	NB	720.7	0.62 (0.44-0.88)**	0.008	0.56 (0.42-0.75)***	<.001	0.81 (0.60-1.08)	0.15
ART Initiation Rate	Sex	Female	rate	Bin	961.7	0.30 (0.21-0.44)***	<.001	16.02 (3.94-65.09)***	<.001	19.43 (4.77-79.16)***	<.001
ART Initiation	Sex	Male	count	NB	678.7	0.76 (0.53-1.08)	0.13	0.91 (0.69-1.21)	0.53	1.20 (0.89-1.61)	0.23
ART Initiation Rate	Sex	Male	rate	Bin	572.8	7.66 (1.87-31.42)**	0.005	10.12 (3.18-32.26)***	<.001	5.62 (2.41-13.11)***	<.001
ART Initiation	Age	0-24 years	count	NB	544.8	0.50 (0.33-0.76)**	0.001	0.45 (0.32-0.64)***	<.001	0.66 (0.47-0.92)*	0.014
ART Initiation	Age	25-34 years	count	NB	521.4	0.71 (0.57-0.87)***	<.001	0.85 (0.73-1.00)*	0.043	0.88 (0.74-1.03)	0.11
ART Initiation	Age	35-44 years	count	NB	616.0	0.89 (0.66-1.21)	0.46	0.71 (0.55-0.92)**	0.010	0.87 (0.67-1.14)	0.31
ART Initiation	Age	45+ years	count	NB	556.6	0.62 (0.40-0.96)*	0.032	0.81 (0.58-1.14)	0.23	1.16 (0.82-1.63)	0.41
ART Initiation	Facility level	Primary	count	NB	681.6	0.52 (0.28-0.96)*	0.037	0.28 (0.17-0.48)***	<.001	0.37 (0.21-0.65)***	<.001
ART Initiation Rate	Facility level	Primary	rate	Bin	500.0	7.30 (1.78-30.05)**	0.006	4.93 (1.53-15.90)**	0.007	33963571.38 (0.00-Inf)	0.98
ART Initiation	Facility level	Secondary	count	NB	741.7	0.90 (0.57-1.42)	0.66	1.45 (1.01-2.08)*	0.045	2.14 (1.47-3.12)***	<.001
ART Initiation Rate	Facility level	Secondary	rate	Bin	962.2	24.99 (3.46-180.21)**	0.001	63.65 (8.87-456.87)***	<.001	38.36 (9.40-156.50)***	<.001
ART Initiation	Facility level	Tertiary	count	NB	627.0	0.21 (0.11-0.43)***	<.001	0.18 (0.10-0.32)***	<.001	0.15 (0.08-0.29)***	<.001
ART Initiation	Ownership	NGO	count	NB	427.0	0.48 (0.30-0.77)**	0.002	0.27 (0.17-0.41)***	<.001	0.25 (0.16-0.38)***	<.001
ART Initiation	Ownership	Public	count	NB	795.3	0.65 (0.46-0.91)*	0.013	0.71 (0.54-0.94)*	0.016	0.99 (0.74-1.32)	0.95
ART Initiation Rate	Ownership	Public	rate	Bin	1.1e+03	13.65 (3.37-55.25)***	<.001	24.07 (5.96-97.30)***	<.001	6.78 (3.30-13.91)***	<.001

© 2026 Kebede HK et al. *JAMA Network Open*.

eTable 9. Sensitivity Analysis 2: Full Slope-Change Model

Added time-after-war and time-after-postwar slope-change terms. Level-change war IRRs become non-significant in many strata but slope-change terms are also non-significant; the level-change-only specification (eTable 5) captures the dominant signal.

Outcome	Stratifier	Stratum	Type	Fam	AIC	Pandemic IRR (95% CI)	P	War IRR (95% CI)	P	Post-war IRR (95% CI)	P
HIV Diagnoses	Overall	all	count	NB	801.5	0.65 (0.52-0.83)***	<.001	0.96 (0.68-1.35)	0.81	1.21 (0.65-2.27)	0.55
HIV Diagnoses	Sex	Female	count	NB	725.9	0.59 (0.46-0.77)***	<.001	0.89 (0.60-1.31)	0.55	1.45 (0.71-2.99)	0.31
HIV Diagnoses	Sex	Male	count	NB	676.8	0.75 (0.57-0.97)*	0.030	1.09 (0.74-1.60)	0.66	1.07 (0.53-2.15)	0.86
HIV Diagnoses	Age	0-24 years	count	NB	574.0	0.60 (0.41-0.87)**	0.008	0.66 (0.37-1.18)	0.16	0.71 (0.25-2.02)	0.52
HIV Diagnoses	Age	25-34 years	count	NB	500.3	0.78 (0.63-0.96)*	0.017	0.91 (0.68-1.20)	0.49	1.01 (0.61-1.67)	0.96
HIV Diagnoses	Age	35-44 years	count	NB	625.7	0.84 (0.65-1.08)	0.18	1.03 (0.70-1.51)	0.89	1.19 (0.57-2.46)	0.65
HIV Diagnoses	Age	45+ years	count	NB	556.4	0.60 (0.42-0.86)**	0.005	1.31 (0.81-2.11)	0.27	1.92 (0.77-4.77)	0.16
HIV Diagnoses	Facility level	Primary	count	NB	743.2	0.49 (0.26-0.91)*	0.023	0.54 (0.21-1.38)	0.20	0.70 (0.13-3.90)	0.68
HIV Diagnoses	Facility level	Secondary	count	NB	751.5	0.87 (0.61-1.23)	0.42	1.42 (0.87-2.33)	0.16	1.65 (0.68-4.01)	0.27
HIV Diagnoses	Facility level	Tertiary	count	NB	653.5	0.22 (0.12-0.42)***	<.001	0.49 (0.18-1.29)	0.15	0.99 (0.16-5.90)	0.99
HIV Diagnoses	Ownership	NGO	count	NB	524.6	0.37 (0.20-0.66)***	<.001	0.30 (0.12-0.73)**	0.008	0.34 (0.07-1.67)	0.18
HIV Diagnoses	Ownership	Public	count	NB	796.4	0.65 (0.51-0.83)***	<.001	1.03 (0.71-1.49)	0.88	1.33 (0.68-2.60)	0.41
Linkage to Care	Overall	all	count	NB	817.5	0.64 (0.48-0.85)**	0.002	1.00 (0.65-1.52)	0.99	1.37 (0.64-2.95)	0.42
Linkage to Care Rate	Overall	all	rate	Bin	703.0	0.82 (0.52-1.29)	0.39	0.65 (0.46-0.90)*	0.010	1.58 (1.01-2.46)*	0.046
Linkage to Care	Sex	Female	count	NB	743.5	0.58 (0.42-0.80)***	<.001	0.90 (0.56-1.46)	0.68	1.51 (0.63-3.65)	0.36
Linkage to Care Rate	Sex	Female	rate	Bin	544.3	0.81 (0.43-1.50)	0.50	0.60 (0.38-0.96)*	0.031	0.94 (0.56-1.56)	0.80
Linkage to Care	Sex	Male	count	NB	684.1	0.73 (0.54-0.99)*	0.041	1.16 (0.74-1.81)	0.51	1.33 (0.59-2.98)	0.49
Linkage to Care Rate	Sex	Male	rate	Bin	473.5	0.80 (0.41-1.54)	0.50	0.71 (0.44-1.15)	0.17	5.03 (1.79-14.13)**	0.002
Linkage to Care	Age	0-24 years	count	NB	569.4	0.54 (0.36-0.80)**	0.002	0.60 (0.32-1.10)	0.097	0.62 (0.20-1.87)	0.39
Linkage to Care Rate	Age	0-24 years	rate	Bin	385.1	1.82 (0.24-13.76)	0.56	0.68 (0.22-2.06)	0.49	0.33 (0.14-0.78)*	0.011
Linkage to Care	Age	25-34 years	count	NB	499.7	0.76 (0.62-0.94)*	0.010	0.94 (0.71-1.26)	0.70	1.06 (0.64-1.76)	0.81
Linkage to Care Rate	Age	25-34 years	rate	Bin	181.0	0.57 (0.18-1.83)	0.35	1.23 (0.38-4.02)	0.73	1.94 (0.51-7.35)	0.33
Linkage to Care	Age	35-44 years	count	NB	636.5	0.88 (0.65-1.17)	0.37	1.05 (0.67-1.64)	0.83	1.36 (0.59-3.14)	0.48
Linkage to Care Rate	Age	35-44 years	rate	Bin	440.1	1.17 (0.52-2.63)	0.70	0.58 (0.34-1.01)	0.056	1.83 (0.80-4.20)	0.15
Linkage to Care	Age	45+ years	count	NB	566.9	0.59 (0.39-0.88)*	0.011	1.31 (0.75-2.27)	0.34	2.05 (0.72-5.82)	0.18
Linkage to Care Rate	Age	45+ years	rate	Bin	357.6	0.90 (0.26-3.07)	0.86	0.79 (0.34-1.82)	0.58	3.76 (0.86-16.54)	0.079
Linkage to Care	Facility level	Primary	count	NB	741.4	0.47 (0.24-0.94)*	0.032	0.56 (0.19-1.63)	0.29	0.85 (0.12-5.85)	0.87
Linkage to Care Rate	Facility level	Primary	rate	Bin	355.0	0.98 (0.34-2.82)	0.98	0.36 (0.18-0.75)**	0.006	1.16 (0.39-3.45)	0.79
Linkage to Care	Facility level	Secondary	count	NB	751.6	0.84 (0.57-1.23)	0.37	1.46 (0.85-2.53)	0.17	1.80 (0.67-4.83)	0.24
Linkage to Care Rate	Facility level	Secondary	rate	Bin	633.3	0.49 (0.24-0.98)*	0.043	0.43 (0.26-0.70)***	<.001	0.78 (0.43-1.43)	0.43
Linkage to Care	Facility level	Tertiary	count	NB	629.5	0.21 (0.11-0.41)***	<.001	0.51 (0.18-1.43)	0.20	1.16 (0.18-7.59)	0.88

© 2026 Kebede HK et al. *JAMA Network Open*.

Outcome	Stratifier	Stratum	Type	Fam	AIC	Pandemic IRR (95% CI)	P	War IRR (95% CI)	P	Post-war IRR (95% CI)	P
Linkage to Care Rate	Facility level	Tertiary	rate	Bin	362.2	0.57 (0.26-1.24)	0.16	0.74 (0.38-1.46)	0.38	1.77 (0.66-4.77)	0.26
Linkage to Care	Ownership	NGO	count	NB	519.6	0.34 (0.18-0.65)**	0.001	0.28 (0.10-0.75)*	0.011	0.34 (0.06-1.89)	0.22
Linkage to Care	Ownership	Public	count	NB	810.8	0.63 (0.47-0.85)**	0.003	1.08 (0.69-1.69)	0.75	1.52 (0.67-3.45)	0.32
Linkage to Care Rate	Ownership	Public	rate	Bin	710.9	0.75 (0.47-1.19)	0.22	0.62 (0.44-0.87)**	0.006	1.46 (0.93-2.29)	0.10
ART Initiation	Overall	all	count	NB	802.7	0.69 (0.50-0.93)*	0.017	1.07 (0.67-1.70)	0.78	1.48 (0.64-3.43)	0.37
ART Initiation Rate	Overall	all	rate	Bin	1.2e+03	12.48 (3.97-39.18)***	<.001	12.74 (5.23-31.06)***	<.001	9.27 (4.54-18.95)***	<.001
ART Initiation	Sex	Female	count	NB	719.4	0.63 (0.45-0.88)**	0.006	0.96 (0.58-1.57)	0.86	1.60 (0.64-4.02)	0.32
ART Initiation Rate	Sex	Female	rate	Bin	961.7	0.30 (0.21-0.44)***	<.001	16.02 (3.94-65.09)***	<.001	19.43 (4.77-79.16)***	<.001
ART Initiation	Sex	Male	count	NB	679.1	0.77 (0.55-1.09)	0.14	1.24 (0.75-2.06)	0.40	1.40 (0.56-3.50)	0.47
ART Initiation Rate	Sex	Male	rate	Bin	572.8	7.66 (1.87-31.42)**	0.005	10.12 (3.18-32.26)***	<.001	5.62 (2.41-13.11)***	<.001
ART Initiation	Age	0-24 years	count	NB	547.0	0.51 (0.34-0.77)**	0.001	0.56 (0.30-1.03)	0.064	0.58 (0.19-1.77)	0.34
ART Initiation	Age	25-34 years	count	NB	528.3	0.70 (0.57-0.86)***	<.001	0.85 (0.64-1.13)	0.27	0.94 (0.57-1.56)	0.82
ART Initiation	Age	35-44 years	count	NB	615.6	0.90 (0.67-1.21)	0.48	1.08 (0.69-1.68)	0.75	1.40 (0.61-3.24)	0.43
ART Initiation	Age	45+ years	count	NB	555.4	0.62 (0.41-0.95)*	0.029	1.37 (0.76-2.46)	0.29	2.14 (0.72-6.42)	0.17
ART Initiation	Facility level	Primary	count	NB	682.8	0.52 (0.29-0.95)*	0.032	0.65 (0.26-1.64)	0.36	1.06 (0.19-5.82)	0.95
ART Initiation Rate	Facility level	Primary	rate	Bin	500.0	7.30 (1.78-30.05)**	0.006	4.93 (1.53-15.90)**	0.007	33963571.38 (0.00-Inf)	0.98
ART Initiation	Facility level	Secondary	count	NB	747.0	0.91 (0.58-1.43)	0.69	1.60 (0.83-3.06)	0.16	1.96 (0.61-6.32)	0.26
ART Initiation Rate	Facility level	Secondary	rate	Bin	962.2	24.99 (3.46-180.21)**	0.001	63.65 (8.87-456.87)***	<.001	38.36 (9.40-156.50)***	<.001
ART Initiation	Facility level	Tertiary	count	NB	628.1	0.22 (0.11-0.42)***	<.001	0.51 (0.18-1.45)	0.21	1.12 (0.17-7.47)	0.91
ART Initiation	Ownership	NGO	count	NB	432.0	0.48 (0.30-0.75)**	0.001	0.40 (0.20-0.81)*	0.011	0.49 (0.14-1.72)	0.26
ART Initiation	Ownership	Public	count	NB	793.5	0.66 (0.48-0.91)*	0.012	1.12 (0.69-1.81)	0.65	1.56 (0.65-3.75)	0.32
ART Initiation Rate	Ownership	Public	rate	Bin	1.1e+03	13.65 (3.37-55.25)***	<.001	24.07 (5.96-97.30)***	<.001	6.78 (3.30-13.91)***	<.001

© 2026 Kebede HK et al. *JAMA Network Open*.

eTable 10. Sensitivity Analysis 3: Alternative War Onset (Q4 2020)

Q4 2020 reassigned from pandemic to war (war = 9 quarters, pandemic = 3 quarters). War IRRs slightly more negative than the main analysis.

Outcome	Stratifier	Stratum	Type	Fam	AIC	Pandemic IRR (95% CI)	P	War IRR (95% CI)	P	Post-war IRR (95% CI)	P
HIV Diagnoses	Overall	all	count	NB	807.5	0.72 (0.55-0.94)*	0.017	0.67 (0.56-0.82)***	<.001	0.98 (0.80-1.20)	0.83
HIV Diagnoses	Sex	Female	count	NB	729.8	0.48 (0.35-0.66)***	<.001	0.62 (0.50-0.76)***	<.001	0.86 (0.69-1.08)	0.19
HIV Diagnoses	Sex	Male	count	NB	681.7	0.80 (0.59-1.09)	0.16	0.88 (0.71-1.08)	0.22	1.18 (0.94-1.48)	0.14
HIV Diagnoses	Age	0-24 years	count	NB	574.1	0.71 (0.46-1.08)	0.11	0.55 (0.40-0.75)***	<.001	0.98 (0.72-1.33)	0.90
HIV Diagnoses	Age	25-34 years	count	NB	497.8	0.77 (0.61-0.98)*	0.030	0.90 (0.77-1.05)	0.18	0.92 (0.78-1.09)	0.34
HIV Diagnoses	Age	35-44 years	count	NB	626.5	0.93 (0.70-1.24)	0.63	0.73 (0.59-0.90)**	0.003	0.92 (0.73-1.14)	0.44
HIV Diagnoses	Age	45+ years	count	NB	560.8	0.57 (0.37-0.87)**	0.009	0.81 (0.63-1.06)	0.12	1.19 (0.91-1.57)	0.20
HIV Diagnoses	Facility level	Primary	count	NB	742.4	0.57 (0.28-1.15)	0.12	0.28 (0.17-0.48)***	<.001	0.39 (0.22-0.70)**	0.002
HIV Diagnoses	Facility level	Secondary	count	NB	752.2	0.91 (0.61-1.35)	0.63	1.35 (1.03-1.77)*	0.030	2.14 (1.60-2.87)***	<.001
HIV Diagnoses	Facility level	Tertiary	count	NB	655.8	0.24 (0.11-0.50)***	<.001	0.18 (0.11-0.32)***	<.001	0.16 (0.08-0.29)***	<.001
HIV Diagnoses	Ownership	NGO	count	NB	520.6	0.42 (0.22-0.80)**	0.008	0.21 (0.13-0.35)***	<.001	0.20 (0.12-0.35)***	<.001
HIV Diagnoses	Ownership	Public	count	NB	802.6	0.71 (0.53-0.95)*	0.022	0.71 (0.58-0.87)***	<.001	1.05 (0.84-1.30)	0.69
Linkage to Care	Overall	all	count	NB	822.3	0.72 (0.52-1.00)*	0.050	0.64 (0.51-0.81)***	<.001	0.98 (0.76-1.26)	0.85
Linkage to Care Rate	Overall	all	rate	Bin	699.4	1.09 (0.63-1.88)	0.76	0.60 (0.44-0.82)**	0.002	1.57 (1.01-2.45)*	0.047
Linkage to Care	Sex	Female	count	NB	747.3	0.46 (0.32-0.68)***	<.001	0.60 (0.46-0.77)***	<.001	0.84 (0.63-1.11)	0.22
Linkage to Care Rate	Sex	Female	rate	Bin	544.9	0.63 (0.32-1.24)	0.18	0.68 (0.44-1.07)	0.095	0.94 (0.56-1.57)	0.81
Linkage to Care	Sex	Male	count	NB	687.9	0.79 (0.56-1.12)	0.19	0.84 (0.66-1.07)	0.17	1.22 (0.94-1.58)	0.14
Linkage to Care Rate	Sex	Male	rate	Bin	473.0	0.92 (0.43-1.96)	0.83	0.68 (0.43-1.09)	0.11	5.02 (1.79-14.10)**	0.002
Linkage to Care	Age	0-24 years	count	NB	569.2	0.64 (0.41-0.99)*	0.046	0.47 (0.34-0.65)***	<.001	0.79 (0.57-1.09)	0.15
Linkage to Care Rate	Age	0-24 years	rate	Bin	385.4	1.66 (0.22-12.61)	0.62	0.72 (0.24-2.18)	0.56	0.33 (0.14-0.78)*	0.011
Linkage to Care	Age	25-34 years	count	NB	496.3	0.74 (0.58-0.94)*	0.014	0.91 (0.78-1.06)	0.21	0.93 (0.79-1.10)	0.43
Linkage to Care Rate	Age	25-34 years	rate	Bin	180.6	0.45 (0.14-1.47)	0.19	1.36 (0.42-4.42)	0.61	1.97 (0.52-7.45)	0.32
Linkage to Care	Age	35-44 years	count	NB	636.2	1.01 (0.73-1.40)	0.96	0.69 (0.54-0.88)**	0.003	0.93 (0.72-1.20)	0.57
Linkage to Care Rate	Age	35-44 years	rate	Bin	434.6	2.38 (0.73-7.75)	0.15	0.53 (0.31-0.89)*	0.016	1.82 (0.79-4.17)	0.16
Linkage to Care	Age	45+ years	count	NB	570.3	0.59 (0.37-0.94)*	0.027	0.78 (0.57-1.06)	0.11	1.20 (0.88-1.65)	0.26
Linkage to Care Rate	Age	45+ years	rate	Bin	356.2	2.11 (0.28-16.00)	0.47	0.69 (0.32-1.49)	0.34	3.74 (0.85-16.43)	0.081
Linkage to Care	Facility level	Primary	count	NB	741.1	0.55 (0.25-1.20)	0.13	0.26 (0.14-0.47)***	<.001	0.36 (0.19-0.70)**	0.002
Linkage to Care Rate	Facility level	Primary	rate	Bin	356.4	0.87 (0.30-2.49)	0.79	0.41 (0.20-0.84)*	0.015	1.17 (0.39-3.47)	0.78
Linkage to Care	Facility level	Secondary	count	NB	752.2	0.91 (0.59-1.40)	0.66	1.28 (0.95-1.73)	0.10	2.13 (1.55-2.94)***	<.001
Linkage to Care Rate	Facility level	Secondary	rate	Bin	630.8	0.80 (0.31-2.05)	0.64	0.38 (0.24-0.61)***	<.001	0.78 (0.42-1.42)	0.41
Linkage to Care	Facility level	Tertiary	count	NB	632.1	0.24 (0.11-0.51)***	<.001	0.17 (0.10-0.30)***	<.001	0.16 (0.08-0.29)***	<.001
Linkage to Care Rate	Facility level	Tertiary	rate	Bin	362.2	0.80 (0.32-1.98)	0.63	0.62 (0.33-1.16)	0.13	1.75 (0.65-4.72)	0.27

© 2026 Kebede HK et al. *JAMA Network Open*.

Outcome	Stratifier	Stratum	Type	Fam	AIC	Pandemic IRR (95% CI)	P	War IRR (95% CI)	P	Post-war IRR (95% CI)	P
Linkage to Care	Ownership	NGO	count	NB	515.8	0.39 (0.19-0.79)**	0.009	0.19 (0.11-0.33)***	<.001	0.17 (0.09-0.32)***	<.001
Linkage to Care	Ownership	Public	count	NB	816.0	0.71 (0.50-1.01)	0.056	0.67 (0.52-0.86)**	0.002	1.05 (0.80-1.37)	0.74
Linkage to Care Rate	Ownership	Public	rate	Bin	707.5	1.02 (0.58-1.79)	0.95	0.57 (0.41-0.79)***	<.001	1.45 (0.92-2.28)	0.11
ART Initiation	Overall	all	count	NB	806.0	0.77 (0.54-1.10)	0.15	0.67 (0.52-0.86)**	0.002	0.96 (0.73-1.26)	0.75
ART Initiation Rate	Overall	all	rate	Bin	1.2e+03	15.79 (3.91-63.87)***	<.001	11.59 (5.13-26.20)***	<.001	9.27 (4.53-18.94)***	<.001
ART Initiation	Sex	Female	count	NB	722.3	0.72 (0.49-1.05)	0.091	0.54 (0.41-0.71)***	<.001	0.81 (0.60-1.08)	0.15
ART Initiation Rate	Sex	Female	rate	Bin	1e+03	2.25 (0.90-5.61)	0.083	0.76 (0.53-1.09)	0.13	18.77 (4.61-76.45)***	<.001
ART Initiation	Sex	Male	count	NB	681.3	0.85 (0.57-1.25)	0.41	0.87 (0.67-1.15)	0.33	1.20 (0.90-1.61)	0.22
ART Initiation Rate	Sex	Male	rate	Bin	572.8	12.63 (1.74-91.51)*	0.012	8.26 (3.01-22.68)***	<.001	5.61 (2.40-13.09)***	<.001
ART Initiation	Age	0-24 years	count	NB	545.6	0.61 (0.39-0.95)*	0.030	0.42 (0.31-0.59)***	<.001	0.65 (0.47-0.91)*	0.011
ART Initiation	Age	25-34 years	count	QP	—	0.68 (0.54-0.86)**	0.001	0.84 (0.73-0.97)*	0.021	0.87 (0.74-1.02)	0.085
ART Initiation	Age	35-44 years	count	NB	615.0	1.04 (0.75-1.44)	0.81	0.69 (0.54-0.87)**	0.002	0.88 (0.68-1.13)	0.31
ART Initiation	Age	45+ years	count	NB	558.1	0.62 (0.38-1.02)	0.058	0.80 (0.58-1.11)	0.18	1.18 (0.84-1.65)	0.35
ART Initiation	Facility level	Primary	count	NB	684.1	0.61 (0.31-1.21)	0.15	0.28 (0.17-0.46)***	<.001	0.37 (0.21-0.64)***	<.001
ART Initiation Rate	Facility level	Primary	rate	Bin	500.1	6.31 (1.53-26.04)*	0.011	5.60 (1.74-18.00)**	0.004	33990267.17 (0.00-Inf)	0.98
ART Initiation	Facility level	Secondary	count	NB	746.1	1.00 (0.60-1.67)	1.00	1.36 (0.96-1.93)	0.088	2.14 (1.47-3.12)***	<.001
ART Initiation Rate	Facility level	Secondary	rate	Bin	961.6	15879229.42 (0.00-Inf)	0.98	34.00 (8.36-138.21)***	<.001	38.32 (9.39-156.34)***	<.001
ART Initiation	Facility level	Tertiary	count	NB	630.2	0.24 (0.11-0.53)***	<.001	0.18 (0.10-0.31)***	<.001	0.15 (0.08-0.29)***	<.001
ART Initiation	Ownership	NGO	count	NB	429.1	0.53 (0.32-0.88)*	0.014	0.27 (0.18-0.41)***	<.001	0.25 (0.16-0.38)***	<.001
ART Initiation	Ownership	Public	count	NB	797.5	0.74 (0.51-1.08)	0.12	0.68 (0.52-0.88)**	0.004	0.99 (0.75-1.32)	0.97
ART Initiation Rate	Ownership	Public	rate	Bin	1.1e+03	23.14 (3.23-165.83)**	0.002	17.43 (5.55-54.73)***	<.001	6.77 (3.30-13.90)***	<.001

© 2026 Kebede HK et al. *JAMA Network Open*.

eTable 11. Sensitivity Analysis 4: Prewar Restricted to 2015 Onward

Pre-war baseline restricted to Q1 2015 – Q4 2019 (20 quarters). War IRRs are notably more negative, reflecting a higher recent baseline.

Outcome	Stratifier	Stratum	Type	Fam	AIC	Pandemic IRR (95% CI)	P	War IRR (95% CI)	P	Post-war IRR (95% CI)	P
HIV Diagnoses	Overall	all	count	NB	390.1	0.53 (0.39-0.72)***	<.001	0.52 (0.38-0.73)***	<.001	0.62 (0.39-0.98)*	0.039
HIV Diagnoses	Sex	Female	count	NB	349.9	0.52 (0.37-0.72)***	<.001	0.47 (0.33-0.67)***	<.001	0.61 (0.38-1.00)*	0.048
HIV Diagnoses	Sex	Male	count	NB	321.2	0.54 (0.40-0.73)***	<.001	0.58 (0.42-0.80)***	<.001	0.60 (0.39-0.92)*	0.019
HIV Diagnoses	Age	0-24 years	count	NB	275.1	0.48 (0.29-0.77)**	0.002	0.41 (0.24-0.68)***	<.001	0.54 (0.27-1.07)	0.076
HIV Diagnoses	Age	25-34 years	count	QP	—	0.86 (0.70-1.04)	0.13	1.05 (0.86-1.30)	0.62	1.14 (0.86-1.51)	0.36
HIV Diagnoses	Age	35-44 years	count	NB	299.6	0.67 (0.49-0.91)**	0.010	0.55 (0.39-0.77)***	<.001	0.58 (0.37-0.90)*	0.015
HIV Diagnoses	Age	45+ years	count	NB	261.6	0.42 (0.28-0.62)***	<.001	0.49 (0.33-0.72)***	<.001	0.54 (0.33-0.89)*	0.016
HIV Diagnoses	Facility level	Primary	count	NB	335.5	0.63 (0.39-1.01)	0.056	0.41 (0.24-0.69)***	<.001	0.60 (0.29-1.24)	0.17
HIV Diagnoses	Facility level	Secondary	count	NB	328.5	0.51 (0.37-0.71)***	<.001	0.69 (0.48-0.98)*	0.036	0.75 (0.47-1.20)	0.23
HIV Diagnoses	Facility level	Tertiary	count	NB	294.8	0.42 (0.28-0.64)***	<.001	0.39 (0.25-0.61)***	<.001	0.42 (0.23-0.75)**	0.003
HIV Diagnoses	Ownership	NGO	count	NB	255.5	0.78 (0.49-1.26)	0.31	0.63 (0.37-1.06)	0.084	0.87 (0.44-1.75)	0.70
HIV Diagnoses	Ownership	Public	count	NB	382.4	0.50 (0.37-0.69)***	<.001	0.51 (0.36-0.72)***	<.001	0.59 (0.37-0.95)*	0.029
Linkage to Care	Overall	all	count	NB	392.4	0.52 (0.37-0.72)***	<.001	0.49 (0.34-0.71)***	<.001	0.59 (0.36-0.98)*	0.043
Linkage to Care Rate	Overall	all	rate	Bin	225.8	0.59 (0.35-1.02)	0.058	0.39 (0.23-0.68)***	<.001	0.71 (0.33-1.54)	0.39
Linkage to Care	Sex	Female	count	NB	351.0	0.50 (0.35-0.72)***	<.001	0.43 (0.29-0.64)***	<.001	0.57 (0.34-0.97)*	0.040
Linkage to Care Rate	Sex	Female	rate	Bin	185.9	0.61 (0.29-1.28)	0.20	0.39 (0.19-0.80)*	0.010	0.45 (0.17-1.19)	0.11
Linkage to Care	Sex	Male	count	NB	323.3	0.52 (0.37-0.73)***	<.001	0.54 (0.38-0.78)***	<.001	0.58 (0.35-0.94)*	0.027
Linkage to Care Rate	Sex	Male	rate	Bin	167.0	0.53 (0.24-1.18)	0.12	0.39 (0.17-0.88)*	0.023	2.00 (0.49-8.19)	0.33
Linkage to Care	Age	0-24 years	count	NB	272.5	0.45 (0.28-0.72)***	<.001	0.38 (0.23-0.64)***	<.001	0.51 (0.26-0.99)*	0.046
Linkage to Care Rate	Age	0-24 years	rate	Bin	98.4	4.81 (0.51-45.75)	0.17	5.13 (0.71-36.88)	0.10	10.46 (0.73-150.01)	0.084
Linkage to Care	Age	25-34 years	count	NB	256.1	0.80 (0.63-1.03)	0.089	0.99 (0.76-1.29)	0.97	1.05 (0.74-1.50)	0.77
Linkage to Care Rate	Age	25-34 years	rate	Bin	101.3	0.07 (0.01-0.53)**	0.009	0.07 (0.01-0.80)*	0.032	0.03 (0.00-0.78)*	0.035
Linkage to Care	Age	35-44 years	count	NB	300.0	0.71 (0.51-0.98)*	0.038	0.53 (0.37-0.76)***	<.001	0.59 (0.36-0.96)*	0.034
Linkage to Care Rate	Age	35-44 years	rate	Bin	157.7	1.33 (0.52-3.37)	0.55	0.64 (0.27-1.54)	0.32	1.97 (0.53-7.34)	0.31
Linkage to Care	Age	45+ years	count	NB	259.9	0.40 (0.27-0.60)***	<.001	0.46 (0.31-0.68)***	<.001	0.52 (0.31-0.87)*	0.014
Linkage to Care Rate	Age	45+ years	rate	Bin	109.9	0.50 (0.12-2.20)	0.36	0.35 (0.09-1.44)	0.15	1.15 (0.12-10.78)	0.90
Linkage to Care	Facility level	Primary	count	NB	335.4	0.63 (0.38-1.03)	0.065	0.38 (0.22-0.67)***	<.001	0.58 (0.27-1.24)	0.16
Linkage to Care Rate	Facility level	Primary	rate	Bin	131.6	0.89 (0.27-3.00)	0.86	0.30 (0.10-0.92)*	0.035	0.73 (0.14-3.90)	0.71
Linkage to Care	Facility level	Secondary	count	NB	329.0	0.49 (0.34-0.71)***	<.001	0.65 (0.44-0.96)*	0.029	0.72 (0.42-1.21)	0.21
Linkage to Care Rate	Facility level	Secondary	rate	Bin	173.2	0.63 (0.28-1.44)	0.28	0.48 (0.22-1.08)	0.075	0.74 (0.24-2.24)	0.59
Linkage to Care	Facility level	Tertiary	count	NB	294.5	0.38 (0.23-0.60)***	<.001	0.36 (0.22-0.59)***	<.001	0.39 (0.20-0.75)**	0.005
Linkage to Care Rate	Facility level	Tertiary	rate	Bin	149.5	0.36 (0.14-0.91)*	0.030	0.36 (0.13-0.99)*	0.048	0.76 (0.17-3.32)	0.71

© 2026 Kebede HK et al. *JAMA Network Open*.

Outcome	Stratifier	Stratum	Type	Fam	AIC	Pandemic IRR (95% CI)	P	War IRR (95% CI)	P	Post-war IRR (95% CI)	P
Linkage to Care	Ownership	NGO	count	NB	251.1	0.85 (0.53-1.35)	0.49	0.68 (0.41-1.15)	0.15	1.00 (0.50-1.98)	1.00
Linkage to Care	Ownership	Public	count	NB	385.0	0.48 (0.33-0.68)***	<.001	0.47 (0.32-0.69)***	<.001	0.55 (0.32-0.94)*	0.028
Linkage to Care Rate	Ownership	Public	rate	Bin	223.0	0.47 (0.27-0.82)**	0.008	0.31 (0.17-0.54)***	<.001	0.48 (0.22-1.08)	0.076
ART Initiation	Overall	all	count	NB	394.0	0.48 (0.33-0.70)***	<.001	0.43 (0.28-0.64)***	<.001	0.45 (0.26-0.79)**	0.005
ART Initiation Rate	Overall	all	rate	Bin	175.4	0.77 (0.22-2.64)	0.67	0.34 (0.12-1.01)	0.052	0.06 (0.02-0.18)***	<.001
ART Initiation	Sex	Female	count	NB	355.5	0.48 (0.32-0.71)***	<.001	0.38 (0.24-0.59)***	<.001	0.45 (0.24-0.82)**	0.010
ART Initiation Rate	Sex	Female	rate	Bin	223.5	0.01 (0.01-0.03)***	<.001	0.29 (0.06-1.49)	0.14	0.07 (0.01-0.46)**	0.005
ART Initiation	Sex	Male	count	NB	323.2	0.49 (0.34-0.70)***	<.001	0.48 (0.33-0.71)***	<.001	0.46 (0.27-0.78)**	0.004
ART Initiation Rate	Sex	Male	rate	Bin	123.3	0.75 (0.16-3.58)	0.72	0.49 (0.11-2.11)	0.34	0.08 (0.02-0.38)**	0.001
ART Initiation	Age	0-24 years	count	NB	274.7	0.44 (0.27-0.72)**	0.001	0.37 (0.21-0.62)***	<.001	0.47 (0.23-0.94)*	0.034
ART Initiation	Age	25-34 years	count	NB	258.8	0.80 (0.62-1.02)	0.074	0.99 (0.77-1.29)	0.97	1.07 (0.76-1.51)	0.71
ART Initiation	Age	35-44 years	count	NB	299.7	0.67 (0.47-0.94)*	0.022	0.46 (0.31-0.68)***	<.001	0.46 (0.27-0.77)**	0.003
ART Initiation	Age	45+ years	count	NB	261.0	0.38 (0.24-0.58)***	<.001	0.39 (0.25-0.61)***	<.001	0.39 (0.22-0.69)**	0.001
ART Initiation	Facility level	Primary	count	NB	333.9	0.55 (0.32-0.94)*	0.029	0.30 (0.17-0.54)***	<.001	0.38 (0.17-0.86)*	0.021
ART Initiation	Facility level	Secondary	count	NB	333.9	0.47 (0.31-0.70)***	<.001	0.57 (0.37-0.88)*	0.012	0.55 (0.30-1.01)	0.053
ART Initiation Rate	Facility level	Secondary	rate	Bin	137.7	1.20 (0.15-9.69)	0.87	1.25 (0.14-10.95)	0.84	0.19 (0.03-1.27)	0.087
ART Initiation	Facility level	Tertiary	count	NB	291.5	0.38 (0.24-0.60)***	<.001	0.35 (0.22-0.58)***	<.001	0.37 (0.19-0.70)**	0.002
ART Initiation	Ownership	NGO	count	NB	246.8	0.73 (0.45-1.20)	0.21	0.48 (0.28-0.85)*	0.012	0.57 (0.27-1.20)	0.14
ART Initiation	Ownership	Public	count	NB	385.9	0.45 (0.31-0.66)***	<.001	0.42 (0.28-0.64)***	<.001	0.44 (0.25-0.78)**	0.005
ART Initiation Rate	Ownership	Public	rate	Bin	155.3	0.87 (0.19-3.89)	0.86	0.70 (0.15-3.32)	0.65	0.05 (0.01-0.17)***	<.001

© 2026 Kebede HK et al. *JAMA Network Open*.

eTable 12. Period × Stratifier Interaction Tests (Likelihood Ratio Tests)

For each outcome–stratifier combination, two MASS::glm.nb models were compared by likelihood ratio test: a main-effects-only model (linear time + period indicators + seasonal harmonics + stratum) versus a model adding period × stratum interactions. A significant LR statistic indicates that the period effects differ across strata of that stratifier (i.e., effect modification).

Outcome	Stratifier	Test	χ^2	df	P value
HIV Diagnoses	Sex	LR: period x stratum	4.86	3	0.18
Linkage to Care	Sex	LR: period x stratum	3.70	3	0.30
ART Initiation	Sex	LR: period x stratum	1.69	3	0.64
HIV Diagnoses	Age	LR: period x stratum	92.08	9	<.001
Linkage to Care	Age	LR: period x stratum	70.41	9	<.001
ART Initiation	Age	LR: period x stratum	28.02	9	<.001
HIV Diagnoses	Ownership	LR: period x stratum	20.78	3	<.001
Linkage to Care	Ownership	LR: period x stratum	20.05	3	<.001
ART Initiation	Ownership	LR: period x stratum	31.98	3	<.001
HIV Diagnoses	Facility level	LR: period x stratum	4.55	6	0.60
Linkage to Care	Facility level	LR: period x stratum	5.35	6	0.50
ART Initiation	Facility level	LR: period x stratum	5.98	6	0.43

Significant interactions (P < .05) were observed for Age and Ownership across all three outcomes, indicating that pandemic, war, and post-war effects differed substantially across age groups and between Public vs NGO facilities. Sex and Facility level showed no significant effect modification for any outcome. These results corroborate the stratum-specific IRRs in eTable 5.

© 2026 Kebede HK et al. *JAMA Network Open*.

eTable 13. Cumulative Cascade Deficits With Parametric Bootstrap 95% CIs

Outcome	n war Q	n post-war Q	War deficit	Post-war deficit	Total deficit	95% CI (bootstrap)	Method
HIV Diagnoses	8	9	+247	+17	+265	(+7 to +544)	NB+bootstrap
Linkage to Care	8	9	+259	+17	+276	(-38 to +610)	NB+bootstrap
ART Initiation	8	9	+232	+32	+264	(-68 to +624)	NB+bootstrap

Deficits computed as Σ(counterfactual − observed) over war (n=8) and post-war (n=9) quarters using the fitted negative binomial segmented regression with seasonal harmonics. 95% CIs from 5,000 parametric bootstrap replicates. Positive values indicate that observed counts fell below the counterfactual trajectory.

© 2026 Kebede HK et al. *JAMA Network Open*.

eAppendix 1. Description of eFigures

Count-model interrupted time-series analysis

Tigray War and HIV Care Cascade Entry in Mekelle, Ethiopia

Hafte Kahsay Kebede, BPharm, MSc; Hailay Abrha Gesesew, PhD; Lillian Mwanri, PhD; Paul Ward, PhD

Scope. This document presents 12 eFigures organised in five sections: stratified main analyses, DHARMa diagnostics for the main analysis, ARIMA complementary fits and diagnostics, four sensitivity analyses, and a sensitivity DHARMa overview. It deliberately excludes (a) Figure 1 of the main manuscript (descriptive overview) and Figure 2 (overall cascade fit), and (b) all rate-based offset-model figures, in line with the count-model focus of the manuscript. Each eFigure groups multiple individual source files (from the R output directory) into one composite display.

Note on additional files. *Beyond the 12 eFigures presented here, the analysis also produced (i) 132 stratum-level sensitivity ITS plots (4 sensitivities × 33 strata-outcome combinations) and (ii) 33 stratum-level DHARMa diagnostic composites for the main analysis (3 outcomes × 11 stratum levels). These granular files are not included here to keep the supplement at a manageable length; they are available from the corresponding author on request. All rate-model figures (*_rate*) are deliberately excluded.*

Contents (12 eFigures, count models only)

Section A. Stratified main analyses

eFigure 1. Sex-stratified main analysis (6 panels)

eFigure 2. Age-stratified main analysis (12 panels)

eFigure 3. Facility-level stratified main analysis (9 panels)

eFigure 4. Ownership-stratified main analysis (6 panels)

Section B. Main analysis DHARMa diagnostics

eFigure 5. Overall DHARMa residual diagnostics (3 panels)

Section C. ARIMA complementary analysis

eFigure 6. ARIMA fitted vs observed (3 panels)

eFigure 7. ARIMA residual diagnostics (3 panels)

Section D. Sensitivity analyses (overall)

eFigure 8. Sensitivity 1 — no seasonality (3 panels)

eFigure 9. Sensitivity 2 — full slope-change (3 panels)

eFigure 10. Sensitivity 3 — alt war onset Q4 2020 (3 panels)

eFigure 11. Sensitivity 4 — recent baseline 2015+ (3 panels)

Section E. Sensitivity DHARMa diagnostics

eFigure 12. Sensitivity DHARMa overview (12 panels)

© 2026 Kebede HK et al. *JAMA Network Open*.

Section A. Stratified main analyses

eFigure 1. Sex-Stratified Main Analysis: Cascade Fits by Female and Male

▶ **Source file(s):**

- *main_its_hiv_diagnoses_female_count.pdf*
- *main_its_hiv_diagnoses_male_count.pdf*
- *main_its_linkage_to_care_female_count.pdf*
- *main_its_linkage_to_care_male_count.pdf*
- *main_its_art_initiation_female_count.pdf*
- *main_its_art_initiation_male_count.pdf*

© 2026 Kebede HK et al. *JAMA Network Open*.

A

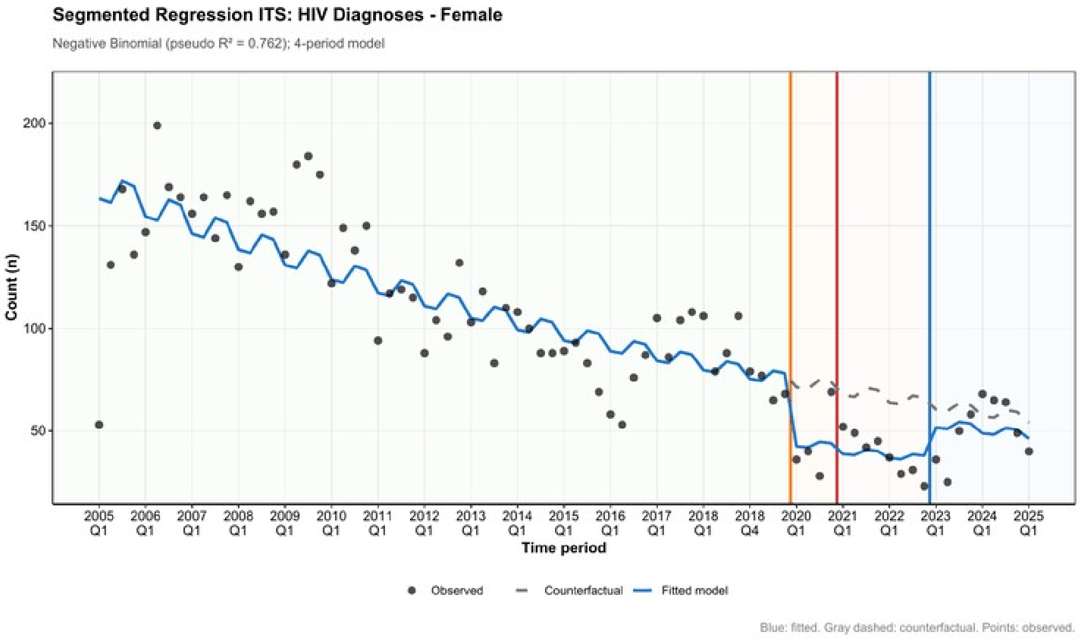

B

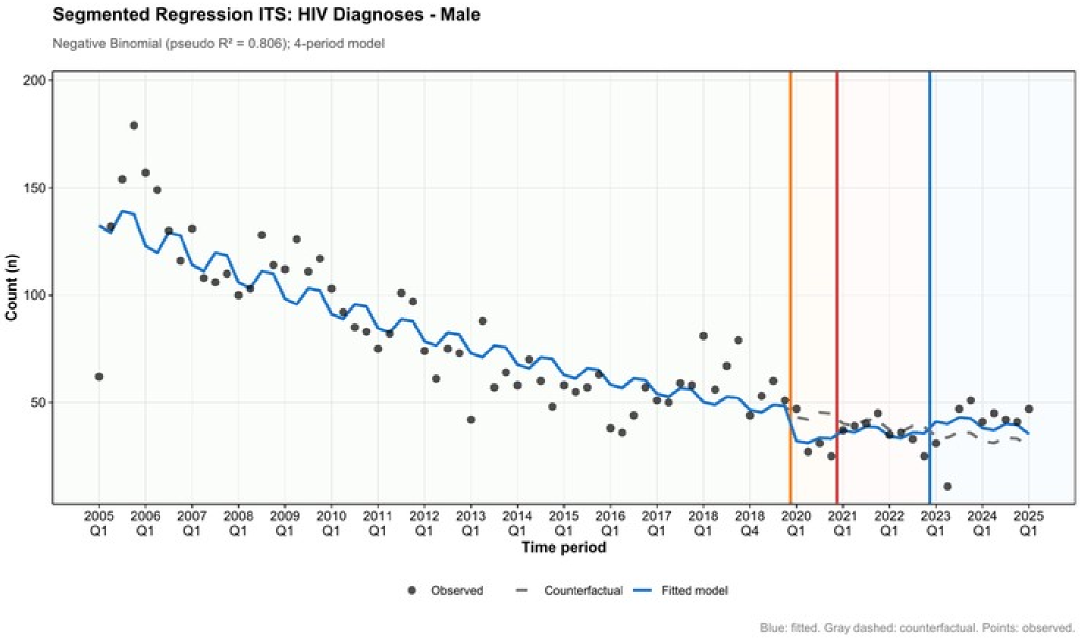

© 2026 Kebede HK et al. *JAMA Network Open*.

C

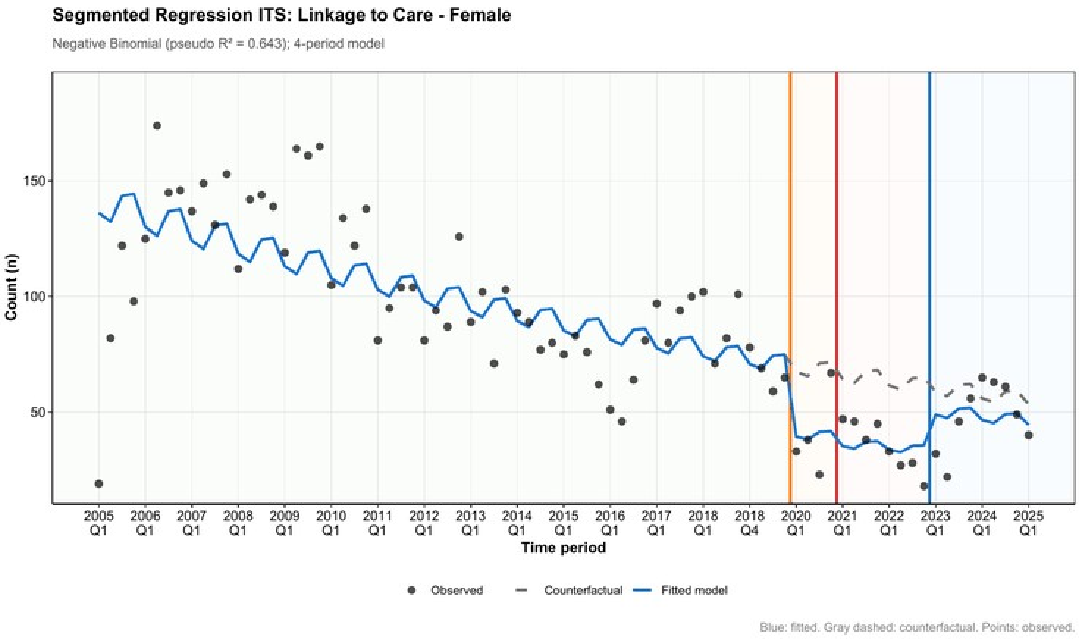

D

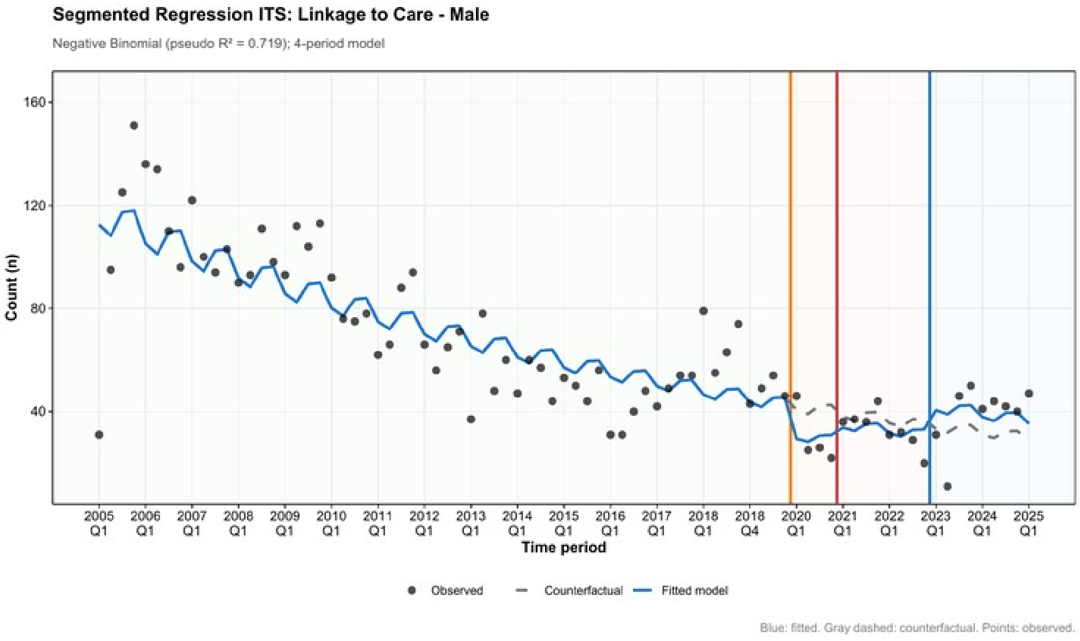

© 2026 Kebede HK et al. *JAMA Network Open*.

E

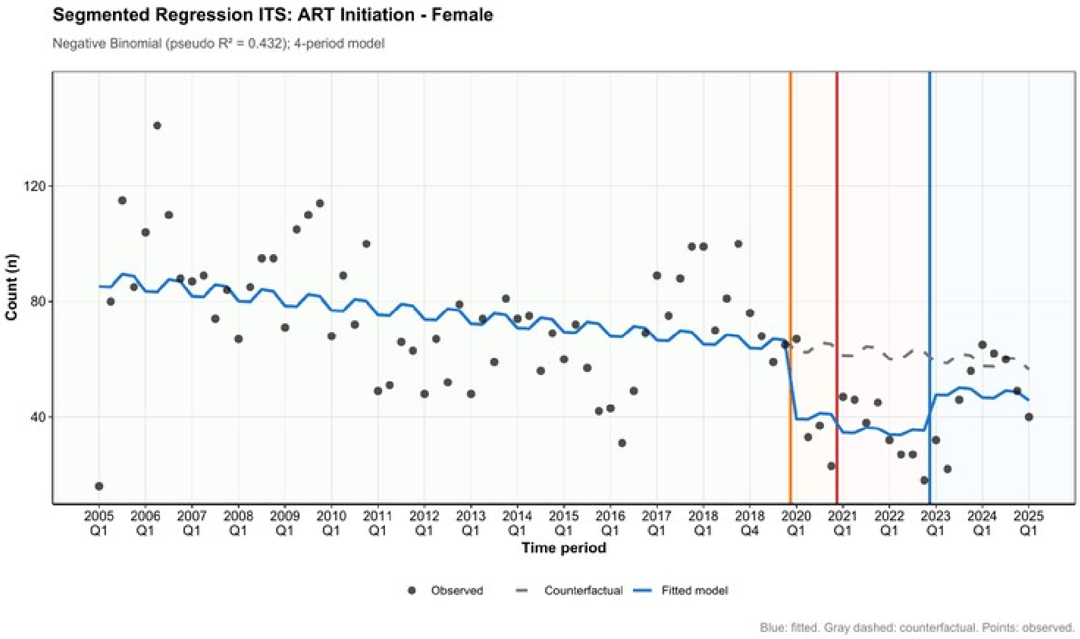

F

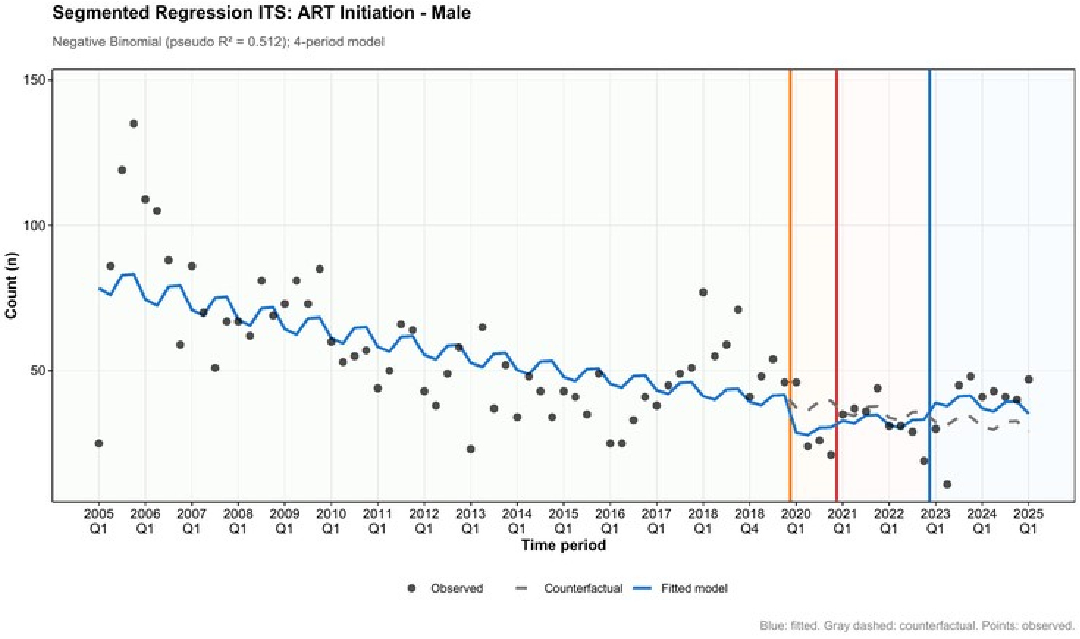

eFigure 1. Six-panel composite of negative binomial segmented regression fits, stratified by sex. Top row: HIV diagnoses (Female; Male). Middle row: linkage to care (Female; Male). Bottom row: ART initiation (Female; Male). In each panel, the solid blue line is the fitted segmented regression, the dashed gray line is the counterfactual

(intervention regressors set to 0), and points are observed quarterly counts. Background shading marks pre-war (green), pandemic (orange), war (red), and post-war (blue) periods. Larger war-period reductions among women than men are visually evident across all three outcomes; corresponding IRRs are reported in eTable 5.

© 2026 Kebede HK et al. *JAMA Network Open*.

eFigure 2. Age-Stratified Main Analysis: Cascade Fits by 4 Age Groups

▶ **Source file(s):**

- *main_its_hiv_diagnoses_0_24_years_count.pdf*
- *main_its_hiv_diagnoses_25_34_years_count.pdf*
- *main_its_hiv_diagnoses_35_44_years_count.pdf*
- *main_its_hiv_diagnoses_45_years_count.pdf*
- *main_its_linkage_to_care_0_24_years_count.pdf*
- *main_its_linkage_to_care_25_34_years_count.pdf*
- *main_its_linkage_to_care_35_44_years_count.pdf*
- *main_its_linkage_to_care_45_years_count.pdf*
- *main_its_art_initiation_0_24_years_count.pdf*
- *main_its_art_initiation_25_34_years_count.pdf*
- *main_its_art_initiation_35_44_years_count.pdf*
- *main_its_art_initiation_45_years_count.pdf*

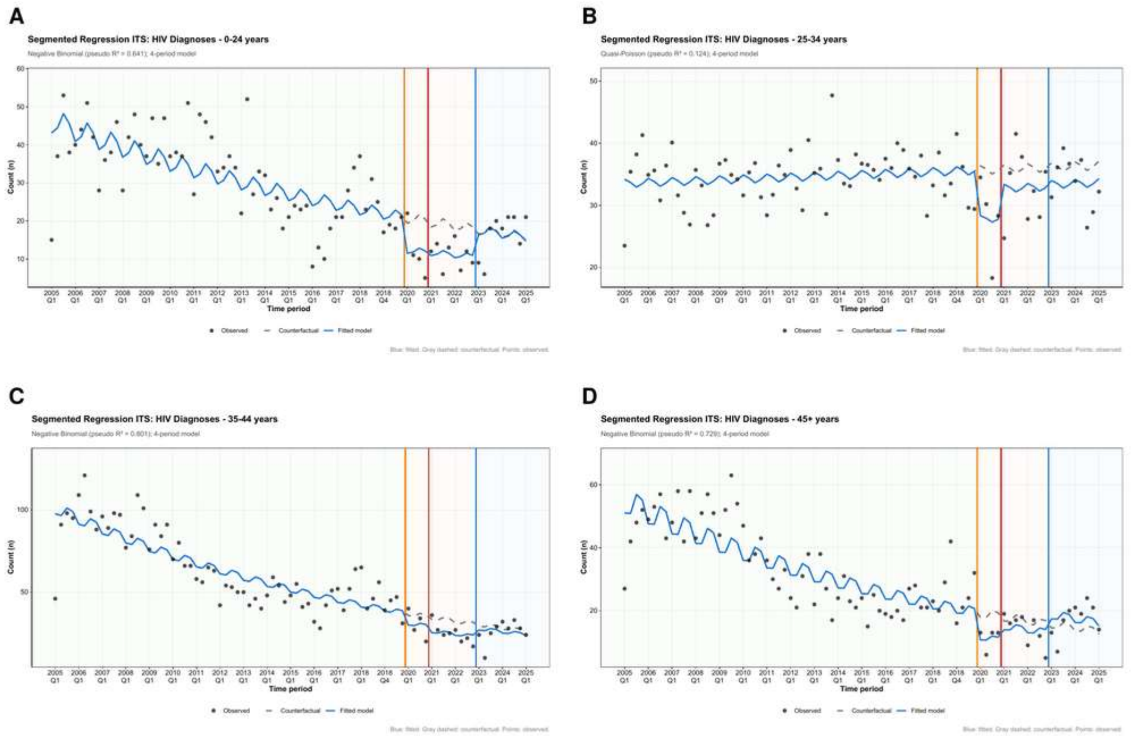

© 2026 Kebede HK et al. *JAMA Network Open*.

E

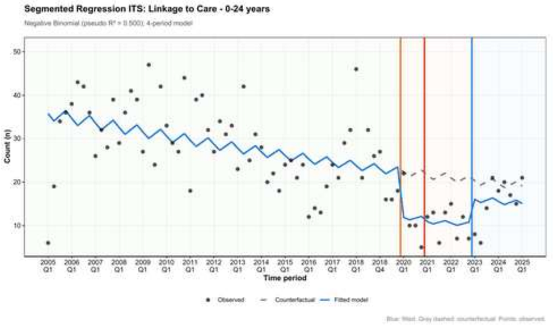

F

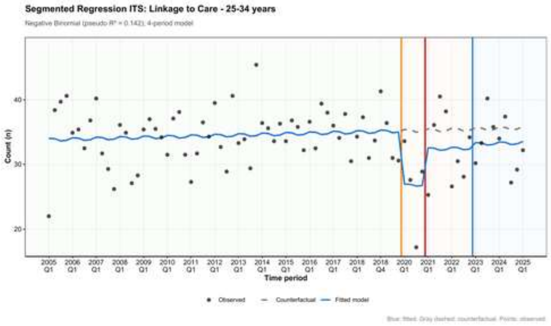

G

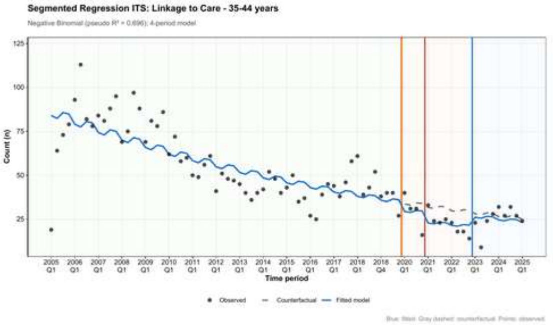

H

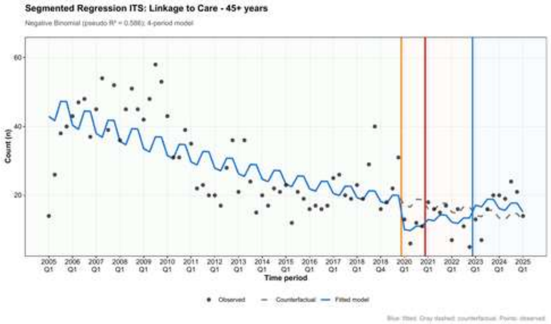

© 2026 Kebede HK et al. *JAMA Network Open*.

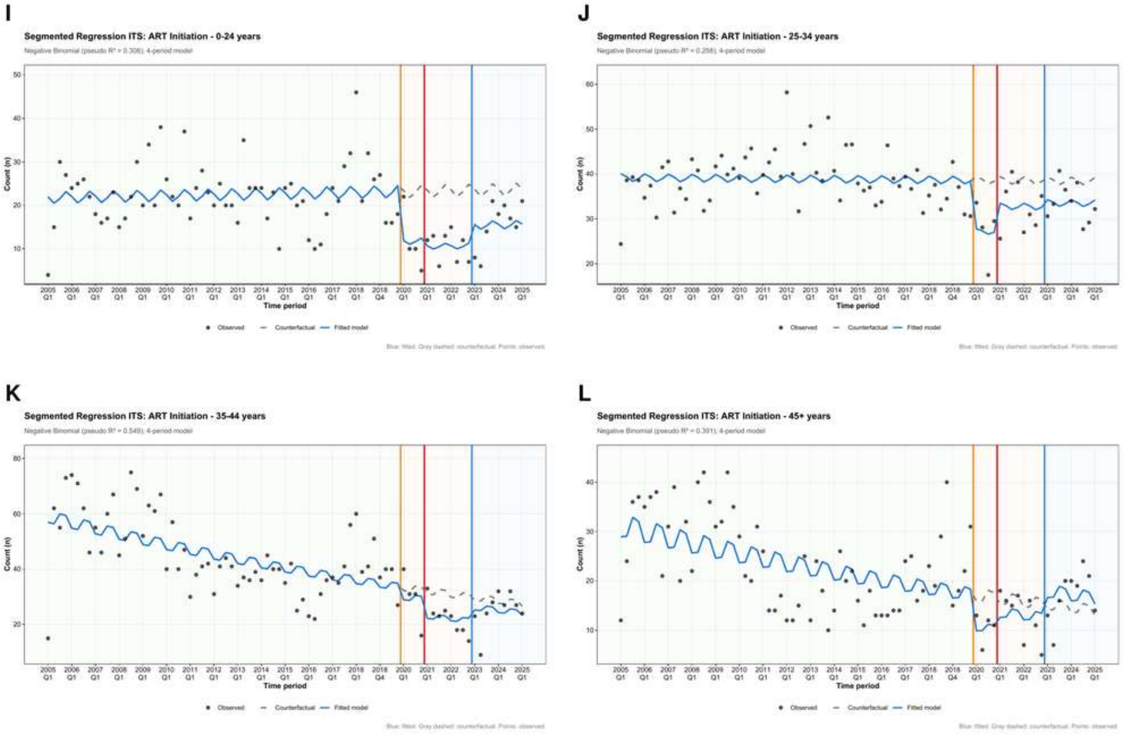

eFigure 2. Twelve-panel composite of negative binomial segmented regression fits, stratified by age group (0–24, 25–34, 35–44, ≥45 years). Three rows = cascade outcomes (HIV diagnoses, linkage, ART initiation); four columns = age strata. The 0–24-year stratum shows the largest war-period reductions across all three outcomes; the 25–34-year stratum shows the smallest changes. The likelihood-ratio test for period × age interaction was highly significant for all three outcomes (Table 3; all $P < .001$). The HIV Diagnoses, age 25–34-year model is the single quasi-Poisson fit (all others are negative binomial).

© 2026 Kebede HK et al. *JAMA Network Open*.

eFigure 3. Facility-Level Stratified Main Analysis: Primary, Secondary, Tertiary

▶ **Source file(s):**

- *main_its_hiv_diagnoses_primary_count.pdf*
- *main_its_hiv_diagnoses_secondary_count.pdf*
- *main_its_hiv_diagnoses_tertiary_count.pdf*
- *main_its_linkage_to_care_primary_count.pdf*
- *main_its_linkage_to_care_secondary_count.pdf*
- *main_its_linkage_to_care_tertiary_count.pdf*
- *main_its_art_initiation_primary_count.pdf*
- *main_its_art_initiation_secondary_count.pdf*
- *main_its_art_initiation_tertiary_count.pdf*

© 2026 Kebede HK et al. *JAMA Network Open*.

A

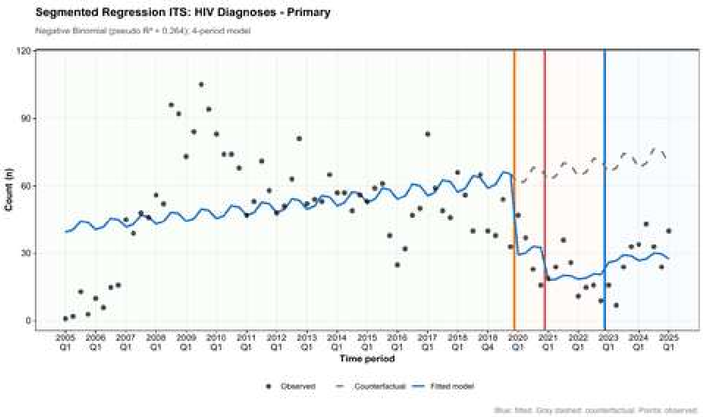

B

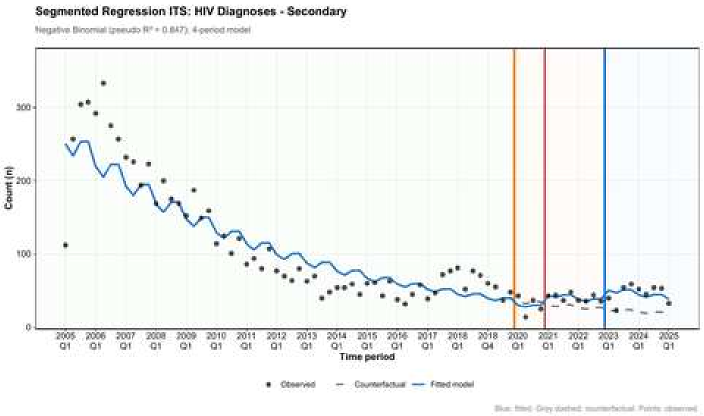

C

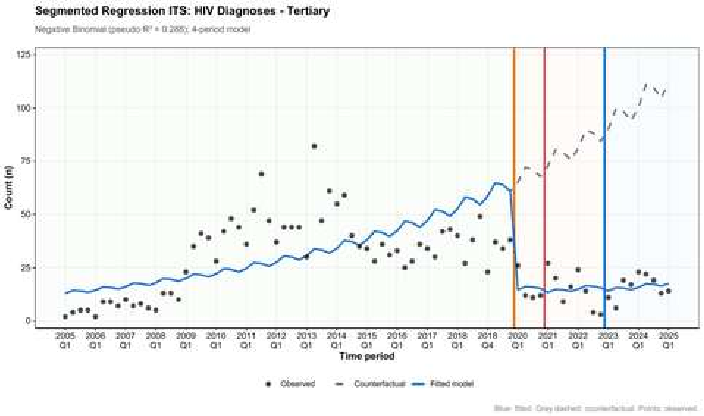

© 2026 Kebede HK et al. *JAMA Network Open*.

D

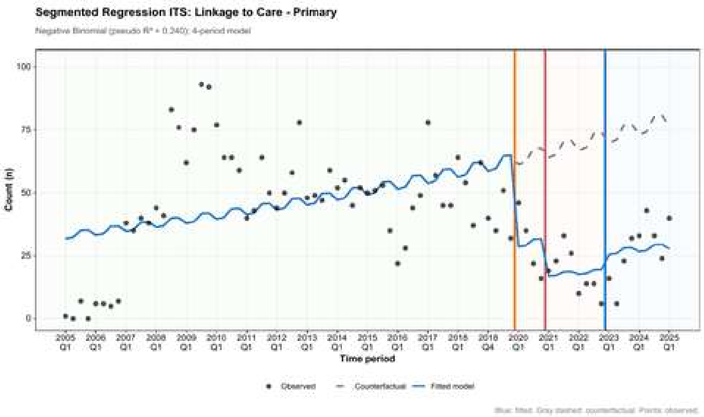

E

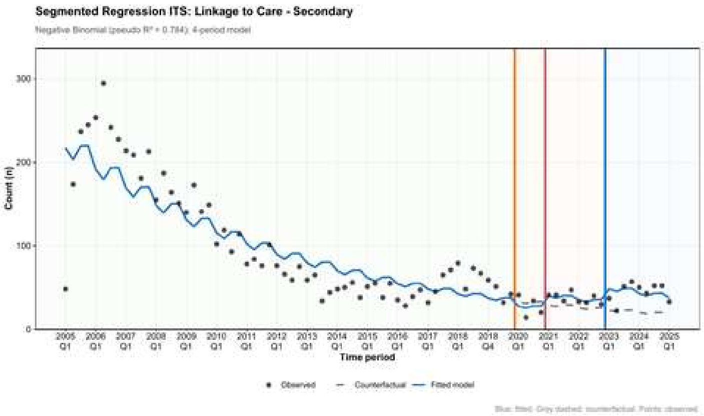

F

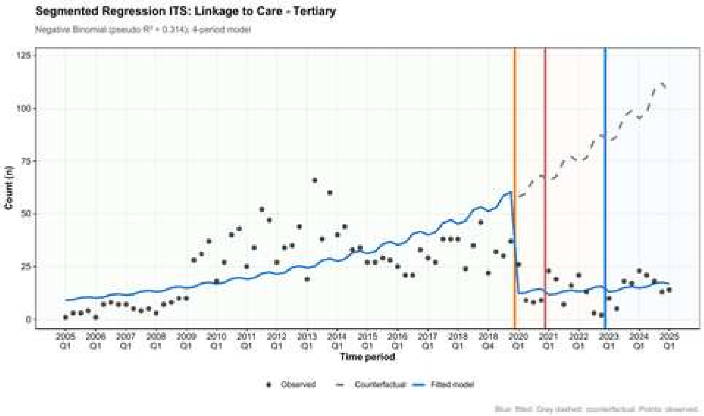

© 2026 Kebede HK et al. *JAMA Network Open*.

G

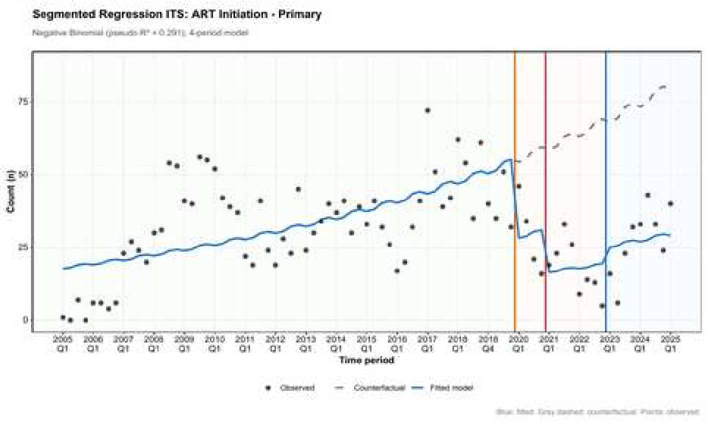

H

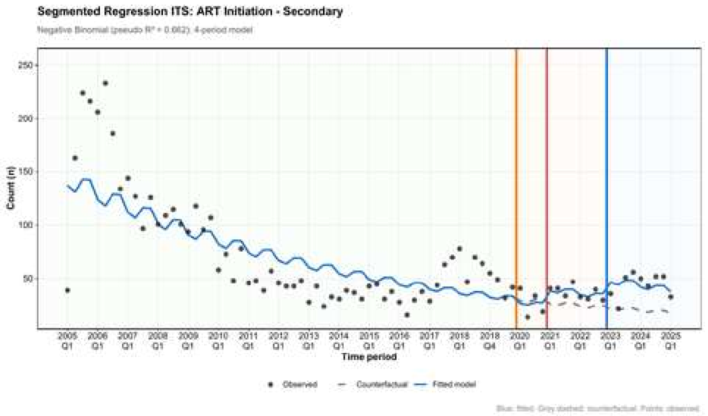

I

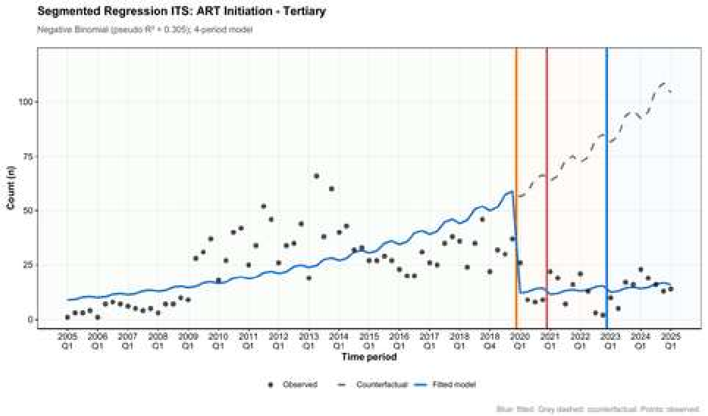

eFigure 3. Nine-panel composite stratified by facility level. Three rows (HIV diagnoses, linkage, ART initiation) by three columns (Primary, Secondary, Tertiary). Notable pattern: secondary-level general hospitals show war-period

INCREASES (war IRRs ≈ 1.38–1.46) and continued elevation post-war (IRRs ≈ 2.15–2.16), consistent with redistribution of services from primary (IRRs ≈ 0.26–0.29) and tertiary (IRRs ≈ 0.18) sites toward secondary general hospitals during and after the conflict. The period × facility-level interaction did not reach significance in the LR test (Table 3), reflecting opposite-direction shifts that partially cancel.

eFigure 4. Ownership-Stratified Main Analysis: Public and the Single NGO-Managed Facility

▶ **Source file(s):**

- *main_its_hiv_diagnoses_public_count.pdf*
- *main_its_hiv_diagnoses_ngo_count.pdf*
- *main_its_linkage_to_care_public_count.pdf*
- *main_its_linkage_to_care_ngo_count.pdf*
- *main_its_art_initiation_public_count.pdf*
- *main_its_art_initiation_ngo_count.pdf*

© 2026 Kebede HK et al. *JAMA Network Open*.

A

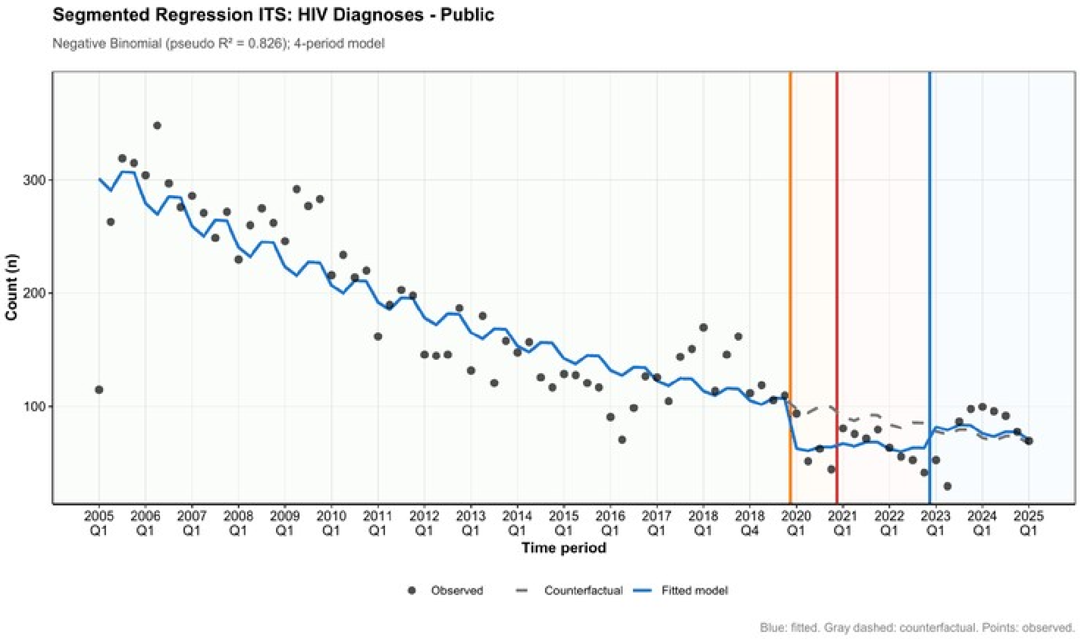

B

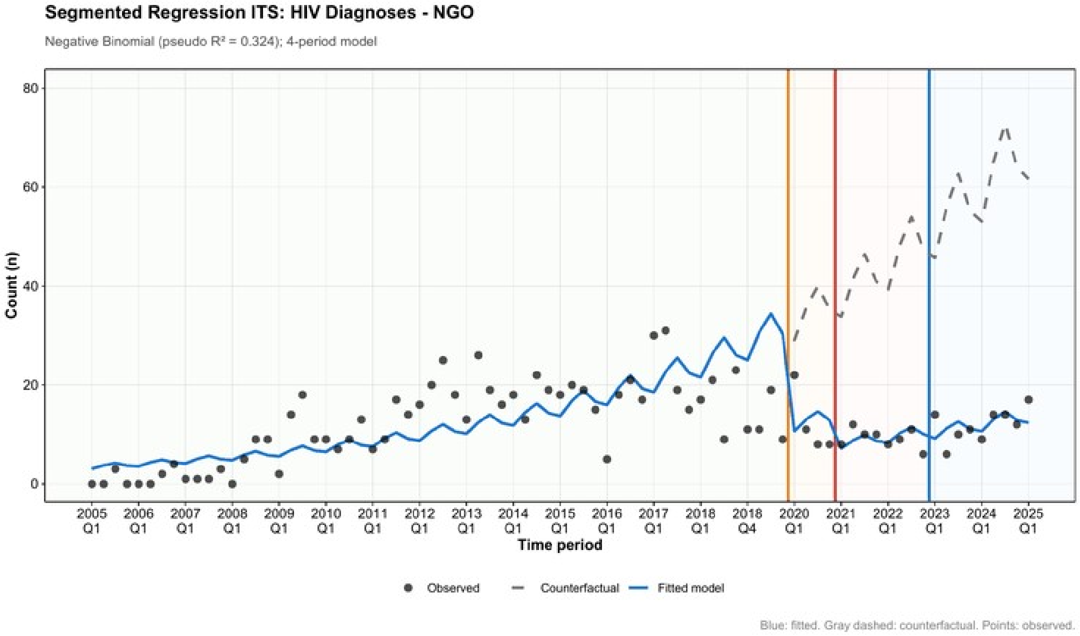

© 2026 Kebede HK et al. *JAMA Network Open*.

C

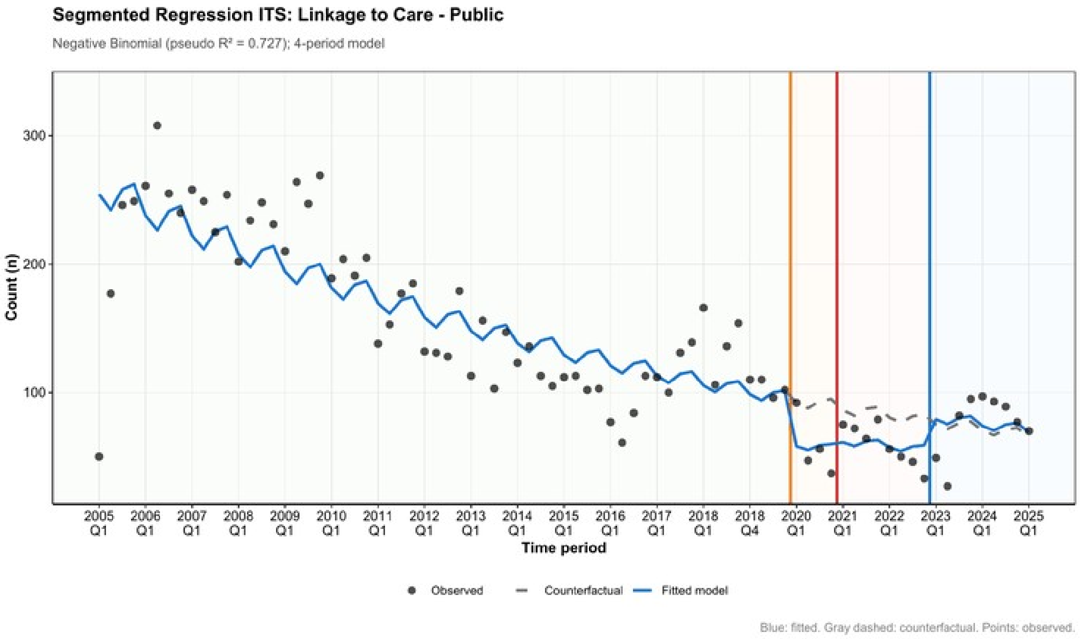

D

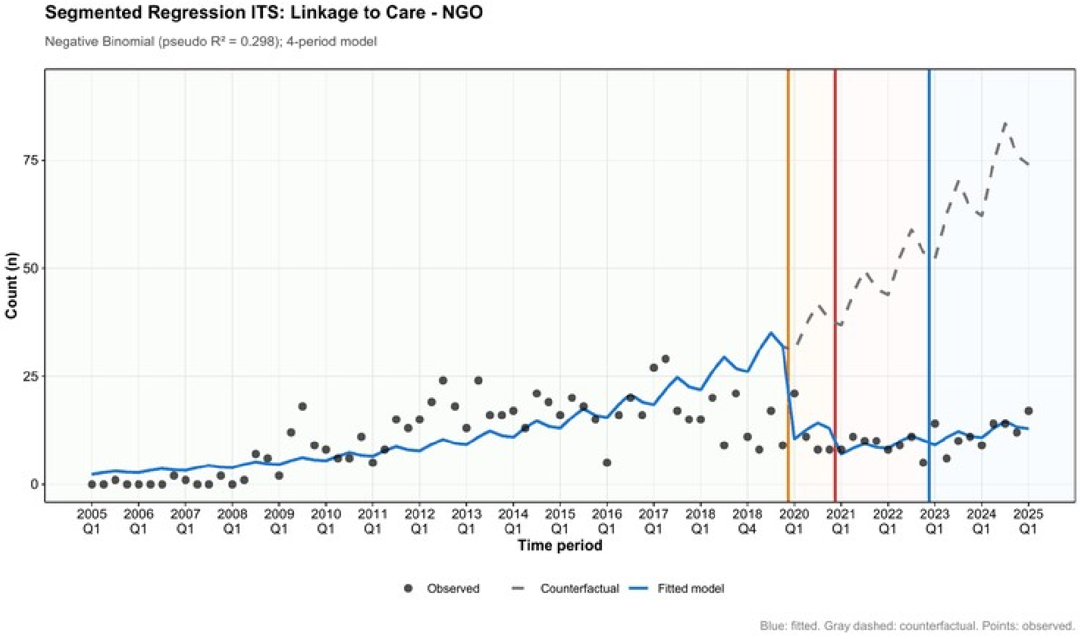

© 2026 Kebede HK et al. *JAMA Network Open*.

E

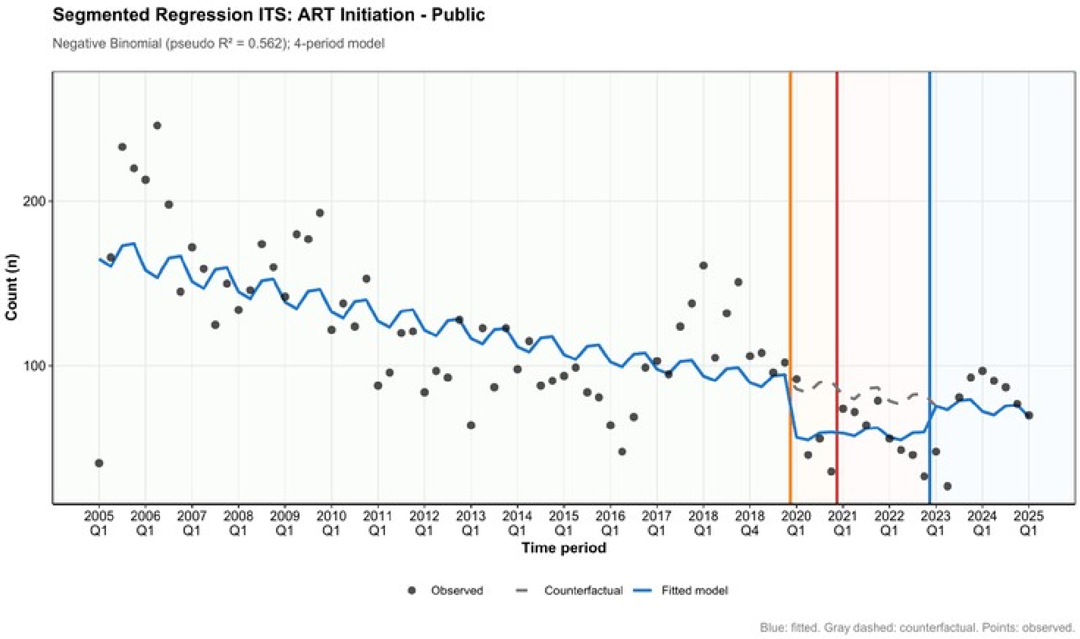

F

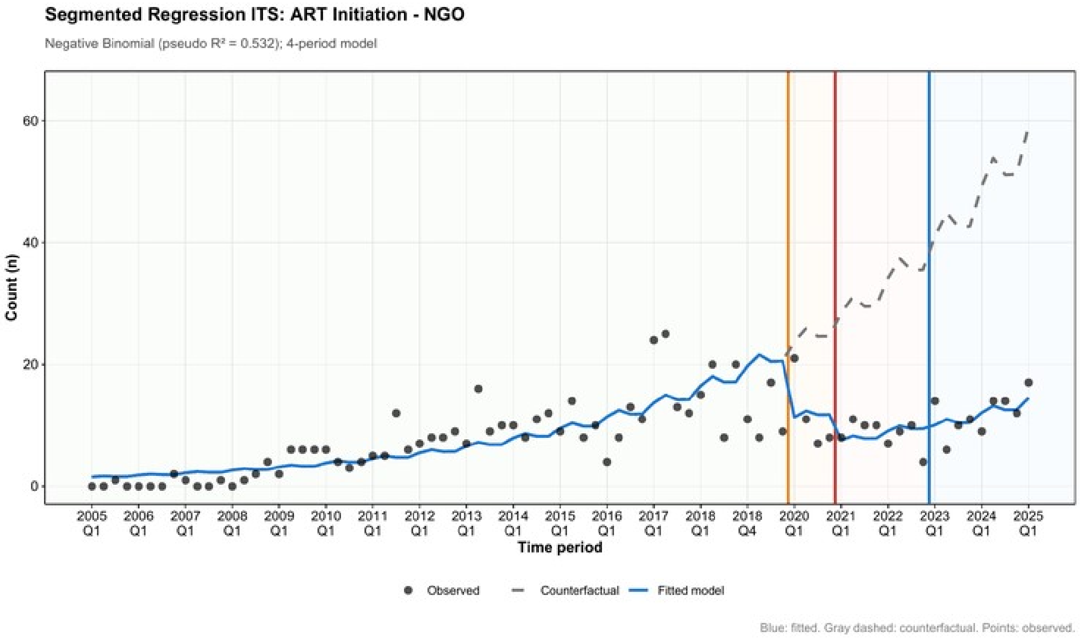

© 2026 Kebede HK et al. *JAMA Network Open*.

eFigure 4. Six-panel composite stratified by facility ownership. Three rows (HIV diagnoses, linkage, ART initiation) by two columns (Public; NGO). Public facilities (left) showed moderate war-period reductions (IRRs ≈ 0.72–0.74) with post-war values consistent with pre-war. The single NGO-managed facility (right; n = 1, not generalisable) showed large war-period reductions (IRRs ≈ 0.19–0.27) and no post-war recovery (post-war IRRs ≈ 0.17–0.25). The period × ownership interaction was highly significant for all three outcomes (Table 3; all P < .001). CAVEAT: the NGO stratum reflects a single facility and findings cannot be generalised to NGO-managed HIV programmes broadly.

© 2026 Kebede HK et al. *JAMA Network Open*.

Section B. Main analysis DHARMa diagnostics

eFigure 5. DHARMa Residual Diagnostics: Overall Negative Binomial Models

▶ **Source file(s):**

- *main_diag_hiv_diagnoses_combined.pdf*
- *main_diag_linkage_to_care_combined.pdf*
- *main_diag_art_initiation_combined.pdf*

© 2026 Kebede HK et al. *JAMA Network Open*.

A

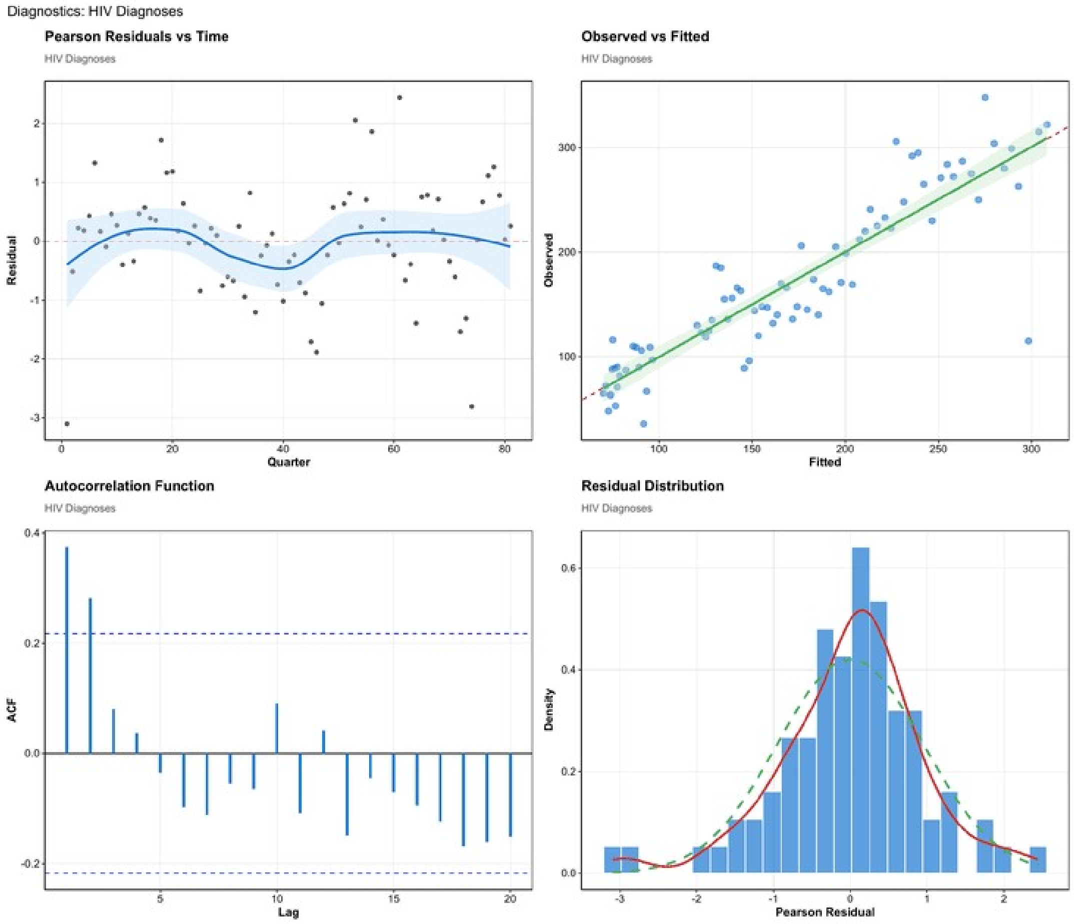

© 2026 Kebede HK et al. *JAMA Network Open*.

B

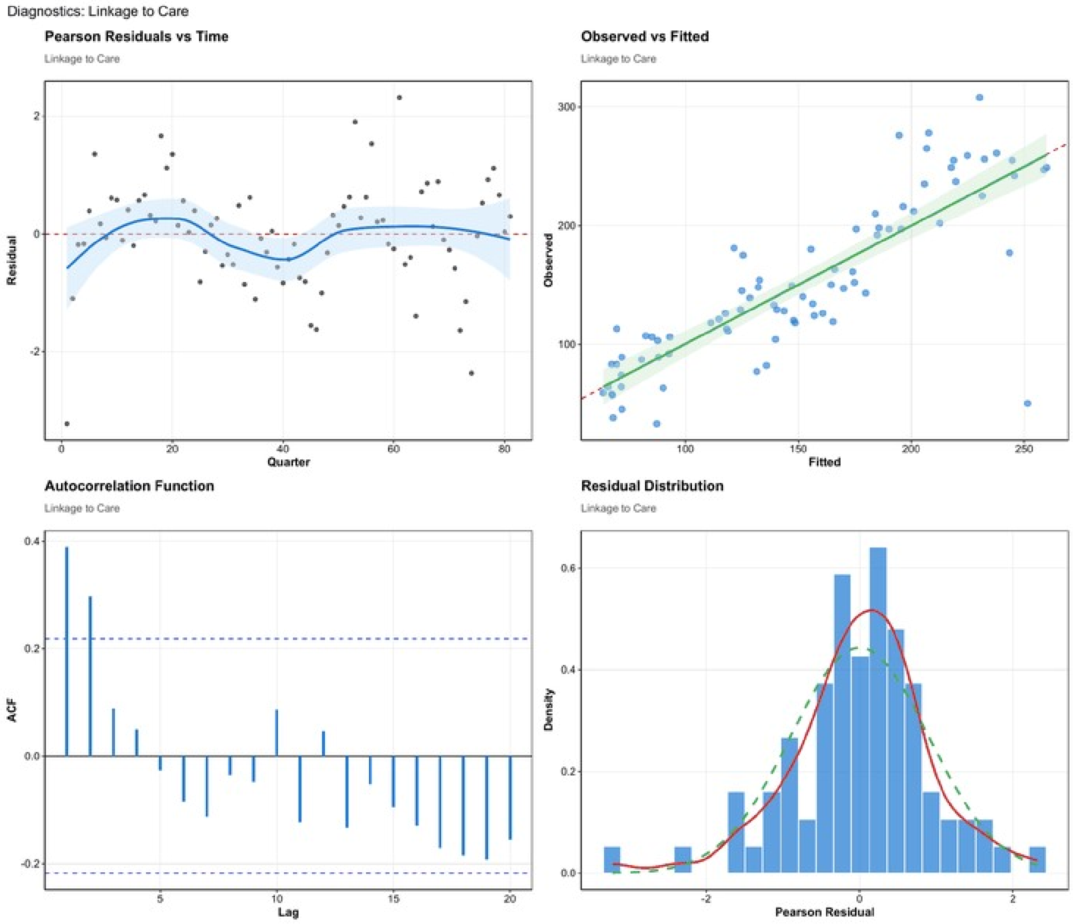

© 2026 Kebede HK et al. *JAMA Network Open*.

C

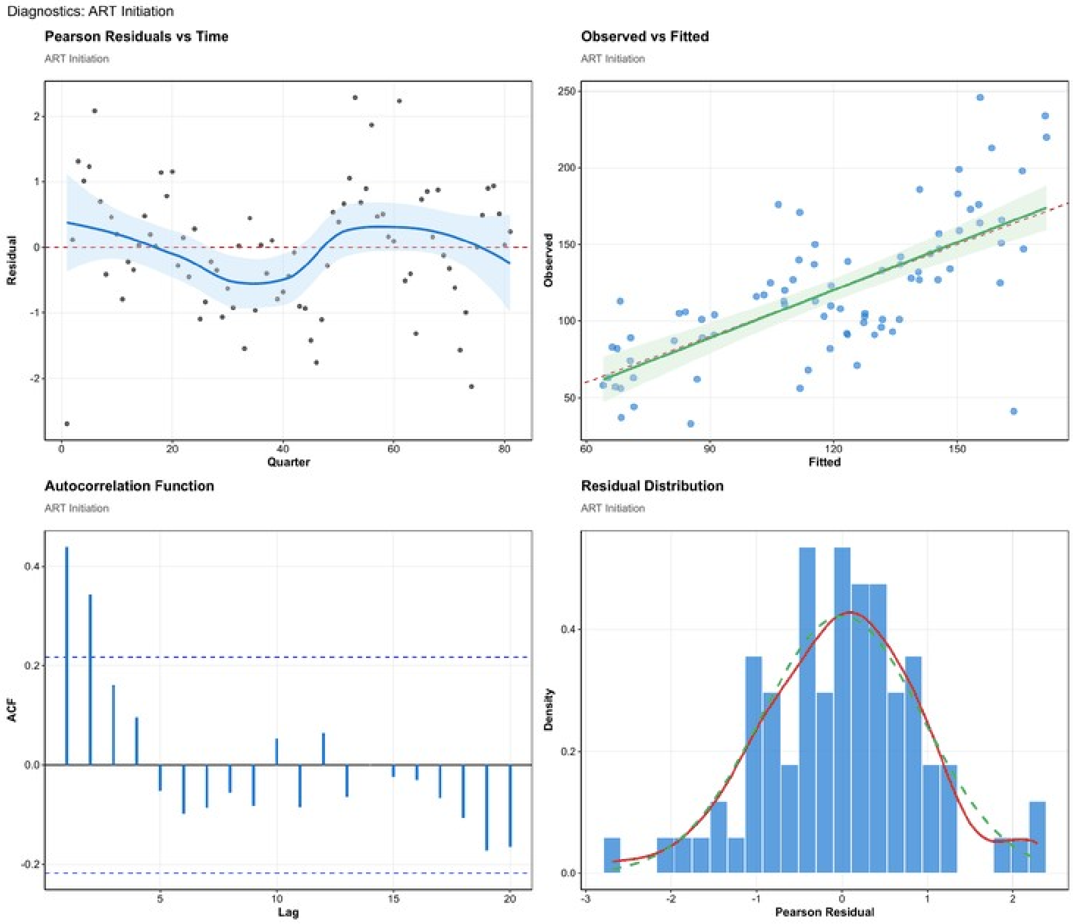

© 2026 Kebede HK et al. *JAMA Network Open*.

eFigure 5. Three-row composite of four-panel DHARMa diagnostic plots for the overall negative binomial segmented regression models for HIV diagnoses (top row), linkage to care (middle), and ART initiation (bottom). Within each row, panels show: (left-top) Pearson residuals vs time with LOESS smoother; (right-top) observed vs fitted with 1:1 reference line; (left-bottom) autocorrelation function with 95% Bartlett bands; (right-bottom) histogram of Pearson residuals with overlaid normal density. DHARMa formal tests (testDispersion(), testTemporalAutocorrelation()) returned non-significant P values for all three overall models, supporting model adequacy. Stratum-level DHARMa diagnostics for all 33 stratum-outcome combinations are available from the corresponding author on request.

© 2026 Kebede HK et al. *JAMA Network Open*.

Section C. ARIMA complementary analysis

eFigure 6. ARIMA Fitted vs Observed: With Intervention Regressors

▶ **Source file(s):**

- *Figure_ARIMA_Combined.pdf (preferred; pre-composed 3-panel)*
- *--- build composite from these 3 component files: ---*
- *arima_its_hiv_diagnoses.pdf*
- *arima_its_linkage_to_care.pdf*
- *arima_its_art_initiation.pdf*

© 2026 Kebede HK et al. *JAMA Network Open*.

A. HIV Diagnoses

ARIMA(0,1,0)(1,0,1)[4]; AICc=791.4

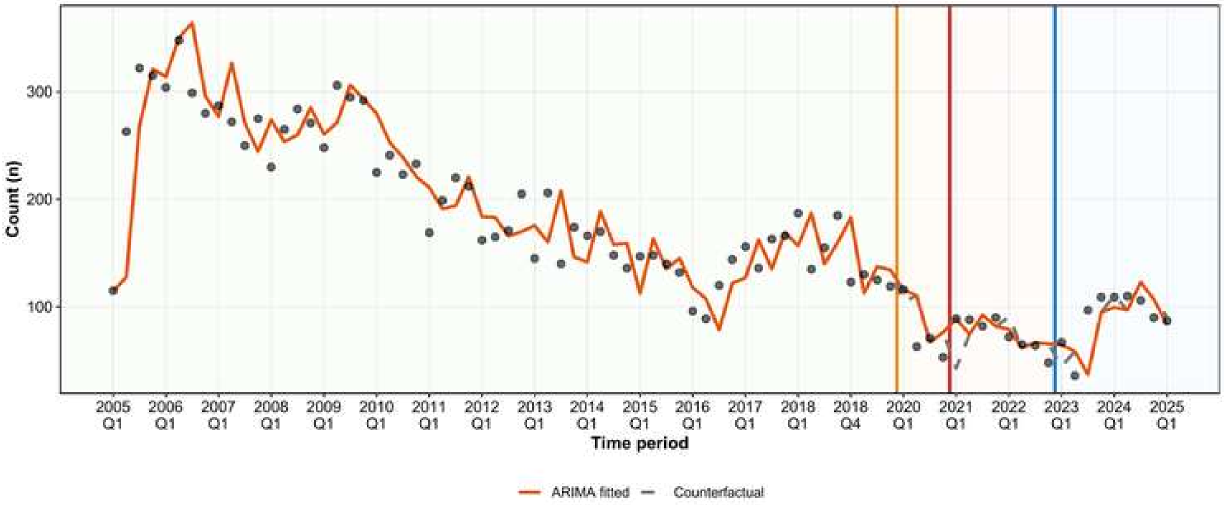

B. Linkage to Care

ARIMA(0,1,0)(1,0,1)[4]; AICc=792.3

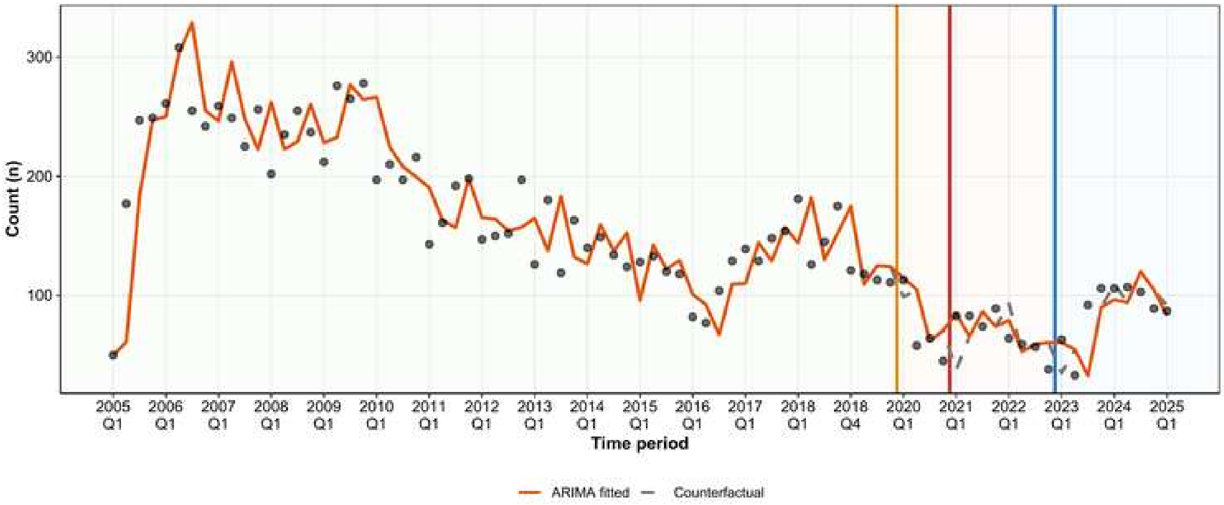

C. ART Initiation

ARIMA(1,1,1)(0,0,1)[4]; AICc=785.7

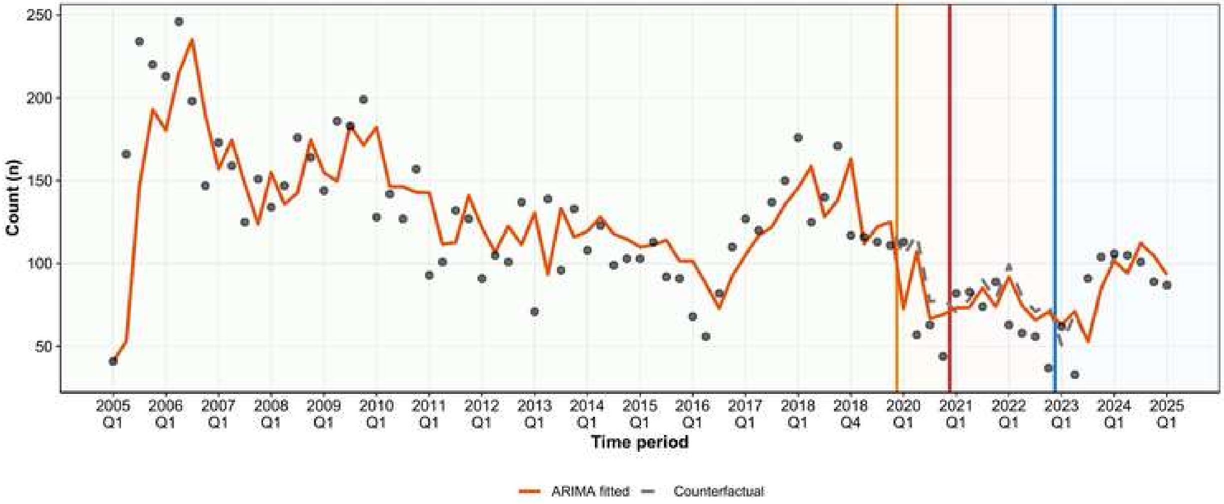

© 2026 Kebede HK et al. *JAMA Network Open*.

eFigure 6. Three-panel ARIMA-with-intervention-regressors fits for the three cascade outcomes. Solid orange line: ARIMA fitted values; dashed gray line: counterfactual (intervention regressors set to 0); black points: observed quarterly counts. ARIMA orders selected by auto.arima(stepwise = FALSE, approximation = FALSE): HIV diagnoses ARIMA(0,1,0)(1,0,1)[4], AICc = 791.4; linkage ARIMA(0,1,0)(1,0,1)[4], AICc = 792.3; ART initiation ARIMA(1,1,1)(0,0,1)[4], AICc = 785.7. All ARIMA intervention coefficients had the SAME DIRECTION as the negative binomial segmented regression IRRs (eTable 6). Larger ARIMA P values reflect standard errors that fully account for residual autocorrelation through the AR/MA structure.

© 2026 Kebede HK et al. *JAMA Network Open*.

eFigure 7. ARIMA Residual Diagnostics: Residuals Over Time and ACF

▶ **Source file(s):**

- *arima_diag_hiv_diagnoses.pdf (2-subpanel: residuals + ACF)*
- *arima_diag_linkage_to_care.pdf (2-subpanel: residuals + ACF)*
- *arima_diag_art_initiation.pdf (2-subpanel: residuals + ACF)*

© 2026 Kebede HK et al. *JAMA Network Open*.

A

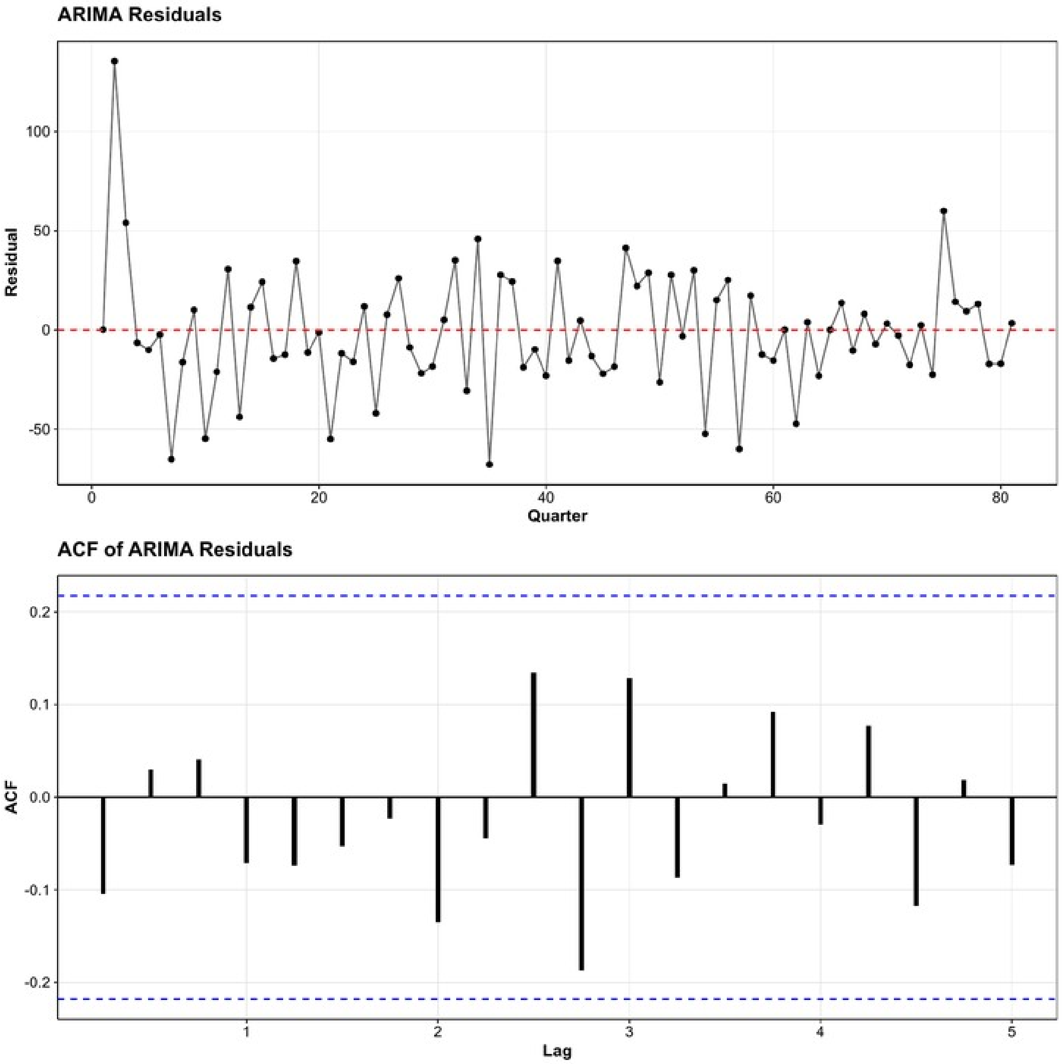

© 2026 Kebede HK et al. *JAMA Network Open*.

B

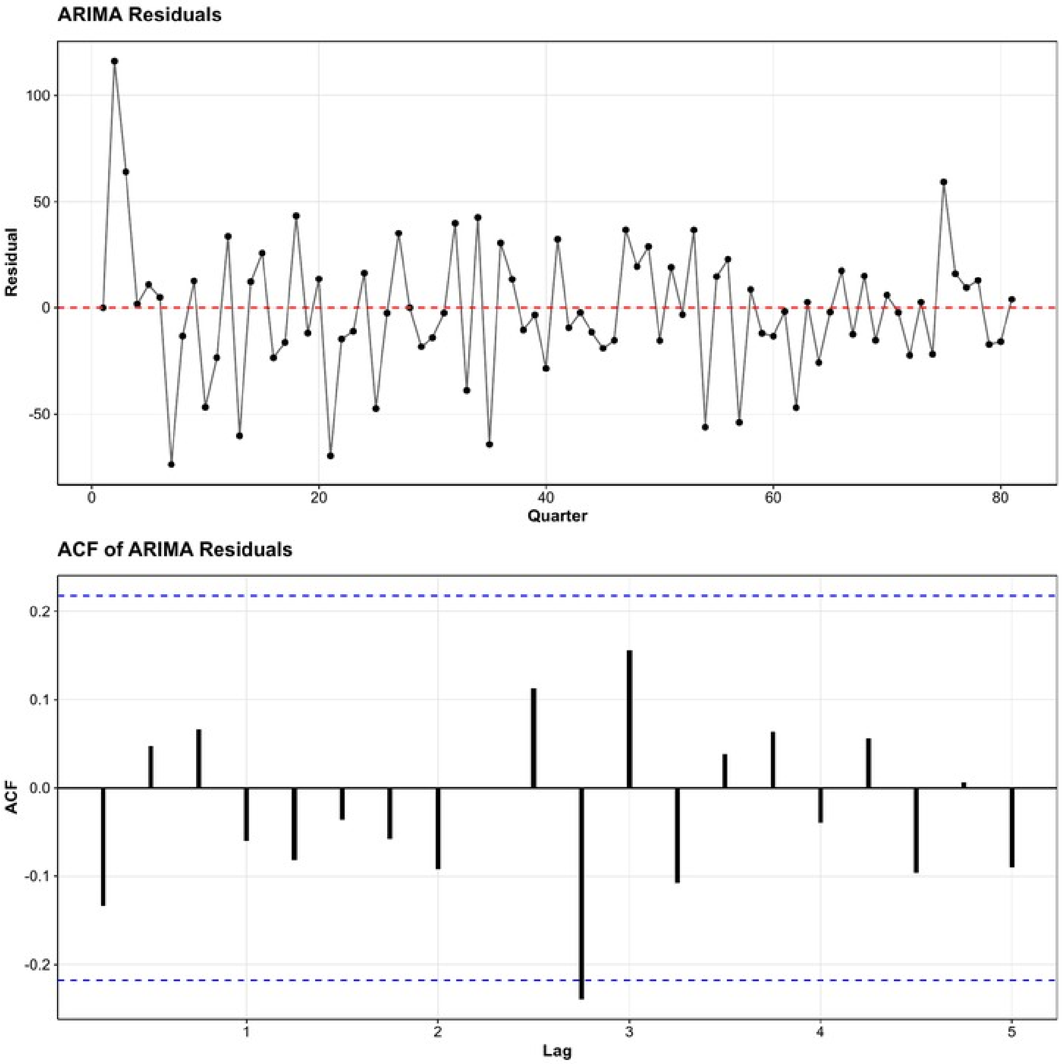

© 2026 Kebede HK et al. *JAMA Network Open*.

C

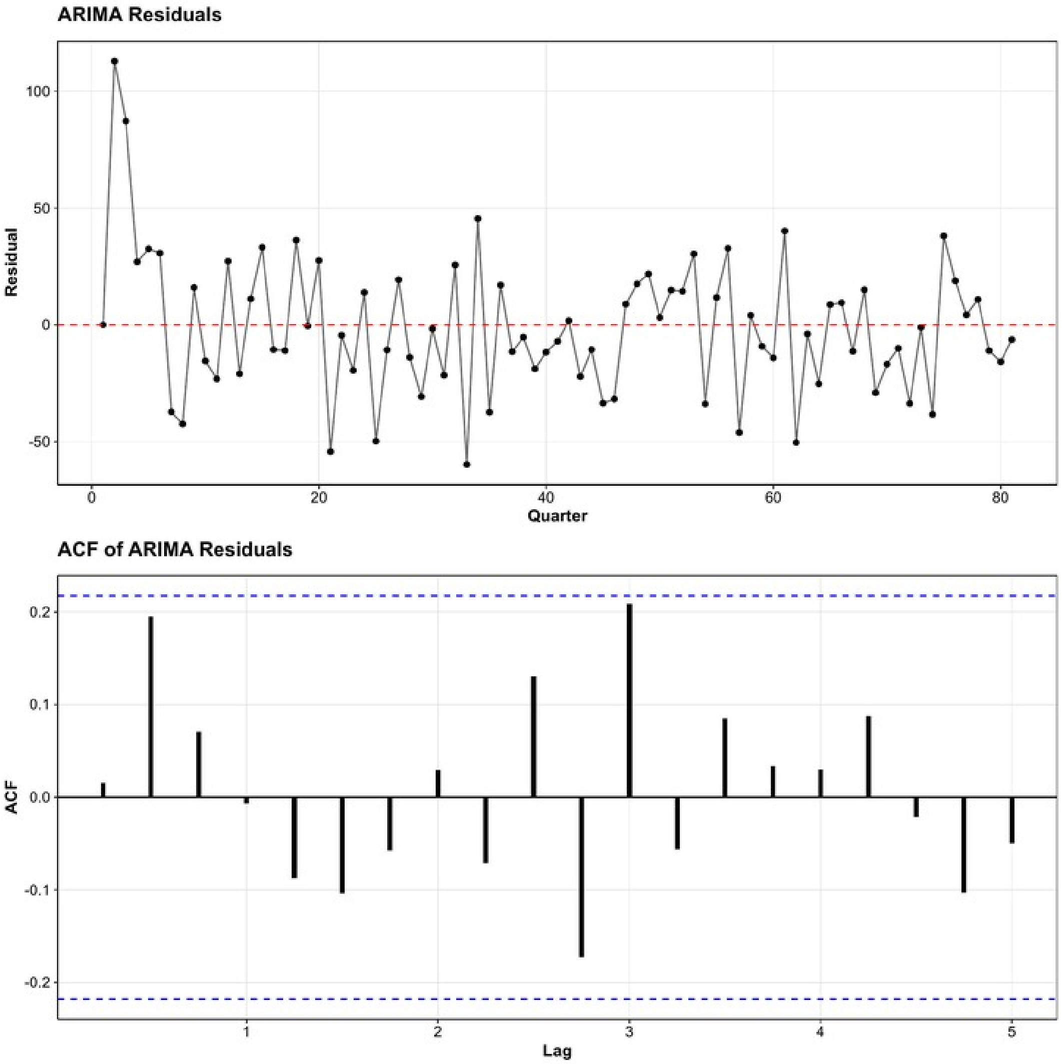

© 2026 Kebede HK et al. *JAMA Network Open*.

eFigure 7. ARIMA residual diagnostics for the three primary outcomes. For each outcome the upper subpanel shows residuals over time (any systematic trend would suggest model misspecification) and the lower subpanel shows the autocorrelation function with 95% Bartlett bands at $\pm 1.96/\sqrt{n}$. No autocorrelation peaks meaningfully exceed the bands, supporting the adequacy of the selected ARIMA orders. Together with the segmented regression DHARMa diagnostics in eFigure 5, these plots indicate that residual temporal structure has been adequately captured.

© 2026 Kebede HK et al. *JAMA Network Open*.

Section D. Sensitivity analyses (overall cascade)

eFigure 8. Sensitivity Analysis 1 (No Seasonal Harmonics), Overall

▶ **Source file(s):**

- *sens1_no_season_its_hiv_diagnoses_count.pdf*
- *sens1_no_season_its_linkage_to_care_count.pdf*
- *sens1_no_season_its_art_initiation_count.pdf*

© 2026 Kebede HK et al. *JAMA Network Open*.

A

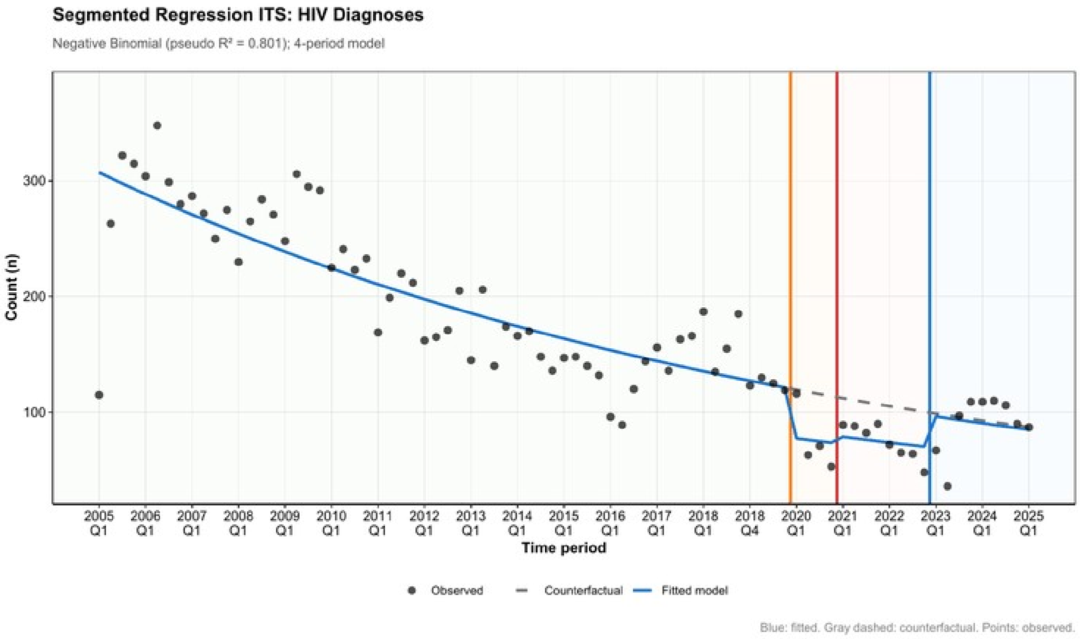

© 2026 Kebede HK et al. *JAMA Network Open*.

B

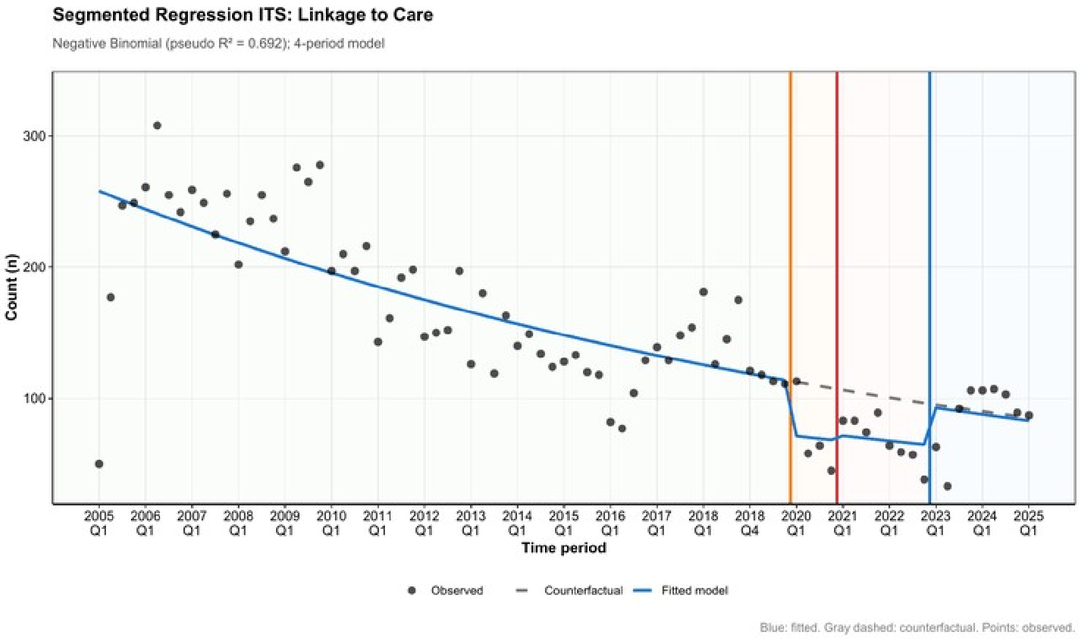

© 2026 Kebede HK et al. *JAMA Network Open*.

C

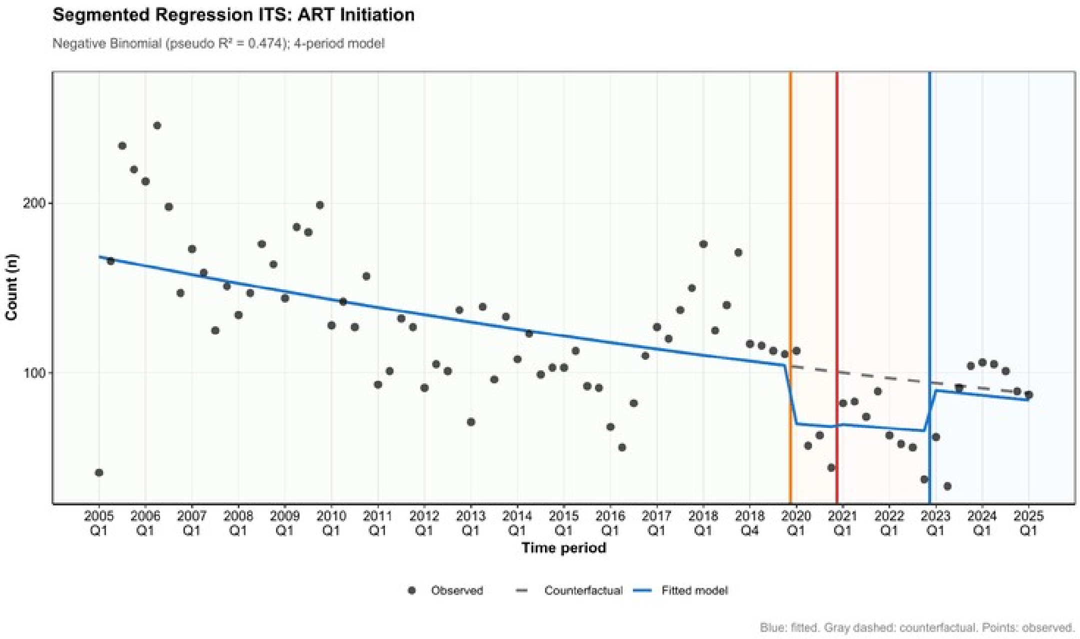

© 2026 Kebede HK et al. *JAMA Network Open*.

eFigure 8. Three-panel composite of negative binomial segmented regression fits with the sin and cos seasonal harmonic terms removed, for the three primary outcomes (overall, not stratified). Pandemic and war IRRs change by less than 2% from the main analysis (eTable 7), indicating that the seasonality assumption does not drive the headline findings. Direction and statistical significance of all three intervention effects are preserved.

© 2026 Kebede HK et al. *JAMA Network Open*.

eFigure 9. Sensitivity Analysis 2 (Full Slope-Change Specification), Overall

▶ **Source file(s):**

- *sens2_full_slopes_its_hiv_diagnoses_count.pdf*
- *sens2_full_slopes_its_linkage_to_care_count.pdf*
- *sens2_full_slopes_its_art_initiation_count.pdf*

© 2026 Kebede HK et al. *JAMA Network Open*.

A

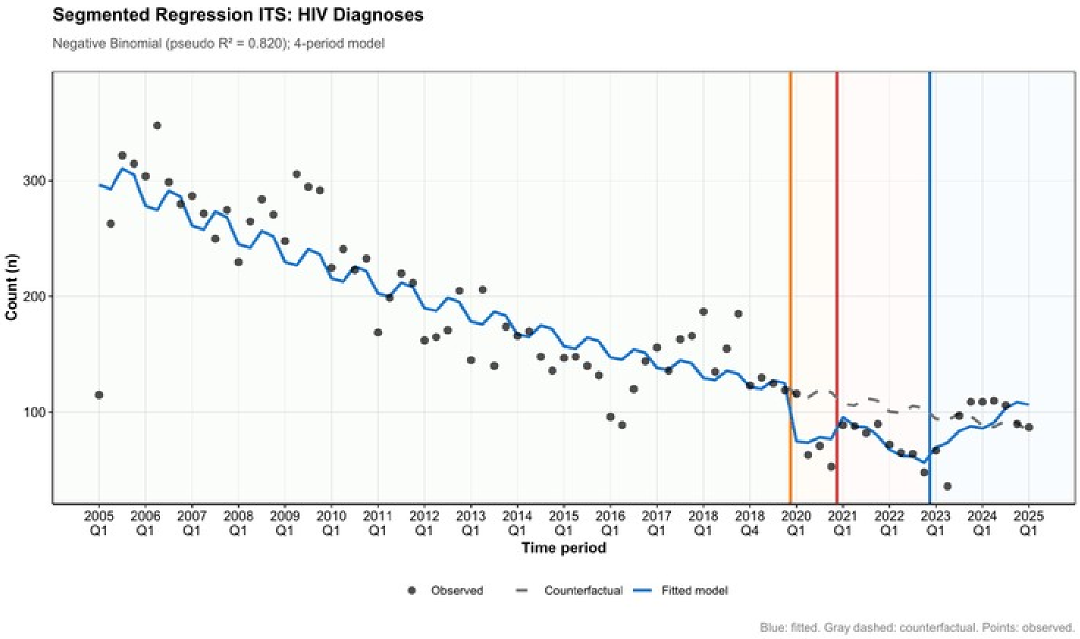

© 2026 Kebede HK et al. *JAMA Network Open*.

B

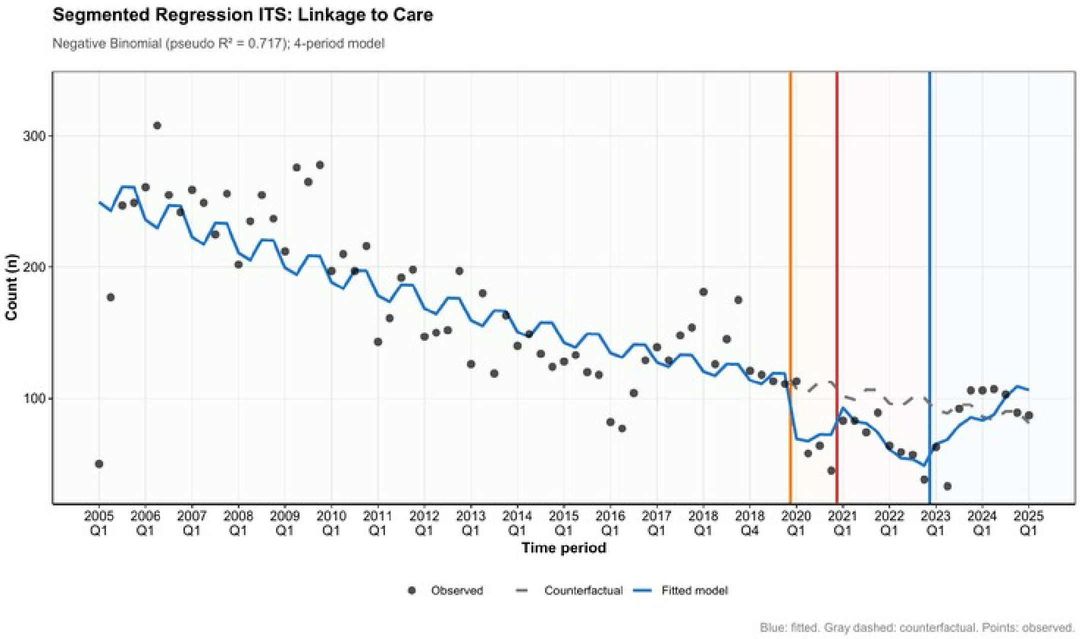

© 2026 Kebede HK et al. *JAMA Network Open*.

C

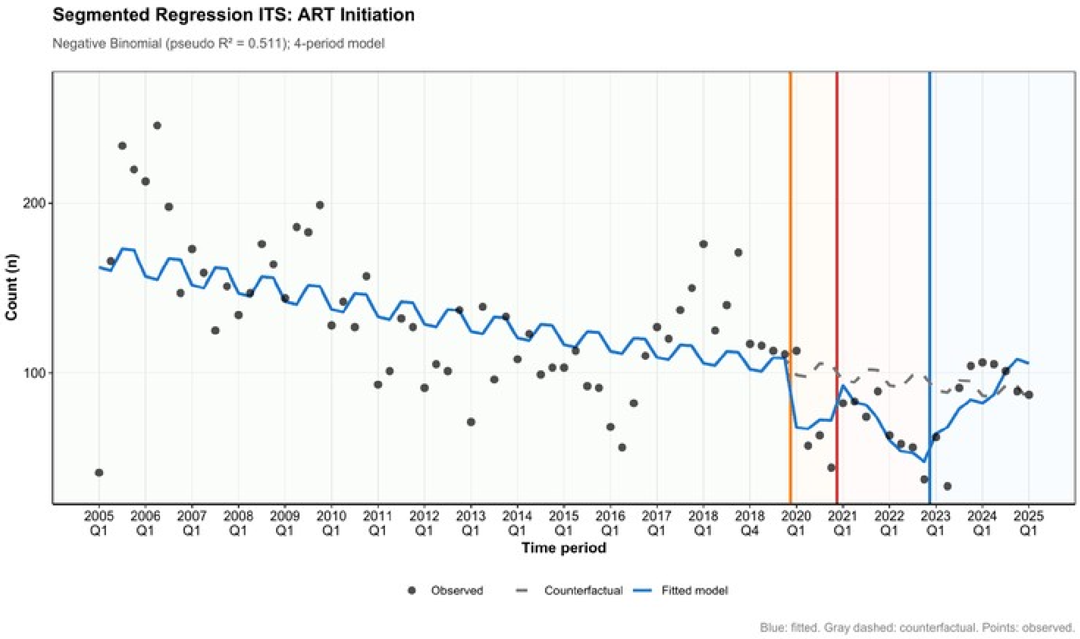

© 2026 Kebede HK et al. *JAMA Network Open*.

eFigure 9. Three-panel composite of fits with time-after-war and time-after-postwar slope-change terms added to the standard level-change specification, for the three primary outcomes. War-period level-change IRRs become non-significant in this specification (eTable 8), but the slope-change terms are also non-significant. This pattern is a known artefact of joint level-and-slope segmented regression with moderate within-period sample sizes and indicates that the level-change-only specification (main analysis) captures the dominant signal. Within-period slopes did not differ detectably from the pre-war secular trend once level shifts were modelled.

© 2026 Kebede HK et al. *JAMA Network Open*.

eFigure 10. Sensitivity Analysis 3 (Alternative War Onset Q4 2020), Overall

▶ **Source file(s):**

- *sens3_alt_onset_its_hiv_diagnoses_count.pdf*
- *sens3_alt_onset_its_linkage_to_care_count.pdf*
- *sens3_alt_onset_its_art_initiation_count.pdf*

© 2026 Kebede HK et al. *JAMA Network Open*.

A

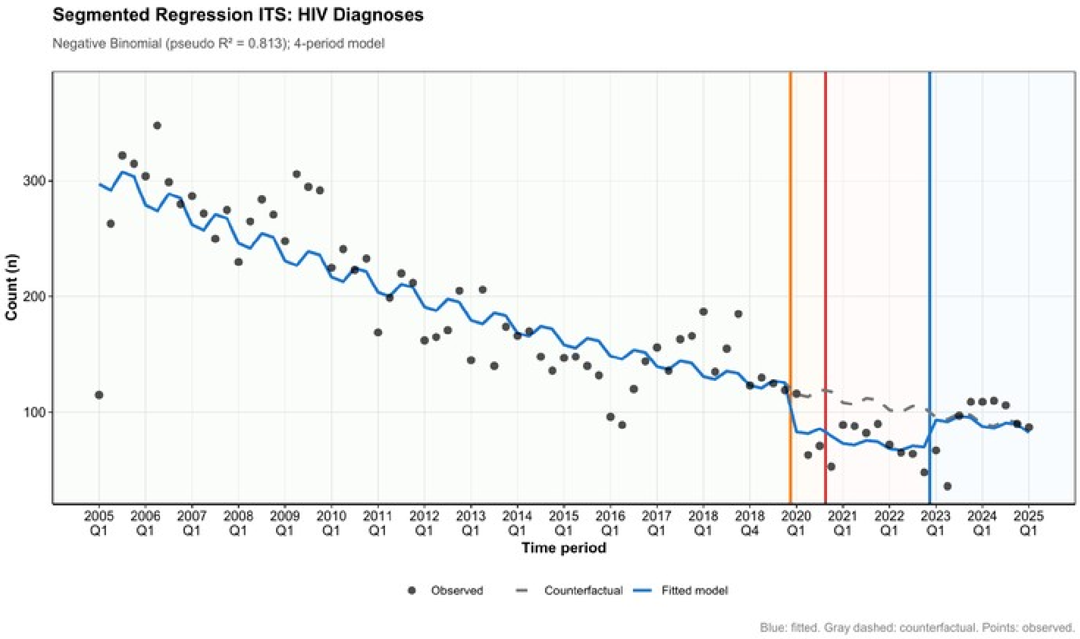

© 2026 Kebede HK et al. *JAMA Network Open*.

B

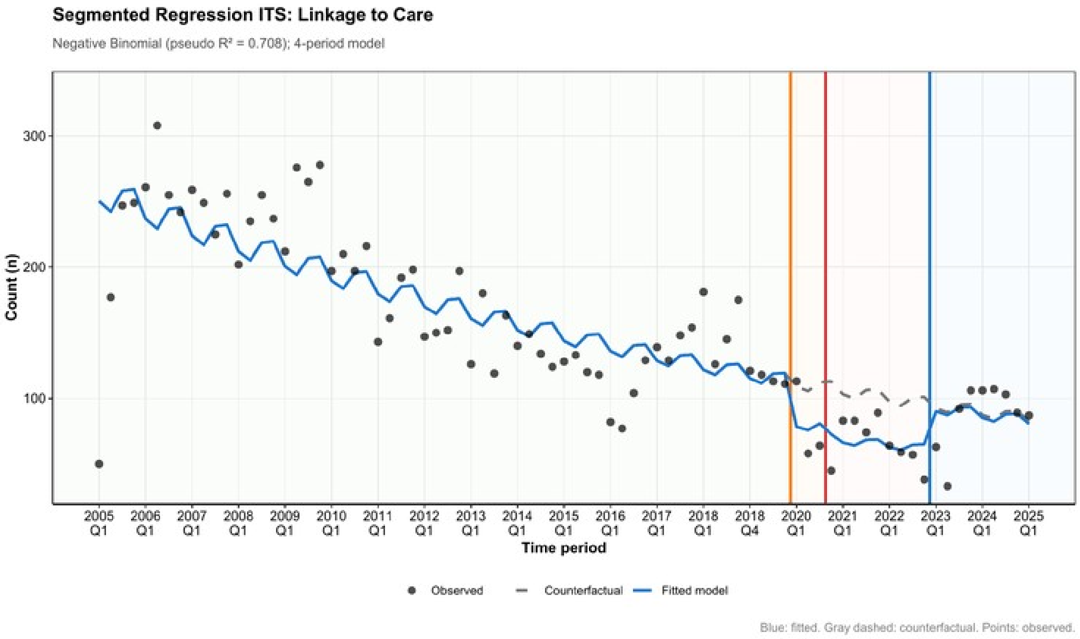

© 2026 Kebede HK et al. *JAMA Network Open*.

C

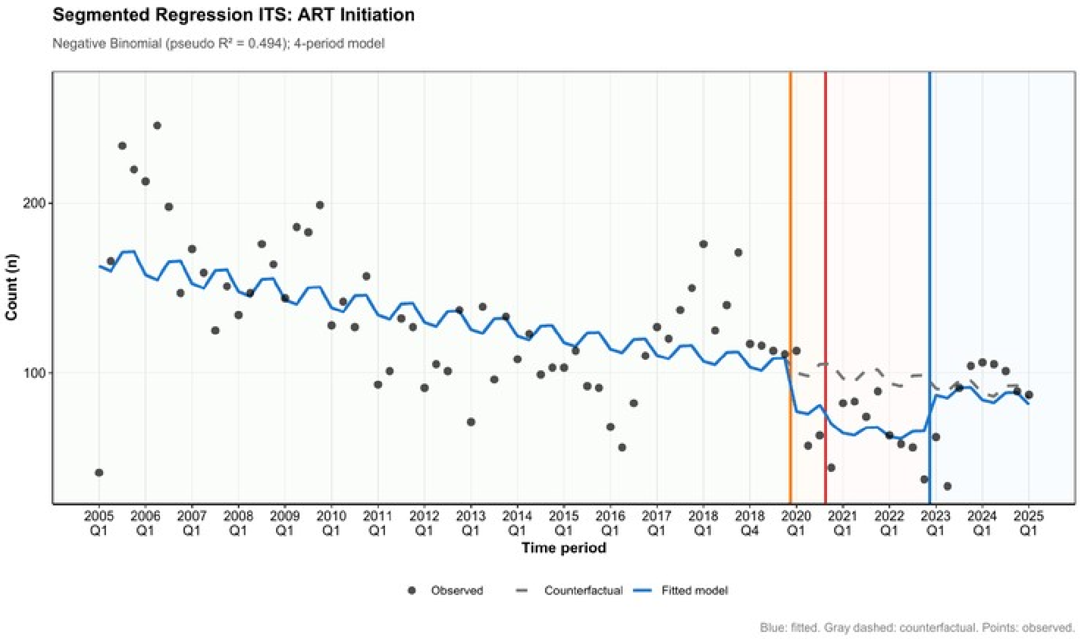

© 2026 Kebede HK et al. *JAMA Network Open*.

eFigure 10. Three-panel composite of fits with Q4 2020 reassigned from the pandemic period to the war period (war = 9 quarters; pandemic = 3 quarters), for the three primary outcomes. War-period IRRs are slightly more negative than the main analysis (e.g., HIV diagnoses 0.67 vs 0.71); pandemic IRRs are slightly attenuated, consistent with absorbing some war-period signal into the pandemic period when the date split is later. Direction and qualitative interpretation are unchanged from the main analysis (eTable 9).

© 2026 Kebede HK et al. *JAMA Network Open*.

eFigure 11. Sensitivity Analysis 4 (Prewar Baseline Restricted to 2015+), Overall

▶ **Source file(s):**

- *sens4_recent_its_hiv_diagnoses_count.pdf*
- *sens4_recent_its_linkage_to_care_count.pdf*
- *sens4_recent_its_art_initiation_count.pdf*

© 2026 Kebede HK et al. *JAMA Network Open*.

A

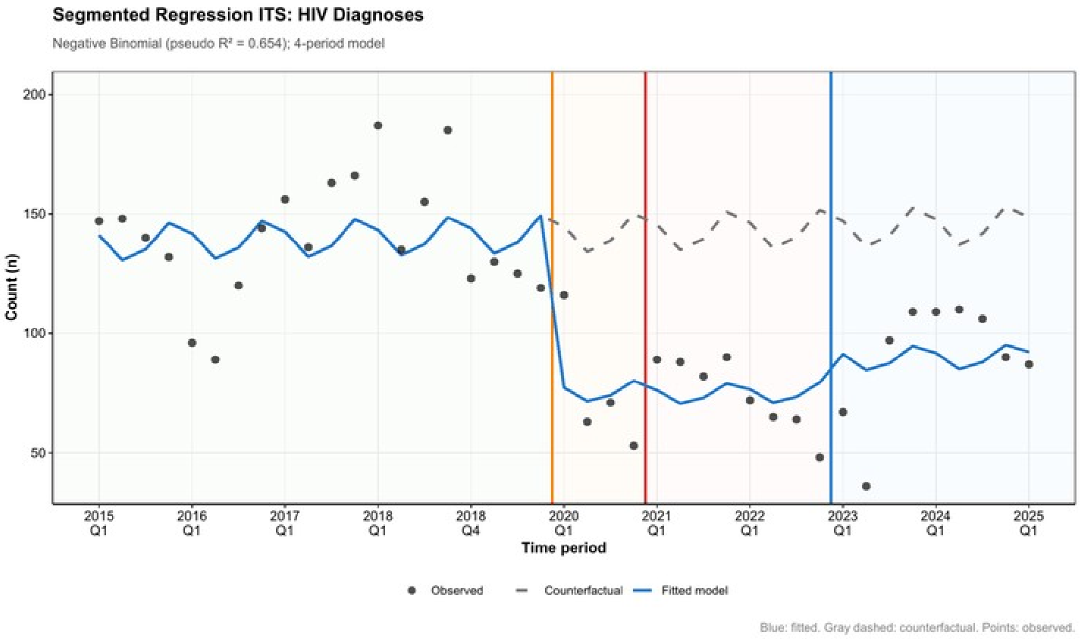

© 2026 Kebede HK et al. *JAMA Network Open*.

B

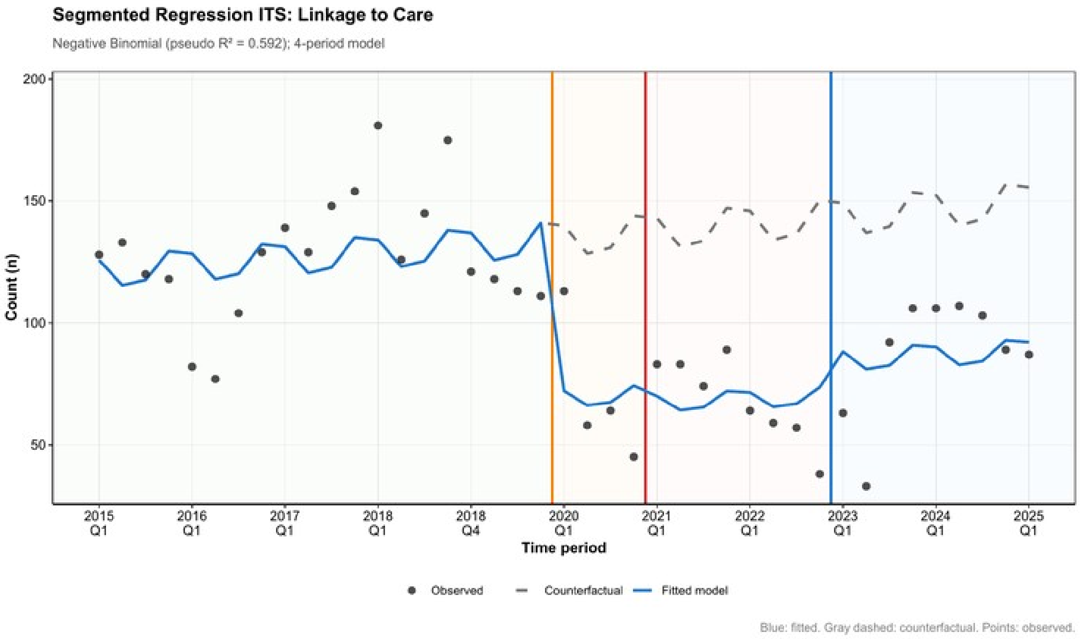

© 2026 Kebede HK et al. *JAMA Network Open*.

C

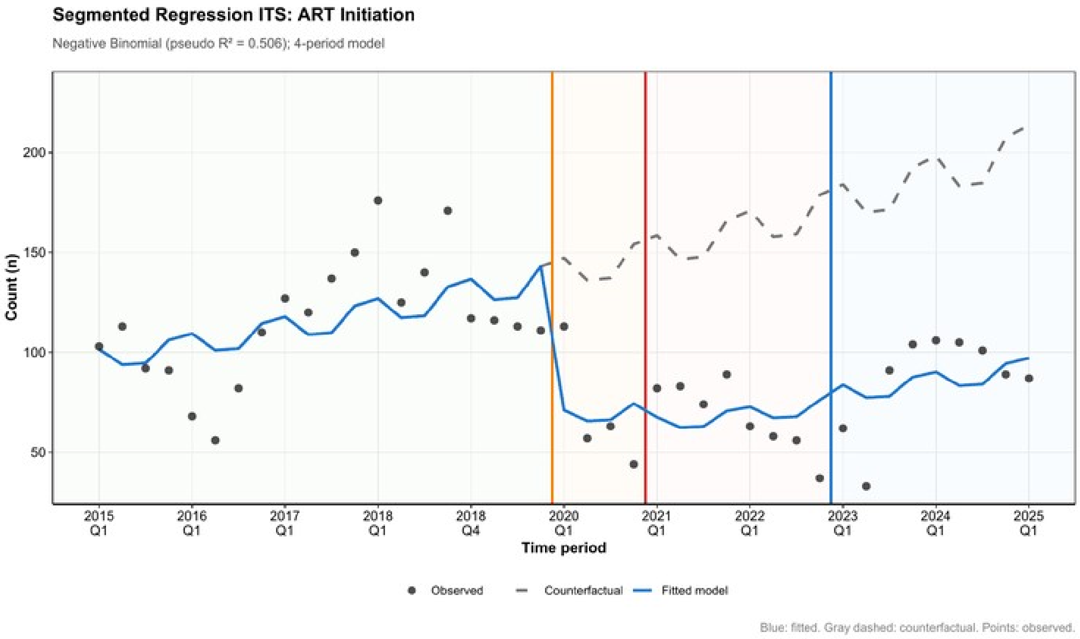

© 2026 Kebede HK et al. *JAMA Network Open*.

eFigure 11. Three-panel composite of fits with the pre-war baseline restricted to Q1 2015 – Q4 2019 (post test-and-treat era when Ethiopia adopted universal ART eligibility), for the three primary outcomes. War-period IRRs are notably more negative than the main analysis (e.g., HIV diagnoses 0.52 vs 0.71), reflecting the higher recent baseline against which the war-period reduction is measured. The direction of all effects is unchanged, and the qualitative conclusion that the war period was associated with substantial reductions in cascade entry is strengthened (eTable 10).

© 2026 Kebede HK et al. *JAMA Network Open*.

Section E. Sensitivity DHARMa diagnostics

eFigure 12. Sensitivity DHARMa Overview: 4 Sensitivities × 3 Outcomes

▶ **Source file(s):**

- *sens1_no_season_diag_hiv_diagnoses_combined.pdf*
- *sens1_no_season_diag_linkage_to_care_combined.pdf*
- *sens1_no_season_diag_art_initiation_combined.pdf*
- *sens2_full_slopes_diag_hiv_diagnoses_combined.pdf*
- *sens2_full_slopes_diag_linkage_to_care_combined.pdf*
- *sens2_full_slopes_diag_art_initiation_combined.pdf*
- *sens3_alt_onset_diag_hiv_diagnoses_combined.pdf*
- *sens3_alt_onset_diag_linkage_to_care_combined.pdf*
- *sens3_alt_onset_diag_art_initiation_combined.pdf*
- *sens4_recent_diag_hiv_diagnoses_combined.pdf*
- *sens4_recent_diag_linkage_to_care_combined.pdf*
- *sens4_recent_diag_art_initiation_combined.pdf*

© 2026 Kebede HK et al. *JAMA Network Open*.

A

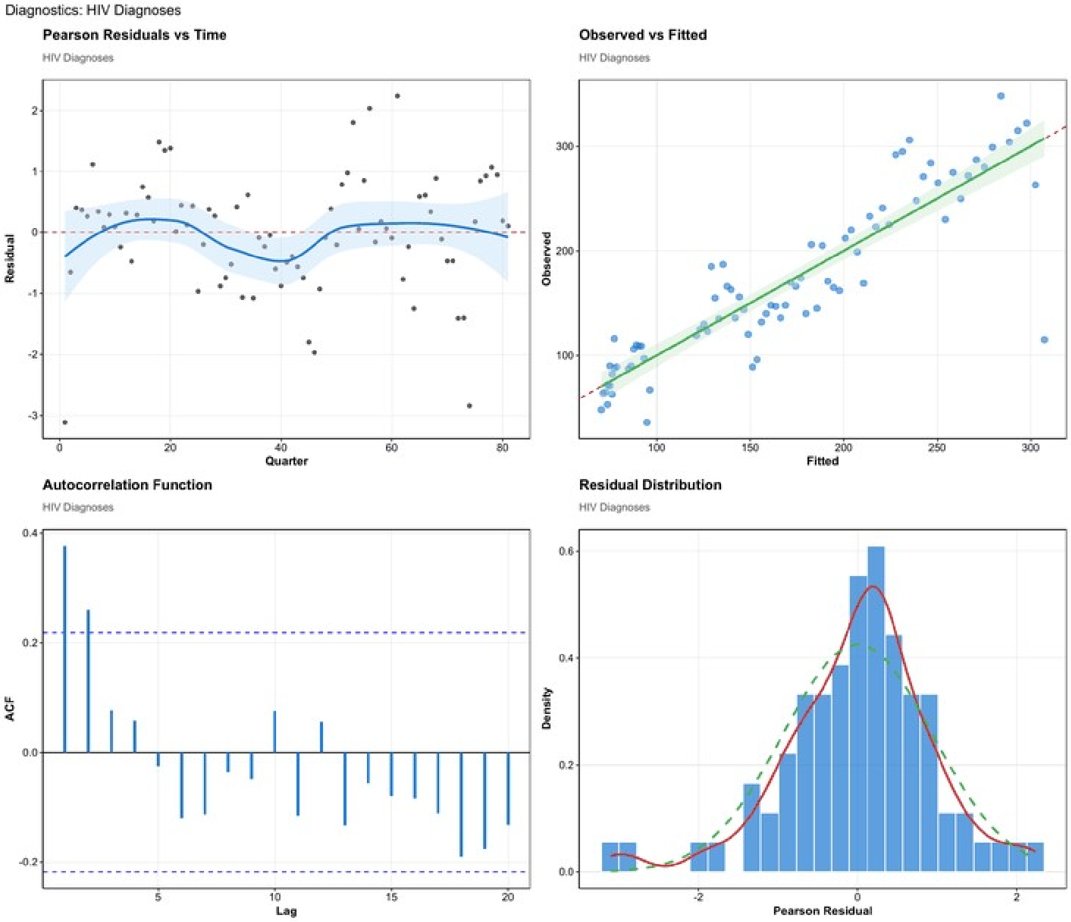

© 2026 Kebede HK et al. *JAMA Network Open*.

B

Diagnostics: Linkage to Care

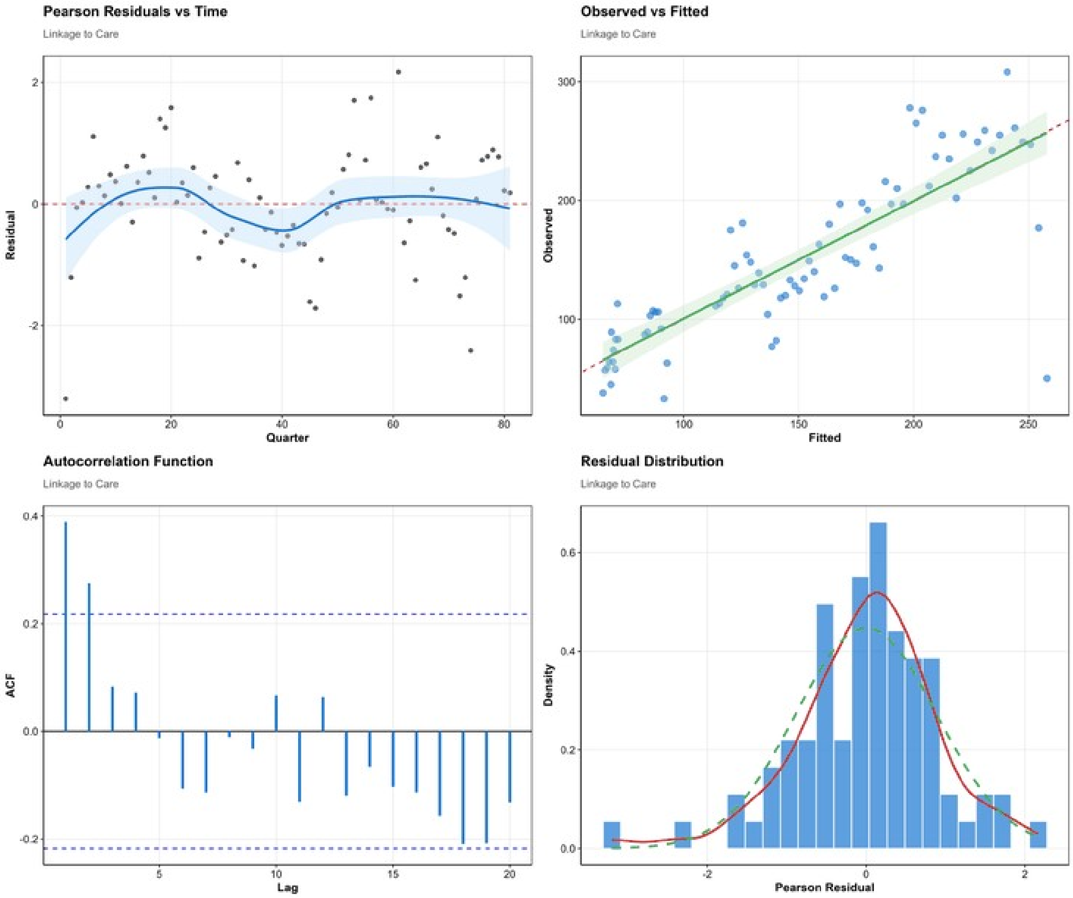

© 2026 Kebede HK et al. *JAMA Network Open*.

C

Diagnostics: ART Initiation

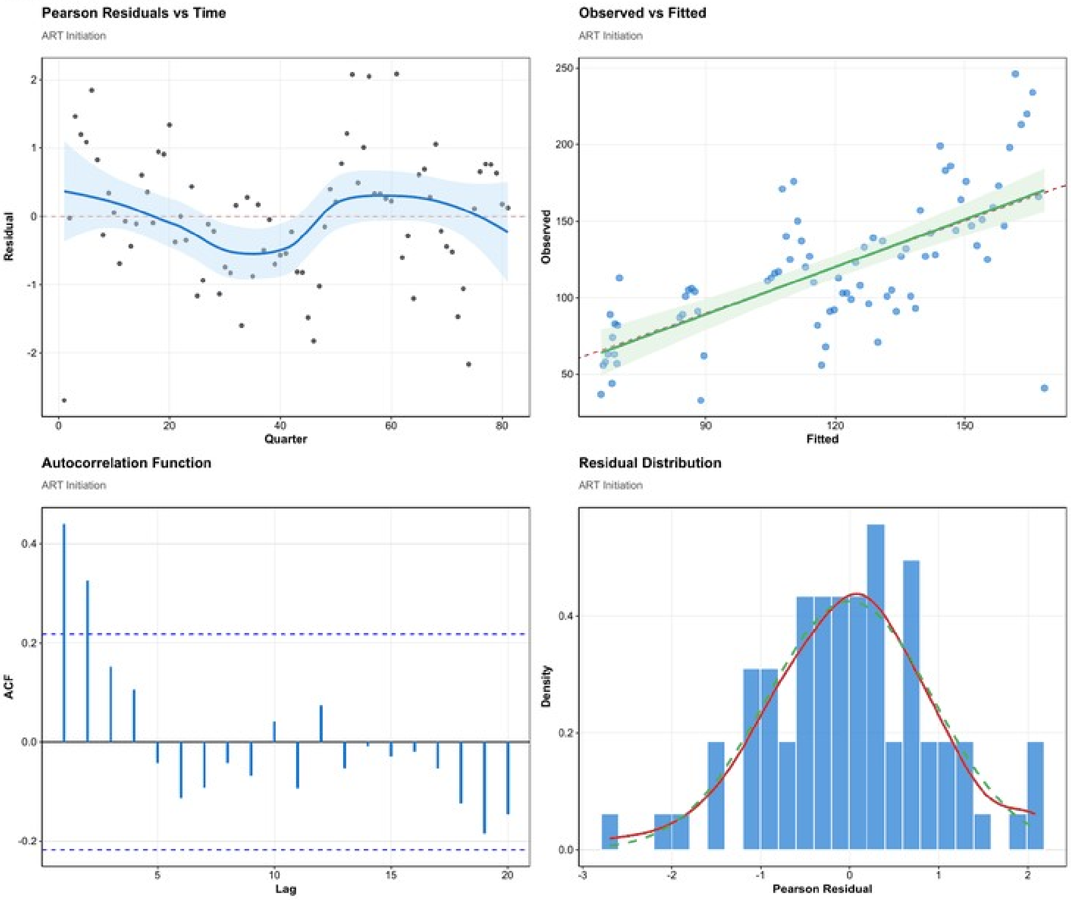

© 2026 Kebede HK et al. *JAMA Network Open*.

D

Diagnostics: HIV Diagnoses

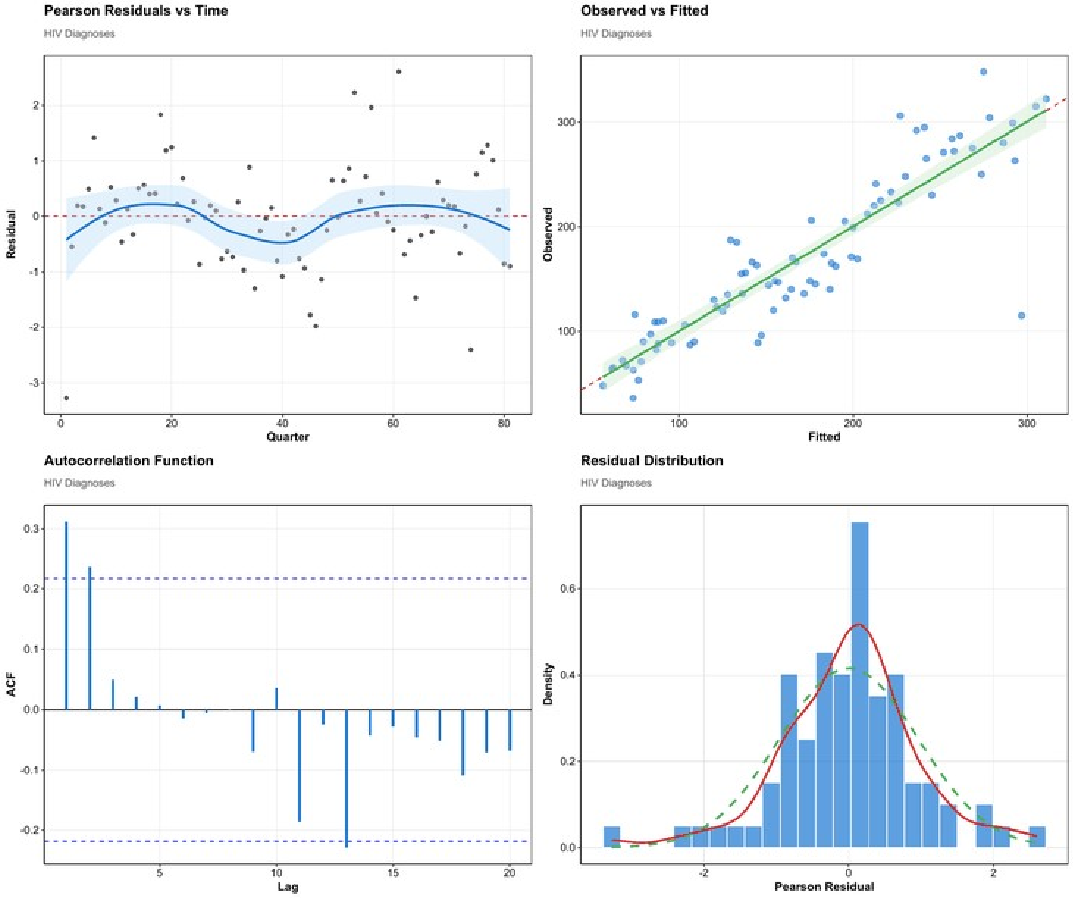

© 2026 Kebede HK et al. *JAMA Network Open*.

E

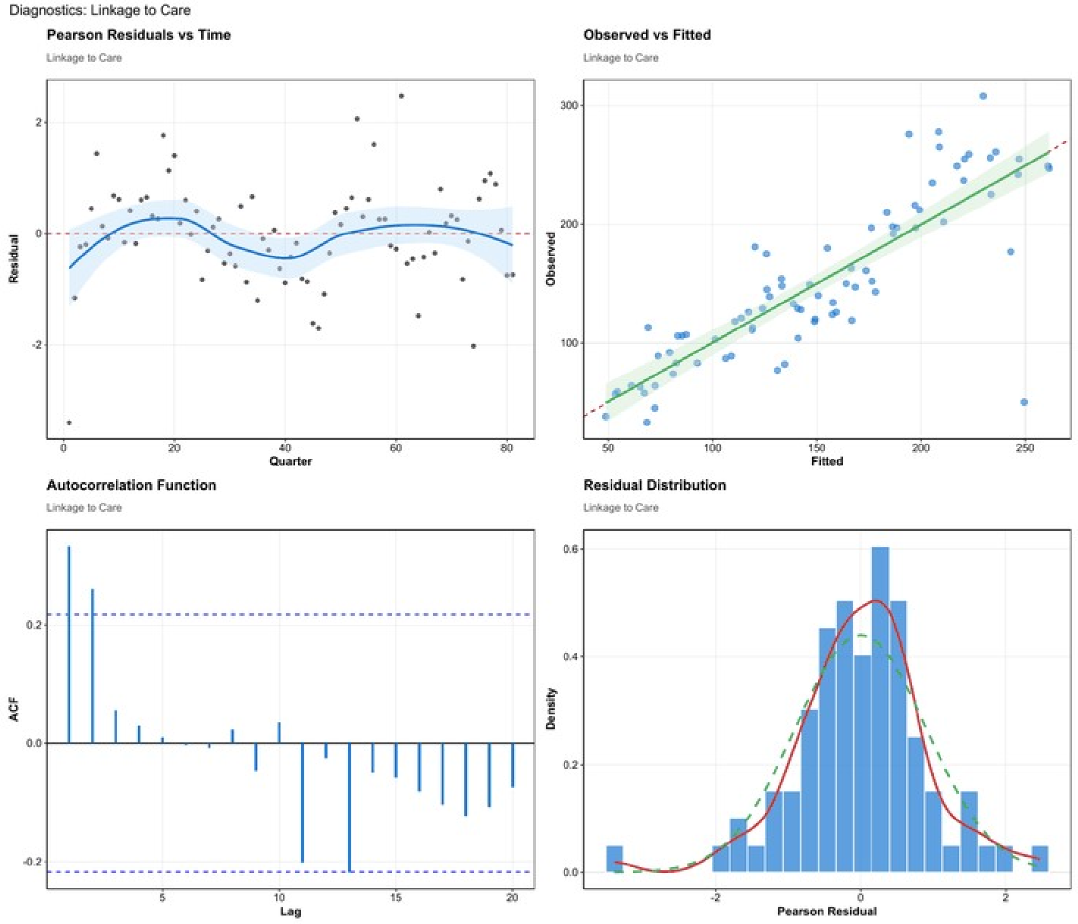

© 2026 Kebede HK et al. *JAMA Network Open*.

F

Diagnostics: ART Initiation

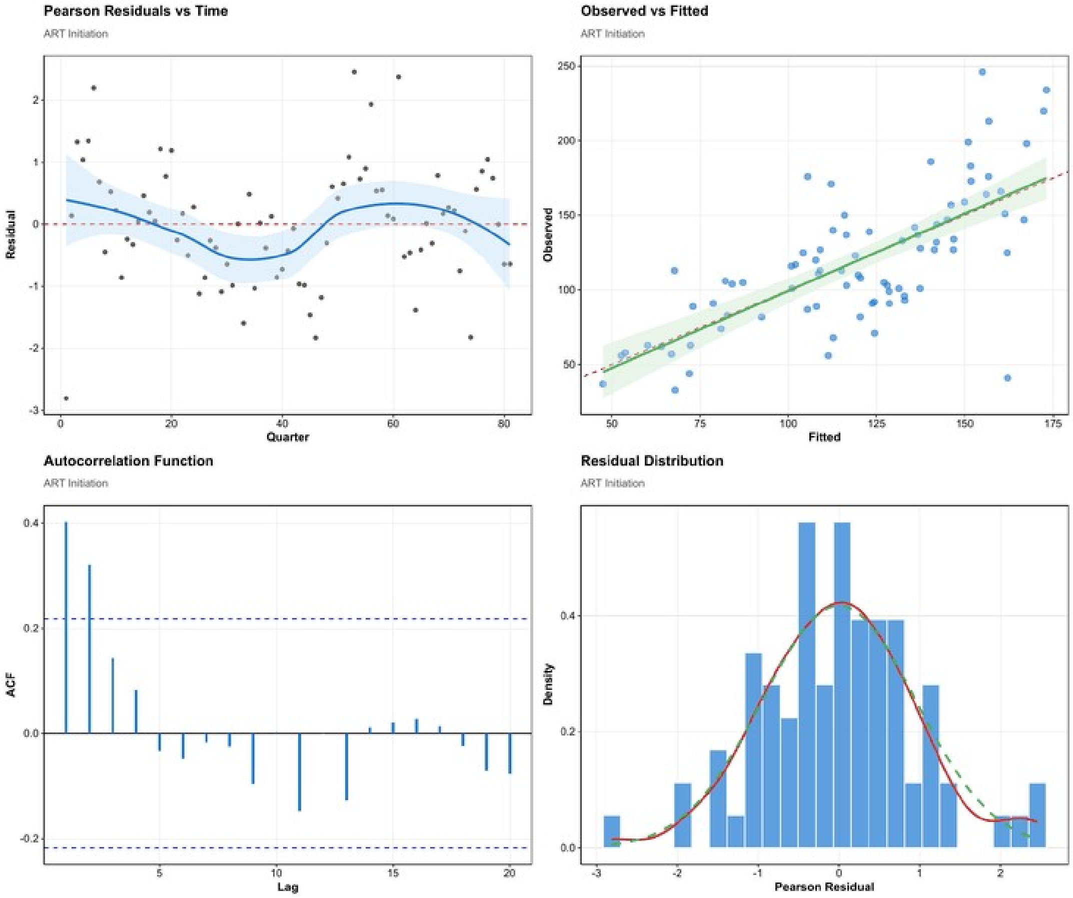

© 2026 Kebede HK et al. *JAMA Network Open*.

G

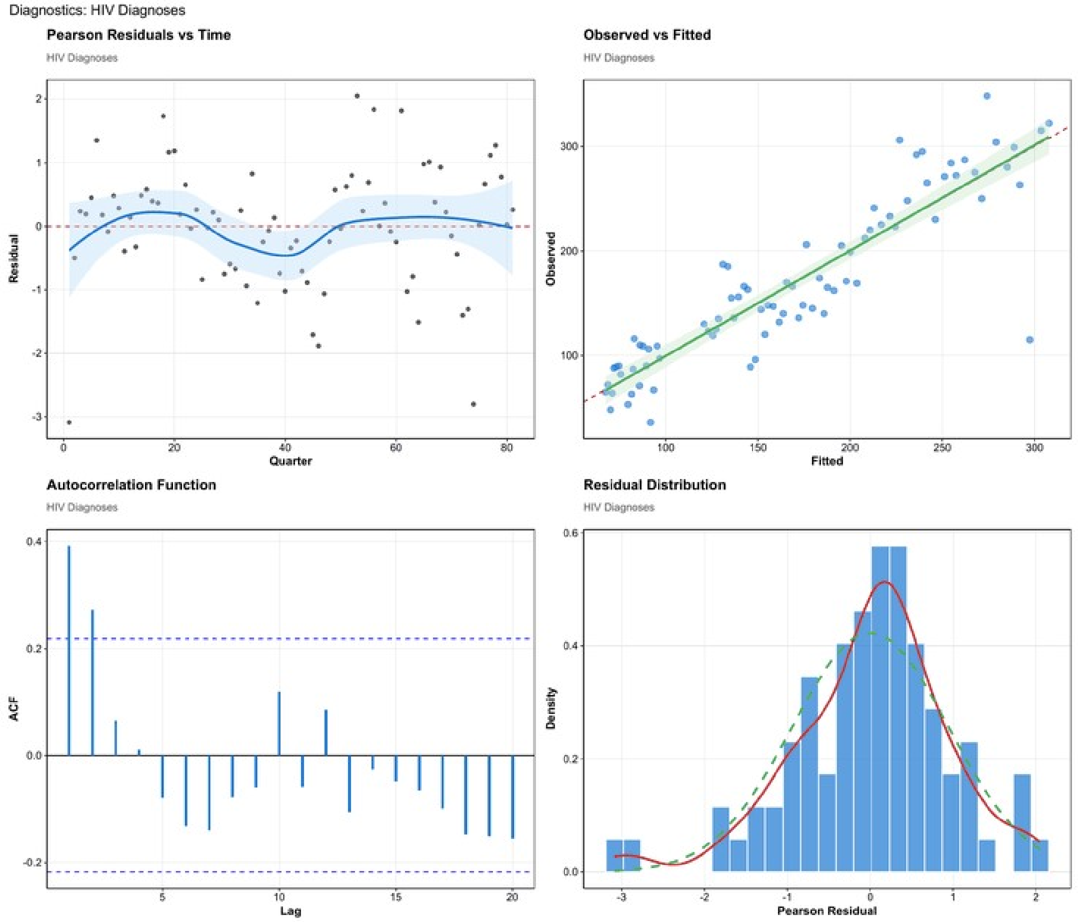

© 2026 Kebede HK et al. *JAMA Network Open*.

H

Diagnostics: Linkage to Care

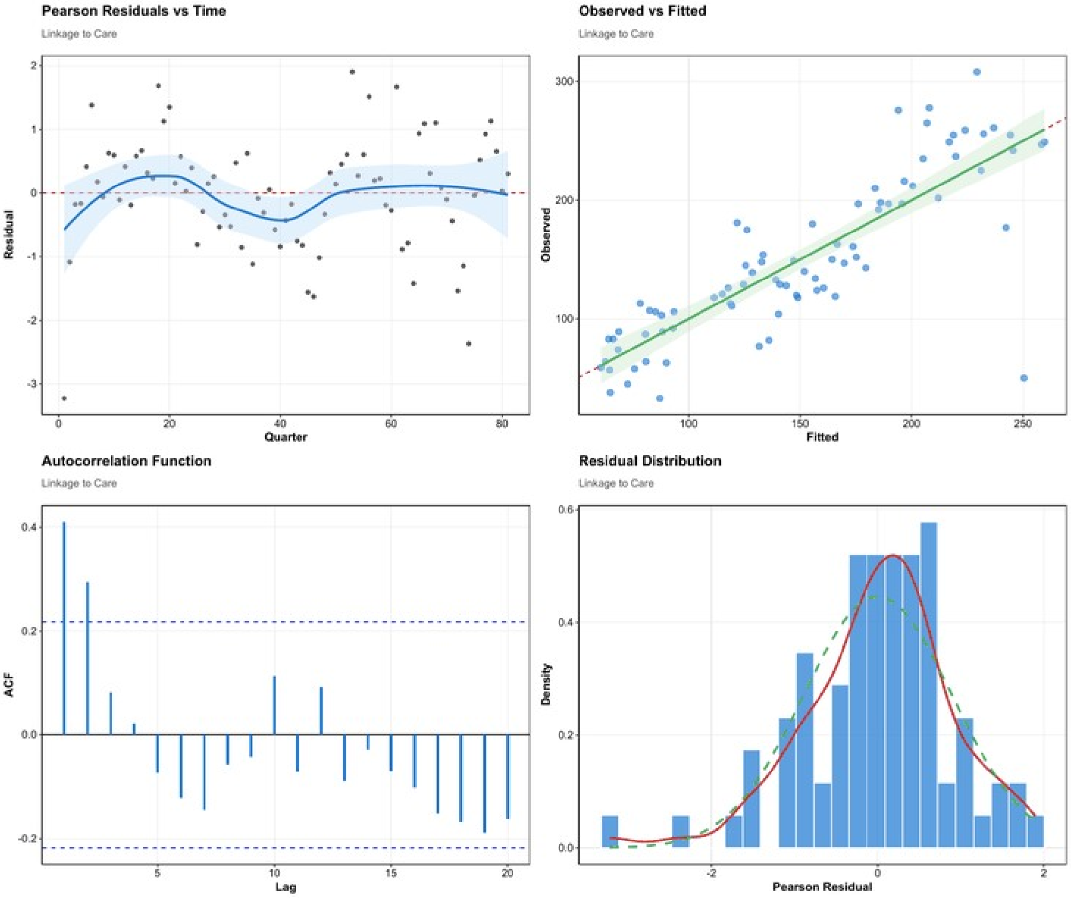

© 2026 Kebede HK et al. *JAMA Network Open*.

I

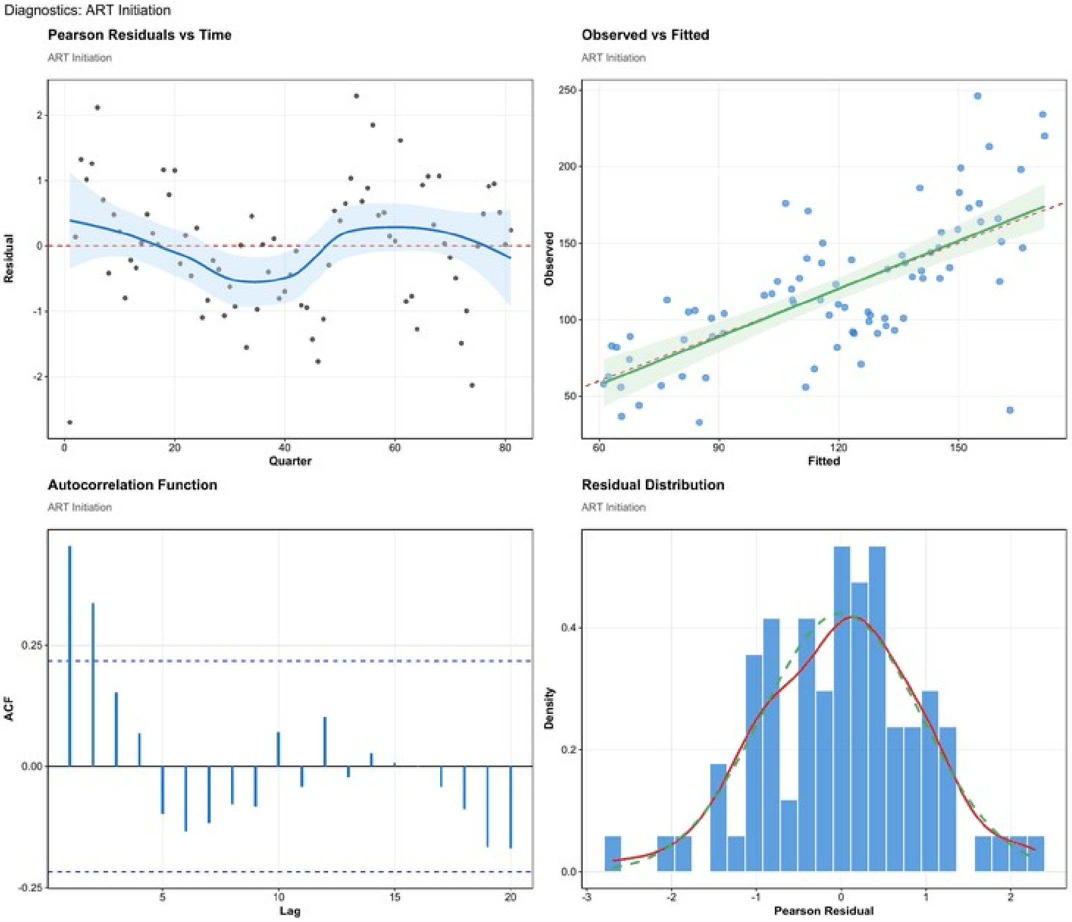

© 2026 Kebede HK et al. *JAMA Network Open*.

J

Diagnostics: HIV Diagnoses

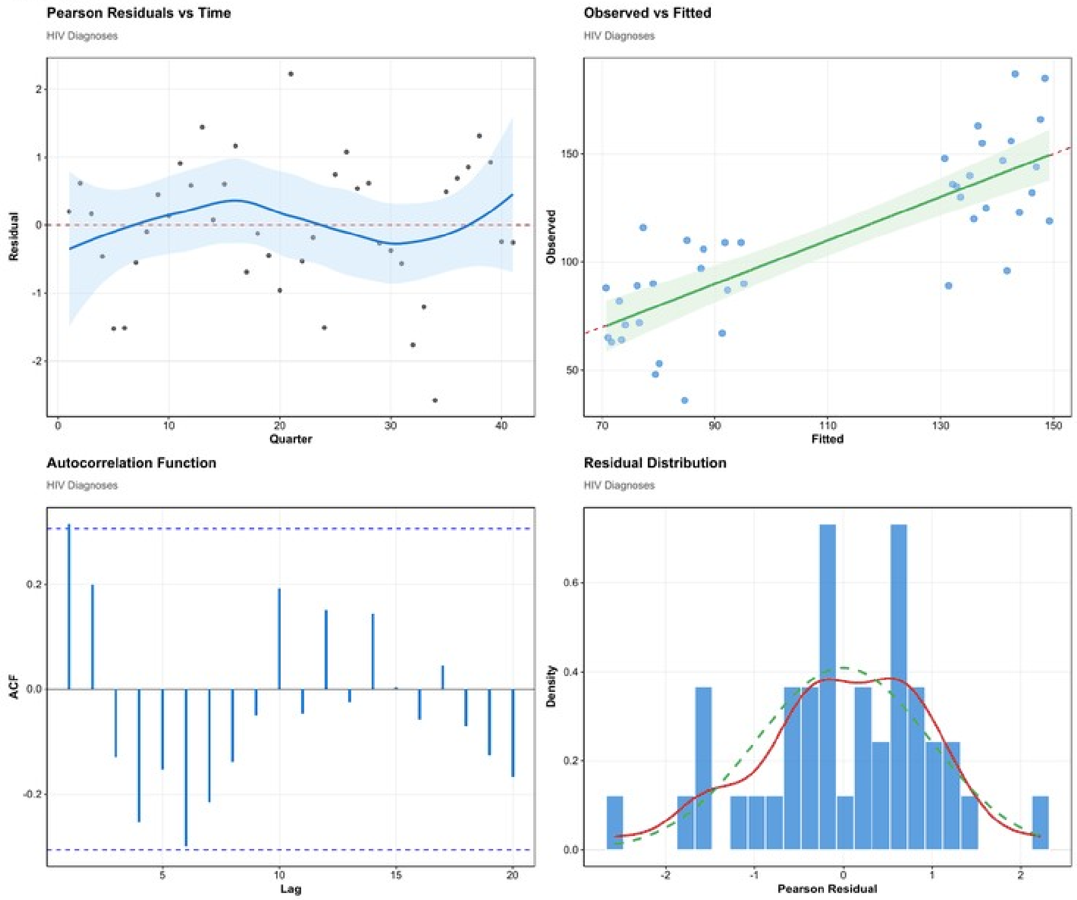

© 2026 Kebede HK et al. *JAMA Network Open*.

K

Diagnostics: Linkage to Care

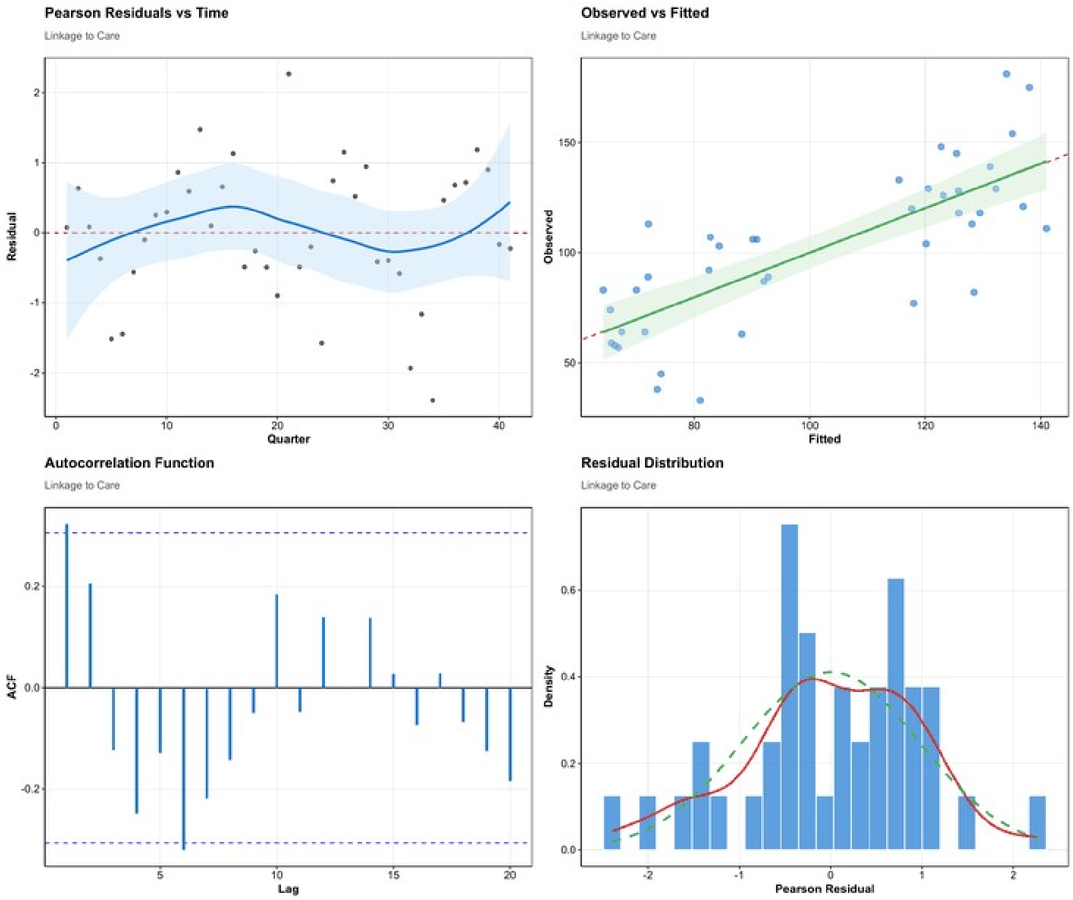

© 2026 Kebede HK et al. *JAMA Network Open*.

L

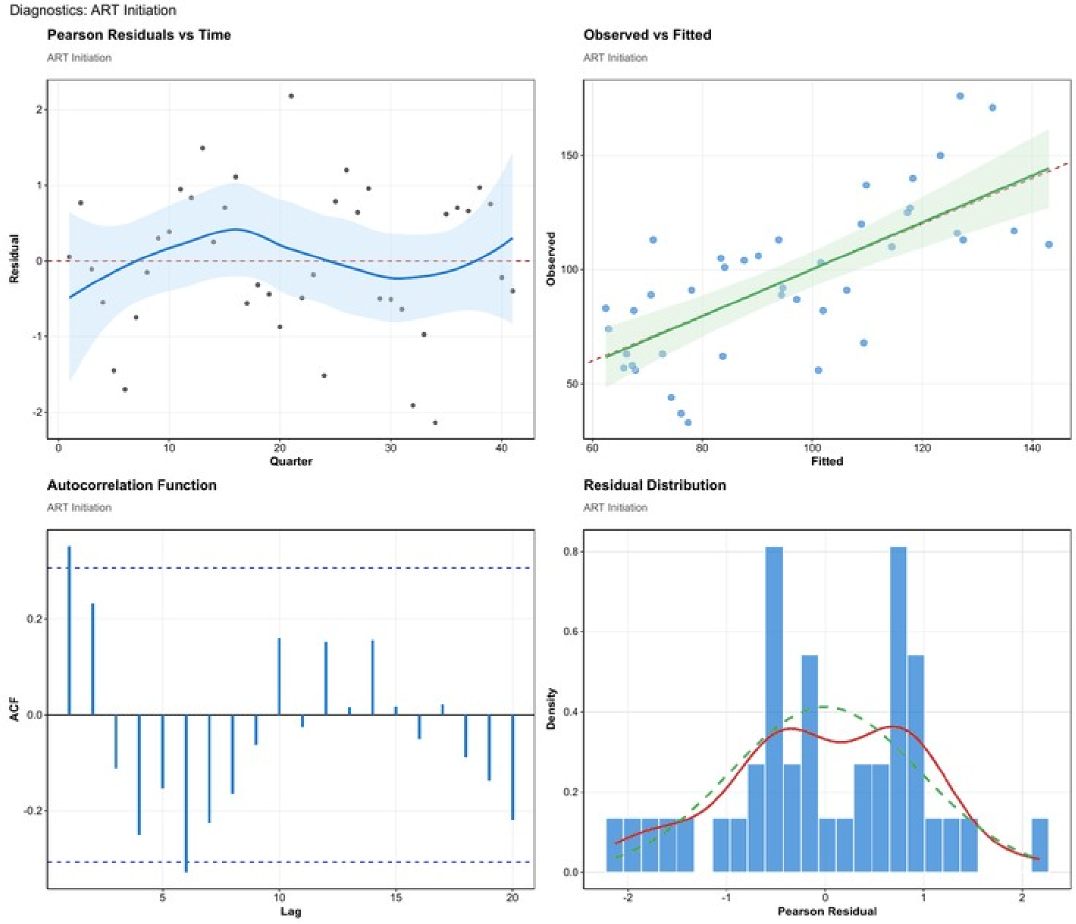

eFigure 12. Twelve-panel composite of DHARMa diagnostic summaries (Pearson residuals vs time + ACF + histogram) for the four sensitivity analyses across the three primary outcomes. Rows = sensitivity (Sens 1: no seasonality; Sens 2: full slope-change; Sens 3: alt onset Q4 2020; Sens 4: recent baseline 2015+); columns = HIV

© 2026 Kebede HK et al. *JAMA Network Open*.

diagnoses, linkage, ART initiation. None of the residual patterns suggest model misspecification under any of the sensitivity specifications, supporting the qualitative robustness of the headline findings to the modelling choices examined. Tabulated formal-test P values are reported in eTables 8–11 of the Supplementary Tables.

© 2026 Kebede HK et al. *JAMA Network Open*.

eMethods 3. *Detailed Methodology for the Count-Model Interrupted Time-Series Analysis*
Tigray War and HIV Care Cascade Entry in Mekelle, Ethiopia

Hafte Kahsay Kebede, BPharm, MSc; Hailay Abrha Gesesew, PhD; Lillian Mwanri, PhD; Paul Ward, PhD
Submitted to JAMA Network Open

Scope. This document expands the Methods section of the main manuscript with the level of detail typical of a methodologically focused supplement. The exposition focuses exclusively on the count-model interrupted time-series segmented regression analysis that produced the incidence rate ratios (IRRs) reported in Table 2 of the manuscript and in eTables 5–5a of the Supplementary Tables. Rate-based offset models are not addressed; the manuscript and supplementary materials focus exclusively on count-model results, consistent with the original manuscript's count-based design.

Contents

1. Study design and ethical considerations
2. Setting, participants, and facility selection
3. Data sources and extraction
4. Time frame and four-period structure
5. Outcome definitions
6. Stratification scheme
7. Statistical analysis: count-model segmented regression
8. Counterfactual projection and cumulative deficits
9. Model diagnostics
10. Sensitivity analyses
11. Complementary ARIMA analysis
12. Effect modification tests
13. Software, reproducibility, and reporting

© 2026 Kebede HK et al. *JAMA Network Open*.

1. Study design and ethical considerations

1.1 Study design

We conducted a retrospective interrupted time-series (ITS) analysis of routinely collected facility-level HIV care cascade data spanning 20 years and 81 quarters in Mekelle, Tigray, Ethiopia. ITS is a quasi-experimental design that estimates the change in an outcome's level and/or trend at the time of an interruption, using each unit as its own historical control. The design is well-suited for population-level evaluations when randomization is neither feasible nor ethical, as is the case for armed conflict and pandemic exposures.

The interruptions of interest were the COVID-19 pandemic (Q1–Q4 2020), the Tigray War (November 2020 – November 2022; analytically Q1 2021 – Q4 2022), and the post-war reconstruction period (Q1 2023 – Q1 2025). The pre-war period (Q1 2005 – Q4 2019; 60 quarters) served as the analytical reference.

1.2 Ethical approval

Ethical approval was obtained from the Institutional Review Board of Tigray Health Research Institute (THRI/4031/0502/16, 7 February 2024) and the Torrens University Australia Human Research Ethics Committee (Ethics Application 0333, 14 May 2024). The Tigray Region Health Bureau granted permission for facility access (Ref No. 2579/365/16, 12 March 2024).

Because data were retrospectively extracted in deidentified, aggregate form, the requirement for individual informed consent was waived by both review boards. No personally identifiable information was extracted from the SmartCare system at any stage; only quarterly aggregate counts within pre-specified strata (sex, age, facility level, facility ownership) were transferred to the analytic dataset.

1.3 Reporting guideline

The study was designed and reported in accordance with the Strengthening the Reporting of Observational Studies in Epidemiology (STROBE) statement for cross-sectional studies and its extension for time-series designs.

© 2026 Kebede HK et al. *JAMA Network Open*.

2. Setting, participants, and facility selection

2.1 Study setting

The study was conducted in Mekelle City, the capital of Tigray Region, in northern Ethiopia. Mekelle has an estimated population of approximately 540,000 and serves as the regional referral centre for the Tigray health system. Pre-war, the city maintained a relatively concentrated HIV care infrastructure compared with rural Tigray; during the 2020–2022 conflict, urban Mekelle facilities suffered less direct physical damage than rural facilities, although blockade and supply-chain disruption affected all facility types. These contextual features influence the interpretation of our findings: this study characterises the impact of the conflict in a relatively favourable urban setting and likely understates the reduction in cascade entry that occurred in rural Tigray.

2.2 Facility eligibility and selection

According to records of the Tigray Region Health Bureau, 24 health facilities provided routine HIV care services in Mekelle City prior to the conflict. We applied the following eligibility criteria:

- Inclusion: any facility in Mekelle that provided routine HIV diagnosis, linkage, or ART services and used SmartCare for electronic record-keeping at any time during the Q1 2005 – Q1 2025 study window.
- Exclusion: privately owned facilities that declined participation; facilities that were destroyed or non-functional during the war and could not have their 2021–2022 records reconstructed; facilities with unrecoverable damage to SmartCare records during the conflict; facilities without pre-war SmartCare deployment.

Of the 24 facilities, 17 were excluded as follows:

Facilities	Reason for exclusion
4 facilities	Privately owned; declined participation.
11 facilities	Used HMIS rather than SmartCare for HIV records; did not contribute to the SmartCare-derived patient-level dataset.
2 facilities	Migrated from SmartCare to a different electronic record system since February 2024.

The remaining 7 facilities were eligible and contributed to the analysis. Their composition by facility level and ownership was:

n	Facility level	Ownership
1	Referral Hospital (Tertiary)	Public
2	General Hospital (Secondary)	Public
1	Primary Hospital (Primary)	NGO
2	Health Centre (Primary)	Public
1	Medium Clinic (Primary)	NGO

Together, these 7 facilities served a catchment population exceeding 1 million people and delivered an estimated 78–82% of pre-war routine HIV care services in Mekelle, based on 2018–2019 reports of the regional Health Management Information System.

2.3 Study population

The study population comprised all individuals who accessed HIV care services at the 7 included facilities during the Q1 2005 – Q1 2025 window. There were no exclusions by age, sex, or clinical status. Because data were aggregated

at the facility-quarter level, the unit of analysis is the quarterly aggregate count rather than the individual patient. Across all 7 facilities and 81 quarters, the study captured 13,478 incident HIV diagnoses, 11,959 linkages to care, and 9,399 ART initiations.

© 2026 Kebede HK et al. *JAMA Network Open*.

3. Data sources and extraction

3.1 SmartCare electronic medical record

Data were extracted from SmartCare, an electronic medical record (EMR) platform supported by the President's Emergency Plan for AIDS Relief (PEPFAR) and implemented in Ethiopia by the International Center for AIDS Care and Treatment Programs (ICAP) at Columbia University. SmartCare maintains comprehensive longitudinal records of HIV care at the individual patient level (registration, screening, diagnosis, linkage, ART initiation, follow-up visits, viral load testing) and supports built-in reporting modules that produce quarterly aggregate summaries by sex, age, and facility characteristics.

3.2 Data extraction procedures

Between 1 April and 30 May 2025, deidentified clinical data were extracted from each of the 7 included facilities. Extraction was performed by trained data analysts at each facility using SmartCare's standard reporting modules under the supervision of the corresponding facility HIV programme coordinator and the regional HMIS team. The extraction protocol generated quarterly aggregate counts only; no patient-level data were transferred outside the facility. Data files were transferred securely to a central study database maintained at Tigray Health Research Institute and Torrens University Australia.

3.3 Data quality and missing-data handling

We performed range checks (non-negative integer counts), consistency checks (cascade counts non-decreasing across stages within a quarter where structurally expected), and comparison against published facility annual reports for selected quarters. Sporadic missing values for individual stratum-quarters affected fewer than 5% of cells; these were imputed by linear interpolation using the zoo::na.approx function. Outcome–quarter cells with missing total counts (i.e., the facility did not operate that quarter) were left as missing rather than imputed.

3.4 Quarterly aggregation

Counts were aggregated at the facility–quarter level. Calendar quarters were defined according to the Gregorian calendar (Q1 = January–March; Q2 = April–June; Q3 = July–September; Q4 = October–December), reflecting the SmartCare reporting convention. The Ethiopian calendar quarters used by the regional HMIS were converted to Gregorian quarters before aggregation.

© 2026 Kebede HK et al. *JAMA Network Open*.

4. Time frame and four-period structure

4.1 Analytical time window

The analytical time frame spanned Q1 2005 (the earliest quarter for which SmartCare records were available across all 7 facilities) through Q1 2025 (the most recent complete calendar quarter at the time of data extraction in May 2025), yielding 82 potential quarterly observations. We excluded Q2 2025 because data extraction occurred in mid-May 2025 and the quarter therefore contained only 2 of 3 calendar months (April and May, with June incomplete), leaving 81 quarters for analysis.

4.2 Four-period structure

We divided the 81-quarter window into 4 mutually exclusive analytical periods:

Period	Quarters	n	Description
Pre-war	Q1 2005 – Q4 2019	60	Reference group
Pandemic	Q1 2020 – Q4 2020	4	COVID-19 pandemic; before war onset
War	Q1 2021 – Q4 2022	8	Active armed conflict in Tigray
Post-war	Q1 2023 – Q1 2025	9	Post-ceasefire reconstruction

4.3 Why 2020 is its own period

An earlier version of this analysis excluded all four quarters of 2020 to avoid confounding by the COVID-19 pandemic. On revision (per Reviewer 1's recommendation), we instead retained 2020 as a separate "pandemic" period in a 4-period segmented regression. This change has two consequences:

- It makes use of all available data (81 quarters rather than 78), increasing statistical efficiency.
- It separates the pandemic-attributable level shift (β_2) from the war-attributable level shift (β_3). The war-period coefficient is therefore interpreted as the change beyond the pandemic baseline rather than the change relative to a pre-pandemic baseline.

Under this specification, both β_2 (pandemic) and β_3 (war) were negative and significant for all three cascade outcomes, supporting the interpretation that war-period reductions exceeded what would be expected from the pandemic alone.

5. Outcome definitions

5.1 Three primary outcomes

We analysed three sequential indicators along the HIV care cascade, each measured as quarterly aggregate counts at the facility level:

Outcome (variable name)	Operational definition
HIV diagnoses (dx_total)	Number of individuals newly diagnosed with HIV through facility-based testing services during the quarter (incident diagnoses).
Linkage to care (lx_total_n)	Number of newly diagnosed HIV-positive individuals who enrolled in HIV care services at the facility during the quarter.
ART initiation (rx_total_n)	Number of newly diagnosed HIV-positive individuals who started antiretroviral therapy at the facility during the quarter.

5.2 Why ART initiation, not ART coverage

ART initiation as we have defined it represents incident treatment starts: the number of newly diagnosed individuals who began ART within a given quarter. It does NOT represent ART coverage (i.e., the prevalent number of individuals on ART at a given moment, which would require a different denominator and a different analytical framework). This distinction matters because the determinants of incident treatment starts (testing throughput, linkage capacity, drug supply for new initiations) differ from the determinants of prevalent ART coverage (retention, mortality, transfers in/out, drug supply for refills). Our findings should therefore be interpreted as describing disruption to cascade ENTRY rather than to overall HIV programme coverage.

5.3 Transfer-in and transfer-out definitions

Patient mobility was substantial during the conflict due to mass internal displacement. We applied the following operational definitions, derived from the SmartCare register:

- Transfer-out: A patient was classified as transferred out only when a clinician registered the patient as such in SmartCare with a destination facility specified.
- Transfer-in: A patient was classified as transferred in only when arriving with a referral note from another SmartCare-using facility.
- Patients lost to physical contact during the wartime communications blockade (approximately late 2020 through 2022) for whom no destination could be confirmed were not classified as transfers and remained in the linkage and ART denominators.

We retained this conservative approach because misclassifying disengaged patients as transfers would underestimate the conflict's impact on cascade engagement.

5.4 Exclusion handling

We did not exclude any otherwise eligible patient records. Records with missing registration dates, diagnosis dates, or ART initiation dates that could not be reconstructed from supplementary SmartCare fields were excluded only from the relevant outcome (e.g., a record missing a valid ART initiation date was excluded from the ART-initiation count for that quarter but retained in the diagnosis and linkage counts if those dates were valid).

6. Stratification scheme

6.1 Pre-specified strata

Four stratifying variables were pre-specified, yielding 11 stratum levels in addition to the overall analysis (1 + 11 = 12 stratum levels per outcome; 36 count models in total).

Stratifier	Levels	Categories
Sex	2	Female; Male
Age group	4	0–24; 25–34; 35–44; ≥45 years
Facility level	3	Primary; Secondary; Tertiary
Facility ownership	2	Public; NGO

6.2 Justification for each stratification

Sex

Sex was included because women in conflict settings face additional barriers to health-service access, including increased caregiving responsibilities, restricted mobility, elevated risk of gender-based violence, and reduced economic autonomy. Pre-war, women comprised 58.0% of HIV diagnoses in our sample; understanding whether the conflict differentially affected women is a public-health priority.

Age group

The 0–24, 25–34, 35–44, and ≥45-year cut-points align with WHO HIV surveillance categories used widely in sub-Saharan Africa and reflect the concentration of HIV epidemiology in the 25–44 reproductive-age groups in Ethiopia. The 0–24 group includes adolescents and young adults whose engagement with HIV services is particularly vulnerable to disruption.

Facility level

Primary, secondary, and tertiary facilities differ in physical infrastructure, staffing depth, supply-chain redundancy, and patient catchment. We expected differential vulnerability and differential capacity for service redistribution.

Facility ownership

Public and NGO-managed facilities depend on different funding streams, supply chains, and personnel arrangements. The single NGO-managed facility in our sample was included as a separate stratum so that its experience would not be obscured in pooled estimates; we explicitly caveat that findings for this stratum reflect a single institution and should not be generalised to NGO-managed HIV programmes broadly.

6.3 Single-NGO-facility caveat

Because only two NGO-managed facilities met the eligibility criteria (one primary hospital and one clinic), the "NGO" ownership stratum comprises a small sample (n = 2). All findings for this stratum are presented with an explicit caveat in the manuscript and supplement: stratum-level estimates describe the trajectory of two facilities and should not be extrapolated to NGO-managed HIV programmes more broadly. The contrast between the public and NGO strata is presented as a hypothesis-generating observation rather than as evidence of a systematic differential effect by ownership type.

© 2026 Kebede HK et al. *JAMA Network Open*.

7. Statistical analysis: count-model segmented regression

7.1 Overall analytical strategy

The primary analysis used negative binomial segmented regression to estimate level changes in the three cascade outcomes at the boundaries of the pandemic, war, and post-war periods relative to the pre-war reference. Quasi-Poisson regression was used as a fallback for the small minority of stratum-level models where the negative binomial fit failed to converge. We refer to this collectively as the COUNT-MODEL segmented regression.

All effect estimates in the manuscript Results section, in Table 2, and in eTable 5 of the Supplementary Tables are from this count-model framework, with one exception: a single rate-based offset model for linkage to care, fitted as a sensitivity check responding to Reviewer 1, is reported separately in eTable 6 of the Supplementary Tables.

7.2 Mathematical specification

For each cascade outcome at quarter t (t = 1, 2, ..., 81), we fitted:

$$\log(E[Y_t]) = \beta_0 + \beta_1 \cdot t + \beta_2 \cdot pandemic_t + \beta_3 \cdot war_t + \beta_4 \cdot post_t + \beta_5 \cdot \sin(2\pi t/4) + \beta_6 \cdot \cos(2\pi t/4)$$

where:

- Y_t is the quarterly aggregate count of the cascade outcome (HIV diagnoses, linkages, or ART initiations) at quarter t.
- $E[Y_t]$ is the expected count, with Y_t assumed to follow a negative binomial distribution with mean $E[Y_t]$ and dispersion parameter θ estimated from the data.
- t is the quarter index (1–81), capturing the linear secular trend.
- $pandemic_t$, war_t, and $post_t$ are 0/1 indicator variables for the corresponding analytical periods (Section 4.2).
- sin(2πt/4) and cos(2πt/4) form a sine–cosine pair capturing annual seasonality at quarterly frequency. Including both terms allows the harmonic to fit any phase.

The seven coefficients are interpreted as follows:

Coef	Term	Interpretation
β_0	Intercept	Log of expected count at t = 0 (Q1 2005), with all dummies = 0.
β_1	Linear time trend	Log of the per-quarter multiplicative change; $\exp(\beta_1)$ is the per-quarter IRR.
β_2	Pandemic effect	Log IRR of pandemic period vs pre-war counterfactual.
β_3	War effect	Log IRR of war period vs pre-war counterfactual.
β_4	Post-war effect	Log IRR of post-war period vs pre-war counterfactual.
β_5	Sine seasonal	Sine component of annual seasonality.
β_6	Cosine seasonal	Cosine component of annual seasonality.

7.3 Reference group and effect-estimate scale

All effect estimates are reported relative to the pre-war period, which is the reference group (all three period dummies = 0). Coefficients β_2, β_3, and β_4 were exponentiated to obtain incidence rate ratios (IRRs):

- IRR < 1 indicates a reduction in expected count compared with pre-war (e.g., IRR = 0.71 indicates a 29% reduction).
- IRR > 1 indicates an increase compared with pre-war.

© 2026 Kebede HK et al. *JAMA Network Open*.

- IRR = 1 indicates no change.

95% Wald confidence intervals were computed on the log scale and exponentiated: exp(β ± 1.96·SE).

7.4 Why level-change only (no time × period interactions)

The primary specification includes period-level dummies but does not include time × period interaction terms (i.e., post-war within-period slopes are constrained to equal the pre-war secular trend). This choice was made for two reasons:

- The clinical question is the average level shift in each period ("did the war suppress cascade entry?"), not the within-period rate of change.
- The pandemic period (4 quarters) and war period (8 quarters) are too short to estimate stable within-period slopes without imposing strong distributional assumptions.

A slope-change specification with time-after-war and time-after-postwar terms is reported as Sensitivity Analysis 2 (Section 10.2; eTable 9 of the Supplementary Tables); the slope-change terms were not statistically significant for any of the three primary outcomes, confirming that the level-change specification captures the dominant signal.

7.5 Estimator and link function

7.5.1 Negative binomial regression (primary)

Negative binomial regression with a log link was the primary estimator. We used the glmmTMB::glmmTMB() function with family = nbinom2, which estimates the dispersion parameter θ from the data. The optimizer was nlminb with eval.max = 2000 and iter.max = 1000.

```
glmmTMB(formula = Y ~ t + pandemic + war + post + sin_annual + cos_annual,
       family = nbinom2(),
       data = analysis_data,
       control = glmmTMBControl(optimizer = nlminb,
                         optCtrl = list(eval.max = 2000, iter.max = 1000)))
```

7.5.2 Convergence verification

For each fitted model, we verified two convergence conditions before accepting the estimates:

- The Hessian was positive-definite (sdr$pdHess == TRUE), indicating a valid local optimum.
- All parameter standard errors were finite (not NaN, not Inf) and below 1000 (extremely large standard errors typically indicate identifiability issues such as separation).

Models that failed either condition were treated as non-convergent and refitted using the quasi-Poisson fallback.

7.5.3 Quasi-Poisson regression (fallback)

When negative binomial fitting failed to converge, the model was refitted using quasi-Poisson regression via stats::glm with family = quasipoisson(link = "log"). Quasi-Poisson, rather than ordinary Poisson, was used as the fallback because it estimates the dispersion parameter from the data and does not assume mean = variance, which would be unrealistic for our overdispersed counts. Of the 36 count models reported in the manuscript, 35 used negative binomial regression and 1 (HIV Diagnoses, age 25–34 years) used quasi-Poisson regression.

© 2026 Kebede HK et al. *JAMA Network Open*.

7.5.4 Why not Poisson?

We did not use Poisson regression as a fallback because Poisson assumes mean = variance, which is rarely satisfied for facility-level health-service counts (where overdispersion is the norm). A Poisson fallback would have understated the standard errors and produced inappropriately narrow confidence intervals.

© 2026 Kebede HK et al. *JAMA Network Open*.

8. Counterfactual projection and cumulative deficits

8.1 Counterfactual projection

The counterfactual is the trajectory each cascade outcome would have followed in the absence of the pandemic and war shocks, assuming the pre-war secular trend and seasonal pattern would have continued unchanged. Operationally, we generated counterfactual predictions by:

- Setting $pandemic_t = 0$, $war_t = 0$, and $post_t = 0$ for all post-2019 observations.
- Holding the linear time term and seasonal harmonics at their fitted values.
- Predicting on the response (count) scale using stats::predict(model, newdata, type = "response").

```
counterfactual_data <- analysis_data %>%
  mutate(pandemic_dummy = 0,
         war_dummy      = 0,
         postwar_dummy  = 0)

counterfactual_pred <- predict(model, newdata = counterfactual_data, type = "response")
```

8.2 Cumulative deficit definition

The cumulative cascade deficit is the integrated difference between the counterfactual trajectory and the observed trajectory across the war and post-war quarters. Formally:

$$Deficit = \Sigma\ (counterfactual_t - observed_t)\ \ for\ t \in \{war \cup post\text{-}war\ quarters\}$$

A positive deficit indicates that fewer individuals entered the cascade than would have been expected under the counterfactual; a negative deficit (rare in our data) would indicate higher than expected enrolment.

8.3 Bootstrap confidence intervals

Confidence intervals for cumulative deficits were obtained via parametric bootstrap with 5000 replications. The procedure for each replicate b = 1, ..., 5000 was:

- For each quarter t, draw $Y_t^{(b)}$ from a negative binomial distribution with mean μ_t (the fitted value from the original model) and dispersion θ (the dispersion estimate from the original model).
- Refit the segmented regression on the bootstrap sample.
- Generate counterfactual predictions for the bootstrap fit.
- Compute the cumulative deficit for the bootstrap replicate.

We then took the 2.5th and 97.5th percentiles of the resulting 5000-replicate empirical distribution as the 95% bootstrap confidence interval. CIs that include zero indicate that statistical evidence for a non-zero cumulative gap is uncertain at the 95% level even though point estimates are large.

Random seed for reproducibility: set.seed(2025) was set at the top of the analysis script.

© 2026 Kebede HK et al. *JAMA Network Open*.

9. Model diagnostics

9.1 DHARMa simulation-based residuals

We assessed model adequacy using the DHARMa package (Hartig 2022, version 0.4.6 or later). DHARMa generates simulation-based scaled residuals by simulating from the fitted model and computing the empirical CDF of the observed value relative to the simulated values, yielding residuals that are uniformly distributed on [0, 1] under correct model specification regardless of the underlying GLM family. This makes DHARMa diagnostics valid for negative binomial and quasi-Poisson models alike.

```
library(DHARMa)
sim_res <- simulateResiduals(fittedModel = model, n = 250, plot = FALSE)
```

9.2 Formal tests

Two formal DHARMa tests were applied to each fitted model:

9.2.1 testDispersion()

A permutation test comparing the observed simulated dispersion to that expected under correct model specification. The null hypothesis is that the model captures the data dispersion correctly. P values are reported in eTable 5 of the Supplementary Tables.

9.2.2 testTemporalAutocorrelation()

A Durbin-Watson-style test applied to the simulated quantile residuals ordered by time. The null hypothesis is no residual temporal autocorrelation. We applied this test to ordered quarterly residuals using time = seq_along(scaledResiduals).

9.3 Why DHARMa rather than Durbin-Watson on Pearson residuals?

An earlier version of this analysis used Durbin-Watson tests applied directly to the Pearson residuals from the GLM. The Durbin-Watson test, however, is strictly valid only for ordinary least squares residuals; applying it to Pearson residuals from a GLM lacks a rigorous theoretical justification and can mislead. DHARMa's testTemporalAutocorrelation is valid for GLMs because the simulated quantile residuals are uniform under the null, removing the dependence on the GLM family.

9.4 Visual diagnostics

For each overall (non-stratified) model, we generated a four-panel visual diagnostic (eFigure 9 of the Supplementary Figures):

- Pearson residuals vs time, with LOESS smoother and 95% confidence band.
- Observed vs fitted values, with 1:1 reference line and linear regression line.
- Autocorrelation function of residuals, with 95% Bartlett bands at $\pm 1.96/\sqrt{n}$.
- Histogram of Pearson residuals, with overlaid normal density and kernel density.

Stratum-level diagnostics were inspected during model development; those for the Female and 0–24-year strata are included in the Supplementary Figures as exemplars.

10. Sensitivity analyses

Four pre-specified sensitivity analyses tested the robustness of the main count-model findings to specific modelling assumptions. Detailed results appear in eTables 8–11 of the Supplementary Tables.

10.1 Sensitivity 1: model without seasonal harmonics

We refitted all overall models with the sin and cos terms removed, retaining only the linear time and period dummies. Pandemic and war IRRs changed by less than 2% from the main analysis, indicating that the seasonality assumption does not drive findings.

10.2 Sensitivity 2: full slope-change specification

We refitted all overall models with two additional terms, time-after-war (slope after war onset) and time-after-postwar (slope after ceasefire), allowing within-period trends to differ from the pre-war secular trend. The slope-change terms were non-significant for all three outcomes, indicating that within-period slopes did not differ detectably from pre-war once the level shifts were modelled. War-period level-shift IRRs in this specification absorb some of the slope-change signal and therefore appear attenuated relative to the main analysis; this is a known artefact of joint level-and-slope segmented regression and should not be interpreted as evidence against the main level-shift findings.

10.3 Sensitivity 3: alternative war onset (Q4 2020)

The war began in early November 2020, partway through Q4 2020. The main analysis assigned Q4 2020 to the pandemic period (the war was less than 8 weeks of the quarter at the time of the November 3 ceasefire-trigger event); this sensitivity analysis alternatively assigned Q4 2020 to the war period (war = 9 quarters; pandemic = 3 quarters). War-period IRRs were slightly more negative than the main analysis (e.g., HIV diagnoses 0.67 vs 0.71); pandemic IRRs were slightly attenuated. Direction and qualitative interpretation are unchanged.

10.4 Sensitivity 4: pre-war restricted to 2015 onward

Ethiopia adopted the WHO test-and-treat universal-eligibility recommendation in approximately 2016. To address the concern that the long pre-war baseline (2005–2019) could include eras with very different ART eligibility criteria, we refitted all overall models with the pre-war baseline restricted to Q1 2015 – Q4 2019 (the post-test-and-treat era). War-period IRRs were notably more negative in this sensitivity analysis (e.g., HIV diagnoses 0.52 vs 0.71), reflecting the higher recent baseline against which the war-period reduction is measured. The direction of all effects is unchanged, and the qualitative conclusion that the war period was associated with substantial reductions in cascade entry is strengthened.

© 2026 Kebede HK et al. *JAMA Network Open*.

11. Complementary ARIMA analysis

11.1 Rationale

ARIMA (autoregressive integrated moving average) modelling provides a complementary time-series framework that explicitly accommodates serial autocorrelation in the outcome through autoregressive and moving-average terms. We fitted ARIMA models with exogenous intervention regressors (the same pandemic, war, and post-war 0/1 indicators as in the segmented regression) to provide a sensitivity check on the period-effect estimates that does not rely on the negative binomial dispersion assumption.

11.2 Specification

We used forecast::auto.arima to identify ARIMA orders, with the following options:

```
library(forecast)
y_ts  <- ts(Y, frequency = 4)
xreg  <- cbind(pandemic = pandemic_dummy,
               war      = war_dummy,
               postwar  = postwar_dummy)

arima_fit <- auto.arima(y_ts,
                        xreg          = xreg,
                        seasonal      = TRUE,
                        stepwise      = FALSE,
                        approximation = FALSE,
                        max.p = 4, max.q = 4)
```

Setting stepwise = FALSE and approximation = FALSE forces auto.arima to perform a full search over candidate orders rather than a stepwise approximation, at the cost of computation time. This choice yields more reliable order selection for the moderate series lengths in our data (81 quarters).

11.3 Counterfactual prediction for ARIMA

ARIMA counterfactuals were generated by setting the xreg matrix to zero for all post-2019 observations and refitting using stats::Arima with model = arima_fit (which fixes the AR/MA orders and parameters from the original fit while updating the intervention coefficients):

```
counterfactual_xreg <- xreg; counterfactual_xreg[,] <- 0
arima_cf <- Arima(y_ts, model = arima_fit, xreg = counterfactual_xreg)
counterfactual_pred <- as.numeric(fitted(arima_cf))
```

11.4 Cross-validation against segmented regression

All ARIMA intervention coefficients had the SAME DIRECTION as the corresponding negative binomial segmented regression IRRs (pandemic and war negative; post-war near pre-war). ARIMA P values were generally larger than the segmented regression P values, reflecting ARIMA standard errors that fully account for residual autocorrelation through the AR/MA structure. The convergence of the two analytical frameworks on the same qualitative conclusions strengthens confidence in the main findings. ARIMA results appear in eTable 7 of the Supplementary Tables.

12. Effect modification tests

12.1 Long-format model construction

To formally test effect modification by sex, age, facility level, and ownership, we constructed long-format datasets by stacking the stratum-level counts within each stratifier. For each combination of cascade outcome and stratifier, we fitted two MASS::glm.nb models:

Main-effects model

```
main_model <- MASS::glm.nb(
  count ~ t + pandemic_dummy + war_dummy + postwar_dummy +
          sin_annual + cos_annual + stratum,
  data = long_data)
```

Interaction model

```
interaction_model <- MASS::glm.nb(
  count ~ t + pandemic_dummy * stratum +
          war_dummy      * stratum +
          postwar_dummy  * stratum +
          sin_annual + cos_annual,
  data = long_data)
```

12.2 Likelihood ratio test

We compared the two models using a likelihood ratio test (LRT):

```
anova(main_model, interaction_model)
```

The LRT compares twice the difference in log-likelihoods to a chi-squared distribution with degrees of freedom equal to the number of additional parameters in the interaction model. A small P value ($P < .05$ by convention) indicates that the period effect varies across strata after adjusting for the main stratum effect and other covariates.

12.3 Implementation note: glm.nb vs glmmTMB

We used MASS::glm.nb rather than glmmTMB for the interaction tests because anova(...) with method = "Chisq" is more reliable for nested glm.nb models and the syntax is more familiar to most readers. Estimates from glm.nb and glmmTMB(family = nbinom2) are equivalent for the count specifications used here.

12.4 Reporting

LRT chi-squared statistics, degrees of freedom, and JAMA-formatted P values are reported in eTable 12 of the Supplementary Tables. Significant interactions for age and ownership across all three outcomes (all $P < .001$) confirm heterogeneous effects by these stratifiers. Sex and facility-level interactions did not reach conventional significance thresholds, although stratum-level IRRs differed substantially in both cases (e.g., women IRR ≈ 0.55–0.57 vs men IRR ≈ 0.89–0.92 for HIV diagnoses); the non-significant interaction LRTs reflect the low statistical power for detecting interactions in moderate-sized time series.

© 2026 Kebede HK et al. *JAMA Network Open*.

13. Software, reproducibility, and reporting

13.1 R version and packages

All analyses were conducted in R version 4.6.0 (or later) using CRAN-published packages only:

Domain	Packages
Data wrangling	dplyr, tidyr, readr, stringr, purrr, tibble
Modelling	glmmTMB (negative binomial), MASS (glm.nb for interactions), DHARMa (diagnostics), forecast (ARIMA)
Visualisation	ggplot2, scales, patchwork
Output	openxlsx (Excel workbook export)
Utility	zoo (linear interpolation of <5% missing data)

13.2 Random seed

set.seed(2025) was set at the top of the analysis script. This affects the parametric bootstrap for cumulative deficit confidence intervals and the DHARMa simulation-based residuals; segmented regression point estimates are deterministic given the data.

13.3 Reproducibility

The complete R code (974 lines) is provided as the eAppendix of the Supplementary Tables document. With access to the input data file at the path defined at the top of the script (an investigator with appropriate ethical clearance from THRI and TUA may request access via the corresponding author), running the script from start to finish reproduces all numerical results, all figures, and the Excel workbook used to populate the supplementary tables.

13.4 Reporting standard

The study was designed and reported in accordance with the STROBE statement for observational studies, with attention to the recommendations for time-series designs. A completed STROBE checklist is provided as Supplementary Material on submission.

13.5 P value and CI formatting

All P values reported in the manuscript, tables, and supplement follow JAMA Network Open formatting conventions:

- P < .001 → reported as "<.001"
- P > .99 → reported as ">.99"
- 0.001 ≤ P < 0.01 → 3 decimal places (e.g., ".003")
- P ≥ 0.01 → 2 decimal places (e.g., ".04", ".45")
- Leading zeros are omitted (".04", not "0.04").

All confidence intervals are 95% Wald intervals computed on the log scale and exponentiated to the IRR scale. Bootstrap intervals (used only for cumulative deficits) use the 2.5th and 97.5th percentiles of the empirical distribution.

© 2026 Kebede HK et al. *JAMA Network Open*.

eAppendix 2. Complete R Analysis Script

Tigray War and HIV Care Cascade Entry in Mekelle, Ethiopia

The R code reproduced in the following pages (974 lines) runs the complete interrupted time-series analysis: data wrangling and quarterly aggregation, the negative binomial segmented regression with seasonal harmonics, the four pre-specified sensitivity analyses, ARIMA-with-intervention-regressor models, DHARMa simulation-based residual diagnostics, parametric bootstrap (5,000 replications) for cumulative cascade deficit confidence intervals, period × stratifier likelihood-ratio interaction tests, and all output (Excel workbook and PDF/PNG figures). With access to the input data file (path defined at the top of the script), running the file end-to-end deterministically reproduces every numerical result in this supplement.

© 2026 Kebede HK et al. *JAMA Network Open*.

R Analysis Script

Interrupted time-series analysis of the HIV care cascade

What this script does. Fits a four-period interrupted time-series segmented regression to quarterly facility-level HIV care cascade counts (HIV diagnoses, linkage to care, ART initiation), with stratified analyses by sex, age group, facility level, and ownership; produces counterfactual projections, cumulative deficits with parametric bootstrap confidence intervals, complementary ARIMA-with-intervention-regressors fits, four prespecified sensitivity analyses, period × stratifier interaction tests, and DHARMa residual diagnostics. Outputs a multi-sheet Excel workbook with all numerical results and PNG/PDF figures for each model and stratum.

© 2026 Kebede HK et al. *JAMA Network Open*.

Requirements and how to run

R version and packages

R version 4.6.0 or later. CRAN-published packages only:

- Data wrangling: dplyr, tidyr, readr, stringr, purrr, tibble, zoo
- Modelling: glmmTMB (negative binomial), MASS (glm.nb for interaction tests), forecast (ARIMA)
- Diagnostics: DHARMa
- Visualisation: ggplot2, scales, patchwork
- Output: openxlsx

Reproducibility

- Random seed: set.seed(2025) at the top of the script.
- All numerical results, figures, and the Excel workbook are reproduced deterministically given the input data.

Inputs

The script expects a quarterly aggregate dataset with one row per facility-quarter and columns for HIV diagnoses, linkage to care, and ART initiation counts, broken down by the four stratifying variables (sex, age group, facility level, ownership). The data path is defined at the top of the script (DATA_PATH); replace the placeholder with the path to your input CSV.

Outputs

- Multi-sheet Excel workbook (15 sheets): main analysis, publication-style tables, full coefficients, period summaries, outcome statistics, pre-war trends, stratum contributions, ARIMA, four sensitivity analyses, period × stratifier interactions, and cumulative deficits with bootstrap CIs.
- PNG and PDF figures: ITS fits per model and stratum, ARIMA fits and diagnostics, DHARMa diagnostic composites, sensitivity-analysis fits, and combined publication-quality cascade plot.

© 2026 Kebede HK et al. *JAMA Network Open*.

Source code

The complete script is reproduced below. Comments inside the script document each section; running the file from start to finish reproduces every output.

```
################################################################################
# Interrupted time-series analysis of the HIV care cascade
# Tigray, Ethiopia (Q1 2005 – Q1 2025; 81 quarters)
#
# R version 4.6.0 (or later). CRAN-published packages only.
#
# Analysis components:
#   1. Primary: negative binomial segmented regression (level-change with seasonality)
#        log(Y_t) = b0 + b1*t + b2*pandemic + b3*war + b4*post + sin + cos
#   2. Complementary: ARIMA with intervention regressors (auto.arima + xreg)
#   3. Diagnostics: DHARMa residual plots + dispersion + autocorrelation tests
#   4. Sensitivity analyses: (i) no seasonality, (ii) full slope-change model,
#                            (iii) alt war onset Q4 2020, (iv) pre-war restricted to 2015+
#   5. Interaction tests: period x sex / age / ownership / facility level (LR tests)
#   6. Cumulative deficits: parametric bootstrap 95% CIs (5000 replications)
#   7. Figures: vector PDF (cairo_pdf) + PNG at 600 DPI
################################################################################

# ============================================================================
#                              CONFIGURATION
# ============================================================================

DATA_PATH  <- "path/to/cascade_data.csv"
OUTPUT_DIR <- "path/to/cascade_data.csv"

RUN_SENSITIVITY <- TRUE
RUN_BOOTSTRAP   <- TRUE
BOOT_R          <- 5000
FIG_DPI         <- 600     # resolution for vector/raster output

PRE_END        <- list(year = 2019, q = 4)
PANDEMIC_START <- list(year = 2020, q = 1)
PANDEMIC_END   <- list(year = 2020, q = 4)
WAR_START      <- list(year = 2021, q = 1)
WAR_END        <- list(year = 2022, q = 4)
POST_START     <- list(year = 2023, q = 1)
POST_END       <- list(year = 2025, q = 1)   # Q2 2025 excluded (incomplete)

# ============================================================================
#                              PACKAGES (15, CRAN-only)
# ============================================================================

set.seed(2025)
options(warn = -1)

required_packages <- c(
  "dplyr", "tidyr", "readr", "stringr", "purrr", "tibble",
  "glmmTMB", "MASS", "DHARMa",
  "forecast",                   # ARIMA with intervention regressors
  "ggplot2", "scales", "patchwork",
  "openxlsx",
  "zoo"
)
```

© 2026 Kebede HK et al. *JAMA Network Open*.

```
new_pkgs <- required_packages[!(required_packages %in% installed.packages()[, "Package"])]
if (length(new_pkgs) > 0)
  install.packages(new_pkgs, repos = "https://cran.rstudio.com/", dependencies = TRUE)

suppressPackageStartupMessages(
  invisible(lapply(required_packages, library, character.only = TRUE)))
options(warn = 0)

select <- dplyr::select;  filter <- dplyr::filter
`%||%` <- function(a, b) if (is.null(a)) b else a

# ============================================================================
#                              SETUP & LOGGING
# ============================================================================

cat("\n+------------------------------------------------------------+\n")
cat("|  Interrupted time-series cascade analysis              |\n")
cat("|  R ", as.character(getRversion()), " | NB Seg-Reg + ARIMA + DHARMa     |\n", sep = "")
cat("|  All figures: PDF vector + PNG at ", FIG_DPI, " DPI       |\n", sep = "")
cat("+----------------------------------------------------------+\n\n")

output_subdirs <- c(
  "figures/publication", "figures/descriptive", "figures/sensitivity",
  "figures/arima", "figures/diagnostics",
  "tables", "rds/main", "rds/sensitivity"
)
dir.create(OUTPUT_DIR, recursive = TRUE, showWarnings = FALSE)
for (d in output_subdirs)
  dir.create(file.path(OUTPUT_DIR, d), recursive = TRUE, showWarnings = FALSE)

log_file <- file.path(OUTPUT_DIR, "analysis_log.txt")
try(cat("", file = log_file), silent = TRUE)

log_message <- function(msg) {
  ts <- format(Sys.time(), "%Y-%m-%d %H:%M:%S")
  line <- paste0("[", ts, "] ", msg, "\n")
  cat(line)
  try(cat(line, file = log_file, append = TRUE), silent = TRUE)
}

log_message("=== REVISED ITS CASCADE ANALYSIS STARTED ===")
log_message(paste("R version:", as.character(getRversion())))
log_message(paste("Output:", OUTPUT_DIR, "| DPI:", FIG_DPI))

safe_name <- function(s) {
  s <- tolower(s); s <- str_replace_all(s, "[^a-z0-9]+", "_")
  s <- str_replace_all(s, "_+", "_"); str_replace_all(s, "^_|_$", "")
}

# ============================================================================
#                        PUBLICATION THEME & COLORS
# ============================================================================

theme_publication <- function(base_size = 11) {
  theme_bw(base_size = base_size) +
    theme(
```

© 2026 Kebede HK et al. *JAMA Network Open*.

```
      plot.title       = element_text(size = rel(1.2), face = "bold", hjust = 0, margin = margin(b = 10)),
      plot.subtitle    = element_text(size = rel(0.9), color = "gray30", margin = margin(b = 15)),
      plot.caption     = element_text(size = rel(0.8), color = "gray50", hjust = 1, margin = margin(t = 10)),
      axis.title       = element_text(size = rel(1.0), face = "bold"),
      axis.text        = element_text(size = rel(0.9), color = "black"),
      axis.line        = element_line(color = "black", linewidth = 0.5),
      legend.position  = "bottom",
      panel.grid.major = element_line(color = "gray90", linewidth = 0.3),
      panel.grid.minor = element_blank(),
      panel.border     = element_rect(color = "black", fill = NA, linewidth = 0.5),
      strip.background = element_rect(fill = "gray95", color = "black", linewidth = 0.5),
      strip.text       = element_text(size = rel(1.0), face = "bold"))
}

period_colors <- c("Pre-war"="#1B5E20", "Pandemic"="#FF8F00", "War"="#B71C1C", "Post-war"="#0D47A1")
intervention_colors <- c(pandemic="#F57C00", war="#D32F2F", postwar="#1976D2")
fitted_colors <- c(Observed="#000000", Fitted="#1976D2", Counterfactual="#757575")

# ============================================================================
#                        DATA LOADING & CLEANING  (4-period model)
# ============================================================================

log_message("Step 1: Loading and cleaning data (4-period structure)")
if (!file.exists(DATA_PATH)) stop("Data file not found: ", DATA_PATH)
raw_data <- read_csv(DATA_PATH, show_col_types = FALSE)
log_message(paste("Loaded:", nrow(raw_data), "rows,", ncol(raw_data), "cols"))

clean_data_4period <- function(df,
                               pandemic_end_q      = PANDEMIC_END$q,
                               war_start_year      = WAR_START$year,
                               war_start_quarter   = WAR_START$q,
                               postwar_start_year  = POST_START$year,
                               postwar_start_quarter = POST_START$q) {

  df <- df %>%
    mutate(year_quarter = as.character(year_quarter),
           year    = as.integer(str_extract(year_quarter, "^\\d{4}")),
           quarter = as.integer(str_extract(year_quarter, "(?<=_q)\\d"))) %>%
    filter(!is.na(year), !is.na(quarter),
           !(year == 2025 & quarter >= 2),
           year <= POST_END$year)

  # --- 4-period assignment ---
  # When war_start is moved to Q4 2020, pandemic shrinks to Q1-Q3 2020
  # (fixes Sensitivity 3 producing identical results)
  df <- df %>%
    mutate(
      period = case_when(
        year < PANDEMIC_START$year ~ "pre",
        # Pandemic: 2020 quarters BEFORE war_start (handles alt onset)
        year == 2020 & (year < war_start_year |
                        (year == war_start_year & quarter < war_start_quarter)) ~ "pandemic",
        # War period
        (year == war_start_year & quarter >= war_start_quarter) |
          (year > war_start_year & year < postwar_start_year) |
          (year == postwar_start_year & quarter < postwar_start_quarter) ~ "war",
        # Post-war
```

© 2026 Kebede HK et al. *JAMA Network Open*.

```
        (year == postwar_start_year & quarter >= postwar_start_quarter) |
          (year > postwar_start_year) ~ "post",
        TRUE ~ NA_character_
      ),
      period = factor(period, levels = c("pre", "pandemic", "war", "post"))
    ) %>%
    filter(!is.na(period)) %>%
    arrange(year, quarter) %>%
    mutate(t = row_number(),
           date_numeric = year + (quarter - 1) / 4,
           date_label   = paste0(year, " Q", quarter),
           pandemic_dummy = as.integer(period == "pandemic"),
           war_dummy      = as.integer(period == "war"),
           postwar_dummy  = as.integer(period == "post"))

  # Slope-change vars (sensitivity only)
  if (any(df$war_dummy == 1)) {
    wt <- min(df$t[df$war_dummy == 1 | df$postwar_dummy == 1])
    df$time_after_war <- ifelse(df$war_dummy + df$postwar_dummy > 0, df$t - wt + 1, 0)
  } else df$time_after_war <- 0
  if (any(df$postwar_dummy == 1)) {
    pt <- min(df$t[df$postwar_dummy == 1])
    df$time_after_postwar <- ifelse(df$postwar_dummy == 1, df$t - pt + 1, 0)
  } else df$time_after_postwar <- 0

  df <- df %>% mutate(sin_annual = sin(2*pi*t/4), cos_annual = cos(2*pi*t/4))

  # Coerce types
  for (col in names(df)[str_detect(names(df), "_pct$")])
    if (is.character(df[[col]])) df[[col]] <- as.numeric(str_remove(df[[col]], "%"))
  for (col in names(df)[str_detect(names(df), "_n$|_total$") & !str_detect(names(df), "_pct")])
    df[[col]] <- as.numeric(df[[col]])
  df
}

analysis_data <- clean_data_4period(raw_data)
log_message(paste("Analysis:", nrow(analysis_data), "quarters"))
pc <- table(analysis_data$period)
log_message(paste("Periods:", paste(names(pc), pc, sep="=", collapse=", ")))

# Interpolate sporadic missing (<5%)
ms <- analysis_data %>%
  summarise(across(where(is.numeric), ~sum(is.na(.))/n()*100)) %>%
  pivot_longer(everything(), names_to="v", values_to="p") %>% filter(p > 0 & p < 5)
if (nrow(ms) > 0) {
  log_message(paste("Interpolating", nrow(ms), "vars with <5% missing"))
  for (v in ms$v) analysis_data[[v]] <- na.approx(analysis_data[[v]], na.rm = FALSE)
}

# ============================================================================
#                         OUTCOME DEFINITIONS
# ============================================================================

outcomes <- list(
  diagnosis = list(
    total = "dx_total", label = "HIV Diagnoses",
    strata = list(
```

© 2026 Kebede HK et al. *JAMA Network Open*.

```r
      sex = c(Female="dx_female_n", Male="dx_male_n"),
      age = c("0-24 years"="dx_aged_0_24yrs_n", "25-34 years"="dx_aged_25_34_yrs_n",
              "35-44 years"="dx_aged_35_44_yrs_n", "45+ years"="dx_aged_45plus_yrs_n"),
      facility_level = c(Primary="dx_facility_level_primary_n",
                         Secondary="dx_facility_level_secondary_n",
                         Tertiary="dx_facility_level_tertiary_n"),
      ownership = c(NGO="dx_facility_ownership_ngo_n", Public="dx_facility_ownership_public_n"))),
  linkage = list(
    total = "lx_total_n", label = "Linkage to Care", denominator = "dx_total",
    strata = list(
      sex = c(Female="lx_female_n", Male="lx_male_n"),
      age = c("0-24 years"="lx_aged_0_24yrs_n", "25-34 years"="lx_aged_25_34_yrs_n",
              "35-44 years"="lx_aged_35_44_yrs_n", "45+ years"="lx_aged_45plus_yrs_n"),
      facility_level = c(Primary="lx_facility_level_primary_n",
                         Secondary="lx_facility_level_secondary_n",
                         Tertiary="lx_facility_level_tertiary_n"),
      ownership = c(NGO="lx_facility_ownership_ngo_n", Public="lx_facility_ownership_public_n"))),
  initiation = list(
    total = "rx_total_n", label = "ART Initiation", denominator = "lx_total_n",
    strata = list(
      sex = c(Female="rx_female_n", Male="rx_male_n"),
      age = c("0-24 years"="rx_aged_0_24yrs_n", "25-34 years"="rx_aged_25_34_yrs_n",
              "35-44 years"="rx_aged_35_44_yrs_n", "45+ years"="rx_aged_45plus_yrs_n"),
      facility_level = c(Primary="rx_facility_level_primary_n",
                         Secondary="rx_facility_level_secondary_n",
                         Tertiary="rx_facility_level_tertiary_n"),
      ownership = c(NGO="rx_facility_ownership_ngo_n", Public="rx_facility_ownership_public_n")))
)

# ============================================================================
#                        MODEL FITTING HELPERS  (quasi-Poisson fallback)
# ============================================================================

glmmtmb_ok <- function(mod) {
  if (is.null(mod) || !isTRUE(mod$sdr$pdHess)) return(FALSE)
  se <- tryCatch(summary(mod)$coefficients$cond[,"Std. Error"], error=function(e) NA)
  !any(is.nan(se)|is.infinite(se)|is.na(se)) && all(se < 1e3)
}

glm_ok <- function(mod) {
  if (is.null(mod) || !isTRUE(mod$converged) || any(is.na(coef(mod)))) return(FALSE)
  se <- tryCatch(coef(summary(mod))[,"Std. Error"], error=function(e) NA)
  !any(is.nan(se)|is.infinite(se)|is.na(se)) && all(se < 1e3)
}

fit_with_fallback <- function(formula_obj, data, model_label="", is_binomial=FALSE) {
  if (is_binomial) {
    m <- tryCatch(glm(formula_obj, family=binomial(link="logit"), data=data), error=function(e) NULL)
    if (glm_ok(m)) { log_message(paste("    Binomial OK:", model_label)); return(list(model=m, family="Binomial")) }
    log_message(paste("    FAILED:", model_label)); return(NULL)
  }
  # NB via glmmTMB
  m <- tryCatch(suppressWarnings(glmmTMB(formula_obj, data=data, family=nbinom2(),
    control=glmmTMBControl(optimizer=nlminb, optCtrl=list(eval.max=2000, iter.max=1000)))),
    error=function(e) NULL)
  if (glmmtmb_ok(m)) { log_message(paste("    NB OK:", model_label)); return(list(model=m, family="Negative Binomial")) }
```

© 2026 Kebede HK et al. *JAMA Network Open*.

```
  # Quasi-Poisson fallback
  m <- tryCatch(glm(formula_obj, family=quasipoisson(link="log"), data=data), error=function(e) NULL)
  if (glm_ok(m)) { log_message(paste("    Quasi-Poisson OK:", model_label)); return(list(model=m, family="Quasi-Poisson")) }
  log_message(paste("    FAILED:", model_label)); NULL
}

extract_coefs <- function(model) {
  if (inherits(model,"glmmTMB")) {
    s<-summary(model)$coefficients$cond; est<-s[,"Estimate"]; se<-s[,"Std. Error"]
    z<-s[,"z value"]; p<-s[,"Pr(>|z|)"]; nm<-rownames(s)
  } else { s<-coef(summary(model)); est<-s[,1]; se<-s[,2]; z<-s[,3]; p<-s[,4]; nm<-rownames(s) }
  lo<-est-1.96*se; hi<-est+1.96*se
  tibble(term=nm, estimate=est, std.error=se, statistic=z, p.value=p,
         conf.low=lo, conf.high=hi, irr=exp(est), irr_lo=exp(lo), irr_hi=exp(hi))
}

rate_converged <- function(ct) {
  r <- ct %>% filter(term %in% c("pandemic_dummy","war_dummy","postwar_dummy"))
  nrow(r)>0 && !any(r$irr_lo==0|is.infinite(r$irr_hi)|is.na(r$irr_hi))
}

build_formula <- function(ov, strategy="simple_primary", rlhs=NULL) {
  lhs <- if (is.null(rlhs)) ov else rlhs
  switch(strategy,
    simple_primary  = as.formula(paste0(lhs," ~ t + pandemic_dummy + war_dummy + postwar_dummy + sin_annual + cos_annual")),
    simple_no_season= as.formula(paste0(lhs," ~ t + pandemic_dummy + war_dummy + postwar_dummy")),
    full_with_slopes= as.formula(paste0(lhs," ~ t + pandemic_dummy + war_dummy + time_after_war + postwar_dummy + time_after_postwar + sin_annual + cos_annual")),
    stop("Unknown strategy"))
}

# ============================================================================
#                        DIAGNOSTIC PLOTS  (DHARMa-based)
# ============================================================================
# Produces 4 ggplot diagnostic panels per model; combined + individual saved

create_diagnostic_plots <- function(model, model_data, outcome_var, outcome_label,
                                    stratum_label=NULL) {
  plots <- list()
  tag <- paste(outcome_label, if (!is.null(stratum_label)) paste("-", stratum_label) else "")

  # 1. Residuals vs Time
  plots$resid_time <- tryCatch({
    rd <- data.frame(t=model_data$t, r=residuals(model, type="pearson"))
    ggplot(rd, aes(t,r)) + geom_point(alpha=0.6) +
      geom_hline(yintercept=0, linetype="dashed", color="red") +
      geom_smooth(method="loess", se=TRUE, color="#1976D2", fill="#BBDEFB") +
      labs(title="Pearson Residuals vs Time", subtitle=tag, x="Quarter", y="Residual") +
      theme_publication()
  }, error=function(e) NULL)

  # 2. Observed vs Fitted
  plots$obs_fitted <- tryCatch({
    fd <- data.frame(obs=model_data[[outcome_var]], fit=fitted(model))
    ggplot(fd, aes(fit,obs)) + geom_point(alpha=0.6, color="#1976D2", size=2.5) +
      geom_abline(slope=1, intercept=0, linetype="dashed", color="red") +
      geom_smooth(method="lm", se=TRUE, color="#4CAF50", fill="#C8E6C9") +
      labs(title="Observed vs Fitted", subtitle=tag, x="Fitted", y="Observed") +
```

© 2026 Kebede HK et al. *JAMA Network Open*.

```
    theme_publication()
  }, error=function(e) NULL)

  # 3. ACF of residuals
  plots$acf <- tryCatch({
    res <- residuals(model, type="pearson")
    av <- acf(res, plot=FALSE, lag.max=20)
    ad <- data.frame(lag=as.numeric(av$lag[-1]), acf=as.numeric(av$acf[-1]))
    ci <- 1.96/sqrt(length(res))
    ggplot(ad, aes(lag,acf)) +
      geom_hline(yintercept=c(-ci,ci), linetype="dashed", color="blue") +
      geom_hline(yintercept=0) +
      geom_segment(aes(xend=lag, yend=0), linewidth=1.2, color="#1976D2") +
      labs(title="Autocorrelation Function", subtitle=tag, x="Lag", y="ACF") +
      theme_publication()
  }, error=function(e) NULL)

  # 4. Residual histogram
  plots$resid_hist <- tryCatch({
    rd <- data.frame(r=residuals(model, type="pearson"))
    ggplot(rd, aes(r)) +
      geom_histogram(aes(y=after_stat(density)), bins=25, fill="#1976D2", alpha=0.7, color="white") +
      geom_density(color="#D32F2F", linewidth=1) +
      stat_function(fun=dnorm, args=list(mean=mean(rd$r), sd=sd(rd$r)),
                    color="#4CAF50", linewidth=1, linetype="dashed") +
      labs(title="Residual Distribution", subtitle=tag, x="Pearson Residual", y="Density") +
      theme_publication()
  }, error=function(e) NULL)

  plots
}

save_diagnostic_plots <- function(plots, outcome_label, stratum_label=NULL, analysis_type="main") {
  bn <- paste0(analysis_type, "_diag_", safe_name(outcome_label),
               if (!is.null(stratum_label)) paste0("_", safe_name(stratum_label)) else "")
  gp <- plots[sapply(plots, function(x) inherits(x, "gg"))]
  # Combined panel
  if (length(gp) >= 3) {
    comb <- tryCatch(wrap_plots(gp, ncol=2) +
      plot_annotation(title=paste("Diagnostics:", outcome_label,
                      if(!is.null(stratum_label)) paste("-",stratum_label) else "")),
      error=function(e) NULL)
    if (!is.null(comb)) tryCatch({
      ggsave(file.path(OUTPUT_DIR, "figures/diagnostics", paste0(bn,"_combined.pdf")),
             comb, width=14, height=12, device=cairo_pdf, bg="white")
      ggsave(file.path(OUTPUT_DIR, "figures/diagnostics", paste0(bn,"_combined.png")),
             comb, width=14, height=12, dpi=FIG_DPI, bg="white")
    }, error=function(e) NULL)
  }
}

# ============================================================================
#                        ITS MODEL FITTING
# ============================================================================

fit_its_model <- function(data, outcome_var, outcome_label, model_type="count",
                          denominator_var=NULL, stratifier=NULL, stratum_label=NULL,
```

© 2026 Kebede HK et al. *JAMA Network Open*.

```
                        analysis_type="main", strategy="simple_primary") {
  tryCatch({
    d <- data %>% filter(!is.na(.data[[outcome_var]])) %>% arrange(t)
    if (nrow(d) < 12) { log_message(paste("  SKIP:", outcome_label, "|", stratum_label %||% "all")); return(NULL) }
    lbl <- paste(outcome_label, if(!is.null(stratum_label)) paste("|",stratum_label) else "", "|", strategy, "|", model_type)

    if (model_type == "count") {
      forms <- switch(strategy,
        simple_primary   = list(simple_primary=build_formula(outcome_var,"simple_primary"),
                                simple_no_season=build_formula(outcome_var,"simple_no_season")),
        simple_no_season = list(simple_no_season=build_formula(outcome_var,"simple_no_season")),
        full_with_slopes = list(full_with_slopes=build_formula(outcome_var,"full_with_slopes")),
        stop("Unknown"))
      fo<-NULL; fu<-NULL
      for (nm in names(forms)) { fo<-fit_with_fallback(forms[[nm]], d, paste(lbl,"[",nm,"]")); if(!is.null(fo)){fu<-nm;break} }
      if (is.null(fo)) return(NULL)
    } else if (model_type=="rate" && !is.null(denominator_var)) {
      d <- d %>% mutate(success=pmax(0,.data[[outcome_var]]), total=pmax(0,.data[[denominator_var]]),
                        failure=pmax(0,total-success)) %>% filter(total>0)
      d$success <- pmin(d$success, d$total); d$failure <- d$total - d$success
      if (nrow(d)<12) return(NULL)
      fo <- fit_with_fallback(build_formula(NULL,"simple_primary",rlhs="cbind(success, failure)"),
                              d, paste(lbl,"[binomial]"), is_binomial=TRUE)
      if (is.null(fo)) return(NULL); fu<-"rate_binomial_simple"
    } else return(NULL)

    model<-fo$model; mfam<-fo$family
    ct <- tryCatch(extract_coefs(model), error=function(e) NULL)
    if (is.null(ct)||nrow(ct)==0) return(NULL)

    rok <- if(model_type=="rate") rate_converged(ct) else NA
    if(model_type=="rate"&&!isTRUE(rok)) log_message(paste("  NOTE: 0-Inf CIs |", lbl))

    pand<-ct%>%filter(term=="pandemic_dummy"); war<-ct%>%filter(term=="war_dummy"); post<-ct%>%filter(term=="postwar_dummy")
    if(nrow(pand)>0) log_message(sprintf("    Pandemic: IRR=%.3f (%.3f-%.3f), p=%.4f", pand$irr[1], pand$irr_lo[1], pand$irr_hi[1], pand$p.value[1]))
    if(nrow(war)>0)  log_message(sprintf("    War:      IRR=%.3f (%.3f-%.3f), p=%.4f", war$irr[1], war$irr_lo[1], war$irr_hi[1], war$p.value[1]))
    if(nrow(post)>0) log_message(sprintf("    Post:     IRR=%.3f (%.3f-%.3f), p=%.4f", post$irr[1], post$irr_lo[1], post$irr_hi[1], post$p.value[1]))

    # DHARMa diagnostics
    sim <- tryCatch(simulateResiduals(model, n=250, plot=FALSE), error=function(e) NULL)
    dp <- if(!is.null(sim)) tryCatch(testDispersion(sim, plot=FALSE)$p.value, error=function(e) NA_real_) else NA_real_
    ap <- if(!is.null(sim)) tryCatch(testTemporalAutocorrelation(sim,
      time=seq_along(sim$scaledResiduals), plot=FALSE)$p.value, error=function(e) NA_real_) else NA_real_

    # Diagnostic plots (overall models only to avoid 300+ files)
    if (is.null(stratum_label) || analysis_type == "main") {
      dplots <- create_diagnostic_plots(model, d, outcome_var, outcome_label, stratum_label)
      save_diagnostic_plots(dplots, outcome_label, stratum_label, analysis_type)
    }

    # Predictions & counterfactual
    fv <- tryCatch(predict(model, type="response"), error=function(e) rep(NA_real_, nrow(d)))
    cfd <- d %>% mutate(pandemic_dummy=0, war_dummy=0, postwar_dummy=0)
    if ("time_after_war" %in% names(cfd)) cfd <- cfd %>% mutate(time_after_war=0, time_after_postwar=0)
    cfv <- tryCatch(predict(model, newdata=cfd, type="response"), error=function(e) rep(NA_real_, nrow(d)))
    pd <- d %>% mutate(fitted=fv, counterfactual=cfv)
```

© 2026 Kebede HK et al. *JAMA Network Open*.

```
    # ITS plot
    yl <- if(model_type=="count") "Count (n)" else "Proportion"
    pp <- create_its_plot(pd, outcome_var, outcome_label, stratum_label, mfam, yl)

    # Save plot at 600 DPI
    if (!is.null(pp)) {
      bn <- paste0(analysis_type,"_its_",safe_name(outcome_label),
                   if(!is.null(stratum_label)) paste0("_",safe_name(stratum_label)) else "","_",model_type)
      tryCatch({
        ggsave(file.path(OUTPUT_DIR,"figures/publication",paste0(bn,".pdf")), pp, width=11, height=6.5, device=cairo_pdf, bg="white")
        ggsave(file.path(OUTPUT_DIR,"figures/publication",paste0(bn,".png")), pp, width=11, height=6.5, dpi=FIG_DPI, bg="white")
      }, error=function(e) log_message(paste("  WARN plot:", conditionMessage(e))))
    }

    # Save RDS
    tryCatch(saveRDS(model, file.path(OUTPUT_DIR,
      if(analysis_type=="main")"rds/main" else "rds/sensitivity",
      paste0(analysis_type,"_",safe_name(outcome_label),
             if(!is.null(stratum_label)) paste0("_",safe_name(stratum_label)) else "","_",model_type,".rds"))),
      error=function(e) NULL)

    list(model=model, coefficients=ct, pandemic=pand, war=war, postwar=post,
         diagnostics=list(dispersion_p=dp, autocorr_p=ap), predictions=pd, plot=pp,
         info=list(outcome=outcome_label, stratifier=ifelse(is.null(stratifier),"overall",stratifier),
                   stratum=ifelse(is.null(stratum_label),"all",stratum_label), model_type=model_type,
                   family=mfam, formula=fu, n_obs=nrow(d),
                   aic=tryCatch(AIC(model), error=function(e) NA_real_),
                   analysis_type=analysis_type, rate_converged=rok))
  }, error=function(e) { log_message(paste("  ERROR:", outcome_label, conditionMessage(e))); NULL })
}

# ============================================================================
#                        PLOTTING FUNCTIONS
# ============================================================================

create_descriptive_plot <- function(data, ov, ol, sl=NULL) {
  d <- data %>% filter(!is.na(.data[[ov]]))
  if(nrow(d)==0) return(NULL)
  pt<-min(d$t[d$pandemic_dummy==1]); wt<-min(d$t[d$war_dummy==1]); ht<-min(d$t[d$postwar_dummy==1])
  d <- d %>% mutate(pl=factor(period, levels=c("pre","pandemic","war","post"),
                              labels=c("Pre-war","Pandemic","War","Post-war")))
  ggplot(d, aes(t, !!sym(ov))) +
    annotate("rect",xmin=-Inf,xmax=pt-0.5,ymin=-Inf,ymax=Inf,fill="#E8F5E9",alpha=.3) +
    annotate("rect",xmin=pt-0.5,xmax=wt-0.5,ymin=-Inf,ymax=Inf,fill="#FFF3E0",alpha=.3) +
    annotate("rect",xmin=wt-0.5,xmax=ht-0.5,ymin=-Inf,ymax=Inf,fill="#FFEBEE",alpha=.3) +
    annotate("rect",xmin=ht-0.5,xmax=Inf,ymin=-Inf,ymax=Inf,fill="#E3F2FD",alpha=.3) +
    geom_vline(xintercept=c(pt,wt,ht)-0.5, color=c(intervention_colors["pandemic"],
               intervention_colors["war"], intervention_colors["postwar"]), linewidth=1) +
    geom_line(aes(color=pl), linewidth=1) +
    geom_point(aes(fill=pl), shape=21, size=2.5, color="white", stroke=1) +
    scale_color_manual(values=period_colors, name="Period") +
    scale_fill_manual(values=period_colors, name="Period") +
    scale_x_continuous(breaks=seq(min(d$t),max(d$t),by=4),
      labels=function(x){i<-match(x,d$t); ifelse(is.na(i),"",paste0(d$year[i],"\nQ",d$quarter[i]))}) +
    scale_y_continuous(labels=comma_format(), expand=expansion(mult=c(.05,.1))) +
    labs(title=if(is.null(sl)) ol else paste(ol,"-",sl),
         subtitle="Quarterly trends: pre-war, pandemic, war, post-war",
```

© 2026 Kebede HK et al. *JAMA Network Open*.

```
        x="Time period", y="Count (n)",
        caption="Q1 2005-Q1 2025; Q2 2025 excluded") + theme_publication()
}

create_its_plot <- function(pd, ov, ol, sl=NULL, mf="NB", yl="Count (n)") {
  if(is.null(pd)||nrow(pd)==0) return(NULL)
  pt<-min(pd$t[pd$pandemic_dummy==1]); wt<-min(pd$t[pd$war_dummy==1]); ht<-min(pd$t[pd$postwar_dummy==1])
  pr2 <- tryCatch(cor(pd[[ov]], pd$fitted, use="complete.obs")^2, error=function(e) NA_real_)
  ggplot(pd, aes(x=t)) +
    annotate("rect",xmin=-Inf,xmax=pt-0.5,ymin=-Inf,ymax=Inf,fill="#E8F5E9",alpha=.2) +
    annotate("rect",xmin=pt-0.5,xmax=wt-0.5,ymin=-Inf,ymax=Inf,fill="#FFF3E0",alpha=.2) +
    annotate("rect",xmin=wt-0.5,xmax=ht-0.5,ymin=-Inf,ymax=Inf,fill="#FFEBEE",alpha=.2) +
    annotate("rect",xmin=ht-0.5,xmax=Inf,ymin=-Inf,ymax=Inf,fill="#E3F2FD",alpha=.2) +
    geom_vline(xintercept=c(pt,wt,ht)-0.5, color=c(intervention_colors["pandemic"],
               intervention_colors["war"], intervention_colors["postwar"]), linewidth=1) +
    geom_line(aes(y=counterfactual, linetype="Counterfactual"), color=fitted_colors["Counterfactual"], linewidth=1) +
    geom_line(aes(y=fitted, linetype="Fitted model"), color=fitted_colors["Fitted"], linewidth=1) +
    geom_point(aes(y=!!sym(ov), shape="Observed"), color=fitted_colors["Observed"], size=2.5, alpha=.7) +
    scale_linetype_manual(name=NULL, values=c("Fitted model"="solid","Counterfactual"="dashed")) +
    scale_shape_manual(name=NULL, values=c("Observed"=16)) +
    scale_x_continuous(breaks=seq(min(pd$t),max(pd$t),by=4),
      labels=function(x){i<-match(x,pd$t); ifelse(is.na(i),"",paste0(pd$year[i],"\nQ",pd$quarter[i]))}) +
    scale_y_continuous(labels=comma_format(), expand=expansion(mult=c(.05,.15))) +
    labs(title=if(is.null(sl)) paste("Segmented Regression ITS:",ol)
              else paste("Segmented Regression ITS:",ol,"-",sl),
        subtitle=if(!is.na(pr2)) sprintf("%s (pseudo R\u00b2 = %.3f); 4-period model", mf, pr2) else mf,
        x="Time period", y=yl, caption="Blue: fitted. Gray dashed: counterfactual. Points: observed.") +
    theme_publication()
}

# ============================================================================
#                        ARIMA WITH INTERVENTION REGRESSORS
# ============================================================================

fit_arima_model <- function(data, ov, ol) {
  log_message(paste("  ARIMA:", ol))
  d <- data %>% filter(!is.na(.data[[ov]])) %>% arrange(t)
  y <- ts(d[[ov]], frequency=4)
  xr <- cbind(pandemic=d$pandemic_dummy, war=d$war_dummy, postwar=d$postwar_dummy)
  m <- tryCatch(auto.arima(y, xreg=xr, seasonal=TRUE, stepwise=FALSE,
                          approximation=FALSE, max.p=4, max.q=4), error=function(e) NULL)
  if (is.null(m)) { log_message(paste("    ARIMA FAILED:", ol)); return(NULL) }
  log_message(sprintf("    ARIMA(%d,%d,%d)(%d,%d,%d)[4] AICc=%.1f",
                    m$arma[1],m$arma[6],m$arma[2], m$arma[3],m$arma[7],m$arma[4], m$aicc))
  co<-coef(m); se<-sqrt(diag(m$var.coef)); nms<-names(co)
  ii<-which(nms %in% c("pandemic","war","postwar"))
  it <- if(length(ii)>0) tibble(term=nms[ii], estimate=co[ii], std.error=se[ii],
    z=co[ii]/se[ii], p.value=2*pnorm(abs(co[ii]/se[ii]), lower.tail=FALSE),
    conf.low=co[ii]-1.96*se[ii], conf.high=co[ii]+1.96*se[ii]) else NULL
  for(i in seq_along(ii)) log_message(sprintf("    %s: coef=%.1f (%.1f to %.1f), p=%.4f",
    nms[ii[i]], co[ii[i]], co[ii[i]]-1.96*se[ii[i]], co[ii[i]]+1.96*se[ii[i]], it$p.value[i]))

  # Counterfactual
  cfx<-xr; cfx[,]<-0
  cfp <- tryCatch({cf<-Arima(y, model=m, xreg=cfx); as.numeric(fitted(cf))}, error=function(e) rep(NA_real_,length(y)))
  pd <- d %>% mutate(fitted_arima=as.numeric(fitted(m)), counterfactual_arima=cfp)
```

© 2026 Kebede HK et al. *JAMA Network Open*.

```
  # ARIMA ITS plot
  pt<-min(d$t[d$pandemic_dummy==1]); wt<-min(d$t[d$war_dummy==1]); ht<-min(d$t[d$postwar_dummy==1])
  p <- ggplot(pd, aes(x=t)) +
    annotate("rect",xmin=-Inf,xmax=pt-0.5,ymin=-Inf,ymax=Inf,fill="#E8F5E9",alpha=.2) +
    annotate("rect",xmin=pt-0.5,xmax=wt-0.5,ymin=-Inf,ymax=Inf,fill="#FFF3E0",alpha=.2) +
    annotate("rect",xmin=wt-0.5,xmax=ht-0.5,ymin=-Inf,ymax=Inf,fill="#FFEBEE",alpha=.2) +
    annotate("rect",xmin=ht-0.5,xmax=Inf,ymin=-Inf,ymax=Inf,fill="#E3F2FD",alpha=.2) +
    geom_vline(xintercept=c(pt,wt,ht)-0.5, color=c(intervention_colors["pandemic"],
               intervention_colors["war"],intervention_colors["postwar"]), linewidth=1) +
    geom_line(aes(y=counterfactual_arima, linetype="Counterfactual"), color="#757575", linewidth=1) +
    geom_line(aes(y=fitted_arima, linetype="ARIMA fitted"), color="#E65100", linewidth=1) +
    geom_point(aes(y=!!sym(ov)), color="black", size=2, alpha=.6) +
    scale_linetype_manual(name=NULL, values=c("ARIMA fitted"="solid","Counterfactual"="dashed")) +
    scale_x_continuous(breaks=seq(min(d$t),max(d$t),by=4),
      labels=function(x){i<-match(x,d$t); ifelse(is.na(i),"",paste0(d$year[i],"\nQ",d$quarter[i]))}) +
    scale_y_continuous(labels=comma_format()) +
    labs(title=paste("ARIMA with Intervention Regressors:",ol),
         subtitle=sprintf("ARIMA(%d,%d,%d)(%d,%d,%d)[4]; AICc=%.1f",
                          m$arma[1],m$arma[6],m$arma[2],m$arma[3],m$arma[7],m$arma[4],m$aicc),
         x="Time period", y="Count (n)") + theme_publication()

  # ACF diagnostic
  pdiag <- tryCatch({
    ra<-residuals(m); rd<-tibble(t=seq_along(ra),r=as.numeric(ra))
    av<-acf(ra,plot=FALSE,lag.max=20); ad<-tibble(lag=as.numeric(av$lag[-1]),acf=as.numeric(av$acf[-1]))
    ci<-1.96/sqrt(length(ra))
    p1<-ggplot(rd,aes(t,r))+geom_line(color="gray40")+geom_point(size=1.5)+
      geom_hline(yintercept=0,color="red",linetype="dashed")+labs(title="ARIMA Residuals",x="Quarter",y="Residual")+theme_publication()
    p2<-ggplot(ad,aes(lag,acf))+geom_hline(yintercept=c(-ci,ci),color="blue",linetype="dashed")+
      geom_hline(yintercept=0)+geom_segment(aes(xend=lag,yend=0),linewidth=1.5)+
      labs(title="ACF of ARIMA Residuals",x="Lag",y="ACF")+theme_publication()
    p1/p2
  }, error=function(e) NULL)

  fn <- safe_name(ol)
  tryCatch({
    ggsave(file.path(OUTPUT_DIR,"figures/arima",paste0("arima_its_",fn,".pdf")), p, width=11, height=6.5, device=cairo_pdf, bg="white")
    ggsave(file.path(OUTPUT_DIR,"figures/arima",paste0("arima_its_",fn,".png")), p, width=11, height=6.5, dpi=FIG_DPI, bg="white")
    if(!is.null(pdiag)){
      ggsave(file.path(OUTPUT_DIR,"figures/arima",paste0("arima_diag_",fn,".pdf")), pdiag, width=10, height=10, device=cairo_pdf, bg="white")
      ggsave(file.path(OUTPUT_DIR,"figures/arima",paste0("arima_diag_",fn,".png")), pdiag, width=10, height=10, dpi=FIG_DPI, bg="white")
    }
  }, error=function(e) log_message(paste("  WARN ARIMA plot:", conditionMessage(e))))

  list(model=m, coefficients=it, predictions=pd, plot=p,
       info=list(outcome=ol, order=m$arma, aicc=m$aicc))
}

# ============================================================================
#                        ANALYSIS RUNNER
# ============================================================================

run_its_analysis <- function(data, ol, at="main", st="simple_primary") {
  log_message(paste("\n=== Running", toupper(at), "[", st, "] ==="))
  res <- list()
  for (on in names(ol)) {
    log_message(paste("\n--- Processing:", on, "---"))
```

© 2026 Kebede HK et al. *JAMA Network Open*.

```
    oi<-ol[[on]]; tv<-oi$total; dv<-oi$denominator; lb<-oi$label
    if(!(tv %in% names(data))){log_message(paste("  SKIP:",tv)); next}
    if(at=="main"){
      dp<-create_descriptive_plot(data, tv, lb)
      if(!is.null(dp)) tryCatch({
        fn<-paste0("descriptive_",safe_name(on),"_total")
        ggsave(file.path(OUTPUT_DIR,"figures/descriptive",paste0(fn,".pdf")), dp, width=11,height=6.5, device=cairo_pdf, bg="white")
        ggsave(file.path(OUTPUT_DIR,"figures/descriptive",paste0(fn,".png")), dp, width=11,height=6.5, dpi=FIG_DPI, bg="white")
      }, error=function(e) NULL)
    }
    res[[paste0(on,"__overall__count")]] <- fit_its_model(data, tv, lb, "count", analysis_type=at, strategy=st)
    if(!is.null(dv)) res[[paste0(on,"__overall__rate")]] <- fit_its_model(data, tv, paste(lb,"Rate"), "rate", denominator_var=dv, analysis_type=at, strategy=st)
    for(sn in names(oi$strata)){
      log_message(paste("  Stratifier:",sn))
      for(lv in names(oi$strata[[sn]])){
        vr<-oi$strata[[sn]][[lv]]; if(!(vr %in% names(data))) next
        res[[paste0(on,"__",sn,"__",lv,"__count")]] <- fit_its_model(data, vr, lb, "count", stratifier=sn, stratum_label=lv, analysis_type=at, strategy=st)
        if(!is.null(dv)){
          ds <- if(on=="linkage") str_replace(vr,"^lx_","dx_") else if(on=="initiation") str_replace(vr,"^rx_","lx_") else NULL
          if(!is.null(ds)&&ds %in% names(data))
            res[[paste0(on,"__",sn,"__",lv,"__rate")]] <- fit_its_model(data, vr, paste(lb,"Rate"), "rate", denominator_var=ds, stratifier=sn, stratum_label=lv,
analysis_type=at, strategy=st)
        }
      }
    }
  }
  res
}

# ============================================================================
#                        RESULTS COMPILATION
# ============================================================================

fmt_p <- function(p) ifelse(is.na(p), NA_character_,
  ifelse(p < 0.001, "<.001",
    ifelse(p > 0.99, ">.99",
      ifelse(p < 0.01, sprintf("%.3f", p), sprintf("%.2f", p)))))

sig_star <- function(p) ifelse(is.na(p),"", ifelse(p<0.001,"***", ifelse(p<0.01,"**", ifelse(p<0.05,"*",""))))

compile_results <- function(rl, at="main") {
  rows <- lapply(names(rl), function(rn){
    r<-rl[[rn]]; if(is.null(r)||is.null(r$coefficients)||nrow(r$coefficients)==0) return(NULL)
    pa<-r$coefficients%>%filter(term=="pandemic_dummy"); wa<-r$coefficients%>%filter(term=="war_dummy"); po<-r$coefficients%>%filter(term=="postwar_dummy")
    tibble(analysis_type=at, model_id=rn, outcome=r$info$outcome, stratifier=r$info$stratifier, stratum=r$info$stratum,
      model_type=r$info$model_type, family=r$info$family, formula=r$info$formula, n_obs=r$info$n_obs, aic=r$info$aic,
      rate_converged=r$info$rate_converged%||%NA,
      pandemic_irr=if(nrow(pa)>0)pa$irr[1] else NA_real_, pandemic_lo=if(nrow(pa)>0)pa$irr_lo[1] else NA_real_,
      pandemic_hi=if(nrow(pa)>0)pa$irr_hi[1] else NA_real_, pandemic_p=if(nrow(pa)>0)pa$p.value[1] else NA_real_,
      war_irr=if(nrow(wa)>0)wa$irr[1] else NA_real_, war_lo=if(nrow(wa)>0)wa$irr_lo[1] else NA_real_,
      war_hi=if(nrow(wa)>0)wa$irr_hi[1] else NA_real_, war_p=if(nrow(wa)>0)wa$p.value[1] else NA_real_,
      postwar_irr=if(nrow(po)>0)po$irr[1] else NA_real_, postwar_lo=if(nrow(po)>0)po$irr_lo[1] else NA_real_,
      postwar_hi=if(nrow(po)>0)po$irr_hi[1] else NA_real_, postwar_p=if(nrow(po)>0)po$p.value[1] else NA_real_,
      dispersion_p=r$diagnostics$dispersion_p, autocorr_p=r$diagnostics$autocorr_p)
  })
  bind_rows(rows) %>% mutate(
    pandemic_result=sprintf("%.2f (%.2f-%.2f)%s", pandemic_irr, pandemic_lo, pandemic_hi, sig_star(pandemic_p)),
```

© 2026 Kebede HK et al. *JAMA Network Open*.

```
    war_result=sprintf("%.2f (%.2f-%.2f)%s", war_irr, war_lo, war_hi, sig_star(war_p)),
    postwar_result=sprintf("%.2f (%.2f-%.2f)%s", postwar_irr, postwar_lo, postwar_hi, sig_star(postwar_p)),
    pandemic_p_fmt=fmt_p(pandemic_p), war_p_fmt=fmt_p(war_p), postwar_p_fmt=fmt_p(postwar_p))
}

extract_all_coefs <- function(rl, at="main") {
  bind_rows(lapply(names(rl), function(rn){
    r<-rl[[rn]]; if(is.null(r)||is.null(r$coefficients)) return(NULL)
    r$coefficients %>% mutate(analysis_type=at, model_id=rn, outcome=r$info$outcome,
      stratifier=r$info$stratifier, stratum=r$info$stratum) %>%
      select(analysis_type, model_id, outcome, stratifier, stratum, everything())
  }))
}

# ============================================================================
#                DESCRIPTIVE STATS  (from original: trend + contribution)
# ============================================================================

create_descriptive_stats <- function(data, ol) {
  log_message("Creating descriptive statistics...")
  ps <- data %>% mutate(pl=factor(period, levels=c("pre","pandemic","war","post"),
    labels=c("Pre-war","Pandemic","War","Post-war"))) %>%
    group_by(pl) %>% summarise(n_quarters=n(), start=min(date_label), end=max(date_label), .groups="drop")

  one_sum <- function(d, vr, lb, st, sl) {
    d %>% mutate(pl=factor(period, levels=c("pre","pandemic","war","post"),
      labels=c("Pre-war","Pandemic","War","Post-war"))) %>% group_by(pl) %>%
      summarise(outcome=lb, stratifier=st, stratum=sl, n_quarters=n(),
        mean_q=mean(.data[[vr]],na.rm=T), median_q=median(.data[[vr]],na.rm=T),
        sd_q=sd(.data[[vr]],na.rm=T), q25=quantile(.data[[vr]],.25,na.rm=T),
        q75=quantile(.data[[vr]],.75,na.rm=T), iqr=q75-q25,
        min_q=min(.data[[vr]],na.rm=T), max_q=max(.data[[vr]],na.rm=T),
        total=sum(.data[[vr]],na.rm=T), cv_pct=(sd_q/mean_q)*100, .groups="drop") %>%
      mutate(pre_mean=mean_q[pl=="Pre-war"][1], pre_total=total[pl=="Pre-war"][1],
             rel_change_mean_pct=(mean_q-pre_mean)/pre_mean*100,
             rel_change_total_pct=(total-pre_total)/pre_total*100)
  }

  os <- list()
  for(on in names(ol)){
    oi<-ol[[on]]; if(!(oi$total %in% names(data))) next
    os[[paste0(on,"_overall")]] <- one_sum(data, oi$total, oi$label, "Overall", "All")
    for(sn in names(oi$strata)) for(lv in names(oi$strata[[sn]])){
      vr<-oi$strata[[sn]][[lv]]; if(vr %in% names(data)) os[[paste0(on,"_",sn,"_",lv)]] <- one_sum(data, vr, oi$label, sn, lv)
    }
  }

  # Pre-war trend analysis (check baseline stability)
  tr <- list()
  for(on in names(ol)){
    oi<-ol[[on]]; tv<-oi$total; if(!(tv %in% names(data))) next
    pw <- data %>% filter(period=="pre")
    if(nrow(pw)>=3){
      tm <- lm(as.formula(paste0(tv," ~ t")), data=pw)
      sc <- summary(tm)$coefficients
      tr[[on]] <- tibble(outcome=oi$label, slope=sc[2,1], slope_se=sc[2,2], slope_p=sc[2,4],
        r_squared=summary(tm)$r.squared,
```

© 2026 Kebede HK et al. *JAMA Network Open*.

```
      trend=case_when(sc[2,1]>0&sc[2,4]<.05~"Increasing", sc[2,1]<0&sc[2,4]<.05~"Decreasing", TRUE~"Stable"))
    }
  }

  # Contribution analysis (% of total by stratum per period)
  ca <- list()
  for(on in names(ol)){
    oi<-ol[[on]]; tv<-oi$total
    for(sn in names(oi$strata)){
      sv<-oi$strata[[sn]]
      for(lv in names(sv)){
        vr<-sv[[lv]]; if(!(vr %in% names(data))) next
        cd <- data %>% mutate(pl=factor(period, levels=c("pre","pandemic","war","post"),
          labels=c("Pre-war","Pandemic","War","Post-war"))) %>% group_by(pl) %>%
          summarise(stratum_total=sum(.data[[vr]],na.rm=T), overall_total=sum(.data[[tv]],na.rm=T), .groups="drop") %>%
          mutate(outcome=oi$label, stratifier=sn, stratum=lv, pct=stratum_total/overall_total*100)
        ca[[paste0(on,"_",sn,"_",lv)]] <- cd
      }
    }
  }

  list(period_summary=ps, outcome_statistics=bind_rows(os),
       trend_analysis=bind_rows(tr), contribution_analysis=bind_rows(ca))
}

# ============================================================================
#                         INTERACTION TESTS
# ============================================================================

run_interaction_tests <- function(data, ol) {
  log_message("\n=== Interaction tests (period x stratifier) ===")
  rows <- list()
  for(s in c("sex","age","ownership","facility_level")) for(o in names(ol)){
    oi<-ol[[o]]; if(!(s %in% names(oi$strata))) next; vs<-oi$strata[[s]]
    lng <- bind_rows(lapply(names(vs), function(lv) data %>% mutate(stratum=lv, count=.data[[vs[[lv]]]]) %>%
      select(t,period,pandemic_dummy,war_dummy,postwar_dummy,sin_annual,cos_annual,stratum,count)))
    lng$stratum <- factor(lng$stratum, levels=names(vs))
    mm <- tryCatch(MASS::glm.nb(count~t+pandemic_dummy+war_dummy+postwar_dummy+sin_annual+cos_annual+stratum, data=lng), error=function(e) NULL)
    mi <- tryCatch(MASS::glm.nb(count~t+pandemic_dummy*stratum+war_dummy*stratum+postwar_dummy*stratum+sin_annual+cos_annual, data=lng), error=function(e) NULL)
    if(is.null(mm)||is.null(mi)) next
    lr <- tryCatch(anova(mm, mi), error=function(e) NULL); if(is.null(lr)) next
    rows[[length(rows)+1]] <- tibble(outcome=oi$label, stratifier=s, test="LR: period x stratum",
      chisq=lr$`LR stat.`[2], df=lr$`   df`[2], p_value=lr$`Pr(Chi)`[2], p_fmt=fmt_p(lr$`Pr(Chi)`[2]))
  }
  bind_rows(rows)
}

# ============================================================================
#                         CUMULATIVE DEFICITS  (bootstrap CIs)
# ============================================================================

compute_cumulative_deficits <- function(data, ol, R=5000, run_boot=TRUE) {
  log_message(sprintf("\n=== Cumulative deficits (R=%d) ===", if(run_boot) R else 0))
  out <- list()
  for(on in names(ol)){
    oi<-ol[[on]]; vr<-oi$total; if(!(vr %in% names(data))) next
    d<-data %>% filter(!is.na(.data[[vr]])); f<-build_formula(vr,"simple_primary")
```

© 2026 Kebede HK et al. *JAMA Network Open*.

```
    m<-tryCatch(suppressWarnings(glmmTMB(f,data=d,family=nbinom2())), error=function(e) NULL); ug<-FALSE
    if(!glmmtmb_ok(m)){m<-tryCatch(glm(f,family=quasipoisson(),data=d), error=function(e) NULL); ug<-TRUE}
    if(is.null(m)){log_message(paste("  SKIP deficit:",oi$label)); next}
    cfd<-d%>%mutate(pandemic_dummy=0,war_dummy=0,postwar_dummy=0)
    cf<-tryCatch(predict(m,newdata=cfd,type="response"), error=function(e) rep(NA_real_,nrow(d)))
    obs<-d[[vr]]; wi<-which(d$period=="war"); pi<-which(d$period=="post")
    pw<-sum(cf[wi]-obs[wi],na.rm=T); pp<-sum(cf[pi]-obs[pi],na.rm=T); pt<-pw+pp
    bl<-NA_real_; bh<-NA_real_
    if(run_boot&&!ug&&inherits(m,"glmmTMB")){
      mu<-predict(m,type="response"); th<-sigma(m)
      log_message(sprintf("  Bootstrapping %s (R=%d)...", oi$label, R))
      boots<-replicate(R, {
        ys<-rnegbin(length(mu),mu=mu,theta=th); ds<-d; ds[[vr]]<-ys
        ms<-tryCatch(suppressWarnings(glmmTMB(f,data=ds,family=nbinom2())), error=function(e) NULL)
        if(!glmmtmb_ok(ms)) return(NA_real_)
        cfs<-predict(ms,newdata=cfd,type="response")
        sum(cfs[c(wi,pi)]-ys[c(wi,pi)], na.rm=TRUE)
      })
      qs<-quantile(boots, c(.025,.975), na.rm=TRUE); bl<-qs[1]; bh<-qs[2]
    }
    out[[on]]<-tibble(outcome=oi$label, n_war=length(wi), n_post=length(pi),
      deficit_war=pw, deficit_post=pp, deficit_total=pt, boot_lo=bl, boot_hi=bh,
      method=if(ug)"Quasi-Poisson" else if(run_boot)"NB+bootstrap" else "NB")
    log_message(sprintf("  %s: war=%.0f post=%.0f total=%.0f (CI %s)", oi$label, pw, pp, pt,
      if(is.na(bl))"NA" else sprintf("%.0f to %.0f",bl,bh)))
  }
  bind_rows(out)
}

# ============================================================================
#                        EXECUTION
# ============================================================================

# --- STEP 2: MAIN ANALYSIS ---
log_message("\n========== STEP 2: MAIN ANALYSIS ==========")
results_main <- run_its_analysis(analysis_data, outcomes, "main", "simple_primary")
main_summary <- compile_results(results_main, "Main")
main_coefs   <- extract_all_coefs(results_main, "Main")
main_desc    <- create_descriptive_stats(analysis_data, outcomes)
write_csv(main_summary, file.path(OUTPUT_DIR,"tables","Table01_Main_Summary.csv"))
write_csv(main_coefs,   file.path(OUTPUT_DIR,"tables","Table02_All_Coefficients.csv"))
write_csv(main_desc$period_summary, file.path(OUTPUT_DIR,"tables","Table03A_Period_Summary.csv"))
write_csv(main_desc$outcome_statistics, file.path(OUTPUT_DIR,"tables","Table03B_Outcome_Statistics.csv"))
write_csv(main_desc$trend_analysis, file.path(OUTPUT_DIR,"tables","Table03C_PreWar_Trends.csv"))
write_csv(main_desc$contribution_analysis, file.path(OUTPUT_DIR,"tables","Table03D_Contribution_Analysis.csv"))
n_main <- sum(!sapply(results_main, is.null))
log_message(paste("Main complete:", n_main, "models"))

# --- STEP 3: ARIMA ---
log_message("\n========== STEP 3: ARIMA ==========")
arima_results <- list()
for(on in names(outcomes)) arima_results[[on]] <- fit_arima_model(analysis_data, outcomes[[on]]$total, outcomes[[on]]$label)
arima_summary <- bind_rows(lapply(names(arima_results), function(nm){
  r<-arima_results[[nm]]; if(is.null(r)) return(NULL); ct<-r$coefficients
  tibble(outcome=r$info$outcome, arima_order=paste0("(",r$info$order[1],",",r$info$order[6],",",r$info$order[2],
    ")(",r$info$order[3],",",r$info$order[7],",",r$info$order[4],")[4]"), aicc=r$info$aicc,
    pandemic_coef=if(!is.null(ct)&&"pandemic"%in%ct$term) ct$estimate[ct$term=="pandemic"] else NA_real_,
```

© 2026 Kebede HK et al. *JAMA Network Open*.

```
    pandemic_p=if(!is.null(ct)&&"pandemic"%in%ct$term) ct$p.value[ct$term=="pandemic"] else NA_real_,
    war_coef=if(!is.null(ct)&&"war"%in%ct$term) ct$estimate[ct$term=="war"] else NA_real_,
    war_p=if(!is.null(ct)&&"war"%in%ct$term) ct$p.value[ct$term=="war"] else NA_real_,
    postwar_coef=if(!is.null(ct)&&"postwar"%in%ct$term) ct$estimate[ct$term=="postwar"] else NA_real_,
    postwar_p=if(!is.null(ct)&&"postwar"%in%ct$term) ct$p.value[ct$term=="postwar"] else NA_real_)
}))
write_csv(arima_summary, file.path(OUTPUT_DIR,"tables","Table04_ARIMA_Summary.csv"))

# --- STEP 4: SENSITIVITY ---
if (RUN_SENSITIVITY) {
  log_message("\n========== STEP 4: SENSITIVITY ==========")

  log_message("\n--- Sens 1: No seasonality ---")
  sum_s1 <- compile_results(run_its_analysis(analysis_data, outcomes, "sens1_no_season", "simple_no_season"), "NoSeason")
  write_csv(sum_s1, file.path(OUTPUT_DIR,"tables","Table05_Sens_NoSeason.csv"))

  log_message("\n--- Sens 2: Full slopes ---")
  sum_s2 <- compile_results(run_its_analysis(analysis_data, outcomes, "sens2_full_slopes", "full_with_slopes"), "FullSlopes")
  write_csv(sum_s2, file.path(OUTPUT_DIR,"tables","Table06_Sens_FullSlopes.csv"))

  log_message("\n--- Sens 3: Alt war onset Q4 2020 (FIXED: pandemic=Q1-Q3 only) ---")
  data_alt <- clean_data_4period(raw_data, war_start_year=2020, war_start_quarter=4)
  log_message(paste("  Sens3 periods:", paste(names(table(data_alt$period)), table(data_alt$period), sep="=", collapse=", ")))
  sum_s3 <- compile_results(run_its_analysis(data_alt, outcomes, "sens3_alt_onset", "simple_primary"), "AltOnset")
  write_csv(sum_s3, file.path(OUTPUT_DIR,"tables","Table07_Sens_AltOnset.csv"))

  log_message("\n--- Sens 4: Pre-war 2015+  ---")
  data_rec <- analysis_data %>% filter(period!="pre"|year>=2015) %>% arrange(year,quarter) %>%
    mutate(t=row_number(), sin_annual=sin(2*pi*t/4), cos_annual=cos(2*pi*t/4))
  sum_s4 <- compile_results(run_its_analysis(data_rec, outcomes, "sens4_recent", "simple_primary"), "Recent")
  write_csv(sum_s4, file.path(OUTPUT_DIR,"tables","Table08_Sens_Recent.csv"))
} else { sum_s1<-sum_s2<-sum_s3<-sum_s4<-NULL }

# --- STEP 5: INTERACTIONS  ---
int_results <- run_interaction_tests(analysis_data, outcomes)
write_csv(int_results, file.path(OUTPUT_DIR,"tables","Table09_Interaction_Tests.csv"))

# --- STEP 6: DEFICITS ---
deficits <- compute_cumulative_deficits(analysis_data, outcomes,
  R=if(RUN_BOOTSTRAP) BOOT_R else 100, run_boot=RUN_BOOTSTRAP)
write_csv(deficits, file.path(OUTPUT_DIR,"tables","Table10_Cumulative_Deficits.csv"))

# --- STEP 7: COMBINED FIGURES ---
log_message("\n=== Combined publication figures ===")
mp <- list(); for(on in c("diagnosis","linkage","initiation")){
  rk<-paste0(on,"__overall__count")
  if(!is.null(results_main[[rk]])&&!is.null(results_main[[rk]]$plot)) mp[[on]]<-results_main[[rk]]$plot
}
if(length(mp)==3){
  comb<-(mp$diagnosis+labs(title="A. HIV Diagnoses"))/(mp$linkage+labs(title="B. Linkage to Care"))/(mp$initiation+labs(title="C. ART Initiation"))
  tryCatch({
    ggsave(file.path(OUTPUT_DIR,"figures/publication","Figure2_Cascade_Combined.pdf"), comb, width=11, height=16, device=cairo_pdf, bg="white")
    ggsave(file.path(OUTPUT_DIR,"figures/publication","Figure2_Cascade_Combined.png"), comb, width=11, height=16, dpi=FIG_DPI, bg="white")
    log_message("Combined NB figure saved")
  }, error=function(e) log_message(paste("WARN:",conditionMessage(e))))
}
# Combined ARIMA
```

© 2026 Kebede HK et al. *JAMA Network Open*.

```
ap <- list(); for(on in names(arima_results)) if(!is.null(arima_results[[on]]$plot)) ap[[on]]<-arima_results[[on]]$plot
if(length(ap)==3){
  ca<-(ap$diagnosis+labs(title="A. HIV Diagnoses"))/(ap$linkage+labs(title="B. Linkage to Care"))/(ap$initiation+labs(title="C. ART Initiation"))
  tryCatch({
    ggsave(file.path(OUTPUT_DIR,"figures/arima","Figure_ARIMA_Combined.pdf"), ca, width=11, height=16, device=cairo_pdf, bg="white")
    ggsave(file.path(OUTPUT_DIR,"figures/arima","Figure_ARIMA_Combined.png"), ca, width=11, height=16, dpi=FIG_DPI, bg="white")
    log_message("Combined ARIMA figure saved")
  }, error=function(e) log_message(paste("WARN:",conditionMessage(e))))
}

# --- STEP 8: EXCEL ---
log_message("\n=== Excel workbook ===")
wb<-createWorkbook()
hdr<-createStyle(fontSize=11, fontColour="#FFFFFF", halign="center", fgFill="#2C5282", textDecoration="bold")
add_s <- function(nm, dt) {
  if(is.null(dt)||(is.data.frame(dt)&&nrow(dt)==0)) return(invisible(NULL))
  addWorksheet(wb, nm); writeData(wb, nm, dt, headerStyle=hdr); setColWidths(wb, nm, 1:ncol(dt), widths="auto")
}
add_s("01_Main_Summary", main_summary)
add_s("02_Pub_Table", main_summary %>% filter(model_type=="count") %>%
  transmute(outcome,stratifier,stratum,family,aic,
    pandemic_effect=pandemic_result, pandemic_p_fmt, war_effect=war_result, war_p_fmt,
    post_effect=postwar_result, postwar_p_fmt))
add_s("03_Rate_Models", main_summary %>% filter(model_type=="rate") %>%
  transmute(outcome,stratifier,stratum,family,rate_converged,
    pandemic_effect=pandemic_result, pandemic_p_fmt, war_effect=war_result, war_p_fmt,
    post_effect=postwar_result, postwar_p_fmt))
add_s("04_All_Coefficients", main_coefs)
add_s("05_Period_Summary", main_desc$period_summary)
add_s("06_Outcome_Stats", main_desc$outcome_statistics)
add_s("07_PreWar_Trends", main_desc$trend_analysis)
add_s("08_Contributions", main_desc$contribution_analysis)
add_s("09_ARIMA", arima_summary)
if(RUN_SENSITIVITY){add_s("10_Sens_NoSeason",sum_s1); add_s("11_Sens_FullSlopes",sum_s2); add_s("12_Sens_AltOnset",sum_s3); add_s("13_Sens_Recent",sum_s4)}
add_s("14_Interactions", int_results)
add_s("15_Deficits", deficits)

ep <- file.path(OUTPUT_DIR,"tables","COMPLETE_ITS_REVISION_RESULTS.xlsx")
tryCatch({saveWorkbook(wb,ep,overwrite=TRUE); log_message(paste("Excel:",ep))}, error=function(e) log_message(paste("ERROR:",conditionMessage(e))))

# --- FINISH ---
log_message("\n+--------------------------------+")
log_message("|  ANALYSIS COMPLETE             |")
log_message("+--------------------------------+")
log_message(paste("Main models:", n_main))
log_message(paste("ARIMA models:", sum(!sapply(arima_results, is.null))))
log_message(paste("DPI:", FIG_DPI))
log_message(paste("Output:", OUTPUT_DIR))
log_message(paste("Excel:", ep))
log_message("\nReviewer cross-reference:")
log_message("  R1#2: Rate models -> sheet 03_Rate_Models")
log_message("  R1#3: 2020 pandemic period -> sheet 05_Period_Summary")
log_message("  R1#4: Secular trends -> sheet 07_PreWar_Trends + Sens4")
log_message("  R1#5: Quasi-Poisson + DHARMa -> sheets 01 (dispersion_p, autocorr_p)")
log_message("  R2#5: 4-period model -> pandemic=4, war=8, post=9 quarters")
log_message("  R2#9: Interactions -> sheet 14_Interactions")
log_message("  P-values formatted")
```

© 2026 Kebede HK et al. *JAMA Network Open*.

```
log_message("  Figures: PDF vector + PNG 600 DPI")
log_message("==============================")
```

© 2026 Kebede HK et al. *JAMA Network Open*.
