# Supplementary material for: Tigray War and HIV Care Cascade Entry in Mekelle, Ethiopia
Source: JAMA Netw Open. 2026 Jul 24;9(7):e2625130. doi: 10.1001/jamanetworkopen.2026.25130 (PMC13401200; doi:10.1001/jamanetworkopen.2026.25130)
Supplement: Supplement 2. — Data Sharing Statement [file jamanetwopen-e2625130-s002.pdf]

Data Sharing Statement

Kebede. Tigray War and HIV Care Cascade Entry in Mekelle, Ethiopia. *JAMA Netw Open*. Published July 24, 2026. doi:10.1001/jamanetworkopen.2026.25130

Data
Data available: No

Additional Information
Explanation for why data not available: Will individual participant data be available (including data dictionaries)? No. Due to the sensitive nature of the data involving HIV status in a conflict-affected region and ethical clearance requirements from the Torrens University Australia Human Research Ethics Committee (HREC) and the Tigray Health Research Institute Ethical Review Board, the individual-level dataset generated and analyzed during this study is not publicly available. What data will be available? Aggregate quarterly summary statistics and the statistical analysis code (R scripts) used for the interrupted time-series analysis will be made available upon reasonable request. What supporting documents will be available? Study protocol, statistical analysis plan, and analytic code will be available upon reasonable request. When will data be available (start and end dates)? Beginning 3 months after publication and ending 5 years following article publication. With whom, for what types of analyses, and by what mechanism will data be made available? Researchers who provide a methodologically sound proposal for the purposes of replicating the analyses presented in this study or conducting secondary analyses relevant to HIV care delivery in conflict-affected settings may request access by contacting the corresponding author (maymeggeltana@gmail.com). Proposals will be reviewed by the study team and institutional review boards of Torrens University Australia and the Tigray Health Research Institute. Data requestors will be required to sign a data access agreement.
